# The Continuum Between Hexagonal Planar and Trigonal Planar Geometries**

**DOI:** 10.1002/anie.202211948

**Published:** 2022-10-05

**Authors:** Martí Garçon, Andreas Phanopoulos, George A. Sackman, Christopher Richardson, Andrew J. P. White, Richard I. Cooper, Alison J. Edwards, Mark R. Crimmin

**Affiliations:** ^1^ Department of Chemistry Molecular Sciences Research Hub Imperial College London 82 Wood Lane, Shepherds Bush London W12 0BZ UK; ^2^ Chemical Crystallography Chemistry Research Laboratory 12 Mansfield Road Oxford OX1 3TA UK; ^3^ Australian Centre for Neutron Scattering, ANSTO New Illawarra Road Lucas Heights NSW, 2234 Australia; ^4^ School of Chemistry and Molecular Bioscience University of Wollongong Wollongong NSW 2522 Australia

**Keywords:** Bonding, Heterometallics, Hexagonal Planar, Hydrides, Sigma-Complex

## Abstract

New heterometallic hydride complexes that involve the addition of {Mg−H} and {Zn−H} bonds to group 10 transition metals (Pd, Pt) are reported. The side‐on coordination of a single {Mg−H} to Pd forms a well‐defined σ‐complex. In contrast, addition of three {Mg−H} or {Zn−H} bonds to Pd or Pt results in the formation of planar complexes with subtly different geometries. We compare their structures through experiment (X‐ray diffraction, neutron diffraction, multinuclear NMR), computational methods (DFT, QTAIM, NCIPlot), and theoretical analysis (MO diagram, Walsh diagram). These species can be described as snapshots along a continuum of bonding between ideal trigonal planar and hexagonal planar geometries.

## Graphical abstract

The isolation and bonding analysis of a series of heterometallic hydride complexes shows they have subtly different bonding geometries. These compounds can be described as snapshots along a continuum of bonding between trigonal and hexagonal planar geometries.

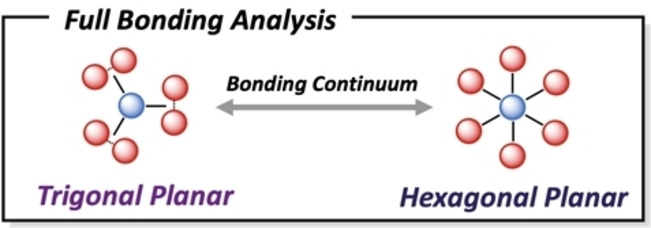

## Introduction

Six coordinate transition metal complexes typically adopt octahedral or trigonal prismatic geometries.[^1^, ^2^] The understanding of six‐coordinate transition metals is a fundamental aspect of coordination and organometallic chemistry and these compounds find applications across a number of disciplines including catalysis, bioinorganic chemistry, and materials chemistry (e.g. metal‐organic frameworks). Alternative geometries such as pentagonal pyramidal and bicapped tetrahedral have been reported but are rare.^[3]^ We focus here on a similarly underappreciated geometry for six‐coordinate transition metals; hexagonal planar. There is limited precedent for transition metal complexes with a hexagonal planar geometry.[^4^, ^5^, ^6^] For those that are known, the analysis of the bonding is often complicated by the fact they are found within transition metal clusters,[^7^, ^8^, ^9^] or in constrained coordination environments such as condensed phases[^10^, ^11^, ^12^] or the hexagonal pores of metal‐organic frameworks.^[13]^

**Figure 1:**
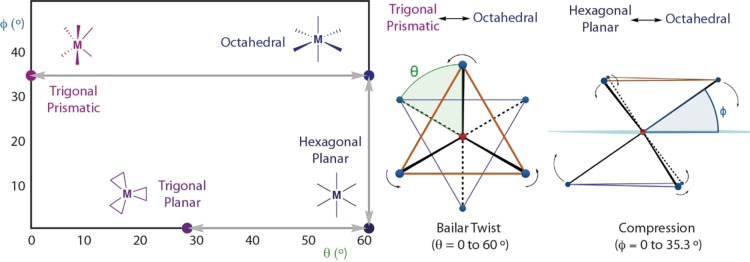
Minimal distortion pathways to interconvert octahedral, trigonal prismatic, hexagonal planar and trigonal planar geometries.

The interconversion of six‐coordinate geometries can be considered by inspecting minimal distortion pathways.^[14]^ An octahedron can be viewed as two tetrahedrons sharing a vertex. Rotation of one tetrahedron around a *C*_3_ axis results in distortion from an octahedral to a trigonal prismatic geometry. The angle associated with this rotation is often referred to as the twist angle (θ). An octahedron has a value of θ=60° while the trigonal prism has a value of θ=0°. Interconversion by rotation (Bailar twist) is a well‐established phenomenon in transition metal chemistry. A similar analysis can be conducted for the theoretical interconversion between octahedral and hexagonal planar geometries. Symmetric flattening of the tetrahedra will result in the formation of a hexagon. A planarization angle (φ) can be defined; an octahedron has a value of φ=sin^−1^(1/√3), which corresponds to 35.3°. When φ=0° a hexagonal planar geometry is obtained. Further, variation of θ from 60° to ≈30° while keeping φ=0° interconverts the hexagonal planar and trigonal planar geometries. This operation results in compression of the ligand‐ligand distances and hence θ cannot reach 0° as it would result in the superposition of the ligand atoms. This distortion formally interconverts a six‐coordinate and three‐coordinate geometry, but it is only possible for systems in which the ligands can form bonds with each other at the expense of metal‐ligand bonding (Figure 1).

A bonding continuum between the hexagonal planar and trigonal planar geometries can, therefore, be considered. *Tris*(alkene) complexes of the form [M(η^2^‐R_2_C=CR_2_)_3_]^n+^ (M=Ni, Pd, Pt, *n*=0; Cu, Ag, Au, *n*=1) are a useful example of this continuum.[^15^, ^16^, ^17^, ^18^, ^19^, ^20^, ^21^] The bonding between the transition metal atom and an array of alkene ligands in these complexes can be conceptualised in terms of the Dewar–Chatt–Duncanson model. Each metal‐alkene interaction is constructed from σ‐donation and π‐backdonation components. If the σ‐donation component of bonding dominates the bonding interaction, a trigonal planar geometry can be assigned. However, as π‐backdonation—and the metallocyclopropane contribution—becomes more important (causing a slight increase in θ), the bonding can be thought of as moving along the continuum from trigonal planar toward hexagonal planar. It is not possible for these complexes to obtain a true hexagonal planar geometry (θ=60°), in part because the carbon atoms in each alkene ligand are still connected by a σ‐bond, which precludes achieving a high value of θ.

In this paper, we describe the synthesis, characterisation, and bonding analysis of heterometallic complexes involving the coordination of {Mg−H} and {Zn−H} bonds to group 10 transition metals. *Tris*(ligated) complexes of this series are structurally related to the *tris*(alkene) complexes described above and demonstrate an alternating array of six atoms interacting with a central transition metal. Variation of the metalloligand (Zn vs Mg) results in subtle, but key, differences to the structure and bonding, describing different points along the continuum between trigonal planar and hexagonal planar geometries.

## Results and Discussion

### Coordination of Mg−H and Zn−H Bonds to Pd

**Scheme 1:**
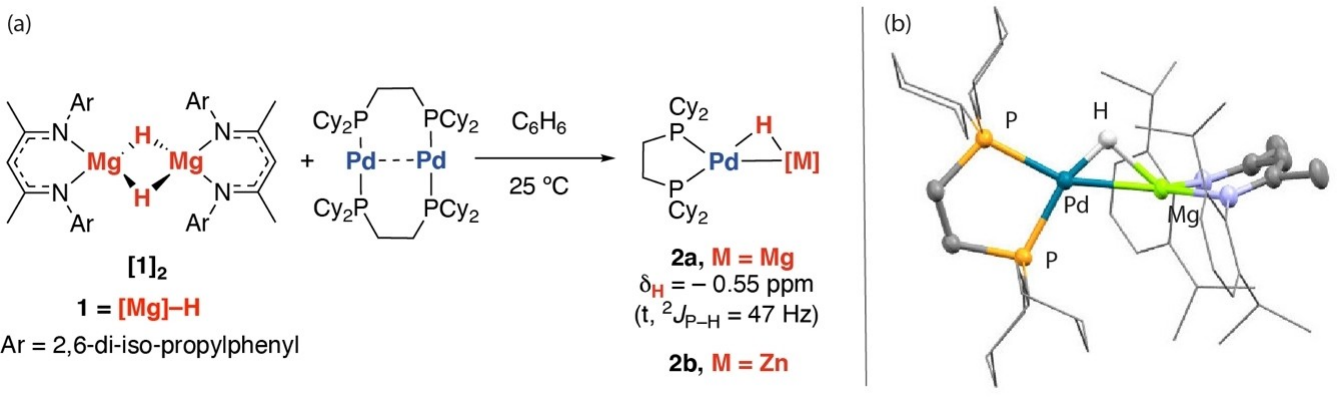
a) Synthesis of **2** **a** and b) molecular structure of **2** **a** from single crystal X‐ray diffraction study.

The magnesium hydride complex **[1]_2_** served as a starting material, a 1 : 1 reaction with [Pd(μ‐dcpe)]_2_ (dcpe=1,2‐bis(dicyclohexylphosphino)ethane) in C_6_H_6_ at 25 °C resulted in clean formation of **2** **a** (Scheme 1). **2** **a** could be isolated as a yellow crystalline solid in 68 % yield and displayed a diagnostic hydride resonance at δ=−0.55 (t, ^2^ *J*_P−H_=47 Hz) ppm in the ^1^H NMR spectrum with coupling occurring to a single ^31^P environment observed at δ=50.4 ppm. **2** **a** is fluxional in solution and undergoes H/D‐exchange at the hydride site in C_6_D_6_.

**2** **a** is a rare example of structurally characterised complex involving coordination of a single Mg−H unit to the transition metal.^[22]^ **2** **a** was characterised by standard techniques as well as neutron diffraction. The structure of **2** **a** contains a planar array of Pd, Mg, H and P atoms.^[23]^ The Pd−P bond lengths are asymmetric with that *trans* to the hydride being ≈0.1 Å shorter than that *trans* to Mg; a likely consequence of the strong *trans*‐effect exerted by the main group atom. The Pd−Mg distance of 2.5469(8) Å is within the sum of covalent radii (Pauling, 2.64 Å; Pyykkö, 2.59 Å).^[24]^ Neutron diffraction studies confirmed the location of the hydride site and allowed the Mg−H and Pd−H bond lengths to be determined as 1.89(5) and 1.70(4) Å respectively. The Pd−H−Mg angle is 89(2)°. We recently reported the zinc analogue of this complex, **2** **b**.^[25]^ Further insight into the bonding in these complexes was obtained through computational methods.^[26]^

Natural Bond Orbital (NBO) analysis was carried out on **2** **a** and **2** **b**. Natural Population Analysis (NPA) returns atomic charges which provide insight into the degree of charge separation and ionic character to the bonding. In all cases negative charge accumulates on the transition metal (Pd) and hydride ligands, while the main group metals (Mg, Zn) show a positive charge. The degree of charge separation is greater for **2** **a** than **2** **b**, consistent with a higher ionic character. The negative charge accumulation at Pd is inconsistent with an increase in formal oxidation state upon coordination, rather **2** **a** and **2** **b** are likely best described as Pd^0^ complexes. These data are complemented by Wiberg Bond Indices (WBI), which provide a measure of the covalent bond order; an entirely covalent single bond between two atoms is expected to show a WBI of 1. Both **2** **a** and **2** **b** show modest WBIs between all three atoms involved in the 3‐centre,2‐electron interaction. WBIs are smaller than those of the parent hydrides **1** and **3** **a**. These calculations suggest that Mg−H and Zn−H bonding is retained, but weakened, on coordination and supports the assignment of **2** **a** and **2** **b** as σ‐complexes (Table 1).

**Table 1:** NPA charges, Wiberg Bond Indices and selected QTAIM data (*ρ* (r) and ∇^2^ *ρ* (r) given in a.u.) for **1**, **2** **a**–**b**, **3** **a**, **5** and **6** **a**–**b**. M^1^=Mg, Zn, M^2^=Pd, Pt. information. Note *ρ* (∇^2^ *ρ*) for **5**, **6** **a** and **6** **b** is averaged over three values. Full details of these calculations are provided in the Supporting Information.

		1	3a	2a	2b	5	6a	6b
***NPA charges***	*M* ^*1*^	1.61	1.38	1.54	1.26	1.39–1.43	1.66–1.68	1.61–1.67
	*M* ^*2*^	–	–	−0.23	−0.16	−0.26	−0.44	−0.56
	*H*	−0.72	−0.54	−0.51	−0.41	−0.46–−0.50	−0.59–−0.62	−0.52–−0.58
***WBI***	*M* ^*1*^−*M* ^*2*^	–	–	0.23	0.31	0.14–0.18	0.10–0.12	0.11–0.17
	*M* ^*1*^−*H*	0.43	0.61	0.21	0.31	0.30–0.31	0.11–0.13	0.09–0.11
	*M* ^*2*^−*H*	–	–	0.39	0.37	0.25–0.28	0.28–0.31	0.30–0.37
***ρ (*** **∇^2^ *ρ*)**	*M* ^*1*^−*M* ^*2*^	–	–	–	0.06 (0.11)	–	0.03 (0.09)	0.04 (0.10)
	*M* ^*1*^−*H*	0.06 (0.18)	0.12 (0.13)	0.04 (0.11)	0.07 (0.12)	0.07 (0.13)	–	–
	*M* ^*2*^−*H*	–	–	0.11 (0.17)	0.11 (0.15)	0.10 (0.17)	0.10 (0.17)	0.12 (0.11)

**Figure 2:**
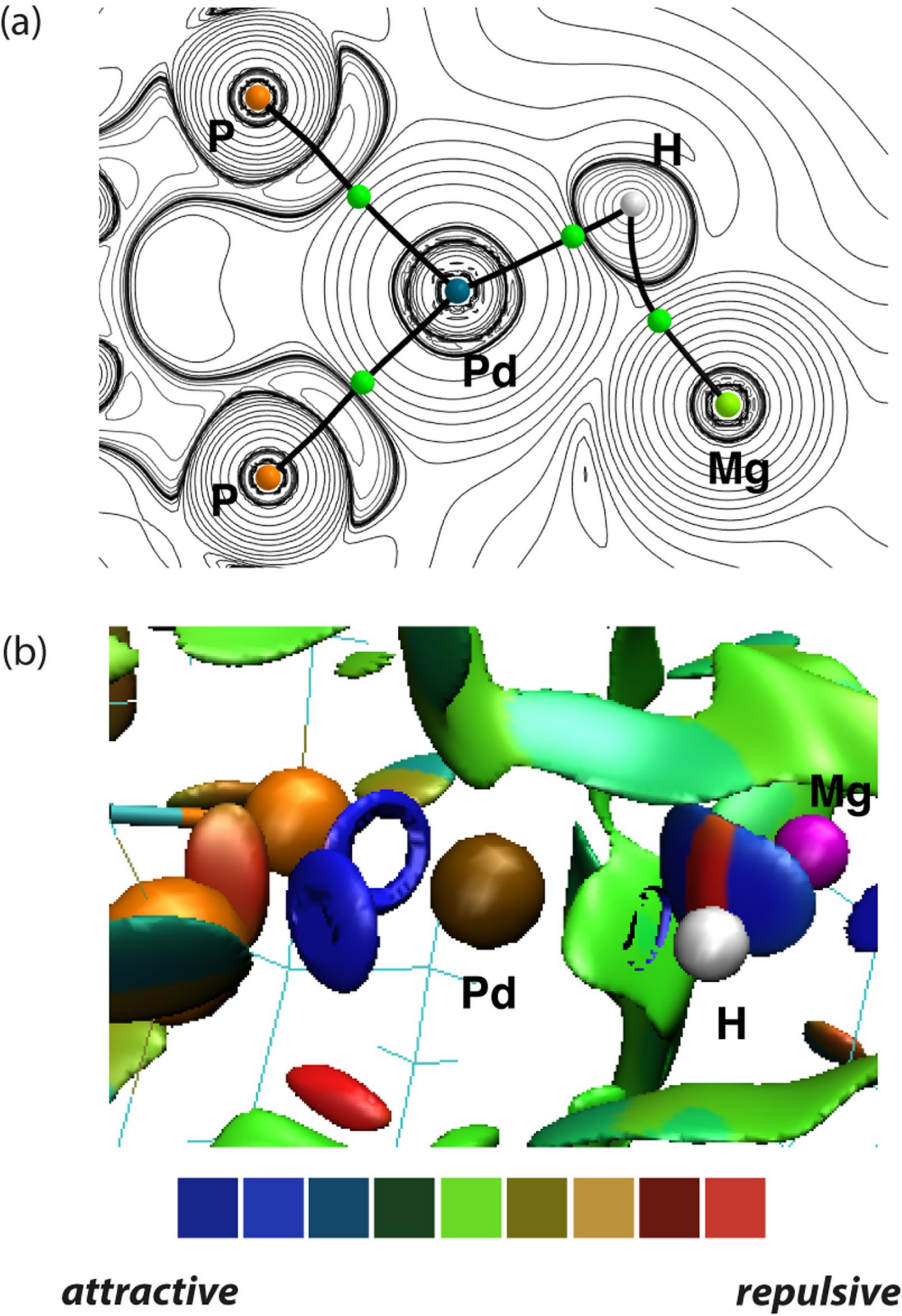
QTAIM molecular graph for a) **2** **a** showing key bond critical points (BCPs, green points). Associated BCP electron densities, *ρ* (r) and ∇^2^ *ρ* (r), are given in Table 1. b) NCIPlot for **2** **a**.

Quantum Theory of Atoms in Molecules (QTAIM) analysis was also used to understand the topology of the electron density in **2** **a** and **2** **b**. For example, QTAIM analysis returns bond paths between Pd and H atoms and Mg and H atoms in **2** **a** (Figure 2a). Each of these bond paths has an associated bond critical point (BCP). The electron density at the BCP, *ρ* (r), provides insight into the strength of the chemical bond, with larger values reflecting higher bond order, while the Laplacian of the electron density ∇^2^ *ρ* (r) is a second‐derivative which describes the sum of the three curvatures of the electron density at this point. Small *ρ* (r) values alongside ∇^2^ *ρ* (r) *>0* calculated for **2** **a** and **2** **b** are consistent with weak, primarily ionic interactions between the Pd, Mg and H nuclei. Again *ρ* (r) values at the BCP between the Zn and H for **2** **b** are larger than Mg and H for **2** **a**, suggesting a stronger covalent interaction.

Non‐Covalent Interaction (NCI) plots provide a qualitative approach to visualise non‐covalent interactions in space. This approach is complementary to NBO and QTAIM analysis in that it captures electrostatic interactions. NCI plot calculations are conveniently derived from geometrical inputs, and they provide visual representations of attractive and repulsive interactions between atoms sites. The NCI plot on the key bonding motif of **2** **a** shows an oblate disc of alternating attractive (blue) and repulsive interactions (red) between the Mg−H units and Pd depicting attractive interactions between the Mg and H, and Pd and Mg atoms (Figure 2b).

**Figure 3:**
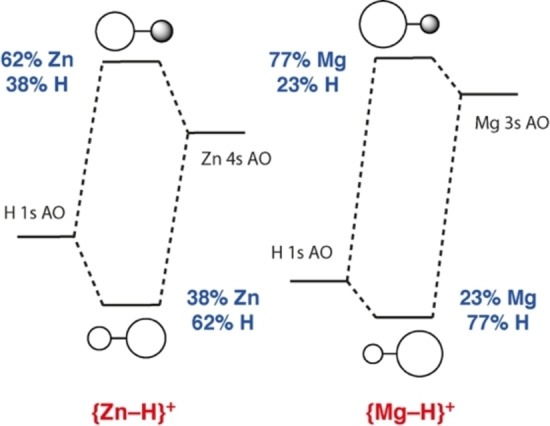
AO contributions to the σ and σ*‐orbitals of {Zn−H}^+^ and {Mg−H}^+^.

The bonding in **2** **a** and **2** **b** can also be considered in terms of molecular orbital theory. DFT calculations on simplified models in which **1** and **3** **a** are approximated by naked {Mg−H}^+^ and {Zn−H}^+^ fragments provide a qualitative insight into the bonding of the complexes. It is worth comparing the {Mg−H}^+^ and {Zn−H}^+^ fragments to archetypical dihydrogen {H−H} ligands. The differences in electronegativity (Δχ_p_=0.55, H *vs*. Zn; Δχ_p_=0.89 H *vs*. Mg) not only increase the ionic component of the bonding but also influence the relative size of the coefficients of the bonding and anti‐bonding orbitals constructed from 1 *s* and 3 *s*/4 *s* AOs (Figure 3). For the covalent component of the bonding in the corresponding σ‐complexes, the interaction of the ligand SALCs with metal‐based orbitals can be conceptualised in terms of the Dewar–Chatt–Duncanson model, being constructed from donation and back donation components. However, unlike the prototypical H−H coordination, the interaction is not symmetric. Based on the relative size of the coefficients on the MOs of the {Zn−H}^+^ and {Mg−H}^+^ fragments, σ‐donation occurs primarily from the H atom, while back donation occurs primarily to the Zn or Mg atom. DFT calculations on the full systems indicate that the acceptor σ* orbitals are the LUMO+1 and have similar energies for both **1** (+0.073 eV) and **3** **a** (+0.074 eV).

**Figure 4:**
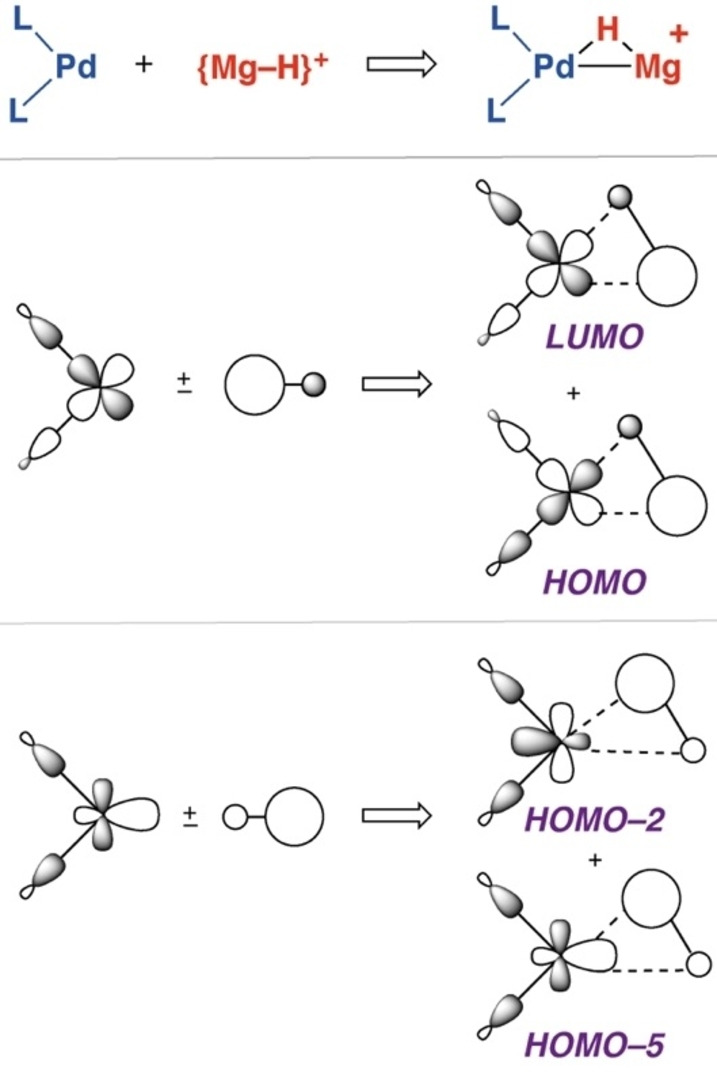
Key a′ symmetry orbital interactions in a model of **2** **a**.

A molecular orbital diagram for a simple model of **2** **a** was constructed from PdL_2_ (L_2_=κ^2^‐(PH_3_)_2_C_2_H_4_) and {Mg−H}^+^ fragments. Within this model, the approach of the bent PdL_2_ fragment to the {Mg−H}^+^ unit takes place in the equatorial (xy) plane and generates a *C*_S_ symmetric molecule (idealised symmetry). The *d*_xy_ and *d*_x2‐y2_ based orbitals (a′ symmetry) are properly oriented to interact with the σ*‐(Mg−H) and σ‐(Mg−H) orbitals (a′ symmetry), respectively. These are the key bonding interactions in this complex. In contrast, the *d*_xz_ and *d*_yz_ based orbitals (a′′ symmetry) are non‐bonding. The *d*_z2_ based orbital (a′ symmetry) could also overlap with the σ‐(Mg−H) orbital, but the overlap is small and to a first approximation can be ignored (Figure 4).

In summary, the key bonding interaction in **2** **a** and **2** **b** both involve 3‐centre,2‐electron bonding between Pd, Mg (or Zn) and H atoms. NBO, QTAIM and NCI Plot calculations return a bonding picture in which the Mg−H (or Zn−H) bond coordinates to Pd, but is not broken upon coordination. As such these species are best described as σ‐complexes. The experimental data and computational analysis of **2** **a** and **2** **b** are important in benchmarking more complex bonding interactions; for example, complexes involving the coordination of three {Mg−H} or {Zn−H} bonds to group 10 metals.

### Hexagonal Planar and Trigonal Planar Complexes

**Scheme 2:**
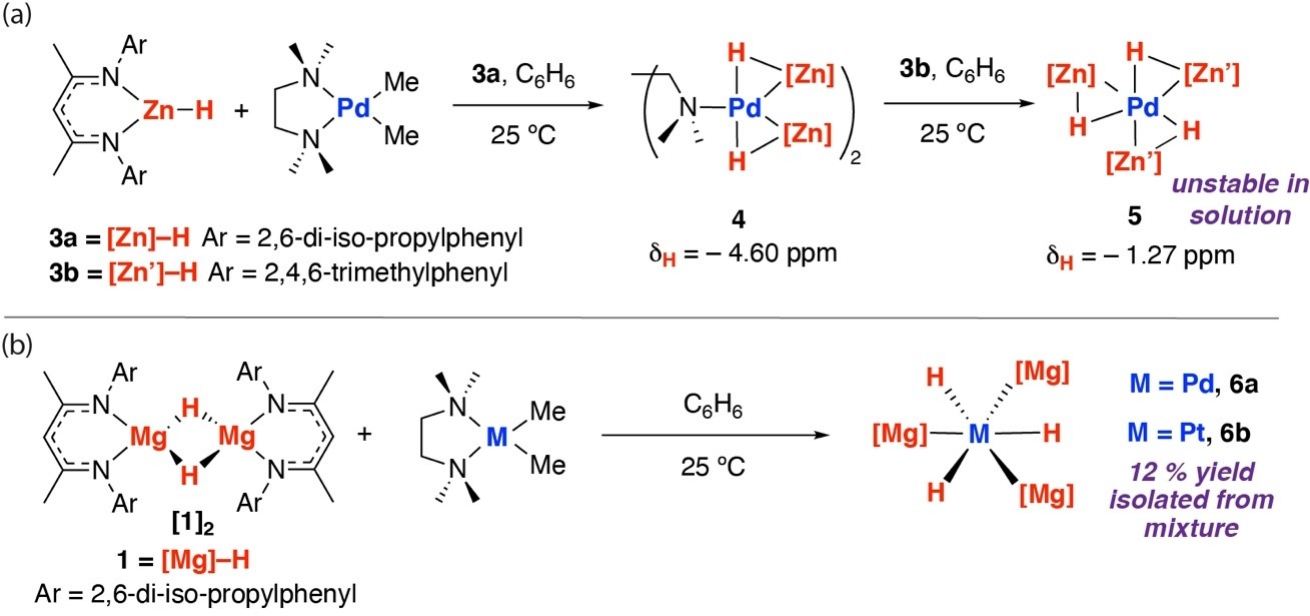
a) Stepwise synthesis of **5** and b) synthesis of **6** **b** and isolation from a mixture.

The heteroleptic *tris*(zinc) complex **5** was prepared by a two‐step procedure via **4** as an intermediate (Scheme 2). **4** was isolated from the reaction of two equiv of **3** **a** with [Pd(Me)_2_(κ^2^‐TMEDA)] (TMEDA=*N*,*N*,*N′*,*N′*‐tetramethylethylenediamine).^[27]^ DOSY experiments, titration experiments, and VT NMR analysis suggest that the TMEDA ligand of **4** is labile and can reversibly dissociate in solution.^[28]^ Although reaction of **4** with further equivalents of **3** **a** did not lead to a homoleptic *tris*(zinc) complex, addition of the less sterically demanding **3** **b** to **4** resulted in formation of the heteroleptic analogue **5** in low yield. C_6_D_6_ solutions containing **5** demonstrated a single resonance at δ=−1.27 ppm consistent with a fast‐exchanging hydride resonance. **5** is unstable in solution and rapidly decomposes in less than 2 h at 25 °C. The purification of this compound was extremely challenging and single crystals could only be isolated on one occasion.

We have previously reported that the reaction of [Pd(Me)_2_(κ^2^‐TMEDA)] with **[1]_2_** affords directly the hexagonal planar palladium complex **6** **a**.^[29]^ The analogous reaction of [Pt(Me)_2_(κ^2^‐TMEDA)] with **[1]_2_** in C_6_H_6_ at 25 °C for 4 days yielded a mixture from which the hexagonal planar complex **6** **b** could be isolated as a minor product in 12 % yield (Scheme 2). **6** **b** displays a diagnostic hydride resonance at δ=−2.88 ppm with ^195^Pt satellites (^1^ *J*_195Pt‐1H_=1080 Hz) in the ^1^H NMR spectrum. The corresponding peak in the ^195^Pt NMR spectrum is observed as a distinct quartet at δ=−6159.1 ppm. These data suggest that all hydride sites of **6** **b** are equivalent in solution. The *J* value is higher than the current range ^1^ *J*_195Pt‐1H_=700–800 Hz established for Pt complexes with coordinated Mg−H and Zn−H σ‐bonds.[^25^, ^29^]

**6** **b** showed evidence for a terminal ν(Pt−H) stretch in the infrared spectrum at 1706 cm^−1^. The assignment of this stretch was unequivocally confirmed by preparation of the deuterated isotopomer d^3^‐**6** **b** (Figure S22). Vibration analysis on models of **5**, **6** **a** and **6** **b** calculated by DFT suggests that distinct symmetric and asymmetric stretches are predicted for both the Pd−H (and Pt−H) and Mg−H (and Zn−H) vibrations. For **6** **b**, the asymmetric ν(Pt−H) stretch is expected to be IR active and calculated to occur at, an uncorrected value of, 1814 cm^−1^. The corresponding ν(Mg−H) stretches of **6** **b** are predicted to occur at much lower frequency (<500 cm^−1^) consistent with a small force constant between these atoms (see Supporting Information).

In the solid‐state, **5** displays an apparent trigonal planar geometry.^[23]^ Three Zn−H units are coordinated to Pd in a planar environment. The Pd−Zn bond lengths range from 2.4660(6) to 2.4768(6) Å and are similar to, or slightly longer, than the sum of the covalent radii (Pauling, 2.53 Å; Pyykkö, 2.38 Å).[^24^, ^25^] While the hydride positions from X‐ray diffraction studies should be treated with caution, the location of these sites has been supported by DFT studies. Furthermore, for **2** **a** we have shown that the single crystal X‐ray diffraction data match well with those collected from neutron diffraction. Both the Pd−H (1.52(4)–1.72(7) Å XRD; 1.69–1.71 Å DFT) bond lengths and Zn−H (1.64(4)–1.87(7) Å XRD; 1.77–1.81 Å DFT) bond lengths of **5** are in the range established for σ‐zincane complexes.[^30^, ^31^] The Zn−Pd−H bond angles around the trigonal plane can be grouped into two sets: a very acute set (40(15)–49(2)° XRD; 45–47° DFT) and a less acute set (63(2)–85(15)° XRD; 67–78° DFT). The asymmetry of the hydride positions is due to association of individual hydrides with a single zinc centre. We have previously structurally characterised the palladium analogue **6** **a** and described its geometry as hexagonal planar.^[30]^ **6** **b** also contains an alternating array of hydride and magnesium ligands arranged in a planar geometry around Pt.^[23]^ The average H−Pt−Mg angle is 60.1°; the largest deviation away from this angle is 4°. The Pt−Mg bond lengths range from 2.5547(11) to 2.5762(11) Å and are well within the sum of the covalent radii (Pauling, 2.65 Å; Pyykkö, 2.62 Å).^[24]^ The biggest distortion of any ligand out of the hexagonal plane is <10°. The Pt−H bond lengths are short (1.61(4)–1.79(4) Å XRD; 1.69–1.71 Å DFT) while the Mg−H separations are extremely long (2.19(4)–2.40(5) Å XRD; 2.18–2.43 Å DFT).

**Figure 5:**
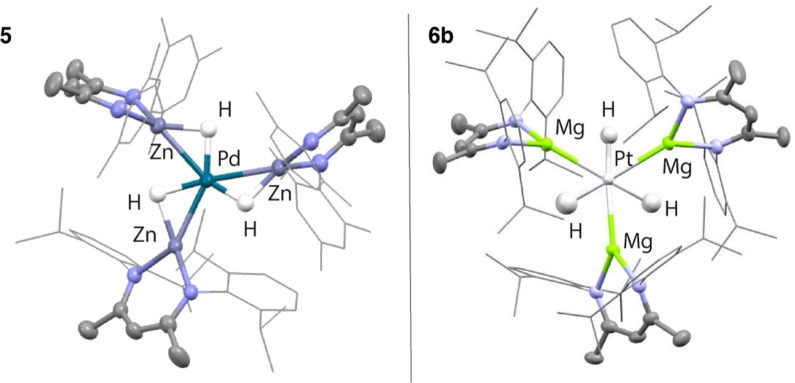
Molecular structures of **5** and **6** **b** from single crystal X‐ray diffraction studies.

For comparison, terminal Mg−H bonds have been characterised by X‐ray diffraction with bond lengths of 1.75(7) and 1.85(3) Å[^32^, ^33^] while those bridging between two Mg sites supported by the β‐diketiminate ligand system typically range from ≈1.8–2.0 Å.[^34^, ^35^] DFT calculations on **1** returns a Mg−H bond length of 1.71 Å for the terminal hydride in the parent compound. This is much shorter than the range established for **6** **a** and **6** **b**. In contrast, the calculated Zn−H distance of **3** **a** of 1.55 Å is closer to the range established for **5**. The data suggest that the Mg−H bonds are stretched to a greater degree than Zn−H bonds upon coordination to Pd or Pt (Figure 5).

The apparent difference in the structures of **5** and **6** **a/b** is further supported by DFT calculations. Analysis of the NPA charges suggests that the trigonal planar zinc analogue **5** shows less charge separation than the hexagonal planar structure **6** **a** (Table 1). The WBIs reflect subtly different bonding within the trigonal or hexagonal plane. For the trigonal planar structure, the covalent interactions between the Zn−H (0.30–0.32) and Pd−Zn (0.14–0.18) bonds are similar, reflecting the σ‐complex character. Indeed, these WBI values are almost identical for **2** **b**, which supports the formulation of **5** as a tris‐σ‐complex. For the hexagonal planar structures, the increased ionic contribution to bonding begins to mask the important covalent bonding interactions. Nevertheless, in the case of **6** **b** the metal‐ligand interactions are more significant than the ligand‐ligand interactions, Pt−Mg (0.11–0.17) >Mg−H (0.09–0.11). For **6** **a** the values are closer. In both cases, the WBI values are markedly different than those of **2** **a**, illustrating a difference in bonding.

**Figure 6:**
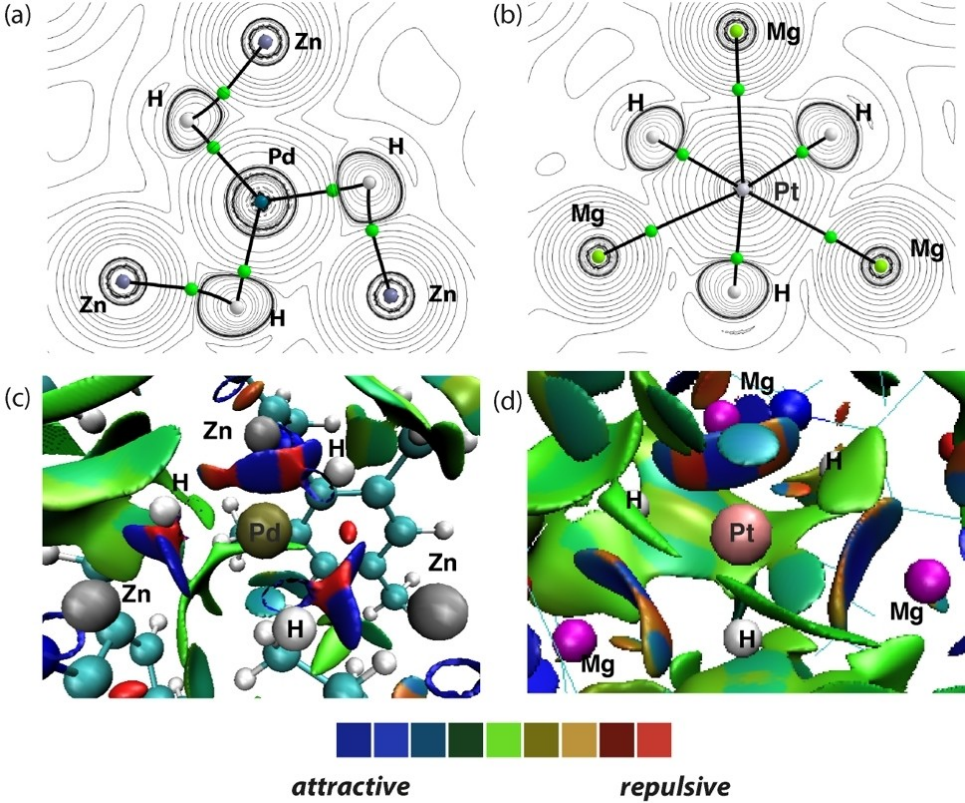
QTAIM molecular graphs for a) **5** **a** and b) **6** **b** showing key bond critical points (BCPs, green spheres). Associated BCP electron densities, *ρ* (r) and ∇^2^ *ρ* (r), are given in Table 1. c) NCIPlot for **5**. d) NCIPlot for **6** **b**.

QTAIM also exposed differences in bonding between **5** and **6** **a/b**. For **5**, QTAIM calculations reveal a molecular topology that is consistent with a *tris*(sigma) complex with bond critical points observed between pairs of Zn and H atoms in a trigonal arrangement around Pd. In contrast, QTAIM data on **6** **b** are consistent with a hexagonal planar geometry in which the central Pt atom interacts with six ligands organised in a hexagonal arrangement in the equatorial plane (Figure 6a–b). NCIPlot for **5** shows strong attractive interactions (deep blue) between three sets of Zn−H ligands and Pd. Each Zn is associated with one H atom. The picture is different for **6** **b**: Three discs capture the key bonding interactions, each disc shows a strongly attractive Pd−Mg interaction (deep blue) flanked by a weaker, less attractive, non‐covalent interactions between the Mg and the adjacent two H atoms (teal).

**Figure 7:**
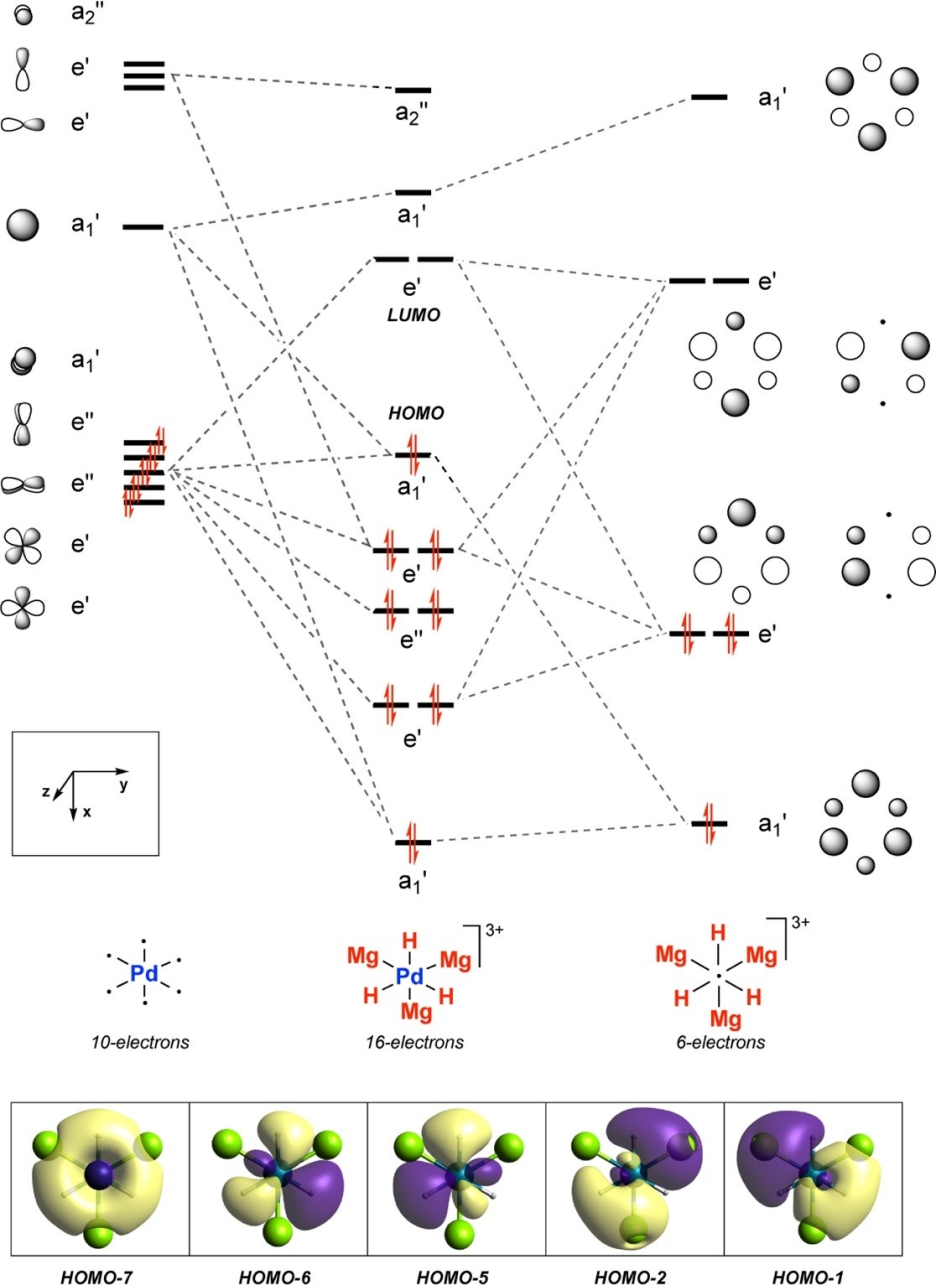
Qualitative MO diagram for a simplified model of **6** **a**.

The qualitative MO diagram for a *D*_3h_ symmetric model of **6** **a** is presented in Figure 7. This geometry is stabilised by a 16‐electron configuration. The symmetry adapted ligand combinations (SALCs) are constructed from six AOs of Mg and H and are reminiscent of those of benzene with a key difference being the effect of the electronegativity on the relative size of the coefficients on the Mg and H atoms. Two sets of doubly degenerate ligand SALCs (e′ symmetry) can engage with Pd *d* orbitals to generate orbitals arising from multicomponent mixing. The HOMO−6 and HOMO−5 are bonding combinations of the *d_xy_* and *d*_*x2‐y2*_ orbitals with the higher e′ ligand set. The LUMO and LUMO+1 are the corresponding antibonding combinations. The remaining e′ set, the HOMO−1 and HOMO−2, arises from overlap from the *d_xy_* and *d*_*x2‐y2*_ Pd orbitals with the lower e′ ligand set, with a non‐negligible contribution from the *p_x_* and *p_y_* Pd orbitals. As expected, the e′′ orbitals (*d_xz_* and *d_yz_*) are non‐bonding. Further insight can be gained by dissecting the key e′ orbitals down to their AO contributions. The lower e′ set (HOMO−5 and HOMO−6) has an almost negligible contribution from Mg *s* AOs (55 % Pd *d*, 37 % H *s* AOs) and along with the HOMO−7 captures the Pd−H bonding interactions. In the second bonding e′ set (HOMO−1 and HOMO−2) there is a more even contribution of AOs (27 % Pd *d*, 29 % H *s*, 24 % Mg *s*) to the MOs; these orbitals are bonding between the two metals and are the origin of the covalent contribution to the Pd−Mg bonds. A closely related MO diagram can be constructed for a *C*_3h_ symmetric model of **5** (Supporting Information, Figure S15). The subtle differences between these geometries are exposed by considering their interconversion.

### The Continuum between Hexagonal Planar and Trigonal Planar

**Figure 8:**
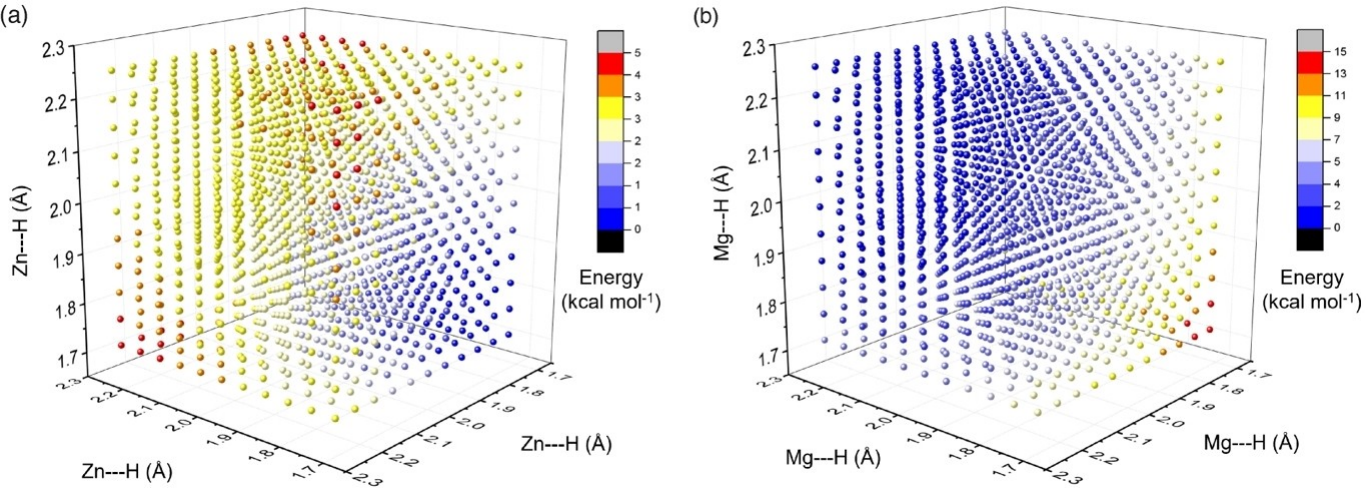
Calculated potential energy surfaces showing the compression and elongation of M−H bonds for a) **5** (M=Zn) and b) **6** **b** (M=Mg).

Using DFT methods, the Zn−H or Mg−H distances in models for **5** and **6** **b** can be stretched or compressed to understand the equilibrium bond length and the energetic cost for deviation from equilibrium values (Figure 8). The results of these calculations can be plotted on a 4‐D potential energy surface (PES). For **5**, the only minimum on the PES is the *tris*(sigma) complex. Stretching of each of the Zn−H bonds comes with a small but real energy cost. The highest energy structures on the PES involve the elongation of two Zn−H bonds while keeping one compressed. The model of **6** **b** essentially shows the opposite trend. The only minimum on the PES is the hexagonal planar complex. The equilibrium Mg−H distances are extremely long and compressing them costs energy. The highest energy structures now involve symmetric compression of all three Mg−H distances from the equilibrium geometry toward a trigonal planar structure.

The general radial shape of both PES suggests that symmetric compression and elongation of the three M−H bonds is the lowest energy pathway to interconvert the trigonal and hexagonal planar geometries. Hence, these surfaces represent the bonding continuum between hexagonal planar and trigonal planar geometries. Complexes **5** and **6** **a**/**6** **b** are experimental snapshots around both ends of this continuum.

**Figure 9:**
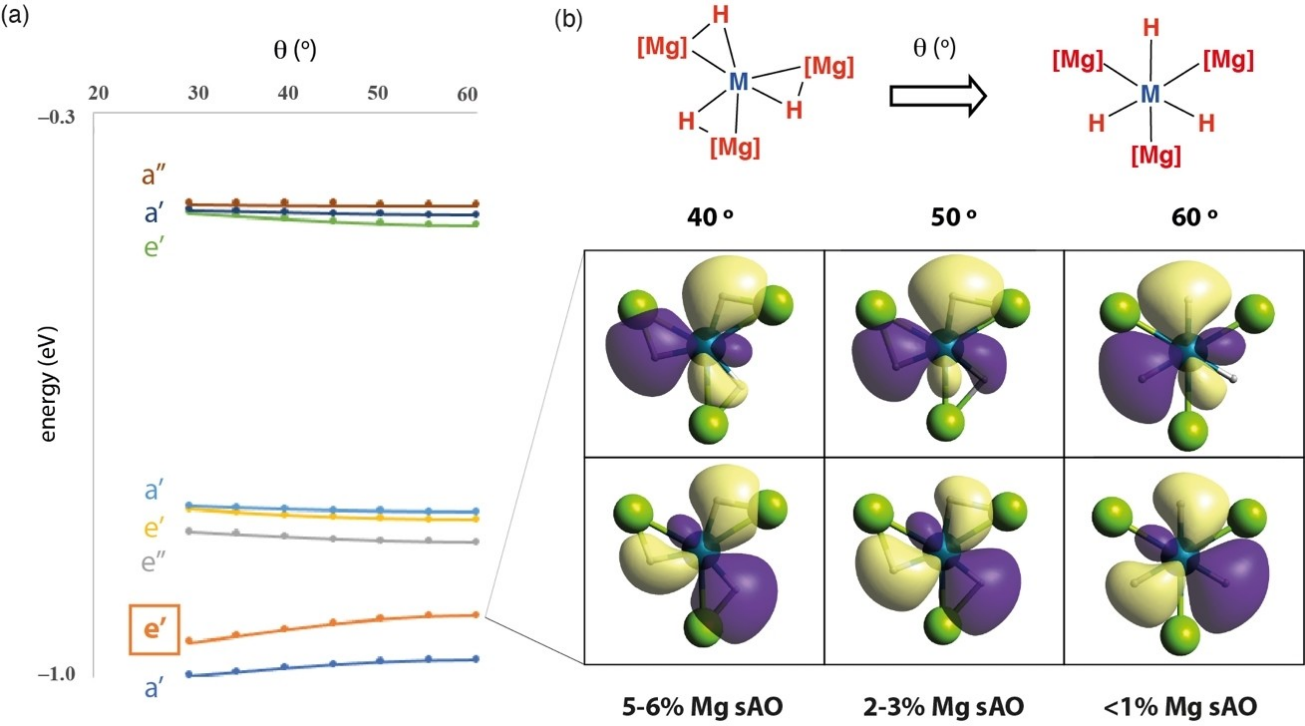
a) Walsh diagram and b) snapshots at different values of θ for the key e′ set (HOMO−5 and HOMO−6) of orbitals of a model of **6** **a**, showcasing the increase in Mg−H interactions upon compression.

The Walsh diagram for the simultaneous symmetric compression of three Mg−H units (variation of θ—Figure 1) in a model of **6** **a** provides further insight into the changes in bonding between hexagonal planar (*D*_3h_) and trigonal planar (*C*_3h_) geometries (Figure 9a). The stabilisation of the key e′ set (HOMO−5 and HOMO−6) upon distortion can be rationalised in terms of the formation of bonding σ‐(Mg−H) interactions. The antibonding e′ set (LUMO and LUMO+1) is destabilised due to the formation of the corresponding anti‐bonding interactions. The hexagonal planar geometry has few MOs with direct Mg−H interactions. For example, the HOMO−5 and HOMO−6 have negligible Mg *s* AO contributions when θ=60°. The Mg *s* AO contribution increases for this e′ set as θ decreases.^[36]^ This can be understood qualitatively by a visual inspection of snapshots of the MOs from points along the pathway of deformation (Figure 9b). These snapshots show that deformation from the hexagonal planar to trigonal planar geometry increases Mg−H bonding in the equatorial plane.

The Walsh diagram suggests that the trigonal planar geometry should be favoured based on covalent bonding arguments. The key e′ bonding orbitals are lower in energy in this geometry than in the hexagonal planar. This covalent (orbital) contribution is more important for **5** than for **6** **a**/**6** **b**. Natural resonance theory analysis, conducted as part of NBO calculations, suggests that models of these complexes have higher covalent contribution to the bonding when M=Zn, compared to M=Mg. We speculate that, hexagonal planar configurations might be more likely to emerge when the ionic contribution to the bonding is large. This may reduce the energy cost in deforming the complex to θ=60°; the weakened covalent interactions between ligands should allow the Mg atoms to translate to a position where they can maximise electrostatic interactions to the transition metal fragment.

## Conclusions

In summary, we describe the synthesis, characterisation, and bonding analysis of a series of complexes involving the coordination of three {Mg−H} bonds or three {Zn−H} bonds to a central Pd or Pt atom. These species possess a near perfectly flat array of six atoms surrounding the central transition metal and describe points along a continuum between trigonal planar and hexagonal planar geometries. While the Zn analogue is best described as trigonal planar, the Mg analogues display a hexagonal planar geometry. The key MO interactions that describe the hexagonal planar geometry are derived from combination of the *d*_xy_ and *d*_x2‐y2_ orbitals with suitable (e′ symmetry) ligand SALCs constructed from 1*s* H AOs and 3*s* Mg AOs. Between them, these molecular orbitals describe aspects of metal‐metal bonding in the hexagonal plane. Consideration of the Walsh diagram that connects hexagonal planar (*D*_3h_) and trigonal planar (*C*_3h_) geometries suggests that deformation should be a low energy process that occurs with a quantifiable increase in the contribution to ligand‐ligand bonding at the expense of metal‐ligand bonding. Our analysis also suggests that the hexagonal planar extreme of the bonding continuum will be favoured only in systems with large ionic contributions to the bonding. This insight will hopefully contribute to the future development of complexes with unusual planar geometries by rational design.

## Conflict of interest

The authors declare no conflict of interest.

1

## Supporting information

As a service to our authors and readers, this journal provides supporting information supplied by the authors. Such materials are peer reviewed and may be re‐organized for online delivery, but are not copy‐edited or typeset. Technical support issues arising from supporting information (other than missing files) should be addressed to the authors.

Supporting Information

Supporting Information

Supporting Information

Supporting Information

Supporting Information

Supporting Information

## Data Availability

The data that support the findings of this study are available in the supplementary material of this article.
