# Supplementary material for: The Continuum Between Hexagonal Planar and Trigonal Planar Geometries
Source: Angew Chem Int Ed Engl. 2022 Oct 5;61(44):e202211948. doi: 10.1002/anie.202211948 (PMC9828084; doi:10.1002/anie.202211948)
Supplement: Supplementary file 2 — Supporting Information [file ANIE-61-0-s003.pdf]

Angewandte Chemie
Eine Zeitschrift der Gesellschaft Deutscher Chemiker

Supporting Information

The Continuum Between Hexagonal Planar and Trigonal Planar Geometries

*M. Garçon, A. Phanopoulos, G. A. Sackman, C. Richardson, A. J. P. White, R. I. Cooper, A. J. Edwards, M. R. Crimmin**

Table of Contents

1. General Experimental 3
2. Synthesis of Starting Materials 4
3. Synthesis of Heterometallic Hydrides 5
4. Products from the Reaction of [PdMe$_2$(κ^2-TMEDA)] and **3a** 14
   4.1. NMR spectroscopy 14
   4.2. X-ray Diffraction Study of Side-Product 16
5. Crystallographic Data – X-ray Diffraction 17
6. Neutron single-crystal Laue Diffraction studies 21
7. Discussion of Ternary Hydrides 22
8. Density Functional Theory Calculations 23
   8.1. Computational Methods 23
   8.2. Comparison of Bonding Parameters for Model and Full Systems 24
   8.3. Molecular Orbital Analysis 26
   8.4. Symmetry Elements for the Hexagonal Planar Geometry 30
   8.5. PES for Hexagonal and Trigonal Planar Geometries 31
   8.6. Vibrational analysis for Hexagonal and Trigonal Planar Geometries 33
9. XYZ Coordinates 35
10. References 80

1. General Experimental

Unless otherwise specified, all manipulations were carried out using standard Schlenk and glovebox techniques, under inert atmosphere (nitrogen or argon). A MBRAUN Labmaster glovebox was employed operating with concentrations of H_2O and O_2 below 0.1 ppm. Anhydrous solvents were obtained from a Grubbs type SPS system and stored over activated 3Å molecular sieves under inert atmosphere. Alternatively, they were dried using molecular sieves and degassed by freeze-pump-thaw procedures. Stable liquid organic reagents were dried over 3Å molecular sieves and degassed by freeze-pump-thaw cycles before use. All other reagents were obtained from commercial suppliers (Sigma-Aldrich, Alfa Aesar, Fluorochem) and used without further purification. The synthesis and characterisation of the β-diketiminate ligand,[1] compounds **2b** and **3a**,[2] **3b**[3] and **6a**[4] have been described elsewhere. **[1]**$_2$ was prepared by modified literature procedures as detailed below. $^{13}CH_3I$[5] and $^{13}CH_3Li$[6] were synthesised sequentially from $^{13}CH_3OH$ (99 atom % ^{13}C, Sigma-Aldrich) as described in the literature. [PdMe$_2$(κ^2-TMEDA)] and [Pd($^{13}CH_3$)$_2$(κ^2-TMEDA)] were synthesised from [PdCl$_2$(κ^2-TMEDA)] and MeLi or $^{13}CH_3Li$, respectively, as described.[7]

^1H, ^2H, $^{13}C\{^1H\}$, $^{31}P\{^1H\}$ and ^{195}Pt NMR spectra and two-dimensional experiments (*e.g.* COSY, NOESY, DOSY, HSQC, HMBC, ^1H-^{31}P HMBC, ^1H-^{195}Pt-HMQC) were conducted in J. Young's NMR tubes on BRUKER 400 MHz or 500 MHz spectrometers. Chemical shifts (δ) were referenced to internal solvent resonances. Data was processed using the MestReNova or TopSpin software packages. The coupling constants (*J*) are reported in Hertz (Hz). The following abbreviations are used to define multiplicities: s (singlet), d (doublet), t (triplet), q (quadruplet), quint. (quintet), sept. (septet), dd (doublet of doublets), m (multiplet), br s (broad signal).

Single crystal X-ray data for compounds **2a**, **4**, **5** and **6b** were collected using an Agilent Xcalibur PX Ultra A diffractometer, and the structures were refined using the SHELXTL and SHELX-2013 program systems.[8] The Laue single-crystal neutron diffraction studies reported here were undertaken by mounting each crystal to the φ axis of the KOALA diffractometer standing at the end of the TG3 supermirror guide at the OPAL nuclear reactor, ANSTO. The crystals were cooled in the open flow of an Oxford Cryosystems COBRA™ 173 K nitrogen stream. Detection was by means of neutron sensitized 'Niimura special' image plates mounted to the fixed radius cylindrical detector drum. Details of the individual data collection and reduction procedures are provided in the relevant CIFs. All crystals are of low symmetry and wherever possible data from two separate orientations of the unit cell with respect to the φ axis of the instrument were recorded to ensure full coverage of the unique fraction of reciprocal space.

2. Synthesis of Starting Materials

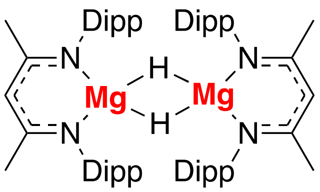

Synthesis of [1]$_2$: The ligand {(DippNCMe)$_2$CH}H (Dipp = 2,6-di-iso-propylphenyl, 5.0 g, 12 mmol, 1 equiv.) was dried under vacuum for 2-3h, and then dissolved in anhydrous toluene (*ca*. 60 mL). *n*-BuLi (7.9 mL, 1.6 M sol. in hexanes, 12.6 mmol, 1.05 equiv.) was then added dropwise at -78 °C with a syringe. The reaction was allowed to reach 25 °C and was stirred for a further 1h. MeMgBr (4.2 mL, 3 M sol. in diethyl ether, 12.6 mmol, 1.05 equiv.) was then added dropwise (during around 5 minutes) *via* syringe at 0°C. The mixture was stirred at 25 ºC overnight. It was then filtered *via* cannula and the solvent removed *in vacuo* to afford a colourless solid, which was then transferred to the glovebox and washed with *n*-hexane (*ca*. 10 mL). The solid was washed again with toluene (*ca*. 5 mL) to remove a small impurity[i] to afford [{(DippNCMe)$_2$CH}Mg(μ-Me)]$_2$ as a colourless solid (3.97 g, 4.3 mmol, 72% yield). The product was used in the next step without further purification.

In a glovebox, [{(DippNCMe)$_2$CH}Mg(μ-Me)]$_2$ (500 mg, 0.55 mmol, 1 equiv.)[ii] was suspended in dry toluene (*ca*. 20 mL) in a J. Young ampoule and $PhSiH_3$ (0.202 mL, 1.64 mmol, 1.5 equiv.) was subsequently added. The ampoule was taken outside the glovebox and the mixture was stirred at 80 °C for 2 days. The white suspension becomes a colourless solution. The solution was cooled down, transferred to a Schlenk flask and concentrated *in vacuo* until precipitation started. The mixture was left in the freezer overnight, filtered *via* cannula (the discarded solid was a mixture of **[1]$_2$** and unreacted [{(DippNCMe)$_2$CH}Mg(μ-Me)]$_2$) and the solvent was evaporated. The residue was washed with *n*-hexane to afford the desired product **[1]$_2$** as an off-white solid (206 mg, 0.23 mmol, 43% yield).

^{1}H-NMR (400 MHz, C_6D_6) δ (ppm): 0.96 (d, $^3J_{H\text{-}H}$ = 6.3 Hz, 24H, CH*Me*$_2$), 1.10 (d, $^3J_{H\text{-}H}$ = 6.4 Hz, 24H, CH*Me*$_2$), 1.48 (s, 12H, *Me*), 3.05 (sept, $^3J_{H\text{-}H}$ = 7.2 Hz, 8H, C*H*Me$_2$), 4.03 (s, 2H, Mg-*H*), 4.83 (s, 2H, β-C*H*), 6.97 – 7.13 (m, 12H, *Ar*). Data are consistent with those previously reported.[9]

[i] It is important to use relatively fresh MeMgBr, as old solutions will increase the amount of impurities, hindering purification.
[ii] The reaction with phenylsilane can be successfully scaled up six-fold. A higher yield was obtained.

3. Synthesis of Heterometallic Hydrides

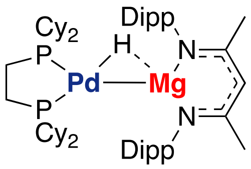

Synthesis of 2a: In a J. Young's NMR tube, **[1]$_2$** (10 mg, 0.011 mmol, 1.0 equiv.) and [Pd(μ-dcpe)]$_2$ (12 mg, 0.011 mmol, 1.0 equiv.) were dissolved in benzene (1 mL). The resulting solution was left at 25 °C for 4 days. The precipitate was filtered off through a glass fibre and the volatiles were removed *in vacuo*. The crude product was dissolved in a small amount of *n*-hexane (0.5 mL), and the solution was stored at –35 °C to afford crystals of **2a**. The desired product **2a** was isolated as yellow crystals (15 mg, 0.015 mmol, 68% yield).

^{1}H NMR (400 MHz, C_6D_6) δ (ppm): –0.55 (t, $^2J_{H–P}$ = 47 Hz, 1H, Pd*H*), 0.93–1.73 (series of overlapping m, *Cy*), 1.23 (d, $^3J_{H-H}$ = 7.1 Hz, 12H, CH*Me*$_2$), 1.35 (d, $^3J_{H-P}$ = 9.6 Hz, 4H, C*H*$_2$C*H*$_2$), 1.45 (d, $^3J_{H-H}$ = 6.5 Hz, 12H, CH*Me*$_2$), 1.76 (s, 6H, *Me*), 3.50 (sept, $^3J_{H-H}$ = 6.9 Hz, 4H, C*H*Me$_2$), 5.03 (s, 1H, β-C*H*), 7.03–7.13 (series of overlapping m, 6H, *Ar*).

***T_1* relaxation time** (Pd*H* signal, 298 K): 2.0 s.

^{13}C{^{1}H} NMR (126 MHz, C_6H_6) δ (ppm): 23.2–23.5 (m, 2xCH$_2$), 24.0 (2xCH$_3$), 24.4 (4xCH$_3$), 26.1 (4xCH$_3$), 26.7 (CH$_2$, *Cy*), 27.7 (d, J_{C-P} = 8 Hz, CH$_2$, *Cy*), 27.8 (d, J_{C-P} = 13 Hz, CH$_2$, *Cy*), 28.4 (4xCH), 29.0 (CH$_2$, *Cy*), 30.2 (d, J_{C-P} = 11 Hz, CH$_2$, *Cy*), 36.2 (br s, 4xCH, *Cy*), 95.0 (CH), 123.6 (4xCH), 125.0 (2xCH), 142.5 (4xC), 145.5 (2xC), 167.8 (2xC).

^{31}P{^{1}H} NMR (202 MHz, C_6H_6) δ (ppm): 50.4 (br s).

ATR IR (cm^{-1}): 3059, 2958, 2922, 2851, 1620, 1549, 1458, 1438, 1362, 1322, 1273, 1173, 1101, 758.

Due to the thermal instability of this compound CHN analysis was not obtained.

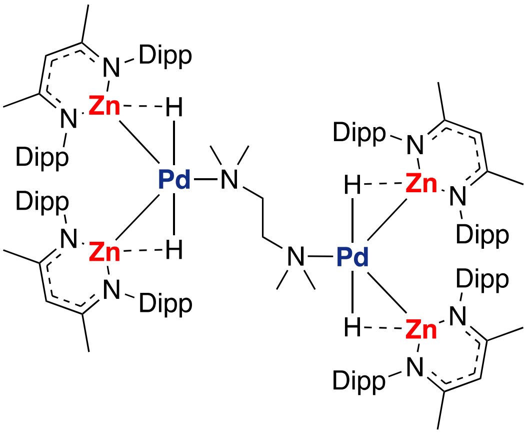

Synthesis of 4: In a J. Young's NMR tube, **3a** (40 mg, 0.083 mmol, 3.0 equiv.) was partially dissolved in benzene (0.6 mL). A solution of [PdMe$_2$(κ^2-TMEDA)] (7 mg, 0.028 mmol, 1.0 equiv.) in benzene (0.6 mL) was added, the tube sealed and inverted three times. The pale-yellow solution was allowed to stand at 25 °C for 25 minutes, during which time bubbles were observed and the colour changed to dark yellow/brown. The solvent and volatiles were removed *in vacuo* for 45 minutes, before the resultant solid was treated with *n*-pentane (1 mL), filtered through a glass fibre, and the solution was stored at –35 °C to afford crystals. The desired product **4** was isolated as yellow block crystals (14.4 mg, 0.0064 mmol, 46% yield).

^{1}H NMR (400 MHz, C_6D_6) δ (ppm): –4.53 (s, 4H, Pd*H*), 1.18 (d, $^3J_{H\text{-}H}$ = 6.9 Hz, 48H, CH*Me*$_2$), 1.20 (d, $^3J_{H\text{-}H}$ = 6.9 Hz, 48H, CH*Me*$_2$), 1.58 (s, 24H, *Me*), 2.12 (s, 12H, N*Me*$_2$), 2.36 (s, 4H, C*H*$_2$C*H*$_2$), 3.17 (sept, $^3J_{H\text{-}H}$ = 6.9 Hz, 16H, C*H*Me$_2$), 4.85 (s, 4H, β-C*H*), 7.12–7.20 (series of overlapping m, 6H, *Ar*).

***T_1* relaxation time** (Pd*H* signal, 298 K): 1.3 s

DOSY: TMEDA resonances diffuse (D_{ave} = 1.70 x 10^{-9} m^2/s) separately to β-diketiminate unit (D_{ave} = 7.05 x 10^{-10} m^2/s).

Variable temperature experiments. ^{1}H NMR spectra in toluene-*d*$_8$ were recorded between 298 and 198 K, with additional spectra measured in 5 K steps between 223 and 198 K. A broadening and eventual splitting of the Pd*H* signal was observed, with a coalescence temperature of 213 K. The non-uniform splitting of the signal (1.00:1.37 ratio of signals at –5.53 ppm and –4.94 ppm, respectively, at 198 K) suggests this is due to a slowing of an equilibrium between a TMEDA-bound and -unbound species, rather than freezing out of inequivalent hydride ligands. These data were modelled using the DNMR package within Topspin. The peak positions, intensities and molecular coefficients were freely iterated during the model, while line broadening was held at 10 Hz. A rate constant was extracted from each spectrum between 223 and 198 K, and Eyring analysis suggests an activation free energy of 8.6 kcal mol^{-1} for this process.

$^{13}C\{^1H\}$ NMR (101 MHz, C_6D_6) δ (ppm): 24.3 (s, *Me*), 25.5 (s, CH*Me*$_2$), 28.4 (s, *C*HMe$_2$), 46.1 (s, N*Me*$_2$), 58.5 (s, *C*H$_2$*C*H$_2$), 96.6 (s, β-*C*H), 123.9 (s, *meta/para*-Ar), 125.8 (s, *meta/para*-Ar), 141.8 (s, *ipso*-Ar), 142.8 (s, *ortho*-Ar), 167.2 (s, *C*=N).

ATR IR (cm^{-1}): 2963, 2922, 2867, 2821, 2775, 1933 (Pd–H), 1918 (Pd–H), 1869 (Pd–H), 1859 (Pd–H), 1522, 1435, 1383, 1314, 1266, 1252, 1177, 1100, 1020, 936, 854, 796, 760.

Anal. Calc. ($C_{122}H_{184}N_{10}Pd_2Zn_4$): C, 64.69; H, 8.19; N, 6.18. Found: C, 65.70; H, 8.49; N, 5.94.

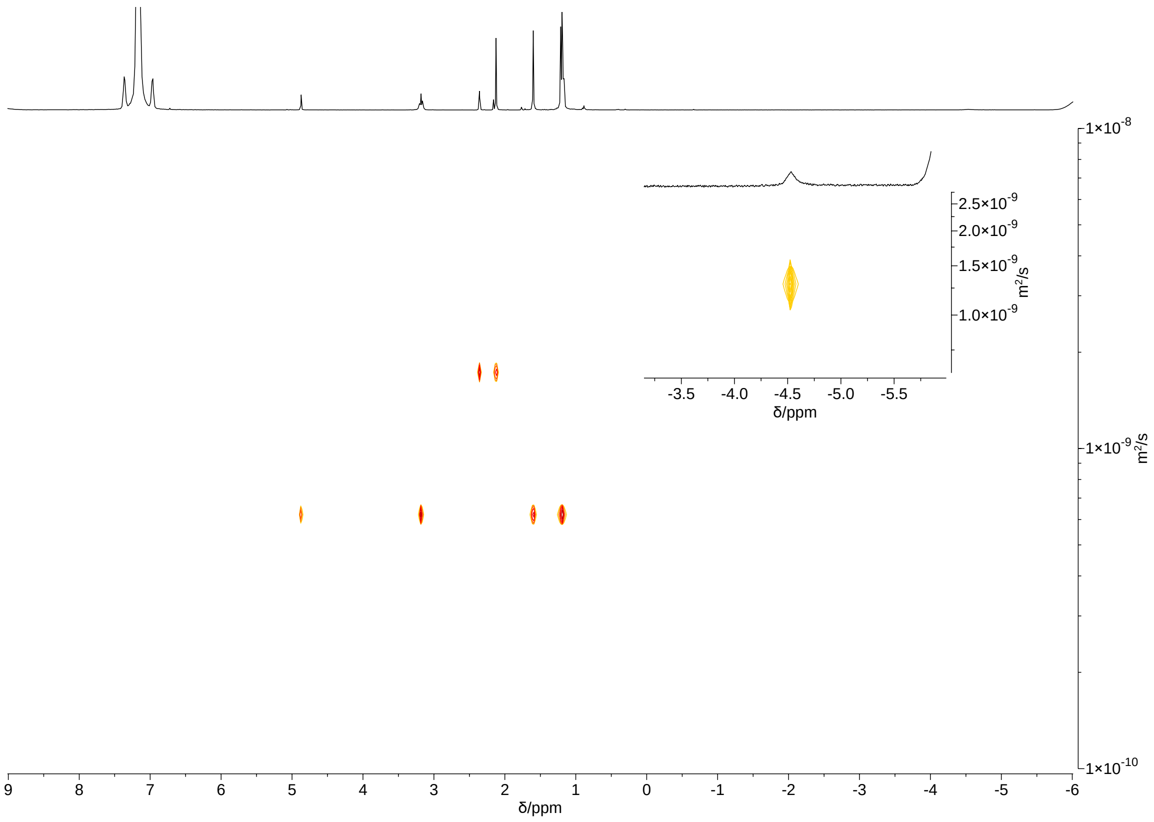

Figure S1. ^{1}H DOSY NMR spectrum of **4** showing different diffusion coefficients for TMEDA and ligand fragments.

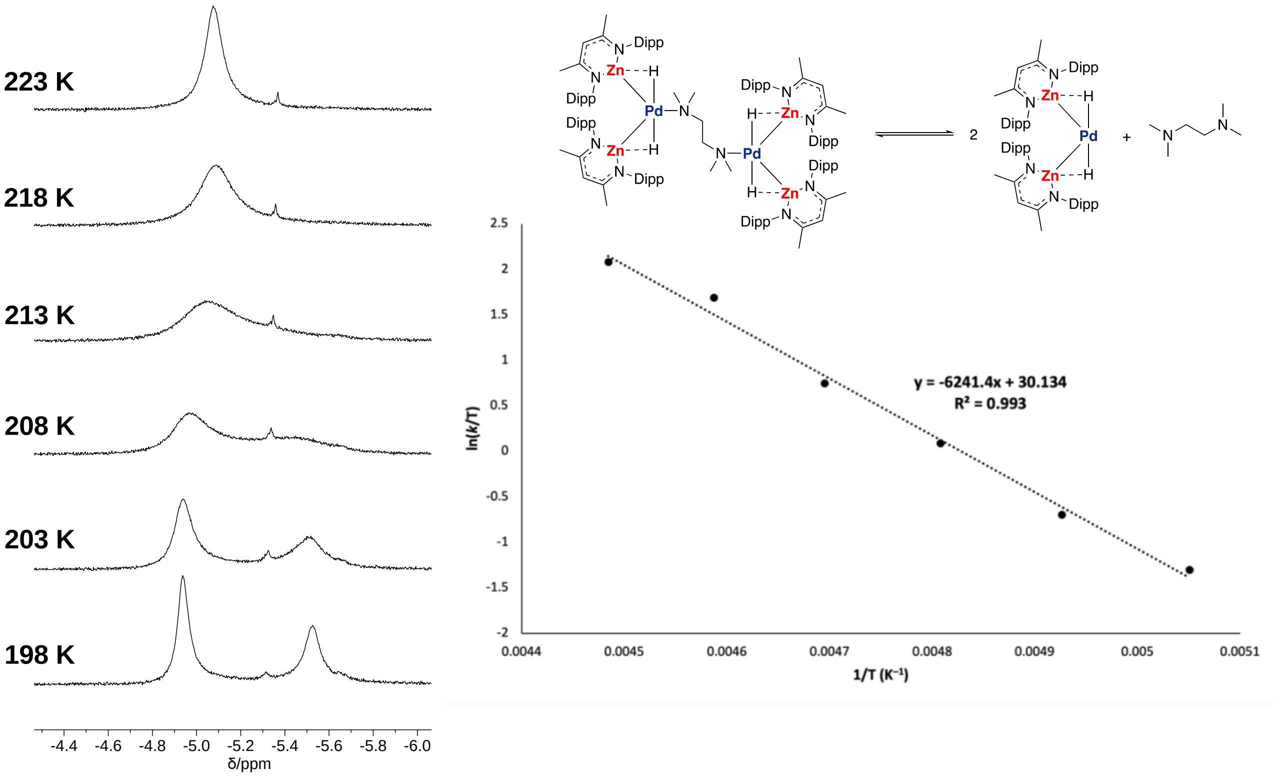

Figure S2. Experimental variable temperature ^{1}H NMR spectra showing the hydride region of **4** (left) and Eyring analysis of modelled data, including a schematic of the equilibrium being studied (right).

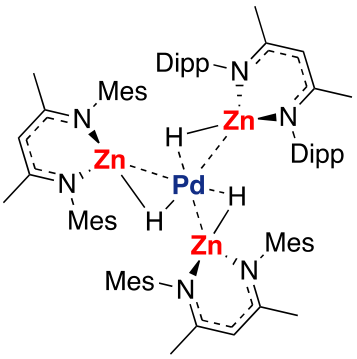

Synthesis of 5: Can be synthesised from isolated crystals of **4** (*Method A*), or in a one-pot reaction starting from **3a**, [$PdMe_2(\kappa^2$-TMEDA)] and **3b** (*Method B*).

Method A. In a J. Young's NMR tube, isolated crystals of **4** (3.4 mg, 0.0015 mmol, 1.0 equiv.) were dissolved in benzene (0.5 mL). A suspension of **3b** (2.4 mg, 0.0060 mmol, 4.0 equiv.) in benzene (0.5 mL) was added, and the tube sealed and inverted three times. The mixture was allowed to stand at 25 °C for 1 h, during which time the initial pale-yellow solution turns colourless. The solvent and volatiles were removed *in vacuo* for 1 h, before the resultant solid was treated with *n*-pentane (1 mL), filtered through a glass fibre, and the solution was stored at –35 °C to afford crystals. The crystals were discarded[iii] and the supernatant re-stored at –35 °C to afford a second batch of crystals, which are again discarded.[iii] The supernatant was evaporated, and the resultant powder washed swiftly with cold (–35 °C) *n*-pentane and dried *in vacuo*. The remaining powder contains an enriched mixture of **5** with some [{$(DippNCMe)_2CH$}ZnMe] (Dipp = 2,6-di-iso-propylphenyl) carried through from synthesis of **4** and **3a** still present.

Method B. In a J. Young's NMR tube, **3a** (20.2 mg, 0.042 mmol, 3.5 equiv.) was dissolved in benzene (0.5 mL). A solution of [$PdMe_2(\kappa^2$-TMEDA)] (3 mg, 0.012 mmol, 1.0 equiv.) in benzene (0.2 mL) was added, the tube sealed and inverted three times. The pale-yellow solution was allowed to stand at 25 °C for 20 minutes, during which time bubbles were observed. A suspension of **3b** (9.5 mg, 0.024 mmol, 2.0 equiv.) in benzene (0.3 mL) was added, and the tube sealed and inverted three times. The mixture was allowed to stand at 25 °C for 1 h, during which time the initial pale-yellow solution turns colourless. The subsequent work-up is identical to *Method A*. The resultant colourless powder (3.7 mg) was found to primarily consist of a mixture of [{$(DippNCMe)_2CH$}ZnMe] and **5** in a 60:40 ratio (Figures S3). NMR yield of **5** can be calculated as >0.0017 mmol, >14%.[iv]

[iii] Note a very small amount of product **5** does crystallise, and can be separated from crystals of [{$(DippNCMe)_2CH$}ZnMe] (major species), **3a** and **3b** manually under a microscope, differentiated by their slightly different morphologies: trapezoidal colourless blocks of **5** vs. more cubic colourless blocks of the other species present.
[iv] Note this is a minor product of the reaction (hence the convoluted purification and low yield).

^{1}H NMR (400 MHz, C_6H_6 w/ C_6D_6 lock tube) δ (ppm) : –1.26 ppm (s, PdH_3), 0.79 (d, $^3J_{HH}$ = 6.8 Hz, CHMe_2), 1.21 (d, $^3J_{HH}$ = 6.8 Hz, CHMe_2), 1.51 (s, Me^{Mes}), 1.66 (s, Me^{Dipp}), 1.93 (s, *ortho*-Me^{Mes}), 2.30 (s, *para*-Me^{Mes}), 3.14 (hept, $^3J_{HH}$ = 6.8 Hz, CHMe$_2$), 4.82 (s, β-CH^{Mes}), 4.92 (s, β-CH^{Dipp}), 6.76 (s, *meta*-CH^{Mes}), other aromatic peaks overlapping with C_6H_6 solvent.

DOSY: Diffusion coefficient for **5** (D_{ave} = 5.39 x 10^{-10} m^2/s) is smaller than corresponding diffusion coefficients for **3a** (D_{ave} = 8.20 x 10^{-10} m^2/s) and **4** (D_{ave} = 7.05 x 10^{-10} m^2/s) in the same solvent at similar concentrations. These data imply hydrodynamic radii of 6.34 Å, 4.17 Å and 5.15 Å, respectively, but with a large degree of error associated with the assumptions made during analysis with the Stokes-Einstein equation.

^{13}C{^{1}H} NMR (101 MHz, C_6H_6 w/ C_6D_6 lock tube) δ (ppm): 19.26 (s, $CH_3^{meta\text{-}Mes}$), 21.08 (s, $CH_3^{para\text{-}Mes}$), 23.12 (s, $CH_3^{BDI,Mes}$), 24.60 (s, $CH_3^{BDI,Dipp}$), 25.59 (s, $CH_3^{iPr,Dipp}$), 28.34 (s, $CH^{iPr,Dipp}$), 34.44 ($CH_3^{iPr,Dipp}$), 96.45 ($CH^{BDI,Mes}$), 96.69 ($CH^{BDI,Dipp}$), 123.44 (s, $CH^{meta\text{-}Dipp}$), 129.42 (s, $CH^{meta\text{-}Mes}$), 131.32 (s, $C^{ipso\text{-}Dipp}$), 131.90 (s, $C^{ipso\text{-}Mes}$), 142.58 (s, $C^{ortho\text{-}Dipp}$), 145.42 (s, $C^{para\text{-}Mes}$), 145.84 (s, $C^{ortho\text{-}Mes}$), 166.24 (s, C=$N^{BDI,Mes}$), 167.11 (s, C=$N^{BDI,Dipp}$).

ATR IR (cm^{-1}): 2960, 2922, 2863, 2821, 2775, 1804 (Pd–H), 1521, 1439, 1387, 1319, 1260, 1200, 1178, 1148, 1103, 1021, 932, 857, 798, 753.

Due to the thermal instability of this compound CHN analysis was not obtained.

Product **5** is not stable in solution at room temperature, and has completely decomposed after several hours. Over time the signals for [{(DippNCMe)$_2$CH}ZnMe] become more intense compared to **5**. The presence of a single hydride resonance observed for **5** (*vide infra*) suggests the species is fluxional on the NMR time-scale and hence each resonance associated with **5** will be timed-average. In solution, **5** is likely to exist as a mixture of complexes ranging from those with three {(DippNCMe)$_2$CH}Zn to three {(MesNCMe)$_2$CH}Zn ligands, as well as mixtures thereof. Computational analysis at the BS3 level (*vide infra*) reveals that moving from zero to three {(MesNCMe)$_2$CH}Zn ligands, while displacing {(DippNCMe)$_2$CH}Zn ligands, is exergonic by –3.7 (1 displaced), –15.8 (2 displaced) and –23.5 kcal mol^{-1} (3 displaced). The analogous species to **5** with three {(MesNCMe)$_2$CH}Zn ligands is computed to be the lowest energy. The crystal analysed by X-ray diffraction was found to contain one {(DippNCMe)$_2$CH}Zn and two {(MesNCMe)$_2$CH}Zn ligands. As such, and in addition to the low stability of the complex, it was not possible to assign accurate integration data to the observed resonances. However, resonances associated with both {(DippNCMe)$_2$CH}Zn and {(MesNCMe)$_2$CH}Zn ligands were observed that are not associated with [{(DippNCMe)$_2$CH}ZnMe].

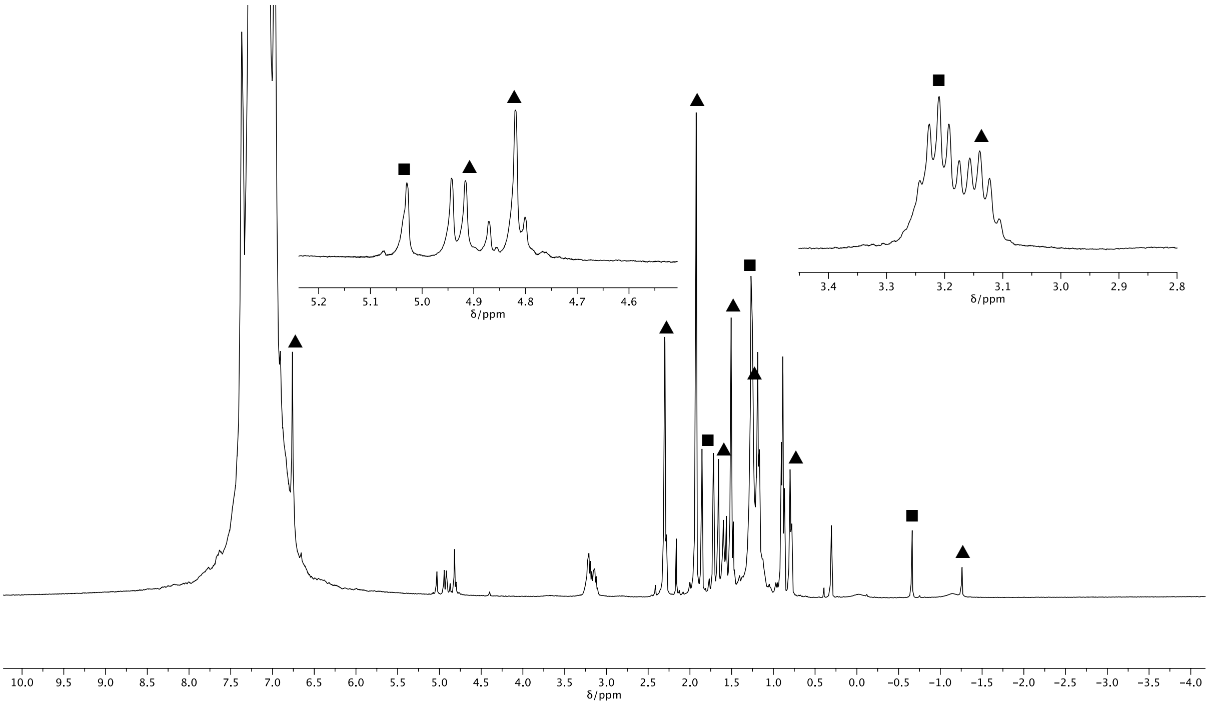

Figure S3. ^{1}H NMR spectrum of a 40:60 mixture of **5** and [{(DippNCMe)$_2$CH}ZnMe]. Identification of the resonances for **5** (▲) was aided by excluding those of [{(DippNCMe)$_2$CH}ZnMe] (■) which can be obtained separately as analytically pure crystals, and from 2D correlation experiments.

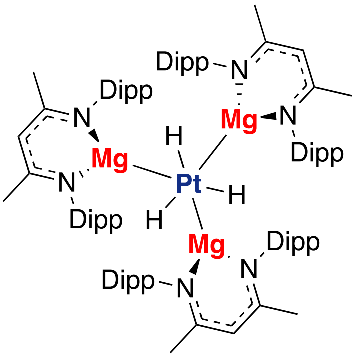

Synthesis of 6b: In an ampoule, [**1**]$_2$ (207.6 mg, 0.234 mmol, 2.0 equiv.) and [PtMe$_2$(κ^2-TMEDA)] (40 mg, 0.117 mmol, 1.0 equiv.) were dissolved in benzene (5 mL). The resulting solution was stirred at 25 °C for 4 days. The ampoule was then taken into the glovebox and the precipitate allowed to settle as a sediment. The supernatant was decanted, filtered through a glass fibre and the volatiles removed *in vacuo*. The residue was washed with cold *n*-hexane (1 mL). The residue was then extracted with cold toluene (1 mL), filtered through a glass fibre and the volatiles evaporated. The residue was then dissolved in a 1:1 *n*-hexane:toluene mixture (0.5 mL) and left at –35 °C. A small amount of solids precipitated out, which was discarded. The filtrate was then evaporated and washed swiftly with *n*-hexane (2 x 1 mL, at 25 °C). The remaining solid was dried *in vacuo* and shown to be the desired product. **6b** was isolated as a white solid (22 mg, 0.014 mmol, 12% yield).[v]

^{1}H NMR (400 MHz, C$_6$D$_6$) δ (ppm): –2.88 (s, $^1J_{H\text{-}Pt}$ = 1080 Hz (satellites), 3H, PtH_3), 0.98 (d, $^3J_{H\text{-}H}$ = 6.8 Hz, 36H, CHMe_2), 1.20 (d, $^3J_{H\text{-}H}$ = 6.9 Hz, 36H, CHMe_2), 1.54 (s, 18H, *Me*), 3.14 (sept, $^3J_{H\text{-}H}$ = 6.8 Hz, 12H, C*H*Me$_2$), 4.87 (s, 3H, β-C*H*), 7.03–7.13 (m, 18H, *Ar*).

***T*₁ relaxation time** (PtH_3 signal, 298 K): 1.0 s.

^{13}C{^{1}H} NMR (101 MHz, C$_6$D$_6$) δ (ppm): 24.2 (12xCH$_3$), 24.7 (6xCH$_3$), 25.7 (12xCH$_3$), 28.5 (12xCH), 96.2 (3xCH), 123.9 (12xCH), 125.4 (6xCH), 142.5 (12xC), 145.9 (6xC), 169.2 (6xC).

^{195}Pt NMR (108 Hz, C$_6$H$_6$) δ (ppm): –6159.1 (q, $^1J_{H\text{-}Pt}$ = 1080 Hz).

ATR IR (cm^{-1}): 3060, 2960, 2926, 2866, 1705, 1621, 1551, 1461, 1439, 1405, 1364, 1320, 1260, 1174, 1103, 1018, 787, 757.

[v] Note this is a minor product of the reaction (hence the convoluted purification and low yield).

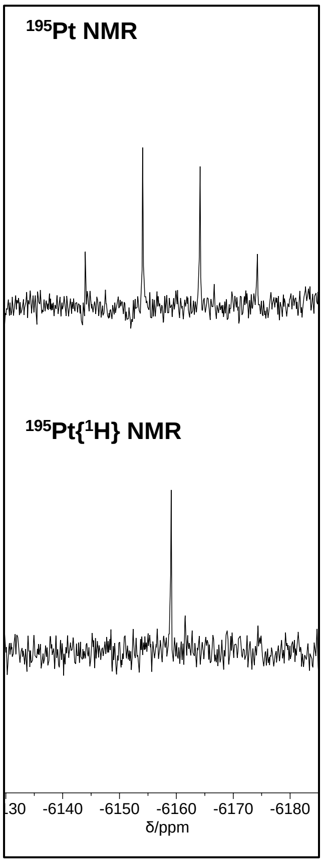

Figure S4. Stacked ^{195}Pt NMR spectra showing the ^{1}H coupled (top) and decoupled (bottom) spectra of **6b.**

4. Products from the Reaction of [PdMe$_2$(κ^2-TMEDA)] and **3a**

4.1. NMR spectroscopy

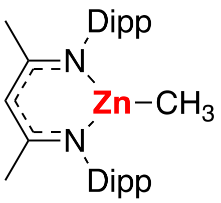

In situ ^{1}H NMR spectra recorded for the reaction between [PdMe$_2$(κ^2-TMEDA)] and **3a** show the formation of two species with a distinct high-field resonances: the Pd-containing species **4** (–4.53 ppm), and another species assigned as the product of a ligand exchange reaction between Pd and Zn (–0.66 ppm). The exchange of a Pd–Me and a Zn–H ligand result in the formation a stable [{(DippNCMe)$_2$CH}ZnMe] (Dipp = 2,6-di-iso-propylphenyl) complex. A labelling experiment was performed where ^{13}C-labelled [Pd(^{13}CH$_3$)$_2$(κ^2-TMEDA)][6,7] was reacted with **3a**. In a J. Young NMR tube, a solution of [Pd(^{13}CH$_3$)$_2$(κ^2-TMEDA)] (4 mg, 0.0157 mmol, 1 equiv.) in C_6H_6 (0.5 mL) was added to a solution of **3a** (22.8 mg, 0.0471 mmol, 3 equiv.) in C_6H_6 (0.5 mL). The resultant *in situ* ^{1}H NMR spectrum shows the formation of two species that incorporate the ^{13}C-label. These were assigned as [{(DippNCMe)$_2$CH}Zn(^{13}CH$_3$)] (–0.66 ppm, $^1J_{CH}$ = 121 Hz) and ^{13}CH$_4$ (0.16 ppm, $^1J_{CH}$ = 126 Hz), with both showing corresponding cross-peaks to intense ^{13}C resonances in a HSQC experiment. The reaction between [PdMe$_2$(κ^2-TMEDA)] and **3a** clearly results in both ligand exchange and reductive elimination processes prior to formation of **4**.

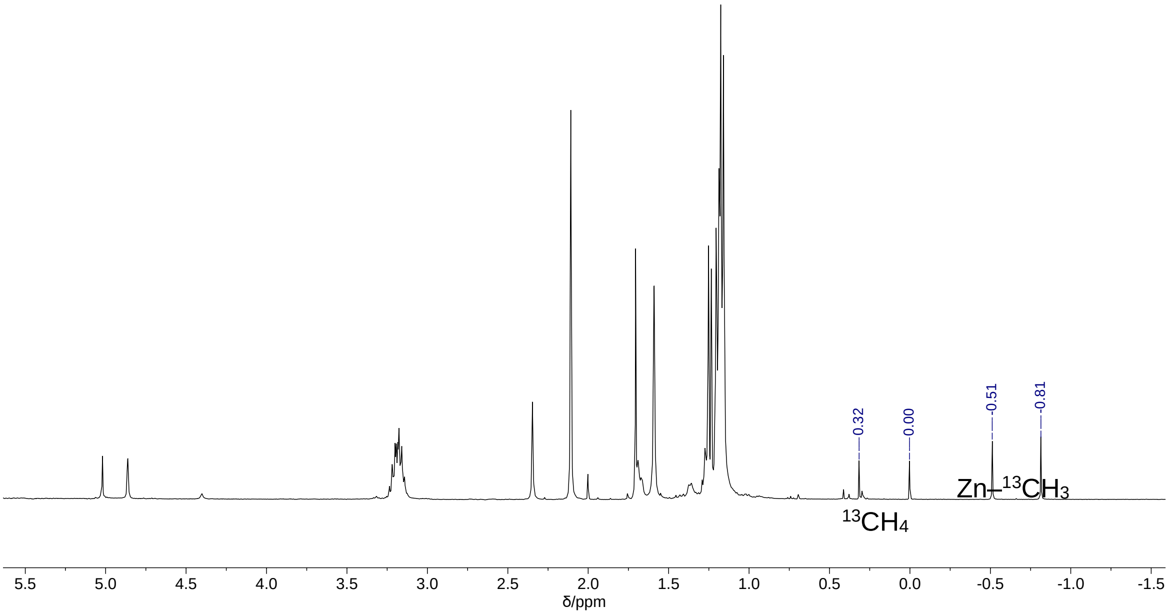

Figure S5. ^{1}H NMR spectrum of reaction mixture between [Pd(^{13}CH$_3$)$_2$(TMEDA)] and **3a.**

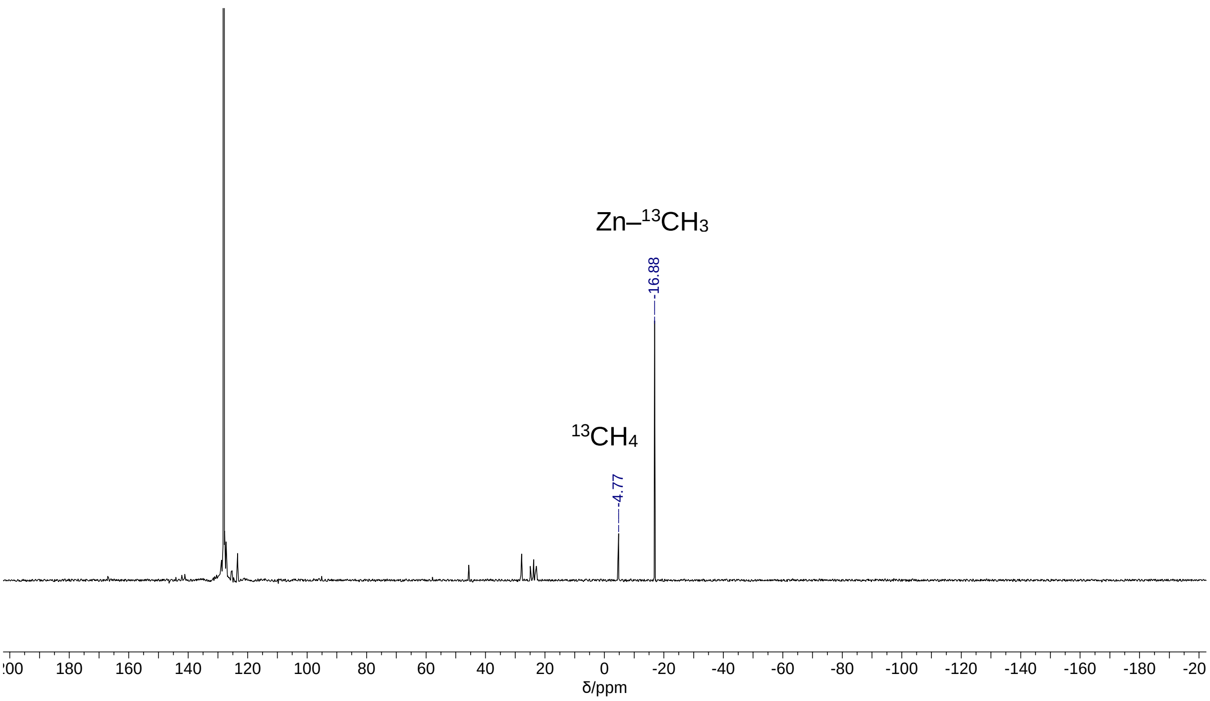

Figure S6. $^{13}C\{^{1}H\}$ NMR spectrum of reaction mixture between $[Pd(^{13}CH_3)_2(TMEDA)]$ and **3a.**

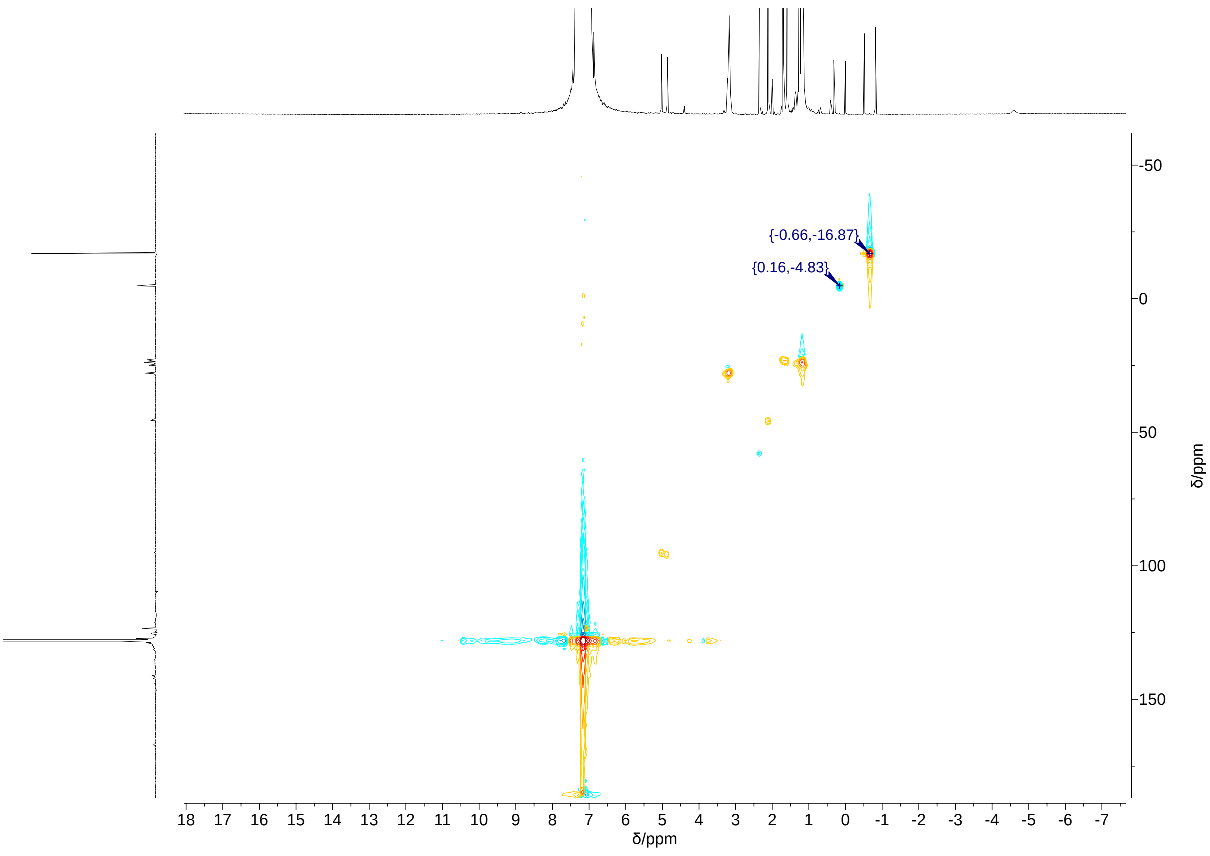

Figure S7. ^{1}H-^{13}C HSQC NMR spectrum of reaction mixture between $[Pd(^{13}CH_3)_2(\kappa^2\text{-TMEDA})]$ and **2.**

[{(DippNCMe)$_2$CH}ZnMe] has been previously reported via a metathesis reaction between [{(DippNCMe)$_2$CH}ZnI] and MeLi.[10] The analogous reaction between [PdMe$_2$(κ^2-TMEDA)] and [{(MesNCMe)$_2$CH}ZnH][3] (Mes = 2,4,6-trimethylphenyl) also resulted in Pd–Me and Zn–H ligand exchange, forming the corresponding [{(MesNCMe)$_2$CH}ZnMe] complex. In this case, the high-field Zn–Me resonance was observed at –0.61 ppm, and gave identical NMR data to those previously reported for this complex.[11]

Products formed from the reaction between [PdMe$_2$(κ^2-TMEDA)] and **3a** are also capable of undergoing solvent C–H activation at higher temperatures. Formation of [{(DippNCMe)$_2$CH}ZnPh][2,9] from activation of the benzene solvent is observed by ^{1}H NMR spectroscopy if the reaction mixture is heated to ≥50 °C.

4.2. X-ray Diffraction Study of Side-Product

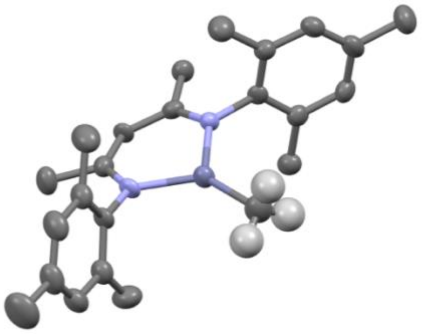

Two distinct products can be crystallized from the reaction between [PdMe$_2$(κ^2-TMEDA)] and **3a**. Larger pale yellow block crystals of **4** and smaller colourless crystals can be separated by hand under a microscope. X-ray diffraction experiments undertaken with the colourless crystals were resolved to show a dimeric structure with two [{(DippNCMe)$_2$CH}Zn] units, but further analysis was inconclusive due to unassignable electron density (max peak 8.3, R_1 = 18.55%, GooF = 1.581) between the two Zn centers. This structure was persistent across several crystals that were analysed, and appears to be a mixture of different species. The analogous [{(MesNCMe)$_2$CH}ZnMe] species was isolated from the reaction between [PdMe$_2$(κ^2-TMEDA)] and **3b**, and afforded crystals suitable for X-ray diffraction which confirmed the structure. The [{(MesNCMe)$_2$CH}ZnMe] complex has been previously reported and gives identical data to the crystals obtained here.[12]

5. Crystallographic Data – X-ray Diffraction

Table S1. Crystal data, data collection and refinement parameters for the structures of **2a**, **4**, **5** and **6b**

data	2a	4	5	6b
**formula**	$C_{55}H_{90}MgN_2P_2Pd$	$C_{122}H_{184}N_{10}Pd_2Z$	$C_{75}H_{102}N_6PdZn_3$	$C_{87}H_{126}Mg_3N_6Pt$
**solvent**	$0.5(C_6H_{14})$	C_6H_{14}	C_6H_{14}	$C_7H_8 \cdot C_6H_{14}$
**formula weight**	1015.02	2351.23	1476.30	1702.26
**colour, habit**	yellow platy needles	yellow blocks	colourless blocky needles	colourless needles
**temperature / K**	173	173	173	173
**crystal system**	triclinic	monoclinic	monoclinic	monoclinic
**space group**	*P*–1 (no. 2)	$P2_1/n$ (no. 14)	C2/*c* (no. 15)	$P2_1/c$ (no. 14)
***a* / Å**	11.9440(4)	14.6246(5)	25.5811(8)	16.2798(5)
***b* / Å**	12.0368(6)	16.6683(5)	13.9813(5)	13.0600(5)
***c* / Å**	22.8595(9)	26.2662(8)	45.002(3)	45.8501(10)
**α / deg**	91.871(4)	90	90	90
**β / deg**	104.635(3)	103.526(3)	102.049(4)	93.226(2)
**γ / deg**	112.172(4)	90	90	90
**V / Å^3**	2914.5(2)	6225.2(3)	15740.7(14)	9732.9(5)
Z	2	2	8	4
**D_c / g cm^{-3}**	1.157	1.254	1.246	1.162
**radiation used**	Cu-Kα	Cu-Kα	Cu-Kα	Cu-Kα
**μ / mm^{-1}**	3.444	1.091	3.175	3.220
**no. of unique reflns**				
**measured (R_{int})**	11034 (0.0388)	12392 (0.0330)	15159 (0.0337)	18623 (0.0424)
**obs, $\|F_o\| > 4\sigma(\|F_o\|)$**	9204	8972	13010	14088
**completeness (%)** [a]	98.0	99.1	98.8	98.7
**no. of variables**	613	680	873	951
**R_1(obs), wR_2(all)** [b]	0.0353, 0.0893	0.0378, 0.0790	0.0479, 0.1108	0.0376, 0.0897
**CCDC code**	2107421	2107423	2150654	2107426

[a] Completeness to 0.84 Å resolution. [b] $R_1 = \Sigma||F_o| - |F_c||/\Sigma|F_o|$; $wR_2 = \{\Sigma[w(F_o^2 - F_c^2)^2] / \Sigma[w(F_o^2)^2]\}^{1/2}$; $w^{-1} = \sigma^2(F_o^2) + (aP)^2 + bP$.

X-ray crystal structure of 2a. The C61-based included hexane solvent molecule in the structure of **2a** was found to be disordered across a centre of symmetry, and two unique orientations were identified of *ca*. 26 and 24% occupancy (with two further orientations of the same occupancies being generated by operation of the inversion centre). The geometries of the two unique orientations were optimised, the thermal parameters of adjacent atoms were restrained to be similar, and all the atoms of both unique orientations were refined isotropically. The Pd–H–Mg bridging hydrogen atom was located from a Δ*F* map and refined freely.

X-ray crystal structure of 4. The structure of **4** was found to sit across a centre of symmetry at the middle of the central C–C bond of the bridging N006-based TMEDA ligand. The C01-based included hexane solvent molecule was found to be disordered across a centre of symmetry, with one unique orientation identified. The geometries of the hexane solvent was optimised and refined anisotropically. The Pd–H–Zn bridging hydrogen atoms were located from a Δ*F* map and refined freely.

X-ray crystal structure of 5. The C21- and C33-based *iso*-propyl groups in the structure of **5** were both found to be disordered and in each case two orientations were identified, of *ca.* 66:34 and 57:43%, respectively. The geometries of each pair of orientations were optimised, the thermal parameters of adjacent atoms were restrained to be similar, and both pairs of orientations were refined anisotropically. The C86-based included solvent molecule was found to sit in one position and was refined anisotropically. Pd–H–Zn bridging hydrogen atoms were located from a Δ*F* map and refined freely.

X-ray crystal structure of 6b. The C12-, C27-, and C42-based *iso*-propyl groups in the structure of **6b** were all found to be disordered and in each case two orientations were identified, of *ca*. 51:49, 54:46 and 87:13% occupancy respectively. The geometries of each pair of orientations were optimised, the thermal parameters of adjacent atoms were restrained to be similar, and only the non-hydrogen atoms of the major occupancy orientations were refined anisotropically (those of the minor occupancy orientations were refined isotropically). The included solvent was found to be highly disordered, and the best approach to handling this diffuse electron density was found to be the SQUEEZE routine of PLATON.[13] This suggested a total of 417 electrons per unit cell, equivalent to 104.3 electrons per asymmetric unit. Before the use of SQUEEZE the solvent most resembled a 1:1 mixture of toluene (C_7H_8, 50 electrons) and hexane (C_6H_{14}, 50 electrons), and one toluene and one hexane molecule corresponds to 100 electrons, so this was used as the solvent present. As a result, the atom list for the asymmetric unit is low by C_7H_8 + C_6H_{14} = $C_{13}H_{22}$ (and that for the unit cell low by $C_{52}H_{88}$) compared to what is actually presumed to be present. The three unique Pt–H–Mg bridging hydrogen atoms were all located from Δ*F* maps and refined freely.

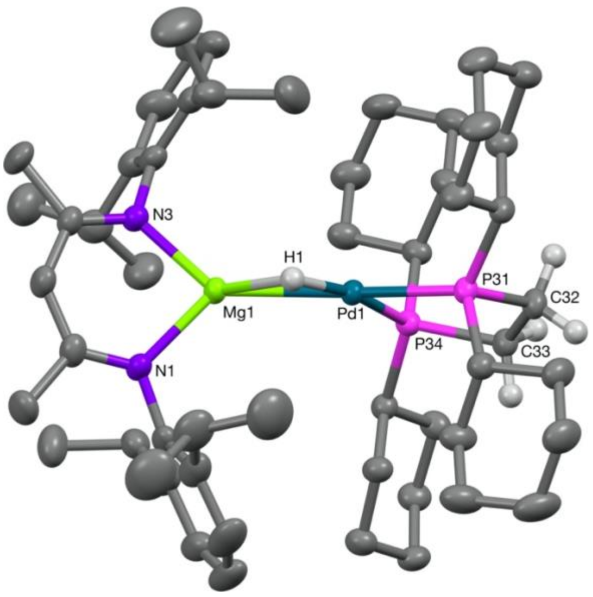

Figure S8. Structure of **2a**. Solvent and selected hydrogens removed for clarity. Thermal ellipsoids drawn at 50% probability.

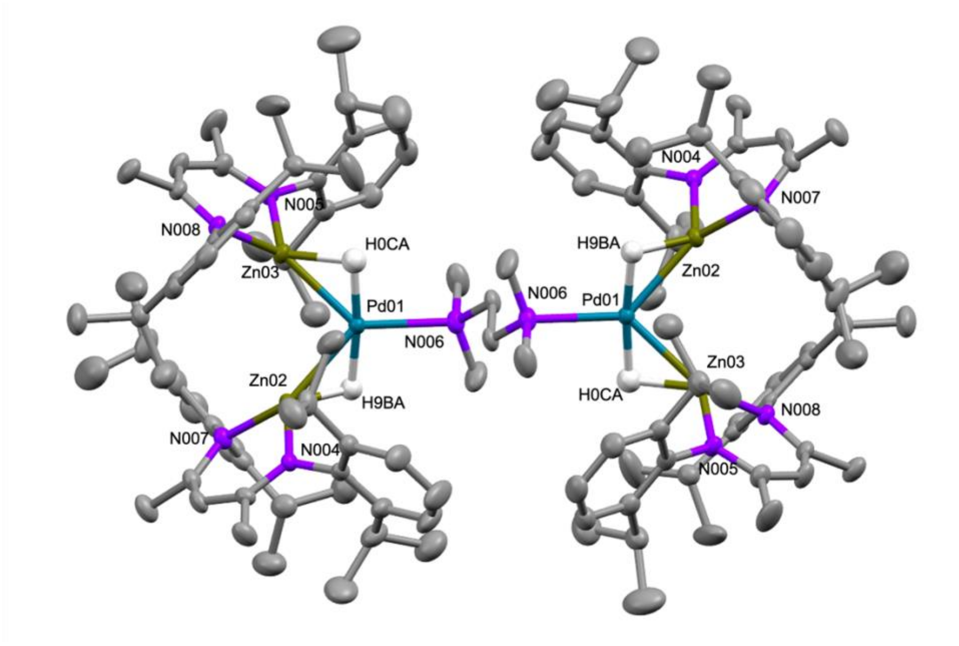

Figure S9. Structure of **4**. Solvent and selected hydrogens removed for clarity. Thermal ellipsoids drawn at 50% probability.

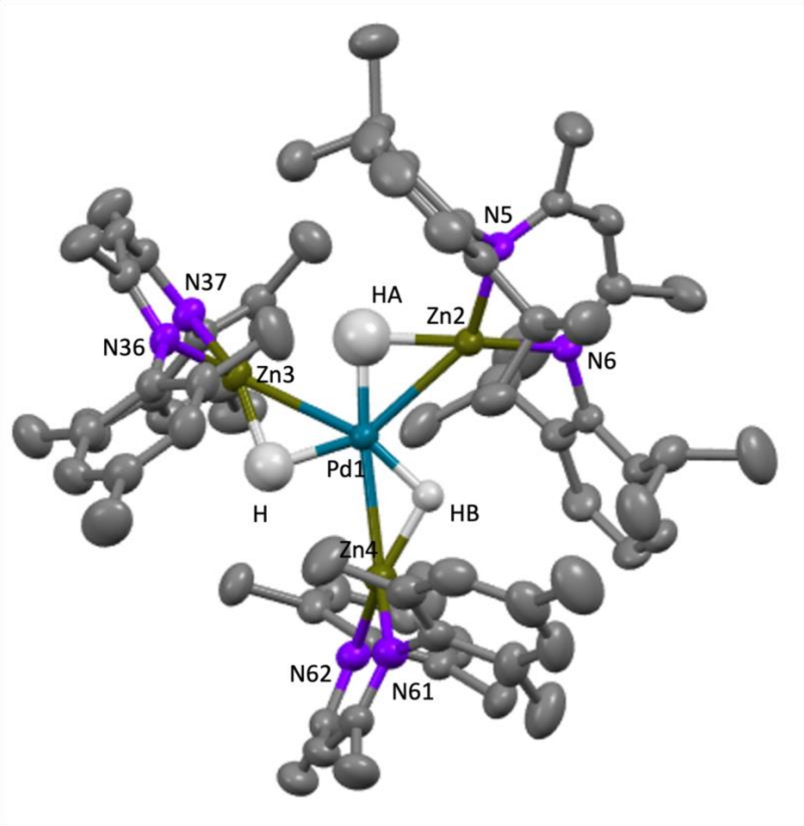

Figure S10. Structure of **5**. Solvent and selected hydrogens removed for clarity. Thermal ellipsoids drawn at 50% probability.

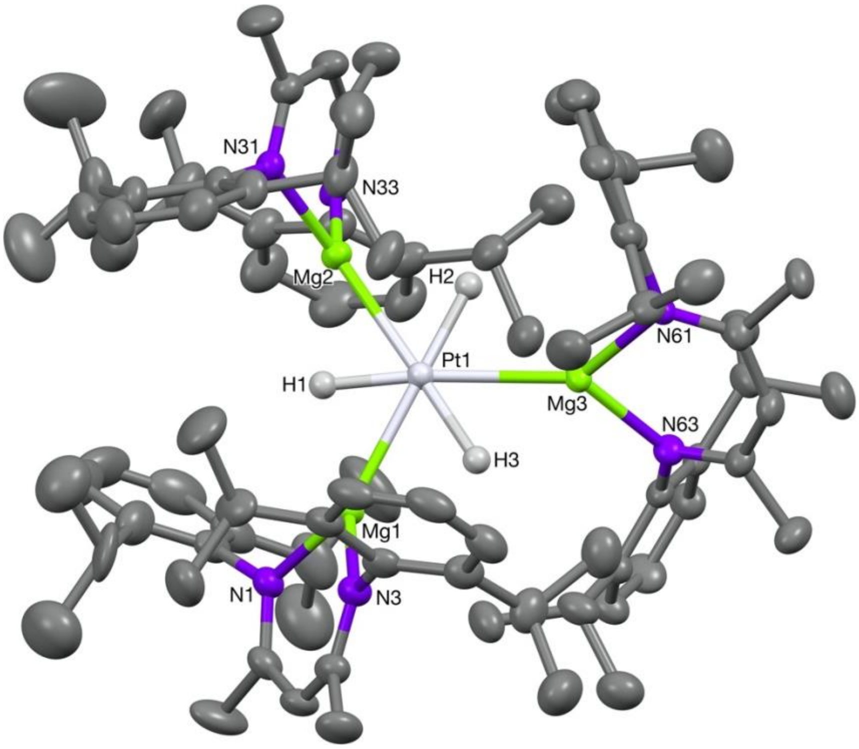

Figure S11. Structure of **6b**. Solvent and selected hydrogens removed for clarity. Thermal ellipsoids drawn at 50% probability.

6. Neutron single-crystal Laue Diffraction studies

Crystals of **2a** arrived to ANSTO *via* air courier and data suitable for structural analysis were collected.

The crystal of **2a** (yellow needle 0.2 x 0.4 x 1.4 mm^3) selected for data collection was the largest available quality specimen and ultimately proved to be a new polymorph of the compound, thus, the unit cell and crystal symmetry were not known at the time of the experiment. 21 x 10000s exposure Laue neutron diffraction images (the crystal was close to the minimum viable size for a unit cell of this volume) with an interframe rotation of 17° about φ were recorded from a single setting of the sample with respect to the φ axis. It has not been possible to acquire the additional data from a second setting of the crystal which would be preferable due to the actual low symmetry unit cell as additional beamtime has not been available.

Data reduction by means of the LaueG[14] suite incorporating ArgonneBoxes[15] to the resolution of the observed pattern (1.1 Å) with all uniquely indexable reciprocal lattice points for all wavelengths $0.85 \leq \lambda < 1.7$Å included in the integration and normalization. The overall R_{int} for all data extracted in this manner is, as is typical for such experiments, meaningless due to the large number of very weak data and the inherently high background in the neutron Laue experiment whereas R_{int} for the 4σ data [7.7(6.6) 18460 reflections 2832 $I > 4\sigma I$] demonstrates that the merging of data is valid. Structure refinement[16] commenced from the X-ray model coordinates and after scale factor refinement only, a difference map phased on the non-hydrogen atom positions revealed all the hydrogen atom sites for the molecule of interest. A full-matrix least-squares refinement on F of all atomic sites modelled with anisotropic displacement parameters for non-hydrogen atoms and the metal bonded hydrides, with remaining hydrogens isotropic and using a Chebychev polynomial weighting scheme (3 term) converged to: R = 10.9%, R_w = 13.7% and S = 0.97 for 1400 parameters, 1364 restraints and 1534 observations $I \geq 3\sigma I$. At convergence, the difference density maps were featureless at +/- ~0.9fm $Å^3$ for **2a** well below the value corresponding to any atom of this structure.

7. Discussion of Ternary Hydrides

Several ionic ternary hydrides have been characterised by neutron diffraction including [Na_2PdH_2], [$NaBaPdD_3$] and [K_2PdH_4].[17,18] Upon first inspection, these species bear some resemblance to the complexes reported herein and hence a critical comparison might be informative. These species possess linear, trigonal planar and square planar geometries at the transition metal, respectively (Figure S12). For example, the closest point of comparison for the hexagonal planar species **6a** or **6b** is [$NaBaPdD_3$]. [$NaBaPdD_3$] contains a trigonal planar Pd centre with a Pd–D bond distance of 1.719(8) Å.[19] The Pd---Na distance of 3.810(1) Å is well beyond the covalent radii (Pauling, 2.85 Å; Pyykkö, 2.75 Å). The Na atoms sit outside the trigonal plane and interact exclusively through bridging hydride interactions. The barium atoms also sit outside the trigonal plane being located in an axial position. The Pd–Ba distance of 3.041(1) Å is long but in a reasonable range for the covalent radii (Pauling, 3.26 Å; Pyykkö, 3.16 Å). While it is difficult to rule out a direct metal---metal interaction in this species it is clearly not one that would result in a hexagonal planar geometry. Similarly, although [Na_2PdH_2] contains a linear H–Pd–H motif related to that found in **4** and [K_2PdH_4] contains a square planar [PdH_4]$^{2-}$ unit, in both cases the s-block counterions do not approach the transition metal[20,21] and are located at remote sites in crystal lattice. For comparison the Pd–H bond lengths in [Na_2PdH_2] and [K_2PdH_4] are 1.68 and 1.625(8) Å, respectively.[16] The bonding in these ternary hydrides is likely dominated by an ionic interaction between the {PdH_n}$^{n-}$ and M^+ or M^{2+} fragments with limited or no interaction between the metals themselves. As such it is reasonable to conclude that the structures of **2**, **5** and **6** are not identical to those known from ionic metal salts as both the geometries and short metal---metal distances are inconsistent with those established for ternary hydrides.

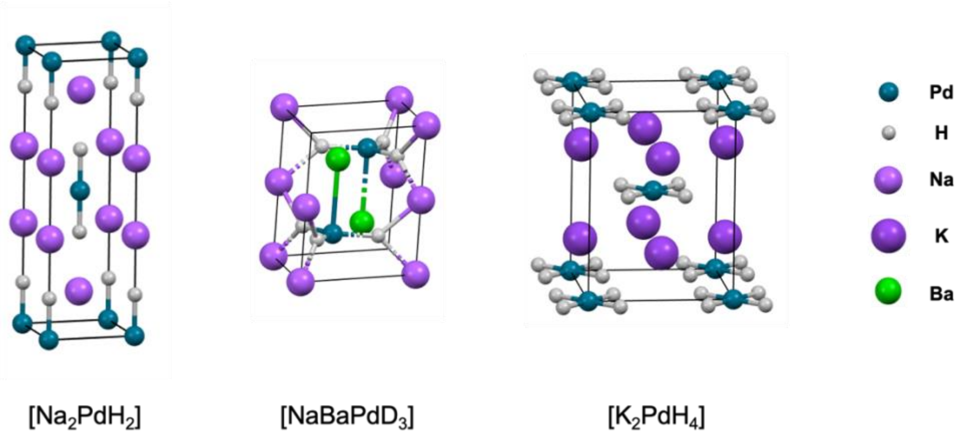

Figure S12. Reported neutron diffraction structures of unit cells containing linear, trigonal and square planar palladium hydride arrangements.

8. Density Functional Theory Calculations

8.1. Computational Methods

DFT calculations were run using Gaussian 09 (Revision D.01)[22] and Gaussian 16[23] using the ωB97X hybrid exchange-correlation functional.[24] NBO analysis was performed using NBO 6.0.[25] QTAIM analysis was conducted using the AIMAll package.[26] Data are presented from *veryfine mesh* calculations but there are no differences in the appearance of bcps when calculations were run with a *superfine* or *ultrafine mesh*. Standard cut-offs for plotting the data with AIMAll were used. Non-covalent interactions were analysed using the NCIPLOT 3.0 program.[27] Geometry optimisations were performed without symmetry constraints, unless otherwise specified, and the nature of the stationary points was confirmed as minima by frequency calculations (no imaginary frequencies). The default numerical integration grid was improved using a pruned grid with 99 radial shells and 590 angular points per shell (int=ultrafine). It should be noted that for the geometry and bonding analysis performed in this work, the use of dispersion or solvent corrections was deemed unnecessary.

The level of theory used has previously been benchmarked in our group and shown to reproduce accurately the experimental results.[2,4] Different basis sets (BS1-4) were used as detailed. Geometry optimisations and population analyses on the complexes were performed mainly using BS1. Geometry optimisation of complex **6b** with BS1 did not accurately reproduce the experimentally observed Mg–H distances, likely due to the flat nature of the PES. Therefore, the optimisation and population analysis of **6b** was performed using a slightly modified basis set, BS2. Optimisation and bonding analysis of the model systems were performed using the larger basis set, BS3, which includes quadruple-ξ functions for Mg and Zn. Finally, wavefunction generation for QTAIM inputs were generated using BS4. Initially single-point calculations for population analyses for all full structures were performed with this basis set as well, but the systems were found to be too big. Bonding analysis was carried out for **1** and **3a** using BS1, BS3 and BS4, and for **2a** and **2b** using BS1 and BS4, with no significant change (±0.03) in values observed (see Table S2). Bonding analysis using BS1 is discussed in the paper.

BS1 was built as follows.[28] The SDD effective core potential was used for all metals (SDDAll). The split-valence 6-31G(d) basis set was used for C and H atoms. The basis set for metal hydrides was expanded by adding one extra set of diffuse functions and three sets of p- and one set of d- polarisation functions, *i.e.* formally [6-31++G(d,3pd)]. The triple-ξ 6-311+G* basis set was used for heteroatoms.

BS2 was built as follows. The SDD effective core potential was used for all metals (SDDAll). The split-valence 6-31G(d,p) basis set was used for C and H atoms, and the triple-ξ 6-311+G* basis set was used for heteroatoms. The metal hydrides used the same expanded basis set as BS1, formally [6-31++G(d,3pd)].

BS3 was built as follows. Pd and Pt were described with the SDD effective core potential, while the other atoms (C, H, N and P) including metals (Mg or Zn) were described using Ahlrichs quadruple-ξ basis set def2-QZVPP.[29]

BS4 was built as follows. Pd and Pt were described with the SDD effective core potential, while the other atoms (C, H, N and P) including metals (Mg or Zn) were described using Ahlrichs triplet-ξ basis set def2-TZVPP.[29]

8.2. Comparison of Bonding Parameters for Model and Full Systems

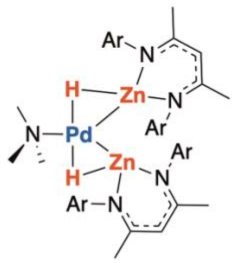

4'

Ar = 2,6-di-iso-propylphenyl

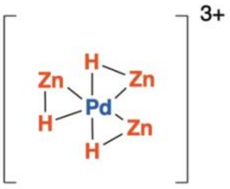

5'

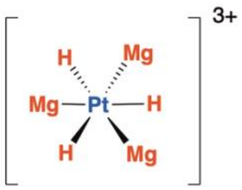

6b'

Table S2. Comparison of NPA charges and Wiberg Bond Indices for **1**, **2a**, **2b**, **3a** and **6b** across different basis sets (M^1 = Mg or Zn, M^2 = Pd or Pt)

		1			3a			2a		2b		6b	
		BS1	***BS3***	***BS4***	***BS1***	***BS3***	***BS4***	***BS1***	***BS4***	***BS1***	***BS4***	***BS1***	***BS2***
NPA charge	*M^1*	1.61	1.59	1.60	1.38	1.40	1.40	1.54	1.51	1.26	1.28	1.61–1.67	1.61–1.67
	M^2	-	-	-	-	-	-	–0.23	–0.24	–0.16	–0.17	–0.56	–0.56
	H	–0.72	–0.71	–0.71	–0.54	–0.56	–0.56	–0.51	–0.50	–0.41	–0.41	–0.52 - –0.58	–0.52 - –0.58
WBI	*M^1–M^2*	-	-	-	-	-	-	0.23	0.24	0.31	0.30	0.11-0.17	0.11-0.17
	M^1–H	0.43	0.45	0.45	0.61	0.59	0.59	0.21	0.23	0.31	0.29	0.10-0.11	0.09-0.11
	M^2–H	-	-	-	-	-	-	0.39	0.37	0.37	0.36	0.30-0.37	0.30-0.37

M^1 = Mg (**1**, **2a**, **6b**), Zn (**3a**, **2b**); M^2 = Pd (**2a**, **2b**), Pt (**6b**)

Table S3. Comparison of selected bond lengths (Å) between computed model (**4’**, **5’** and **6b’** using BS3) and full (**4**, **5** and **6b** using BS1 or BS2) systems.

	**4’**	**4**	**5’**	**5**	**6b’**	**6b**
M^1–H	1.75	1.73, 1.74	1.75	1.77-1.81	2.24-2.26	2.18-2.43
M^2–H	1.68	1.68, 1.69	1.70	1.69, 1.71	1.68	1.69, 1.71
M^1–M^2	2.46	2.45-2.48	2.54	2.48, 2.49	2.56	2.59, 2.61

M^1 = Zn (**4’**, **4**, **5’** and **5**), Mg (**6b’** and **6b**); M^2 = Pd (**4’**, **4**, **5’** and **5**), Pt (**6b’** and **6b**)

Table S4. Comparison of NPA charges and Wiberg Bond Indices between computed model (**4’** using BS1; **5’** and **6b’** using BS3) and full (**4** and **5** using BS1 and **6b** using BS2) systems.

	**4’**	**4**	**5’**	**5**	**6b’**	**6b**
M^1	1.34	1.34-1.35	1.39	1.39-1.43	1.66	1.61-1.67
M^2	–0.15	–0.15	–0.10	–0.26	–0.55	–0.56
H	–0.43 - –0.44	–0.43 - –0.44	–0.36	–0.46 - –0.50	–0.48	–0.52 - –0.58
M^1–M^2	0.26	0.25-0.26	0.24	0.14-0.18	0.45	0.11-0.17
M^1–H	0.33-0.34	0.33-0.35	0.45	0.30-0.32	0.34	0.09-0.11
M^2–H	0.32-0.33	0.32-0.33	0.29	0.25-0.28	0.63, 0.64	0.30-0.37

M^1 = Zn (**4’**, **4**, **5’** and **5**), Mg (**6b’** and **6b**); M^2 = Pd (**4’**, **4**, **5’** and **5**), Pt (**6b’** and **6b**)

8.3. Molecular Orbital Analysis

*Analysis of **2a' (model)***

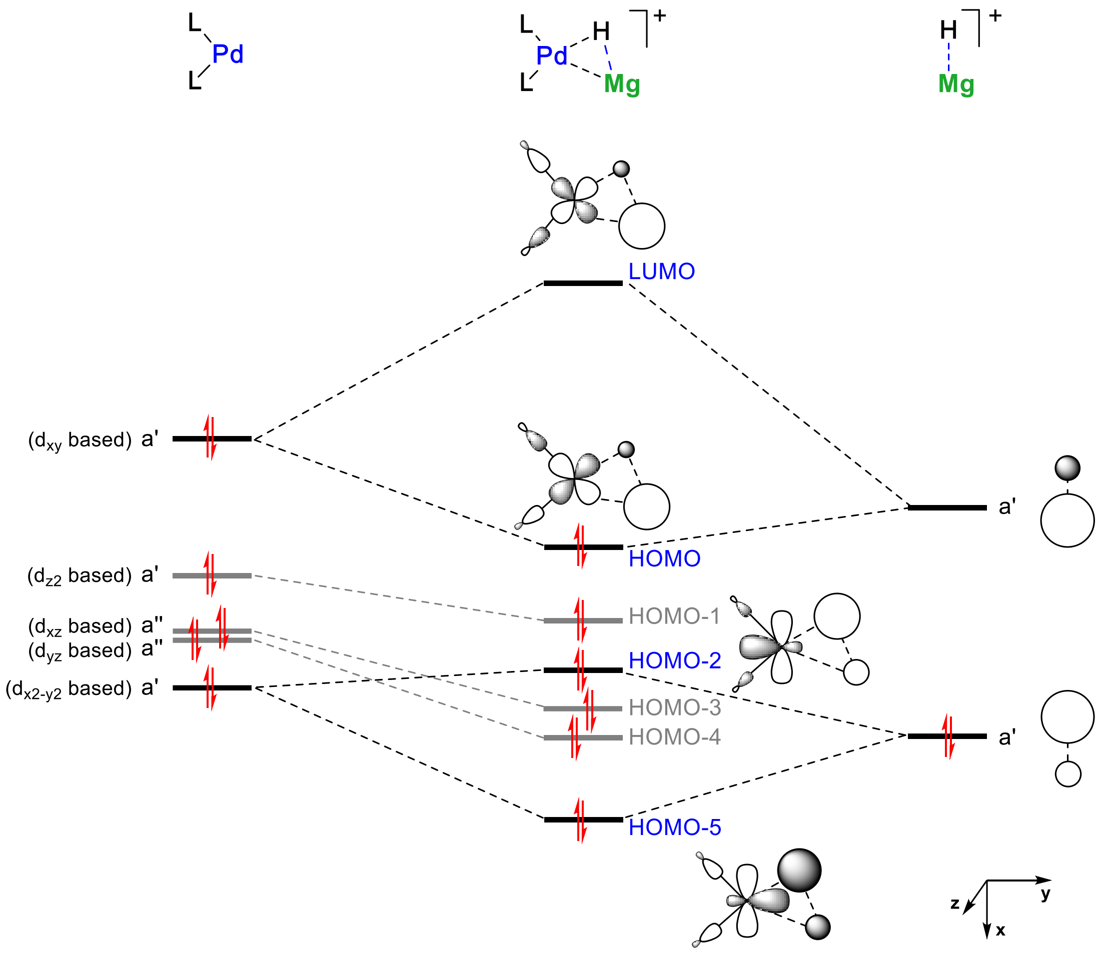

Figure S13. Qualitative MO diagram (idealised to C_s symmetry) of a model of **2a** constructed from interaction of a bent PdL_2 fragment with a $\{MgH\}^+$ unit.

Table S5. Relevant contributions of atomic orbitals to the key bonding molecular orbitals of a model of **2a** (contributions from P and C atoms have been omitted for clarity).

MO	AO contributions (from NBO analysis)	AO contributions (from NBO analysis)
LUMO	**Mg**: s = 22%, p = 48%<br>**Pd**: d = 9%, p = 3%, s = 2%<br>**H**: s = 7%	**Mg**: s = 4%<br>**Pd**: d = 29%, p = 5%<br>**H**: s = 20%
HOMO	**Mg**: s = 38%, p = 2%<br>**Pd**: d = 15%, p = 8%, s = 4%	**Mg**: s = 4%, p = 2%<br>**Pd**: d = 54%<br>**H**: s = 4%

Table S6. Main donor-acceptor interactions from NBO second order perturbation analysis for complexes **2a** and **2b**.

Complex	**Orbitals involved** *(donor → acceptor)*	**Energy of the interaction (kcal·mol^{-1})**
**2a**	Pd d → σ* (Mg-H)	28.5
	σ (Mg-H) → Pd s	300.3
**2b**	Pd d → σ* (Zn-H)	37.3
	σ (Zn-H) → Pd s	262.2

*Analysis of **5' (model)***

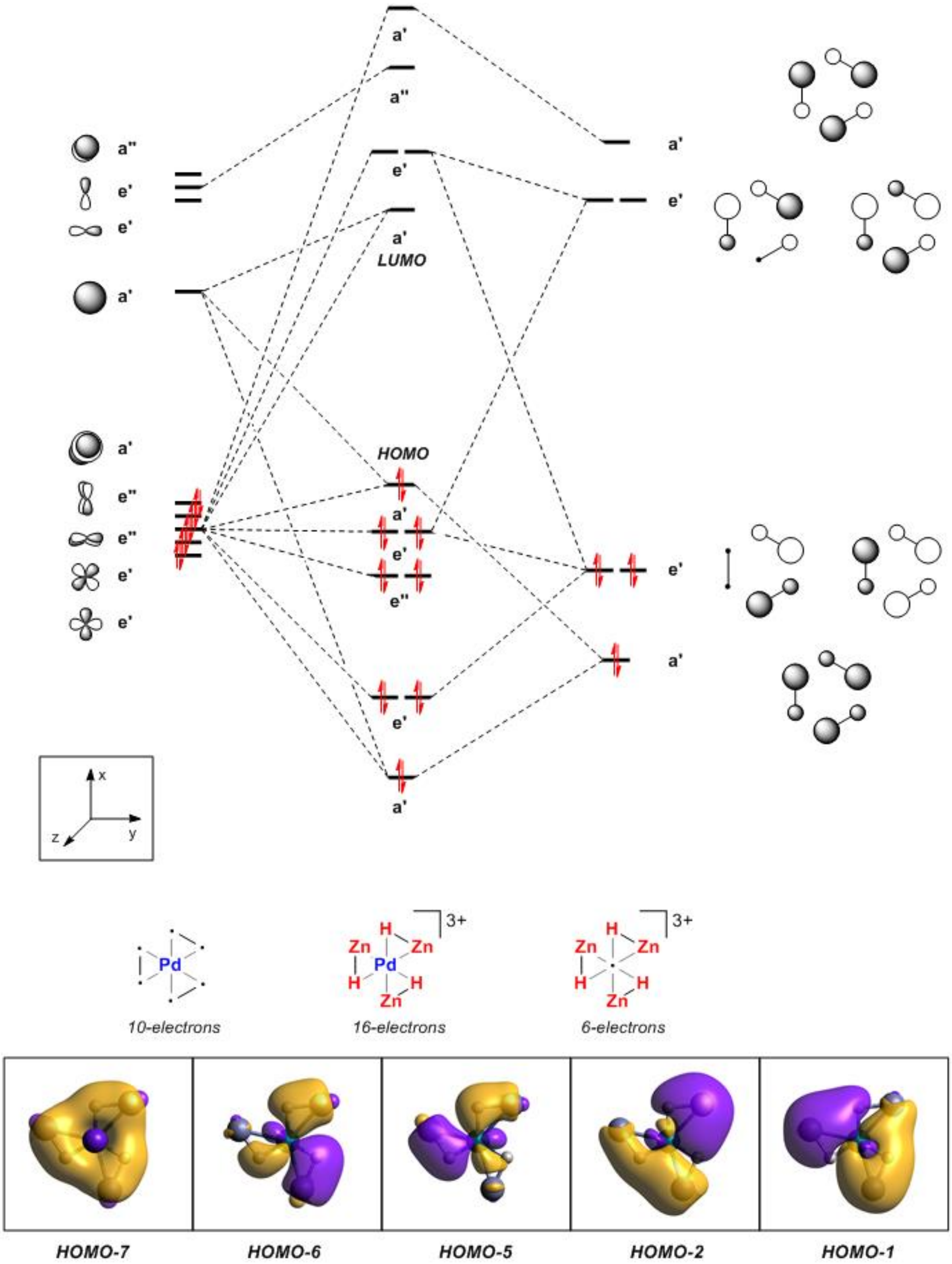

Figure S14. Qualitative MO diagram (idealised to C_{3h} symmetry) of **5'** constructed from interaction of a Pd fragment with three $\{ZnH\}^{+}$ units.

Table S7. Relevant contributions of atomic orbitals to the key bonding molecular orbitals of **5’**

MO	AO contributions (from NBO analysis)	MO	AO contributions (from NBO analysis)
LUMO	**Zn**: s = 57.2%, p = 1.4%<br>**Pd**: d = 0%, p = 0%, s = 0%<br>**H**: s = 19.7%	*HOMO-5*	**Zn**: s = 10.3%, p = 0.3%<br>**Pd**: d = 49.7%, p = 0.1%, s = 0%<br>**H**: s = 30.4%
HOMO	**Zn**: s = 0%, p = 0%<br>**Pd**: d = 80.6%, p = 0%, s = 0.3%<br>**H**: s = 0%	*HOMO-6*	**Zn**: s = 9.6%, p = 0.2%<br>**Pd**: d = 49.8%, p = 0.1%, s = 0%<br>**H**: s = 28.5%
HOMO-1	**Zn**: s = 20.1%, p = 0.5%<br>**Pd**: d = 38.0%, p = 0.1%, s = 0%<br>**H**: s = 33.8%	*HOMO-7*	**Zn**: s = 13.8%, p = 0.3%<br>**Pd**: d = 16.0%, p = 0.4%, s = 97.1%<br>**H**: s = 40.9%
HOMO-2	**Zn**: s = 17.4%, p = 0.4%<br>**Pd**: d = 38.1%, p = 0.1%, s = 0%<br>**H**: s = 34.6%		

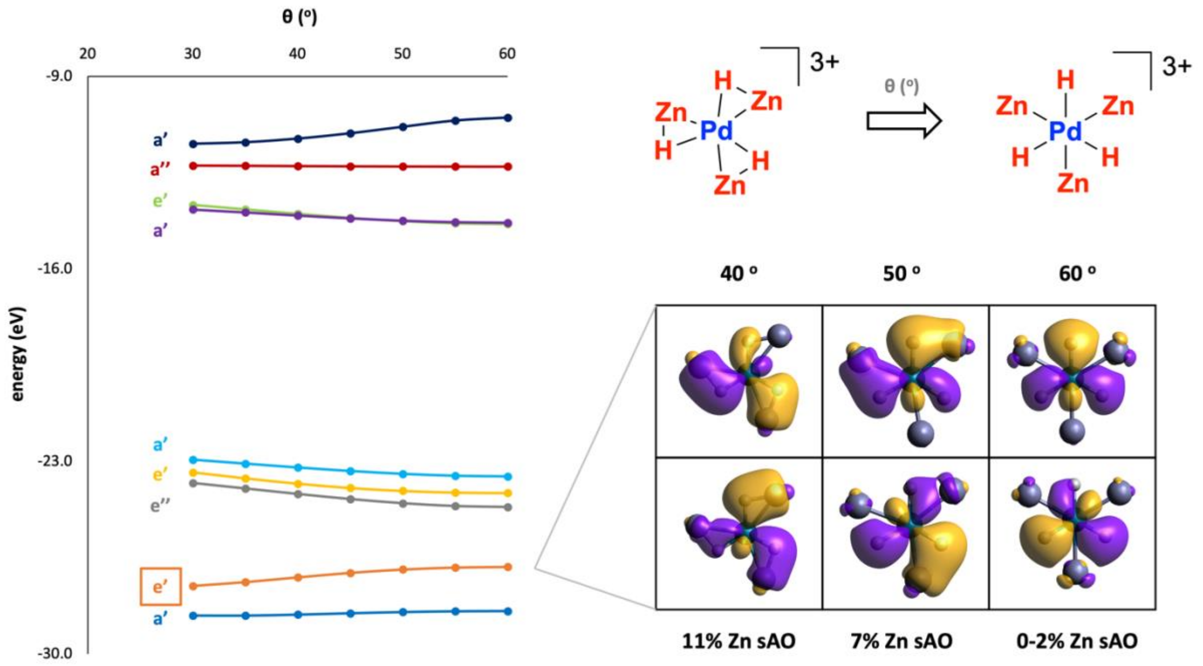

Figure S15. (a) Walsh diagram and (b) snapshots at different values of θ for the key e’ set (HOMO-5 and HOMO-6) of orbitals of a model of 5, showcasing the decrease in Zn–H interactions upon extension.

8.4. Symmetry Elements for the Hexagonal Planar Geometry

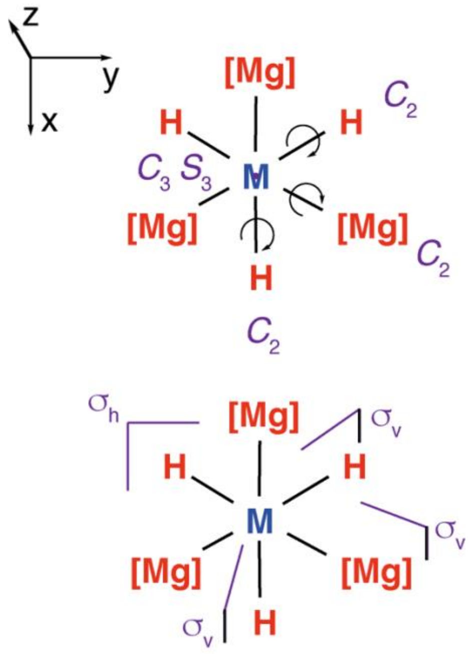

Figure S16. Symmetry elements for **6a** and **6b** (D_{3h} symmetry).

8.5. PES for Hexagonal and Trigonal Planar Geometries

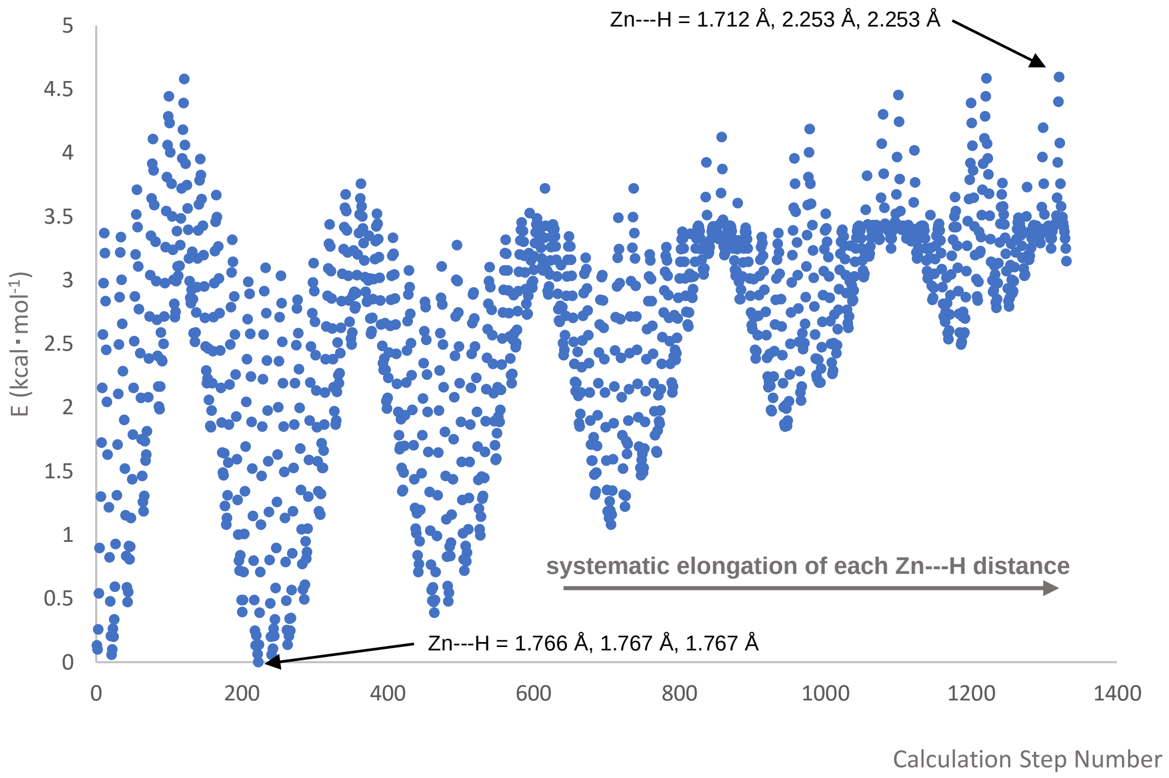

Figure S17. Energy change upon systematic elongation of each Zn---H distance in $PdZn_3H_3$ model. Global minimum and maximum points are highlighted. This is a 1D representation of the 4D PES presented in the paper.

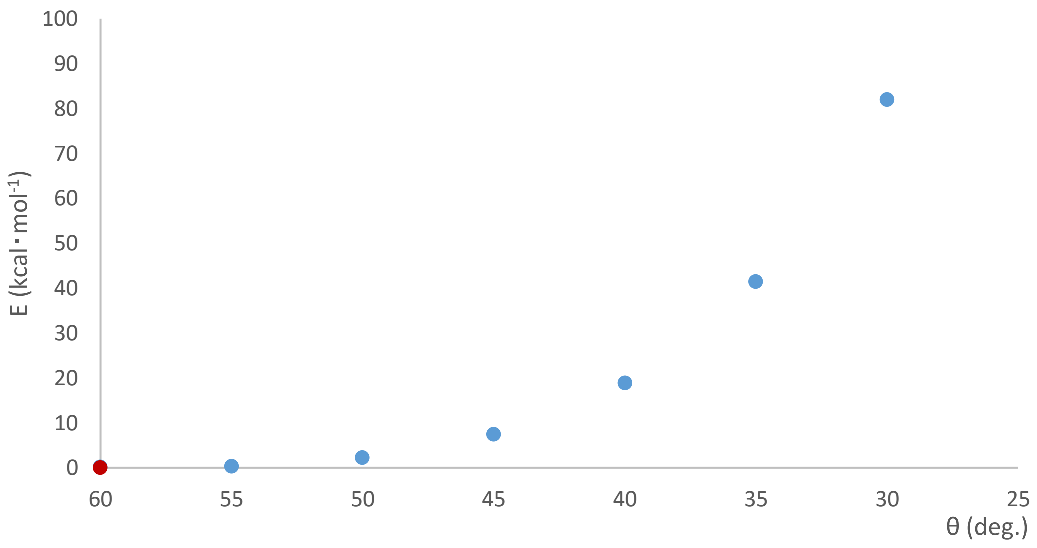

Figure S18. Energy change upon symmetric compression of the Mg---H distances in **6a**. This compression is plotted in terms of the angle θ described in the paper and Figure 1. The red dot shows the calculated lowest energy θ value for **6a**.

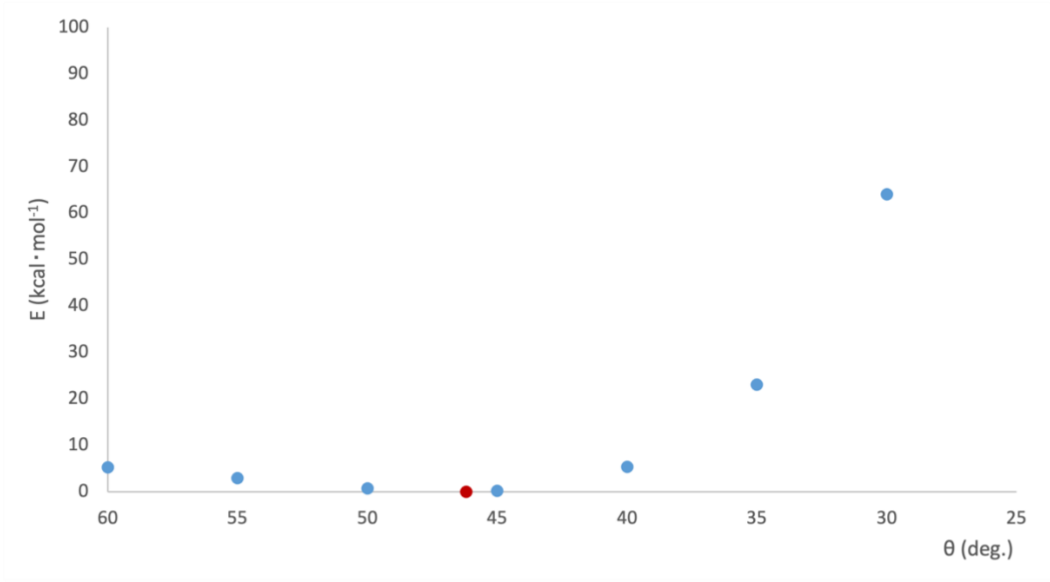

Figure S19. Energy change upon symmetric compression and elongation away from the optimised Zn–H distances in **5**. This change is plotted in terms of the angle θ described in the paper and Figure 1. The red dot shows the calculated lowest energy θ value for **5**.

The trends observed in the 4D PES for both the $PtMg_3H_3$ and $PdZn_3H_3$ model systems calculated at the BS3 level of theory remain consistent when calculated using a much smaller basis set (H: 6-31G; Mg, Zn, Pd, Pt: SDDAll). The largest calculated energy differences using either level of theory are similar ($PtMg_3H_3$: +19.4 kcal mol^{-1} (low) and +14.6 kcal mol^{-1} (BS3); $PdZn_3H_3$: +5.5 kcal mol^{-1} (low) and +4.6 kcal mol^{-1} (BS3)). The 4D PES for both are presented below.

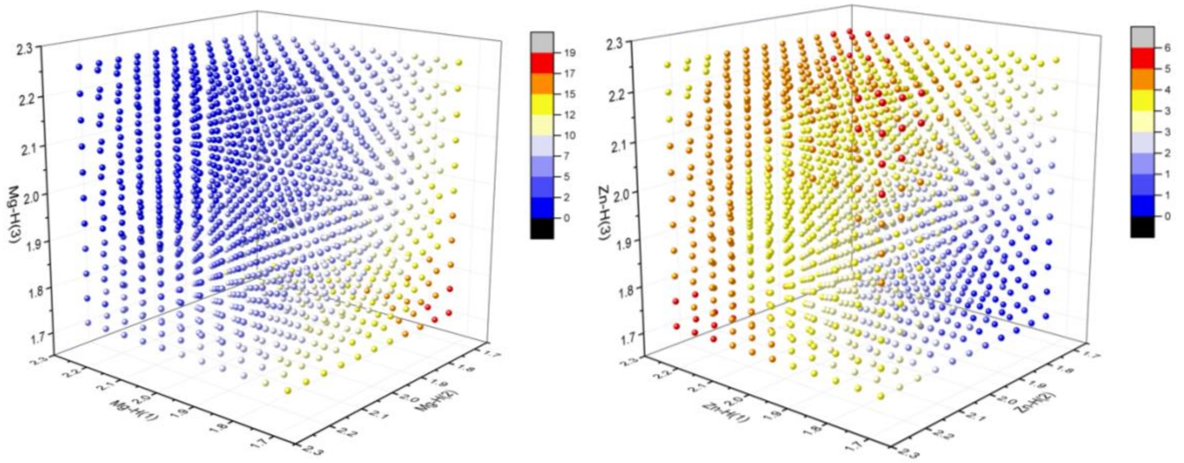

Figure S20. Potential energy surface for $PtMg_3H_3$ (left) and $PdZn_3H_3$ (right) model systems calculated using small basis sets (scale in kcal mol^{-1}).

8.6. Vibrational analysis for Hexagonal and Trigonal Planar Geometries

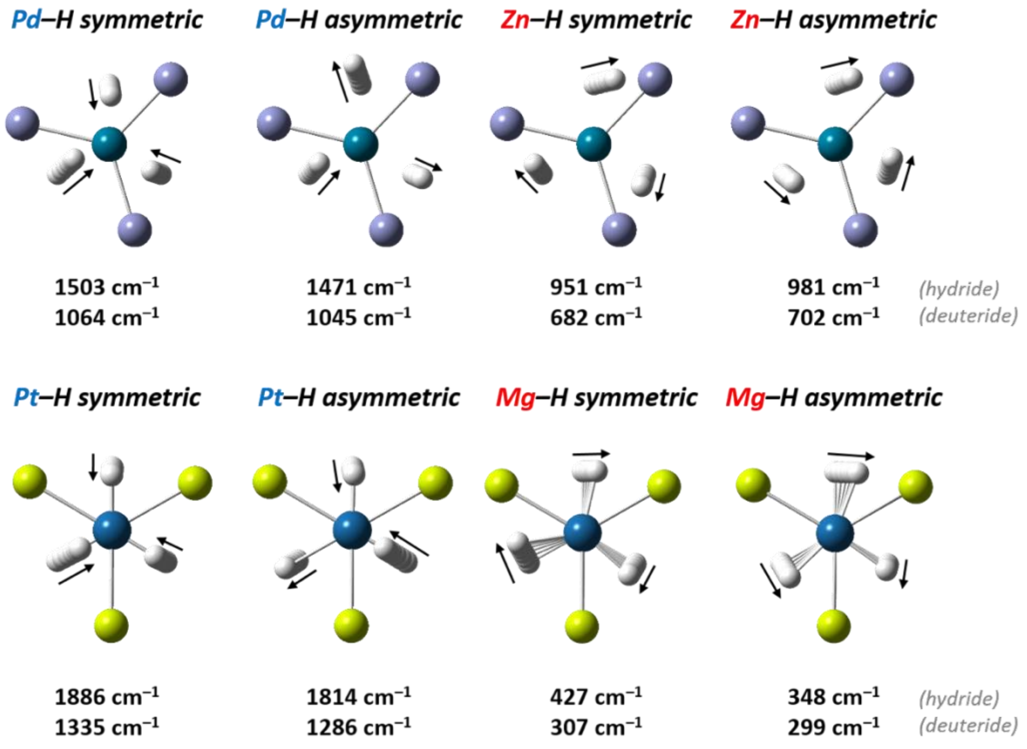

Figure S21. Computed vibrational modes for models **5'** and **6b'** including associated wavenumbers for both hydride and deuteride analogues

The computed stretching frequencies of various vibrational modes for simplified models **5'** and **6b'** were analysed. The models, consisting of only metals and metal hydrides ($\{PdZn_3H_3\}^{3+}$, **5'** and $\{PtMg_3H_3\}^{3+}$, **6b'**) were calculated at the BS3 level of theory (see computational methods). Frequency calculations revealed 15 vibrational modes in each case. The symmetric and asymmetric M---H stretches (M = Pd or Zn (**5'**) and Pt or Mg (**6b'**)) are presented in Figure S21 along with their calculated wavenumbers for both metal hydrides and deuterides.

Assuming a highly simplified harmonic oscillator, the stretching frequencies can be rationalised by considering the reduced mass of the bond which is vibrating. A comparison of

the calculated wavenumbers for each M---H mode against the corresponding M---D mode are in good agreement (0.2 – 0.6%) with the approximation below:

$$\nu_2 = \sqrt{\frac{\nu_1^2 \mu_1}{\mu_2}} \qquad \text{(eq. 1)}$$

where ν_1 and μ_1 are the computed wavenumber and reduced mass of the reference vibration, respectively, and ν_2 is the wavenumber to be calculated.

The much lower computed wavenumbers for the Mg–H and Mg–D stretch relative to Zn–H and Zn–D stretch cannot be accounted for by differences in reduced mass alone. Rather the computed values are consistent with considerably weaker interaction, and smaller force constant, in Mg---H than in Zn---H.

To unequivocally confirm the signal at 1705 cm^{-1} to be the terminal Pt–H stretch in **6b**, the deuterium isotopologue (d^3-**6b**) was prepared, confirming disappearance of this signal by both ^1H NMR and IR spectroscopies. Based on computed Pt–H stretches, the experimentally observed Pt–D stretch would be expected to be around 1201 cm^{-1}, within the fingerprint region of the spectrum. Unfortunately, no definitive Pt–D stretch could be identified due to overlapping peaks and likely broadening.

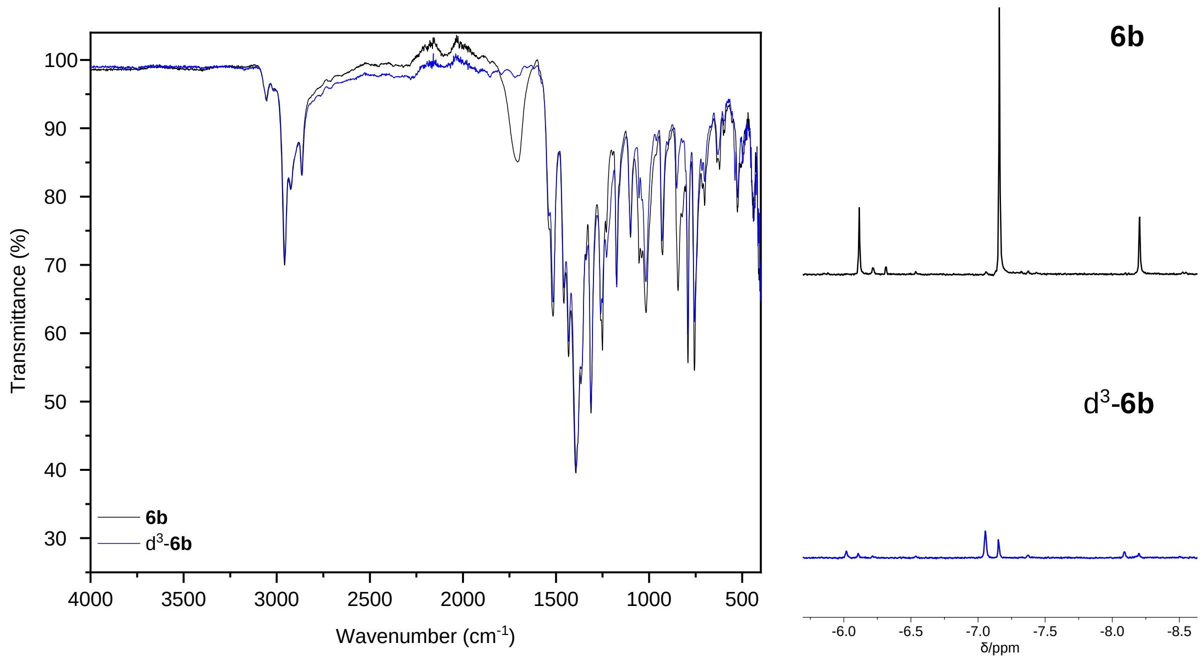

Figure S22. Comparison of infrared (left) and ^1H NMR (right) spectra of **6b** and d^3-**6b**

9. XYZ Coordinates

1

SCF (wB97x) = -1240.51583393
E(SCF)+ZPE(0 K)= -1239.862364
H(298 K)= -1239.825287
G(298 K)= -1239.932430
Lowest Frequency = 9.8772cm-1

N	9.078906	4.681070	3.180555
C	7.869377	5.047131	2.773845
C	7.231752	6.245049	3.150072
H	6.247169	6.402326	2.724647
C	7.677528	7.248434	4.031716
N	8.854986	7.239387	4.644867
C	7.095901	4.129016	1.851663
H	7.665040	3.933949	0.935905
H	6.129138	4.558144	1.581824
H	6.929214	3.157744	2.331291
C	6.725119	8.396803	4.288168
H	6.465520	8.447850	5.351754
H	5.807132	8.289923	3.707249
H	7.196610	9.352493	4.033320
C	9.612231	3.414058	2.772619
C	10.409643	3.332349	1.614986
C	10.996412	2.107513	1.294252
H	11.621519	2.030263	0.407170
C	10.804103	0.987348	2.091463
H	11.272163	0.042309	1.827899
C	10.015633	1.081721	3.230581
H	9.874595	0.202890	3.856140
C	9.409039	2.285392	3.590898
C	10.677009	4.551739	0.743526
H	9.972236	5.337293	1.038316
C	12.096071	5.084703	0.986576
H	12.267419	5.315695	2.045685
H	12.275687	5.995134	0.402132
H	12.845568	4.339843	0.692447
C	10.449974	4.272558	-0.746299
H	10.563415	5.195401	-1.326569
H	9.445434	3.874928	-0.931723
H	11.173277	3.548351	-1.138745
C	8.589130	2.369439	4.871557
H	8.028217	3.310556	4.851693
C	7.566991	1.234373	4.995148
H	6.912829	1.187482	4.116925
H	6.938520	1.384913	5.880373
H	8.053695	0.257995	5.102030
C	9.512026	2.405172	6.097959
H	10.082831	1.472266	6.181239
H	8.932031	2.532346	7.020311
H	10.241132	3.223533	6.035925
C	9.185774	8.289458	5.563625
C	8.888862	8.126885	6.931295
C	9.303812	9.113285	7.826333
H	9.088800	8.998845	8.886605
C	9.994882	10.235568	7.388864
H	10.313982	10.992475	8.100831
C	10.281341	10.382899	6.038559
H	10.829968	11.260077	5.701948
C	9.886051	9.422169	5.106669
C	8.175718	6.883824	7.446672
H	7.739183	6.364490	6.586189
C	7.028584	7.214566	8.407406
H	7.393524	7.665095	9.337704
H	6.485508	6.301735	8.677645
H	6.315986	7.913495	7.954514
C	9.177597	5.926009	8.106887
H	9.998615	5.662528	7.427409
H	8.683079	4.998993	8.422131
H	9.630050	6.388245	8.992709
C	10.254151	9.594267	3.639493
H	9.690804	8.852591	3.062152
C	11.748295	9.315425	3.423607
H	12.361481	10.042260	3.970240
H	12.007747	9.385282	2.360347
H	12.031163	8.317672	3.782554
C	9.875778	10.976751	3.096537
H	8.813599	11.194421	3.257811
H	10.074402	11.028931	2.019932
H	10.456217	11.773798	3.575604
Mg	10.264761	5.798818	4.394334
H	11.862192	5.603861	4.978838

2a

SCF (wB97x) = -3270.13166716
E(SCF)+ZPE(0 K)= -3268.758409
H(298 K)= -3268.689251
G(298 K)= -3268.864927
Lowest Frequency = 7.7918cm-1

Pd	0.933721	-0.408713	-0.112826
Mg	-1.339837	0.184827	-1.095783
H	0.377115	-0.445706	-1.715654
N	-2.009037	1.981241	-1.875453
C	-2.965126	2.046578	-2.783479
C	-3.806793	0.961866	-3.121662
H	-4.519759	1.161627	-3.915374
C	-3.942696	-0.281804	-2.479750
N	-3.141498	-0.724182	-1.515635
C	-3.218941	3.323249	-3.563813
H	-2.726355	4.190548	-3.120035
H	-4.292210	3.521142	-3.640514
H	-2.835622	3.198109	-4.583793

C	-5.117285	-1.134821	-2.915763
H	-4.805757	-2.167813	-3.100576
H	-5.580321	-0.734778	-3.820198
H	-5.877506	-1.168827	-2.126198
C	-1.295025	3.144178	-1.450530
C	-1.796167	3.888753	-0.360494
C	-1.053556	4.973879	0.106670
H	-1.435215	5.567625	0.934838
C	0.166876	5.311079	-0.465025
H	0.732875	6.158969	-0.086435
C	0.653973	4.563209	-1.528358
H	1.606623	4.834294	-1.979674
C	-0.059249	3.477288	-2.041312
C	-3.141896	3.562598	0.277140
H	-3.443980	2.567218	-0.069802
C	-3.076187	3.512416	1.806721
H	-2.342049	2.772911	2.148695
H	-4.053184	3.235338	2.219460
H	-2.804398	4.483383	2.237960
C	-4.215393	4.560831	-0.177822
H	-3.961084	5.578552	0.144021
H	-5.191081	4.303753	0.251723
H	-4.317003	4.571843	-1.268423
C	0.495118	2.724438	-3.243197
H	-0.144550	1.852427	-3.419392
C	0.451085	3.602916	-4.501346
H	-0.561678	3.969386	-4.702930
H	0.791378	3.038069	-5.377316
H	1.104491	4.477482	-4.391573
C	1.915755	2.204012	-2.998310
H	2.624704	3.027433	-2.839995
H	2.261572	1.628617	-3.866268
H	1.938648	1.541789	-2.125765
C	-3.485341	-1.916845	-0.805248
C	-2.857845	-3.138113	-1.123312
C	-3.158091	-4.266545	-0.356067
H	-2.684311	-5.215864	-0.599615
C	-4.048276	-4.202103	0.706401
H	-4.267510	-5.090888	1.293363
C	-4.658561	-2.992115	1.013997
H	-5.355867	-2.944429	1.847994
C	-4.397573	-1.841170	0.270377
C	-1.878150	-3.272218	-2.280943
H	-1.761211	-2.284893	-2.741467
C	-2.410566	-4.224855	-3.359262
H	-2.530617	-5.242295	-2.967341
H	-1.714281	-4.272580	-4.204680
H	-3.384807	-3.898507	-3.741212
C	-0.494562	-3.719353	-1.794456
H	-0.098019	-3.015214	-1.053095
H	0.212659	-3.752060	-2.633317
H	-0.531689	-4.720134	-1.344308
C	-5.073910	-0.530376	0.653253
H	-4.960139	0.169927	-0.180962
C	-4.382542	0.099044	1.868534
H	-4.412692	-0.578837	2.731598
H	-4.875940	1.036067	2.154311
H	-3.329030	0.320563	1.656119
C	-6.576195	-0.693267	0.911852
H	-7.077967	-1.184045	0.069852
H	-7.042113	0.287533	1.061199
H	-6.774660	-1.288490	1.810905
P	3.293614	-0.905044	-0.064058
C	3.679022	-1.177254	1.741975
H	3.560071	-2.252846	1.913689
H	4.730613	-0.950696	1.960717
C	2.769009	-0.398809	2.709615
H	3.137358	0.630441	2.795815
H	2.838780	-0.834308	3.715575
P	0.970775	-0.259221	2.187516
C	4.465145	0.449548	-0.581798
H	4.247627	0.585521	-1.653333
C	4.104262	1.772293	0.114614
H	3.034529	1.985742	-0.008416
H	4.287658	1.674750	1.196344
C	4.945403	2.934311	-0.419776
H	4.699457	3.096082	-1.479970
H	4.683324	3.859107	0.109567
C	6.442113	2.646784	-0.284807
H	6.703451	2.589150	0.782551
H	7.030509	3.469906	-0.708523
C	6.811432	1.326488	-0.963836
H	7.877401	1.108838	-0.822173
H	6.650496	1.420576	-2.048073
C	5.966295	0.161649	-0.434547
H	6.241059	-0.759055	-0.963915
H	6.208681	-0.001467	0.626478
C	4.020283	-2.429145	-0.847464
H	5.038304	-2.588778	-0.456536
C	4.097241	-2.256397	-2.374254
H	4.745744	-1.410759	-2.635954
H	3.092372	-2.009681	-2.749654
C	4.610125	-3.521312	-3.069605
H	4.621414	-3.367230	-4.155698
H	5.652110	-3.704110	-2.766415
C	3.762180	-4.742414	-2.712350
H	2.743708	-4.603307	-3.104606
H	4.166525	-5.642529	-3.191641
C	3.692917	-4.928644	-1.196335
H	4.696476	-5.167948	-0.813588
H	3.048147	-5.779998	-0.944761
C	3.171025	-3.665729	-0.505049
H	3.140289	-3.830229	0.579721
H	2.135175	-3.478872	-0.821633
C	0.177933	-1.631284	3.164483
H	0.475842	-1.509471	4.217864
C	0.686732	-3.002616	2.690727
H	0.466263	-3.114105	1.617825
H	1.777216	-3.062978	2.799520
C	0.036214	-4.147387	3.473145

H	0.400923	-5.109810	3.092474
H	0.346560	-4.087024	4.527365
C	-1.488955	-4.084645	3.388656
H	-1.937632	-4.891279	3.982300
H	-1.801294	-4.242177	2.346499
C	-2.008514	-2.725749	3.859736
H	-3.098295	-2.675896	3.736454
H	-1.802343	-2.606729	4.934350
C	-1.354904	-1.578623	3.083229
H	-1.727942	-0.617668	3.462643
H	-1.658184	-1.641217	2.027331
C	0.454476	1.286691	3.097780
H	-0.640789	1.315474	2.983461
C	0.776448	1.303495	4.600621
H	1.861275	1.193847	4.746930
H	0.302876	0.451322	5.104145
C	0.322579	2.606623	5.266521
H	-0.774280	2.676790	5.214240
H	0.587236	2.591141	6.331186
C	0.936816	3.824789	4.576533
H	2.030528	3.794729	4.695489
H	0.592935	4.749523	5.056252
C	0.585110	3.833230	3.088713
H	1.050645	4.688692	2.583277
H	-0.500505	3.960696	2.977645
C	1.014376	2.537005	2.394624
H	2.114476	2.496037	2.386946
H	0.694671	2.546112	1.343574

2b

SCF (wB97x) = -4849.50272730
E(SCF)+ZPE(0 K)= -4848.128772
H(298 K)= -4848.059609
G(298 K)= -4848.233962
Lowest Frequency = 9.1108cm-1

Pd	-0.858395	0.448530	-0.051956
Zn	1.265121	-0.255873	-1.047217
H	-0.322086	0.432664	-1.634931
N	1.739548	-2.073913	-1.845186
C	2.687918	-2.223724	-2.747431
C	3.619830	-1.216858	-3.084037
H	4.319625	-1.475967	-3.872096
C	3.848413	0.008249	-2.439575
N	3.078888	0.512541	-1.481020
C	2.833704	-3.519528	-3.523956
H	2.247809	-4.335047	-3.096049
H	3.884465	-3.820796	-3.572633
H	2.492421	-3.356185	-4.553396
C	5.087498	0.770128	-2.865966
H	4.853583	1.820641	-3.066117
H	5.530310	0.327238	-3.760626
H	5.838655	0.758222	-2.067409
C	0.915470	-3.159995	-1.423796
C	1.329486	-3.939056	-0.322497
C	0.474424	-4.936345	0.148626
H	0.784252	-5.555554	0.988085
C	-0.768361	-5.153899	-0.433571
H	-1.423206	-5.932937	-0.050133
C	-1.161078	-4.381294	-1.518709
H	-2.128129	-4.565578	-1.983726
C	-0.333363	-3.382565	-2.036582
C	2.702168	-3.751091	0.313054
H	3.099678	-2.788802	-0.029543
C	2.651673	-3.704308	1.842481
H	1.995966	-2.898323	2.192034
H	3.654014	-3.525907	2.248917
H	2.290874	-4.647606	2.269997
C	3.667994	-4.850244	-0.151537
H	3.313167	-5.838389	0.167553
H	4.666240	-4.694814	0.274973
H	3.763969	-4.867354	-1.242445
C	-0.774861	-2.607070	-3.270134
H	-0.038803	-1.815952	-3.449561
C	-0.802531	-3.520377	-4.503808
H	0.166535	-4.003384	-4.671357
H	-1.058651	-2.946472	-5.402343
H	-1.552169	-4.313081	-4.387289
C	-2.133208	-1.926817	-3.075115
H	-2.933020	-2.662220	-2.916626
H	-2.394028	-1.338468	-3.964069
H	-2.099879	-1.248083	-2.216500
C	3.512530	1.671283	-0.766610
C	3.001688	2.940455	-1.100092
C	3.392109	4.042519	-0.335707
H	3.006757	5.028766	-0.588705
C	4.263702	3.905013	0.735743
H	4.555927	4.774222	1.320356
C	4.763523	2.647907	1.053975
H	5.449446	2.544384	1.892345
C	4.407159	1.519792	0.314572
C	2.049451	3.145461	-2.268881
H	1.883077	2.171130	-2.740535
C	2.645213	4.082015	-3.327618
H	2.810734	5.088352	-2.923847
H	1.964819	4.174111	-4.182336
H	3.607261	3.710848	-3.699645
C	0.688839	3.660462	-1.787168
H	0.252782	2.965991	-1.059304
H	-0.009375	3.746879	-2.630155
H	0.777678	4.649823	-1.318769
C	4.966511	0.156449	0.701979
H	4.806633	-0.528844	-0.137041
C	4.206866	-0.417882	1.903376
H	4.284717	0.250677	2.770909
H	4.615412	-1.394880	2.189332
H	3.142048	-0.545910	1.672294
C	6.473230	0.192544	0.981962
H	7.026496	0.643565	0.149942

H	6.853182	-0.824566	1.132051
H	6.707921	0.764346	1.887513
P	-3.164359	1.098306	-0.041997
C	-3.576138	1.436761	1.745248
H	-3.389791	2.505597	1.896023
H	-4.645724	1.285765	1.938926
C	-2.743924	0.623642	2.751841
H	-3.184863	-0.374388	2.858638
H	-2.800398	1.091195	3.743949
P	-0.952944	0.351969	2.268461
C	-4.396480	-0.202141	-0.558784
H	-4.182381	-0.355248	-1.628033
C	-4.105217	-1.536957	0.147098
H	-3.048826	-1.808500	0.022050
H	-4.278839	-1.421391	1.228563
C	-5.008342	-2.657910	-0.373807
H	-4.771434	-2.846508	-1.431515
H	-4.795503	-3.588509	0.167294
C	-6.487426	-2.288774	-0.245481
H	-6.747176	-2.201624	0.820271
H	-7.118678	-3.084883	-0.658914
C	-6.783086	-0.960547	-0.944503
H	-7.836786	-0.684888	-0.812757
H	-6.620775	-1.077588	-2.026289
C	-5.881457	0.165172	-0.424133
H	-6.104554	1.091757	-0.967471
H	-6.122924	0.353632	0.632759
C	-3.793303	2.639236	-0.875331
H	-4.813025	2.848089	-0.513465
C	-3.841543	2.440470	-2.399954
H	-4.519587	1.618151	-2.661240
H	-2.839917	2.144343	-2.747121
C	-4.283908	3.712151	-3.130431
H	-4.275206	3.537879	-4.213441
H	-5.324410	3.943536	-2.856840
C	-3.394534	4.903481	-2.773771
H	-2.373373	4.714989	-3.137282
H	-3.748777	5.810231	-3.279357
C	-3.355663	5.115793	-1.260139
H	-4.357773	5.403392	-0.908066
H	-2.682708	5.944595	-1.007397
C	-2.904073	3.846625	-0.531546
H	-2.897232	4.031548	0.550222
H	-1.868700	3.613463	-0.814564
C	-0.085097	1.694604	3.218410
H	-0.391039	1.610423	4.273220
C	-0.511117	3.084852	2.718865
H	-0.282350	3.165240	1.644901
H	-1.596018	3.212812	2.824835
C	0.206144	4.201009	3.484107
H	-0.100386	5.177317	3.087874
H	-0.108129	4.175652	4.538613
C	1.724837	4.045952	3.402439
H	2.220462	4.833950	3.983647
H	2.045471	4.167914	2.358223
C	2.162794	2.666254	3.895468
H	3.247394	2.549209	3.772825
H	1.951210	2.577618	4.972035
C	1.441369	1.547247	3.138437
H	1.754187	0.572615	3.536384
H	1.746929	1.568825	2.081767
C	-0.553070	-1.202563	3.217986
H	0.538295	-1.310621	3.116429
C	-0.892179	-1.157978	4.716707
H	-1.965998	-0.956896	4.846711
H	-0.355907	-0.335536	5.206542
C	-0.555484	-2.477615	5.418525
H	0.532257	-2.639360	5.383744
H	-0.831317	-2.414327	6.478543
C	-1.259945	-3.657158	4.747832
H	-2.348966	-3.532430	4.846749
H	-1.002567	-4.594897	5.255588
C	-0.885666	-3.732214	3.267497
H	-1.411592	-4.558957	2.773367
H	0.187346	-3.950464	3.182199
C	-1.196153	-2.423660	2.534489
H	-2.289187	-2.297939	2.503471
H	-0.858818	-2.483958	1.490947

3a

SCF (wB97x) = -1466.79656213
E(SCF)+ZPE(0 K)= -1466.142298
H(298 K)= -1466.105165
G(298 K)= -1466.212380
Lowest Frequency = 9.4843cm-1

N	9.022172	4.655857	3.244742
C	7.813302	5.013857	2.831759
C	7.162972	6.198263	3.221242
H	6.176755	6.353453	2.799158
C	7.621903	7.207893	4.085774
N	8.800566	7.190273	4.694577
C	7.067199	4.104515	1.880046
H	7.642197	3.956463	0.959068
H	6.090148	4.518050	1.622921
H	6.925693	3.113782	2.326370
C	6.694588	8.379866	4.322379
H	6.455415	8.472130	5.387781
H	5.765040	8.267557	3.761067
H	7.176521	9.317908	4.024802
C	9.602820	3.426763	2.790466
C	10.415277	3.430518	1.641155
C	11.044490	2.241929	1.269225
H	11.682582	2.226873	0.388452
C	10.876968	1.078597	2.008709
H	11.377989	0.162626	1.705800
C	10.069808	1.090685	3.138469
H	9.947286	0.177333	3.716399
C	9.420326	2.255464	3.549096

C	10.653210	4.704617	0.842138
H	9.882883	5.429835	1.126797
C	12.015054	5.314474	1.202217
H	12.097401	5.505387	2.279225
H	12.170074	6.262187	0.672119
H	12.829172	4.632837	0.926452
C	10.537140	4.487786	-0.670273
H	10.612225	5.446534	-1.195957
H	9.579315	4.026476	-0.937559
H	11.337226	3.843363	-1.052704
C	8.579114	2.255048	4.818169
H	7.966037	3.163066	4.816154
C	7.622872	1.060262	4.895818
H	6.990243	0.993590	4.003053
H	6.969254	1.155827	5.770373
H	8.162808	0.111401	4.994930
C	9.482288	2.312179	6.057980
H	10.114333	1.417529	6.117874
H	8.882539	2.367354	6.974745
H	10.148108	3.183402	6.029534
C	9.178919	8.267758	5.560648
C	8.896273	8.178858	6.936565
C	9.354289	9.195569	7.775340
H	9.152412	9.140583	8.842762
C	10.070807	10.273429	7.272396
H	10.422808	11.054575	7.941508
C	10.338810	10.348032	5.911988
H	10.905262	11.192332	5.525393
C	9.901092	9.355240	5.034577
C	8.154521	6.983195	7.518368
H	7.662874	6.457271	6.692498
C	7.062596	7.388845	8.513869
H	7.483638	7.840956	9.419423
H	6.489965	6.507976	8.825878
H	6.365011	8.110382	8.072920
C	9.143922	6.003276	8.164130
H	9.911456	5.679108	7.450694
H	8.623157	5.112864	8.537184
H	9.659792	6.475326	9.009415
C	10.248574	9.435059	3.554314
H	9.604590	8.728975	3.018512
C	11.702125	8.999600	3.322438
H	12.395863	9.682207	3.828644
H	11.943288	9.000731	2.252369
H	11.887090	7.992454	3.715624
C	9.995191	10.823966	2.958810
H	8.967386	11.158155	3.142119
H	10.159715	10.805943	1.875396
H	10.671942	11.577435	3.378297
Zn	10.111151	5.734094	4.483355
H	11.506293	5.513594	5.080859

3b

SCF (wB97x) = -1230.98337177

E(SCF)+ZPE(0 K)= -1230.504485
H(298 K)= -1230.474150
G(298 K)= -1230.567836
Lowest Frequency = 11.3898cm-1

N	9.057938	4.649278	3.258462
C	7.848816	5.007427	2.844882
C	7.197209	6.191120	3.233818
H	6.211048	6.345260	2.811182
C	7.654409	7.201063	4.098649
N	8.833165	7.186049	4.708255
C	7.101669	4.098441	1.891764
H	7.674393	3.949236	0.970062
H	6.125511	4.514455	1.635044
H	6.956494	3.107598	2.335632
C	6.722823	8.371376	4.334176
H	6.480135	8.464748	5.398259
H	5.794583	8.254864	3.771463
H	7.199490	9.311638	4.036707
C	9.630613	3.421237	2.801481
C	10.435559	3.416600	1.653495
C	11.018205	2.216670	1.248430
H	11.638593	2.209854	0.352742
C	10.834531	1.032587	1.960016
C	10.044194	1.070981	3.107265
H	9.893958	0.157674	3.682162
C	9.443023	2.249065	3.547667
C	9.201050	8.265821	5.570575
C	8.915995	8.186194	6.941094
C	9.319754	9.230735	7.771269
H	9.093174	9.173740	8.835515
C	10.008791	10.337559	7.278902
C	10.294225	10.381053	5.915511
H	10.838579	11.234104	5.511550
C	9.908978	9.358410	5.050013
Zn	10.144710	5.730403	4.496889
H	11.539369	5.512384	5.094353
C	8.200191	6.985040	7.504879
H	7.223310	6.828162	7.032308
H	8.781147	6.068610	7.340593
H	8.042446	7.095762	8.581951
C	10.258299	9.415221	3.584499
H	10.898824	8.570698	3.300273
H	9.368026	9.364573	2.946421
H	10.793741	10.339486	3.347311
C	10.465057	11.440143	8.201987
H	9.789008	11.550466	9.056209
H	11.465711	11.230994	8.599507
H	10.513249	12.402044	7.681090
C	10.676671	4.690089	0.883376
H	11.178027	5.438937	1.509619
H	11.308589	4.503277	0.009870
H	9.741441	5.144737	0.535684
C	8.619242	2.269717	4.810191
H	9.059477	2.944241	5.555551

H	7.597138	2.623166	4.629649
H	8.559818	1.270830	5.252825
C	11.502342	-0.247104	1.521426
H	11.641511	-0.272578	0.435666
H	12.492110	-0.351894	1.982356
H	10.912209	-1.123638	1.808417

4

SCF (wB97x) = -12680.5777333
E(SCF)+ZPE(0 K)= -12677.717906
H(298 K)= -12677.555552
G(298 K)= -12677.924919
Lowest Frequency = 4.9080cm-1

Pd	-6.349774	6.315665	9.479015
Zn	-8.356696	4.908909	9.463290
Zn	-6.924260	7.567717	7.445596
N	-7.431317	7.571463	5.511933
N	-7.153170	9.562652	7.694871
C	-7.821214	10.305853	6.827296
C	-8.276525	9.843397	5.577134
H	-8.797620	10.576711	4.971708
C	-7.021236	6.474003	4.686614
C	-7.858791	5.349364	4.543922
C	-8.258452	8.617038	3.437550
H	-8.531191	7.628600	3.064346
H	-9.050609	9.328176	3.189983
H	-7.353109	8.926843	2.900721
C	-5.770530	6.530407	4.038648
C	-6.534195	10.182139	8.828357
C	-6.225607	4.369265	3.036472
C	-8.111568	11.759100	7.144819
H	-7.212481	12.280672	7.485888
H	-8.513237	12.278758	6.272367
H	-8.846593	11.824311	7.954339
C	-5.393757	5.465638	3.218381
H	-4.430906	5.495479	2.712476
C	-7.444205	4.316028	3.701078
H	-8.088380	3.451007	3.561001
C	-7.260710	10.415176	10.011261
C	-5.158421	10.505275	8.749053
C	-4.551984	11.104416	9.854440
H	-3.498572	11.369869	9.814836
C	-6.604521	11.007409	11.093855
H	-7.156257	11.192575	12.013947
C	-5.265822	11.360992	11.020242
C	-7.981716	8.630997	4.929339
H	-5.479911	6.909715	8.168794
H	-7.357382	5.680179	10.666936
N	-8.015974	2.919481	9.281851
N	-10.338240	4.770390	9.580064
C	-10.976642	3.632689	9.305732
C	-10.356987	2.422754	8.958723
H	-11.030940	1.606767	8.721298
C	-8.993043	2.063477	9.049425
C	-12.490573	3.602672	9.382366
H	-12.875282	2.610348	9.139411
H	-12.834873	3.876758	10.385083
H	-12.929020	4.328393	8.689589
C	-8.705976	0.578982	8.939387
H	-8.788225	0.112328	9.928407
H	-9.439524	0.099346	8.285720
H	-7.701023	0.377839	8.562561
C	-6.707899	2.442465	9.620018
C	-6.461634	1.957269	10.921080
C	-5.662080	2.532065	8.677851
C	-5.171598	1.531704	11.245679
C	-4.392210	2.085001	9.046953
C	-4.141755	1.583126	10.317717
H	-4.970690	1.154100	12.246342
H	-3.580961	2.132173	8.325104
C	-11.087720	5.854731	10.149114
C	-11.724640	6.806077	9.327254
C	-11.161176	5.967281	11.554421
C	-12.463750	7.827558	9.928456
C	-11.939019	6.985660	12.107952
C	-12.594194	7.911017	11.307492
H	-12.957974	8.566252	9.300622
H	-12.021781	7.063725	13.190105
N	-4.585422	6.547147	11.045769
C	-3.520467	7.358787	10.454173
H	-3.198631	6.897179	9.514510
H	-3.913169	8.356685	10.232761
H	-2.648473	7.449975	11.118608
C	-4.089163	5.206965	11.366576
H	-4.885286	4.621604	11.837311
H	-3.806490	4.696715	10.439682
H	-3.211152	5.229427	12.033354
C	-5.196460	7.221662	12.205095
H	-5.681415	8.128251	11.822621
H	-5.992498	6.561807	12.566828
C	-4.347987	10.209951	7.488628
H	-4.719922	9.260281	7.083201
C	-8.716877	10.004249	10.170536
H	-9.081173	9.655104	9.198289
C	-9.210078	5.264001	5.242551
H	-9.186986	5.967078	6.082994
C	-4.808451	7.695289	4.235589
H	-5.341769	8.496559	4.759304
C	-11.621314	6.777235	7.809278
H	-11.120349	5.845236	7.523454
C	-10.411917	5.030561	12.494451
H	-9.774515	4.377389	11.888256
C	-5.903594	3.056599	7.270558
H	-6.741252	3.760926	7.324200
C	-7.535401	1.924759	12.001843
H	-8.502420	2.152889	11.540733
C	-4.239559	7.592089	13.353113
H	-3.549745	8.380665	13.030664

H	-3.622561	6.732445	13.643205	C	-5.697224	10.558419	21.493037
N	-4.905562	8.078202	14.576657	H	-6.541153	9.864988	21.580280
C	-5.737335	9.256970	14.323850	H	-5.429095	10.902091	22.494295
H	-6.630055	9.017743	13.721145	H	-6.047480	11.410633	20.904204
H	-6.070304	9.673107	15.280068	C	-5.859161	9.663043	18.867289
H	-5.148113	10.013964	13.795941	C	-6.755367	8.573683	18.920929
C	-5.706457	7.041510	15.229773	C	-6.177150	10.817686	18.125806
H	-5.078406	6.169070	15.438004	C	-7.956942	8.658332	18.215421
H	-6.082780	7.432892	16.179456	C	-7.398011	10.860112	17.448010
H	-6.572441	6.725639	14.623210	C	-8.283839	9.791813	17.482248
Pd	-3.168399	8.812616	16.075843	H	-8.654106	7.822924	18.247306
Zn	-3.057798	8.693652	18.546421	H	-7.656851	11.750838	16.878398
Zn	-1.121196	10.156036	15.795812	C	-0.997311	7.222390	20.138295
N	-0.182163	11.867802	16.228223	C	0.364135	7.577358	20.078515
N	0.455159	9.304984	14.850537	C	-1.387337	5.863369	20.141208
C	1.676342	9.802542	14.938391	C	1.321423	6.559578	20.084457
C	1.996982	11.009578	15.594789	C	-0.392943	4.884700	20.159299
H	3.050302	11.267540	15.593962	C	0.954485	5.223748	20.142513
C	-0.955528	13.032583	16.551753	H	2.376730	6.822386	20.044357
C	-1.151059	13.421944	17.892415	H	-0.673379	3.835243	20.176393
C	1.786315	13.325627	16.434429	C	0.221341	9.914592	11.996117
H	1.637964	13.563328	17.492184	H	0.602840	10.576758	12.780662
H	2.858247	13.299441	16.229755	C	0.096192	6.610344	15.914135
H	1.339706	14.144944	15.861553	H	-0.165560	7.523643	16.458843
C	-1.533557	13.777695	15.502048	C	-0.571430	12.643300	19.065022
C	0.210212	8.228480	13.938200	H	0.068625	11.852139	18.660206
C	-2.447918	15.328779	17.129598	C	-1.398478	13.372051	14.039618
C	2.835499	9.121236	14.236948	H	-0.870666	12.412699	13.998610
H	2.907554	9.480540	13.203239	C	0.834140	9.022612	19.985768
H	3.776100	9.362433	14.738892	H	-0.035756	9.670838	20.135005
H	2.715728	8.036506	14.197510	C	-2.858422	5.457615	20.131857
C	-2.257914	14.929485	15.813751	H	-3.404529	6.224781	19.569572
H	-2.690736	15.520476	15.008964	C	-5.241444	12.014204	18.034963
C	-1.907645	14.566087	18.155802	H	-4.342300	11.783199	18.615038
H	-2.064555	14.872949	19.188222	C	-6.464146	7.315843	19.732482
C	0.011869	6.922604	14.428018	H	-5.516006	7.460886	20.261700
C	0.132366	8.499400	12.555477	H	-3.019083	16.226202	17.354486
C	-0.083393	7.436511	11.677740	H	-0.410819	5.322455	11.443723
H	-0.134232	7.628429	10.607693	H	-9.227512	9.843987	16.944769
C	-0.211490	5.893510	13.509901	H	1.714223	4.446214	20.158370
H	-0.350291	4.877779	13.871387	H	-13.191440	8.700984	11.756207
C	-0.246842	6.138721	12.143880	H	-3.145643	1.237616	10.583931
C	1.134079	12.003771	16.080179	H	-5.921959	3.553155	2.385445
H	-2.660891	10.001235	14.991422	H	-4.774123	11.832168	11.868936
H	-3.503682	7.646288	17.240893	C	1.171031	10.026624	10.797487
N	-4.600240	9.549382	19.542483	H	2.162818	9.619385	11.025185
N	-2.022217	8.225483	20.195601	H	0.784640	9.490848	9.922338
C	-2.329410	8.736833	21.386176	H	1.291962	11.077111	10.508767
C	-3.423939	9.576711	21.648146	C	-1.177808	10.418530	11.615668
H	-3.497437	9.939906	22.666791	H	-1.615040	9.796173	10.823723
C	-4.524447	9.875356	20.821035	H	-1.858660	10.395076	12.475887
C	-1.465425	8.402989	22.587367	H	-1.127706	11.450896	11.243783
H	-1.921555	8.772763	23.507717	C	1.525870	6.219115	16.310876
H	-1.310789	7.323881	22.679838	H	2.234134	7.034460	16.124179
H	-0.475808	8.859835	22.485464	H	1.570236	5.971730	17.377790

H	1.859896	5.342723	15.740091
C	-0.895400	5.533056	16.359862
H	-0.880494	5.458833	17.452825
H	-1.916974	5.785079	16.050517
H	-0.639586	4.542961	15.960912
C	0.283989	13.520565	19.989764
H	-0.328035	14.263758	20.514739
H	0.771258	12.900226	20.752033
H	1.064366	14.063104	19.444949
C	-1.685270	11.972667	19.874965
H	-2.239136	11.249629	19.265569
H	-1.278712	11.441629	20.743330
H	-2.405685	12.712347	20.247948
C	-0.565484	14.386379	13.246090
H	-0.451304	14.058526	12.205560
H	-1.043939	15.373425	13.235946
H	0.436613	14.503880	13.674527
C	-2.775635	13.170698	13.394343
H	-3.369067	14.092740	13.412467
H	-2.665202	12.868607	12.345931
H	-3.342517	12.392069	13.919891
C	1.881975	9.379103	21.049314
H	2.091913	10.455388	21.023714
H	1.554134	9.120670	22.062183
H	2.829687	8.858257	20.867816
C	1.398367	9.314656	18.590465
H	1.762912	10.346123	18.519641
H	2.239301	8.648694	18.357879
H	0.639839	9.171195	17.812749
C	-3.106344	4.124066	19.419234
H	-2.719336	3.273238	19.992849
H	-4.182918	3.964586	19.294903
H	-2.644944	4.106977	18.425380
C	-3.454578	5.417741	21.545764
H	-2.908095	4.704200	22.175622
H	-3.424436	6.398758	22.031075
H	-4.504368	5.099167	21.507638
C	-5.864878	13.286223	18.624554
H	-6.168951	13.154163	19.669335
H	-5.139989	14.108294	18.582373
H	-6.752155	13.594065	18.057450
C	-4.803987	12.266452	16.588240
H	-4.097031	13.102211	16.545884
H	-4.311910	11.381677	16.169328
H	-5.663246	12.518771	15.951393
C	-7.546461	7.058784	20.789695
H	-8.514578	6.833665	20.326350
H	-7.270633	6.201527	21.415446
H	-7.686578	7.924947	21.446118
C	-6.303266	6.086954	18.829298
H	-7.209581	5.907652	18.237019
H	-5.464004	6.209708	18.134751
H	-6.119688	5.190556	19.434915
C	-4.543798	11.280699	6.405311
H	-5.580967	11.337690	6.062581
H	-4.248795	12.268609	6.781373
H	-3.920354	11.052577	5.531125
C	-2.849806	10.029488	7.754861
H	-2.351029	9.687981	6.840814
H	-2.368911	10.969967	8.050771
H	-2.660302	9.287050	8.537963
C	-8.835464	8.833092	11.152052
H	-9.875851	8.498438	11.227846
H	-8.223914	7.983731	10.826898
H	-8.502230	9.131637	12.156390
C	-9.617994	11.159784	10.624242
H	-9.364081	11.489973	11.638811
H	-9.541178	12.032528	9.965543
H	-10.663942	10.830350	10.638470
C	-4.302619	8.273680	2.908614
H	-3.700193	9.170964	3.093304
H	-3.669522	7.559219	2.369226
H	-5.130725	8.550506	2.246083
C	-3.629259	7.269190	5.121179
H	-3.971760	6.926154	6.104991
H	-3.068670	6.448159	4.656610
H	-2.937454	8.107674	5.270925
C	-10.351810	5.688388	4.308232
H	-10.365648	5.063943	3.405903
H	-11.319643	5.571746	4.811430
H	-10.261907	6.733587	3.994114
C	-9.501462	3.872919	5.812889
H	-8.701769	3.530650	6.477500
H	-10.429154	3.889241	6.396426
H	-9.630890	3.122814	5.022927
C	-11.371086	4.129657	13.281934
H	-12.040967	4.721701	13.917766
H	-11.992414	3.524700	12.611274
H	-10.810241	3.445443	13.930588
C	-9.497676	5.822109	13.440209
H	-10.070312	6.506798	14.077044
H	-8.944726	5.142977	14.099353
H	-8.771369	6.417299	12.873123
C	-10.761363	7.945441	7.311141
H	-10.666953	7.932305	6.219079
H	-11.203809	8.908758	7.596570
H	-9.752102	7.906234	7.738990
C	-12.996287	6.805167	7.128519
H	-12.885338	6.707865	6.042205
H	-13.644298	5.993306	7.477839
H	-13.519092	7.749887	7.318418
C	-4.713890	3.833544	6.701022
H	-5.007276	4.318480	5.762976
H	-4.384683	4.613459	7.397766
H	-3.861541	3.179034	6.477556
C	-6.297671	1.915144	6.323663
H	-5.509782	1.151413	6.294122
H	-7.228135	1.428303	6.637598
H	-6.442807	2.297214	5.306702
C	-7.655446	0.549845	12.671143

H	-6.757227	0.302619	13.249164
H	-8.504698	0.538115	13.364235
H	-7.804227	-0.248236	11.935018
C	-7.265623	3.008633	13.053750
H	-8.048863	3.003430	13.822580
H	-6.303202	2.838771	13.553568
H	-7.238946	4.005040	12.595889

5

SCF (wB97x) = -8713.99975331
E(SCF)+ZPE(0 K)= -8712.027585
H(298 K)= -8711.916581
G(298 K)= -8712.173729
Lowest Frequency = 9.1759cm-1

Pd	11.737350	3.778833	6.636813
H	12.233094	3.345253	5.062417
H	12.552957	3.245777	8.043947
H	10.373192	4.717221	6.983296
N	8.912150	4.140103	3.821969
C	7.771531	4.731769	3.489893
C	7.419612	6.046872	3.848863
H	6.436267	6.372908	3.529396
C	8.257988	7.053526	4.365766
N	9.463659	6.836362	4.872498
C	6.772134	3.984163	2.629701
H	7.150037	3.875555	1.606906
H	5.819948	4.517563	2.589638
H	6.601437	2.973535	3.013913
C	7.731770	8.472514	4.282577
H	7.842206	8.996364	5.237087
H	6.679356	8.480785	3.991285
H	8.299573	9.044506	3.539705
C	9.246261	2.911352	3.170428
C	9.825604	2.954073	1.891291
C	10.072691	1.755288	1.225107
H	10.503776	1.788955	0.224412
C	9.774161	0.519742	1.799870
C	9.269341	0.506526	3.098274
H	9.056649	-0.447932	3.579388
C	9.004576	1.684239	3.800656
C	10.299800	7.962166	5.154395
C	10.337133	8.522726	6.439556
C	11.174161	9.613794	6.674230
H	11.197980	10.050545	7.672596
C	11.974822	10.157744	5.671787
C	11.916017	9.583562	4.402938
H	12.527758	9.996906	3.600753
C	11.098253	8.488148	4.126801
N	15.308554	3.757907	4.842266
C	16.565680	3.402532	5.045497
C	16.952767	2.213347	5.694158
H	18.020811	2.077954	5.818248
C	16.160815	1.100369	6.024209
N	14.829739	1.068840	5.988607
C	17.684516	4.275959	4.517770
H	17.658014	4.308224	3.422808
H	18.659430	3.897473	4.831639
H	17.572502	5.307150	4.867182
C	16.925149	-0.153430	6.403420
H	16.676420	-0.470314	7.419604
H	18.001954	0.015918	6.344005
H	16.663411	-0.984576	5.740685
C	15.030794	4.863817	3.974213
C	14.839212	4.617029	2.599647
C	14.549504	5.692853	1.758851
H	14.402800	5.514106	0.695147
C	14.451350	6.985916	2.254821
H	14.232882	7.814412	1.584666
C	14.626376	7.212257	3.613003
H	14.528318	8.222576	4.004716
C	14.913265	6.166490	4.492627
C	14.928746	3.217995	2.005931
H	15.228512	2.528485	2.802465
C	13.560554	2.761244	1.485655
H	12.810108	2.743375	2.285778
H	13.625964	1.753269	1.057647
H	13.192459	3.433381	0.699691
C	15.989057	3.127825	0.901565
H	15.729486	3.755926	0.040830
H	16.079447	2.095152	0.543104
H	16.973518	3.447224	1.262648
C	15.051497	6.464554	5.976716
H	15.371892	5.547774	6.484785
C	16.098708	7.547955	6.262998
H	17.082792	7.289062	5.853435
H	16.203752	7.685935	7.345647
H	15.803996	8.513733	5.833377
C	13.693214	6.870883	6.557160
H	13.316886	7.783659	6.079686
H	13.781754	7.052922	7.632303
H	12.947107	6.078779	6.407462
C	14.189957	-0.207692	6.138082
C	13.922529	-0.741407	7.412102
C	13.380895	-2.026337	7.499521
H	13.181178	-2.453485	8.480311
C	13.087467	-2.763253	6.363600
H	12.675318	-3.765581	6.450495
C	13.297759	-2.200632	5.110727
H	13.030701	-2.770544	4.225561
C	14.132287	0.048260	8.696099
H	14.594757	1.007717	8.435324
C	15.036126	-0.661330	9.712328
H	14.586310	-1.599366	10.059286
H	15.184441	-0.022717	10.591662
H	16.022902	-0.900448	9.301903
C	12.766508	0.336120	9.330256
H	12.118646	0.871094	8.628308
H	12.874694	0.944312	10.237453

H	12.263894	-0.596967	9.614386
N	12.171542	5.013076	10.607611
C	11.592147	5.104020	11.799521
C	10.294741	4.654374	12.096421
H	9.958990	4.842241	13.109563
C	9.364076	4.007224	11.264482
N	9.595649	3.636210	10.011199
C	12.357368	5.730857	12.949011
H	13.313238	5.216705	13.096957
H	11.782448	5.676170	13.875447
H	12.593446	6.780159	12.744930
C	8.001878	3.740781	11.874532
H	7.227272	4.276473	11.314403
H	7.968675	4.069044	12.915174
H	7.744599	2.678069	11.830850
C	13.468033	5.598679	10.446702
C	14.618994	4.799484	10.543267
C	15.868257	5.396163	10.386947
H	16.759779	4.774406	10.466548
C	16.008230	6.762747	10.142890
C	14.851028	7.530100	10.042176
H	14.934194	8.597221	9.837255
C	13.579296	6.969147	10.176028
C	8.560359	2.923987	9.319680
C	7.607300	3.602288	8.545957
C	6.611842	2.862686	7.903962
H	5.866481	3.397596	7.314037
C	6.542131	1.476328	7.995285
C	7.502614	0.824792	8.768003
H	7.463313	-0.259743	8.866812
C	8.505109	1.523302	9.437067
Zn	10.086118	4.950622	5.209944
Zn	11.319719	4.044061	9.075675
Zn	13.785413	2.740316	5.674154
C	13.835248	-0.920884	4.971225
C	14.020573	-0.314613	3.583402
H	13.834239	0.763632	3.674558
C	13.010961	-0.855638	2.568911
H	13.220731	-1.897511	2.296527
H	11.989178	-0.793689	2.956611
H	13.055069	-0.265256	1.647103
C	15.447173	-0.489756	3.044656
H	15.518525	-0.086075	2.026193
H	16.189136	0.031820	3.657402
H	15.715837	-1.553244	3.004431
C	11.080911	7.871486	2.751404
H	11.518597	6.865616	2.772782
H	10.064233	7.772082	2.353528
H	11.667608	8.472238	2.049344
C	9.483901	7.961199	7.546252
H	8.423271	7.926310	7.268647
H	9.781667	6.932786	7.780128
H	9.577894	8.562120	8.456774
C	12.910622	11.304980	5.960773
H	13.052537	11.937964	5.078503
H	12.533300	11.934408	6.773462
H	13.899129	10.935468	6.263066
C	10.184342	4.272528	1.252133
H	9.330891	4.958849	1.199260
H	10.965995	4.782987	1.829811
H	10.563086	4.119789	0.236297
C	8.443902	1.641111	5.196274
H	9.129670	2.107048	5.916703
H	7.495858	2.187218	5.269193
H	8.270345	0.609436	5.519735
C	9.959240	-0.757866	1.018762
H	10.873854	-0.731162	0.416288
H	10.016863	-1.628125	1.680763
H	9.118841	-0.919865	0.332442
C	14.507231	3.321533	10.811705
H	13.885704	3.111033	11.690470
H	14.043987	2.812372	9.958779
H	15.495294	2.878508	10.974283
C	12.347170	7.817159	9.988008
H	11.759390	7.453181	9.135896
H	11.685757	7.798361	10.862349
H	12.619898	8.858447	9.787472
C	17.376690	7.378843	9.986114
H	17.311259	8.462580	9.845092
H	18.000394	7.191547	10.867808
H	17.902234	6.960500	9.119317
C	7.594647	5.104743	8.411330
H	8.355484	5.577523	9.036938
H	7.781311	5.404051	7.372150
H	6.615865	5.510047	8.695857
C	5.477591	0.701482	7.260024
H	5.259155	-0.248962	7.757878
H	4.544976	1.271413	7.190982
H	5.798213	0.471232	6.235795
C	9.470517	0.788973	10.331273
H	9.201637	0.908320	11.389600
H	9.472983	-0.282509	10.107912
H	10.489055	1.168309	10.221810

5-theta30

SCF (wB97x) = -8713.89760241

Pd	11.737350	3.778833	6.636813
H	12.609858	3.208565	5.285517
H	12.191812	3.479760	8.259591
H	10.353925	4.740282	6.484167
N	8.912150	4.140103	3.821969
C	7.771531	4.731769	3.489893
C	7.419612	6.046872	3.848863
H	6.436267	6.372908	3.529396
C	8.257988	7.053526	4.365766
N	9.463659	6.836362	4.872498
C	6.772134	3.984163	2.629701
H	7.150037	3.875555	1.606906

H	5.819948	4.517563	2.589638
H	6.601437	2.973535	3.013913
C	7.731770	8.472514	4.282577
H	7.842206	8.996364	5.237087
H	6.679356	8.480785	3.991285
H	8.299573	9.044506	3.539705
C	9.246261	2.911352	3.170428
C	9.825604	2.954073	1.891291
C	10.072691	1.755288	1.225107
H	10.503776	1.788955	0.224412
C	9.774161	0.519742	1.799870
C	9.269341	0.506526	3.098274
H	9.056649	-0.447932	3.579388
C	9.004576	1.684239	3.800656
C	10.299800	7.962166	5.154395
C	10.337133	8.522726	6.439556
C	11.174161	9.613794	6.674230
H	11.197980	10.050545	7.672596
C	11.974822	10.157744	5.671787
C	11.916017	9.583562	4.402938
H	12.527758	9.996906	3.600753
C	11.098253	8.488148	4.126801
N	15.308554	3.757907	4.842266
C	16.565680	3.402532	5.045497
C	16.952767	2.213347	5.694158
H	18.020811	2.077954	5.818248
C	16.160815	1.100369	6.024209
N	14.829739	1.068840	5.988607
C	17.684516	4.275959	4.517770
H	17.658014	4.308224	3.422808
H	18.659430	3.897473	4.831639
H	17.572502	5.307150	4.867182
C	16.925149	-0.153430	6.403420
H	16.676420	-0.470314	7.419604
H	18.001954	0.015918	6.344005
H	16.663411	-0.984576	5.740685
C	15.030794	4.863817	3.974213
C	14.839212	4.617029	2.599647
C	14.549504	5.692853	1.758851
H	14.402800	5.514106	0.695147
C	14.451350	6.985916	2.254821
H	14.232882	7.814412	1.584666
C	14.626376	7.212257	3.613003
H	14.528318	8.222576	4.004716
C	14.913265	6.166490	4.492627
C	14.928746	3.217995	2.005931
H	15.228512	2.528485	2.802465
C	13.560554	2.761244	1.485655
H	12.810108	2.743375	2.285778
H	13.625964	1.753269	1.057647
H	13.192459	3.433381	0.699691
C	15.989057	3.127825	0.901565
H	15.729486	3.755926	0.040830
H	16.079447	2.095152	0.543104
H	16.973518	3.447224	1.262648
C	15.051497	6.464554	5.976716
H	15.371892	5.547774	6.484785
C	16.098708	7.547955	6.262998
H	17.082792	7.289062	5.853435
H	16.203752	7.685935	7.345647
H	15.803996	8.513733	5.833377
C	13.693214	6.870883	6.557160
H	13.316886	7.783659	6.079686
H	13.781754	7.052922	7.632303
H	12.947107	6.078779	6.407462
C	14.189957	-0.207692	6.138082
C	13.922529	-0.741407	7.412102
C	13.380895	-2.026337	7.499521
H	13.181178	-2.453485	8.480311
C	13.087467	-2.763253	6.363600
H	12.675318	-3.765581	6.450495
C	13.297759	-2.200632	5.110727
H	13.030701	-2.770544	4.225561
C	14.132287	0.048260	8.696099
H	14.594757	1.007717	8.435324
C	15.036126	-0.661330	9.712328
H	14.586310	-1.599366	10.059286
H	15.184441	-0.022717	10.591662
H	16.022902	-0.900448	9.301903
C	12.766508	0.336120	9.330256
H	12.118646	0.871094	8.628308
H	12.874694	0.944312	10.237453
H	12.263894	-0.596967	9.614386
N	12.171542	5.013076	10.607611
C	11.592147	5.104020	11.799521
C	10.294741	4.654374	12.096421
H	9.958990	4.842241	13.109563
C	9.364076	4.007224	11.264482
N	9.595649	3.636210	10.011199
C	12.357368	5.730857	12.949011
H	13.313238	5.216705	13.096957
H	11.782448	5.676170	13.875447
H	12.593446	6.780159	12.744930
C	8.001878	3.740781	11.874532
H	7.227272	4.276473	11.314403
H	7.968675	4.069044	12.915174
H	7.744599	2.678069	11.830850
C	13.468033	5.598679	10.446702
C	14.618994	4.799484	10.543267
C	15.868257	5.396163	10.386947
H	16.759779	4.774406	10.466548
C	16.008230	6.762747	10.142890
C	14.851028	7.530100	10.042176
H	14.934194	8.597221	9.837255
C	13.579296	6.969147	10.176028
C	8.560359	2.923987	9.319680
C	7.607300	3.602288	8.545957
C	6.611842	2.862686	7.903962
H	5.866481	3.397596	7.314037
C	6.542131	1.476328	7.995285

C	7.502614	0.824792	8.768003
H	7.463313	-0.259743	8.866812
C	8.505109	1.523302	9.437067
Zn	10.086118	4.950622	5.209944
Zn	11.319719	4.044061	9.075675
Zn	13.785413	2.740316	5.674154
C	13.835248	-0.920884	4.971225
C	14.020573	-0.314613	3.583402
H	13.834239	0.763632	3.674558
C	13.010961	-0.855638	2.568911
H	13.220731	-1.897511	2.296527
H	11.989178	-0.793689	2.956611
H	13.055069	-0.265256	1.647103
C	15.447173	-0.489756	3.044656
H	15.518525	-0.086075	2.026193
H	16.189136	0.031820	3.657402
H	15.715837	-1.553244	3.004431
C	11.080911	7.871486	2.751404
H	11.518597	6.865616	2.772782
H	10.064233	7.772082	2.353528
H	11.667608	8.472238	2.049344
C	9.483901	7.961199	7.546252
H	8.423271	7.926310	7.268647
H	9.781667	6.932786	7.780128
H	9.577894	8.562120	8.456774
C	12.910622	11.304980	5.960773
H	13.052537	11.937964	5.078503
H	12.533300	11.934408	6.773462
H	13.899129	10.935468	6.263066
C	10.184342	4.272528	1.252133
H	9.330891	4.958849	1.199260
H	10.965995	4.782987	1.829811
H	10.563086	4.119789	0.236297
C	8.443902	1.641111	5.196274
H	9.129670	2.107048	5.916703
H	7.495858	2.187218	5.269193
H	8.270345	0.609436	5.519735
C	9.959240	-0.757866	1.018762
H	10.873854	-0.731162	0.416288
H	10.016863	-1.628125	1.680763
H	9.118841	-0.919865	0.332442
C	14.507231	3.321533	10.811705
H	13.885704	3.111033	11.690470
H	14.043987	2.812372	9.958779
H	15.495294	2.878508	10.974283
C	12.347170	7.817159	9.988008
H	11.759390	7.453181	9.135896
H	11.685757	7.798361	10.862349
H	12.619898	8.858447	9.787472
C	17.376690	7.378843	9.986114
H	17.311259	8.462580	9.845092
H	18.000394	7.191547	10.867808
H	17.902234	6.960500	9.119317
C	7.594647	5.104743	8.411330
H	8.355484	5.577523	9.036938
H	7.781311	5.404051	7.372150
H	6.615865	5.510047	8.695857
C	5.477591	0.701482	7.260024
H	5.259155	-0.248962	7.757878
H	4.544976	1.271413	7.190982
H	5.798213	0.471232	6.235795
C	9.470517	0.788973	10.331273
H	9.201637	0.908320	11.389600
H	9.472983	-0.282509	10.107912
H	10.489055	1.168309	10.221810

5-theta35

SCF (wB97x) = -8713.96300869

Pd	11.737350	3.778833	6.636813
H	12.493563	3.248723	5.201682
H	12.308756	3.403954	8.206001
H	10.346915	4.742271	6.631561
N	8.912150	4.140103	3.821969
C	7.771531	4.731769	3.489893
C	7.419612	6.046872	3.848863
H	6.436267	6.372908	3.529396
C	8.257988	7.053526	4.365766
N	9.463659	6.836362	4.872498
C	6.772134	3.984163	2.629701
H	7.150037	3.875555	1.606906
H	5.819948	4.517563	2.589638
H	6.601437	2.973535	3.013913
C	7.731770	8.472514	4.282577
H	7.842206	8.996364	5.237087
H	6.679356	8.480785	3.991285
H	8.299573	9.044506	3.539705
C	9.246261	2.911352	3.170428
C	9.825604	2.954073	1.891291
C	10.072691	1.755288	1.225107
H	10.503776	1.788955	0.224412
C	9.774161	0.519742	1.799870
C	9.269341	0.506526	3.098274
H	9.056649	-0.447932	3.579388
C	9.004576	1.684239	3.800656
C	10.299800	7.962166	5.154395
C	10.337133	8.522726	6.439556
C	11.174161	9.613794	6.674230
H	11.197980	10.050545	7.672596
C	11.974822	10.157744	5.671787
C	11.916017	9.583562	4.402938
H	12.527758	9.996906	3.600753
C	11.098253	8.488148	4.126801
N	15.308554	3.757907	4.842266
C	16.565680	3.402532	5.045497
C	16.952767	2.213347	5.694158
H	18.020811	2.077954	5.818248
C	16.160815	1.100369	6.024209
N	14.829739	1.068840	5.988607

C	17.684516	4.275959	4.517770
H	17.658014	4.308224	3.422808
H	18.659430	3.897473	4.831639
H	17.572502	5.307150	4.867182
C	16.925149	-0.153430	6.403420
H	16.676420	-0.470314	7.419604
H	18.001954	0.015918	6.344005
H	16.663411	-0.984576	5.740685
C	15.030794	4.863817	3.974213
C	14.839212	4.617029	2.599647
C	14.549504	5.692853	1.758851
H	14.402800	5.514106	0.695147
C	14.451350	6.985916	2.254821
H	14.232882	7.814412	1.584666
C	14.626376	7.212257	3.613003
H	14.528318	8.222576	4.004716
C	14.913265	6.166490	4.492627
C	14.928746	3.217995	2.005931
H	15.228512	2.528485	2.802465
C	13.560554	2.761244	1.485655
H	12.810108	2.743375	2.285778
H	13.625964	1.753269	1.057647
H	13.192459	3.433381	0.699691
C	15.989057	3.127825	0.901565
H	15.729486	3.755926	0.040830
H	16.079447	2.095152	0.543104
H	16.973518	3.447224	1.262648
C	15.051497	6.464554	5.976716
H	15.371892	5.547774	6.484785
C	16.098708	7.547955	6.262998
H	17.082792	7.289062	5.853435
H	16.203752	7.685935	7.345647
H	15.803996	8.513733	5.833377
C	13.693214	6.870883	6.557160
H	13.316886	7.783659	6.079686
H	13.781754	7.052922	7.632303
H	12.947107	6.078779	6.407462
C	14.189957	-0.207692	6.138082
C	13.922529	-0.741407	7.412102
C	13.380895	-2.026337	7.499521
H	13.181178	-2.453485	8.480311
C	13.087467	-2.763253	6.363600
H	12.675318	-3.765581	6.450495
C	13.297759	-2.200632	5.110727
H	13.030701	-2.770544	4.225561
C	14.132287	0.048260	8.696099
H	14.594757	1.007717	8.435324
C	15.036126	-0.661330	9.712328
H	14.586310	-1.599366	10.059286
H	15.184441	-0.022717	10.591662
H	16.022902	-0.900448	9.301903
C	12.766508	0.336120	9.330256
H	12.118646	0.871094	8.628308
H	12.874694	0.944312	10.237453
H	12.263894	-0.596967	9.614386
N	12.171542	5.013076	10.607611
C	11.592147	5.104020	11.799521
C	10.294741	4.654374	12.096421
H	9.958990	4.842241	13.109563
C	9.364076	4.007224	11.264482
N	9.595649	3.636210	10.011199
C	12.357368	5.730857	12.949011
H	13.313238	5.216705	13.096957
H	11.782448	5.676170	13.875447
H	12.593446	6.780159	12.744930
C	8.001878	3.740781	11.874532
H	7.227272	4.276473	11.314403
H	7.968675	4.069044	12.915174
H	7.744599	2.678069	11.830850
C	13.468033	5.598679	10.446702
C	14.618994	4.799484	10.543267
C	15.868257	5.396163	10.386947
H	16.759779	4.774406	10.466548
C	16.008230	6.762747	10.142890
C	14.851028	7.530100	10.042176
H	14.934194	8.597221	9.837255
C	13.579296	6.969147	10.176028
C	8.560359	2.923987	9.319680
C	7.607300	3.602288	8.545957
C	6.611842	2.862686	7.903962
H	5.866481	3.397596	7.314037
C	6.542131	1.476328	7.995285
C	7.502614	0.824792	8.768003
H	7.463313	-0.259743	8.866812
C	8.505109	1.523302	9.437067
Zn	10.086118	4.950622	5.209944
Zn	11.319719	4.044061	9.075675
Zn	13.785413	2.740316	5.674154
C	13.835248	-0.920884	4.971225
C	14.020573	-0.314613	3.583402
H	13.834239	0.763632	3.674558
C	13.010961	-0.855638	2.568911
H	13.220731	-1.897511	2.296527
H	11.989178	-0.793689	2.956611
H	13.055069	-0.265256	1.647103
C	15.447173	-0.489756	3.044656
H	15.518525	-0.086075	2.026193
H	16.189136	0.031820	3.657402
H	15.715837	-1.553244	3.004431
C	11.080911	7.871486	2.751404
H	11.518597	6.865616	2.772782
H	10.064233	7.772082	2.353528
H	11.667608	8.472238	2.049344
C	9.483901	7.961199	7.546252
H	8.423271	7.926310	7.268647
H	9.781667	6.932786	7.780128
H	9.577894	8.562120	8.456774
C	12.910622	11.304980	5.960773
H	13.052537	11.937964	5.078503
H	12.533300	11.934408	6.773462

H	13.899129	10.935468	6.263066
C	10.184342	4.272528	1.252133
H	9.330891	4.958849	1.199260
H	10.965995	4.782987	1.829811
H	10.563086	4.119789	0.236297
C	8.443902	1.641111	5.196274
H	9.129670	2.107048	5.916703
H	7.495858	2.187218	5.269193
H	8.270345	0.609436	5.519735
C	9.959240	-0.757866	1.018762
H	10.873854	-0.731162	0.416288
H	10.016863	-1.628125	1.680763
H	9.118841	-0.919865	0.332442
C	14.507231	3.321533	10.811705
H	13.885704	3.111033	11.690470
H	14.043987	2.812372	9.958779
H	15.495294	2.878508	10.974283
C	12.347170	7.817159	9.988008
H	11.759390	7.453181	9.135896
H	11.685757	7.798361	10.862349
H	12.619898	8.858447	9.787472
C	17.376690	7.378843	9.986114
H	17.311259	8.462580	9.845092
H	18.000394	7.191547	10.867808
H	17.902234	6.960500	9.119317
C	7.594647	5.104743	8.411330
H	8.355484	5.577523	9.036938
H	7.781311	5.404051	7.372150
H	6.615865	5.510047	8.695857
C	5.477591	0.701482	7.260024
H	5.259155	-0.248962	7.757878
H	4.544976	1.271413	7.190982
H	5.798213	0.471232	6.235795
C	9.470517	0.788973	10.331273
H	9.201637	0.908320	11.389600
H	9.472983	-0.282509	10.107912
H	10.489055	1.168309	10.221810

5-theta40

SCF (wB97x) = -8713.99108736

Pd	11.737350	3.778833	6.636813
H	12.371512	3.292915	5.128768
H	12.421351	3.331000	8.140468
H	10.350487	4.736927	6.778996
N	8.912150	4.140103	3.821969
C	7.771531	4.731769	3.489893
C	7.419612	6.046872	3.848863
H	6.436267	6.372908	3.529396
C	8.257988	7.053526	4.365766
N	9.463659	6.836362	4.872498
C	6.772134	3.984163	2.629701
H	7.150037	3.875555	1.606906
H	5.819948	4.517563	2.589638
H	6.601437	2.973535	3.013913
C	7.731770	8.472514	4.282577
H	7.842206	8.996364	5.237087
H	6.679356	8.480785	3.991285
H	8.299573	9.044506	3.539705
C	9.246261	2.911352	3.170428
C	9.825604	2.954073	1.891291
C	10.072691	1.755288	1.225107
H	10.503776	1.788955	0.224412
C	9.774161	0.519742	1.799870
C	9.269341	0.506526	3.098274
H	9.056649	-0.447932	3.579388
C	9.004576	1.684239	3.800656
C	10.299800	7.962166	5.154395
C	10.337133	8.522726	6.439556
C	11.174161	9.613794	6.674230
H	11.197980	10.050545	7.672596
C	11.974822	10.157744	5.671787
C	11.916017	9.583562	4.402938
H	12.527758	9.996906	3.600753
C	11.098253	8.488148	4.126801
N	15.308554	3.757907	4.842266
C	16.565680	3.402532	5.045497
C	16.952767	2.213347	5.694158
H	18.020811	2.077954	5.818248
C	16.160815	1.100369	6.024209
N	14.829739	1.068840	5.988607
C	17.684516	4.275959	4.517770
H	17.658014	4.308224	3.422808
H	18.659430	3.897473	4.831639
H	17.572502	5.307150	4.867182
C	16.925149	-0.153430	6.403420
H	16.676420	-0.470314	7.419604
H	18.001954	0.015918	6.344005
H	16.663411	-0.984576	5.740685
C	15.030794	4.863817	3.974213
C	14.839212	4.617029	2.599647
C	14.549504	5.692853	1.758851
H	14.402800	5.514106	0.695147
C	14.451350	6.985916	2.254821
H	14.232882	7.814412	1.584666
C	14.626376	7.212257	3.613003
H	14.528318	8.222576	4.004716
C	14.913265	6.166490	4.492627
C	14.928746	3.217995	2.005931
H	15.228512	2.528485	2.802465
C	13.560554	2.761244	1.485655
H	12.810108	2.743375	2.285778
H	13.625964	1.753269	1.057647
H	13.192459	3.433381	0.699691
C	15.989057	3.127825	0.901565
H	15.729486	3.755926	0.040830
H	16.079447	2.095152	0.543104
H	16.973518	3.447224	1.262648
C	15.051497	6.464554	5.976716

H	15.371892	5.547774	6.484785	H	7.463313	-0.259743	8.866812
C	16.098708	7.547955	6.262998	C	8.505109	1.523302	9.437067
H	17.082792	7.289062	5.853435	Zn	10.086118	4.950622	5.209944
H	16.203752	7.685935	7.345647	Zn	11.319719	4.044061	9.075675
H	15.803996	8.513733	5.833377	Zn	13.785413	2.740316	5.674154
C	13.693214	6.870883	6.557160	C	13.835248	-0.920884	4.971225
H	13.316886	7.783659	6.079686	C	14.020573	-0.314613	3.583402
H	13.781754	7.052922	7.632303	H	13.834239	0.763632	3.674558
H	12.947107	6.078779	6.407462	C	13.010961	-0.855638	2.568911
C	14.189957	-0.207692	6.138082	H	13.220731	-1.897511	2.296527
C	13.922529	-0.741407	7.412102	H	11.989178	-0.793689	2.956611
C	13.380895	-2.026337	7.499521	H	13.055069	-0.265256	1.647103
H	13.181178	-2.453485	8.480311	C	15.447173	-0.489756	3.044656
C	13.087467	-2.763253	6.363600	H	15.518525	-0.086075	2.026193
H	12.675318	-3.765581	6.450495	H	16.189136	0.031820	3.657402
C	13.297759	-2.200632	5.110727	H	15.715837	-1.553244	3.004431
H	13.030701	-2.770544	4.225561	C	11.080911	7.871486	2.751404
C	14.132287	0.048260	8.696099	H	11.518597	6.865616	2.772782
H	14.594757	1.007717	8.435324	H	10.064233	7.772082	2.353528
C	15.036126	-0.661330	9.712328	H	11.667608	8.472238	2.049344
H	14.586310	-1.599366	10.059286	C	9.483901	7.961199	7.546252
H	15.184441	-0.022717	10.591662	H	8.423271	7.926310	7.268647
H	16.022902	-0.900448	9.301903	H	9.781667	6.932786	7.780128
C	12.766508	0.336120	9.330256	H	9.577894	8.562120	8.456774
H	12.118646	0.871094	8.628308	C	12.910622	11.304980	5.960773
H	12.874694	0.944312	10.237453	H	13.052537	11.937964	5.078503
H	12.263894	-0.596967	9.614386	H	12.533300	11.934408	6.773462
N	12.171542	5.013076	10.607611	H	13.899129	10.935468	6.263066
C	11.592147	5.104020	11.799521	C	10.184342	4.272528	1.252133
C	10.294741	4.654374	12.096421	H	9.330891	4.958849	1.199260
H	9.958990	4.842241	13.109563	H	10.965995	4.782987	1.829811
C	9.364076	4.007224	11.264482	H	10.563086	4.119789	0.236297
N	9.595649	3.636210	10.011199	C	8.443902	1.641111	5.196274
C	12.357368	5.730857	12.949011	H	9.129670	2.107048	5.916703
H	13.313238	5.216705	13.096957	H	7.495858	2.187218	5.269193
H	11.782448	5.676170	13.875447	H	8.270345	0.609436	5.519735
H	12.593446	6.780159	12.744930	C	9.959240	-0.757866	1.018762
C	8.001878	3.740781	11.874532	H	10.873854	-0.731162	0.416288
H	7.227272	4.276473	11.314403	H	10.016863	-1.628125	1.680763
H	7.968675	4.069044	12.915174	H	9.118841	-0.919865	0.332442
H	7.744599	2.678069	11.830850	C	14.507231	3.321533	10.811705
C	13.468033	5.598679	10.446702	H	13.885704	3.111033	11.690470
C	14.618994	4.799484	10.543267	H	14.043987	2.812372	9.958779
C	15.868257	5.396163	10.386947	H	15.495294	2.878508	10.974283
H	16.759779	4.774406	10.466548	C	12.347170	7.817159	9.988008
C	16.008230	6.762747	10.142890	H	11.759390	7.453181	9.135896
C	14.851028	7.530100	10.042176	H	11.685757	7.798361	10.862349
H	14.934194	8.597221	9.837255	H	12.619898	8.858447	9.787472
C	13.579296	6.969147	10.176028	C	17.376690	7.378843	9.986114
C	8.560359	2.923987	9.319680	H	17.311259	8.462580	9.845092
C	7.607300	3.602288	8.545957	H	18.000394	7.191547	10.867808
C	6.611842	2.862686	7.903962	H	17.902234	6.960500	9.119317
H	5.866481	3.397596	7.314037	C	7.594647	5.104743	8.411330
C	6.542131	1.476328	7.995285	H	8.355484	5.577523	9.036938
C	7.502614	0.824792	8.768003	H	7.781311	5.404051	7.372150

H	6.615865	5.510047	8.695857
C	5.477591	0.701482	7.260024
H	5.259155	-0.248962	7.757878
H	4.544976	1.271413	7.190982
H	5.798213	0.471232	6.235795
C	9.470517	0.788973	10.331273
H	9.201637	0.908320	11.389600
H	9.472983	-0.282509	10.107912
H	10.489055	1.168309	10.221810

5-theta45

SCF (wB97x) = -8713.99934057

Pd	11.737350	3.778833	6.636813
H	12.244635	3.340805	5.067332
H	12.528740	3.261455	8.063491
H	10.364614	4.724292	6.925347
N	8.912150	4.140103	3.821969
C	7.771531	4.731769	3.489893
C	7.419612	6.046872	3.848863
H	6.436267	6.372908	3.529396
C	8.257988	7.053526	4.365766
N	9.463659	6.836362	4.872498
C	6.772134	3.984163	2.629701
H	7.150037	3.875555	1.606906
H	5.819948	4.517563	2.589638
H	6.601437	2.973535	3.013913
C	7.731770	8.472514	4.282577
H	7.842206	8.996364	5.237087
H	6.679356	8.480785	3.991285
H	8.299573	9.044506	3.539705
C	9.246261	2.911352	3.170428
C	9.825604	2.954073	1.891291
C	10.072691	1.755288	1.225107
H	10.503776	1.788955	0.224412
C	9.774161	0.519742	1.799870
C	9.269341	0.506526	3.098274
H	9.056649	-0.447932	3.579388
C	9.004576	1.684239	3.800656
C	10.299800	7.962166	5.154395
C	10.337133	8.522726	6.439556
C	11.174161	9.613794	6.674230
H	11.197980	10.050545	7.672596
C	11.974822	10.157744	5.671787
C	11.916017	9.583562	4.402938
H	12.527758	9.996906	3.600753
C	11.098253	8.488148	4.126801
N	15.308554	3.757907	4.842266
C	16.565680	3.402532	5.045497
C	16.952767	2.213347	5.694158
H	18.020811	2.077954	5.818248
C	16.160815	1.100369	6.024209
N	14.829739	1.068840	5.988607
C	17.684516	4.275959	4.517770
H	17.658014	4.308224	3.422808
H	18.659430	3.897473	4.831639
H	17.572502	5.307150	4.867182
C	16.925149	-0.153430	6.403420
H	16.676420	-0.470314	7.419604
H	18.001954	0.015918	6.344005
H	16.663411	-0.984576	5.740685
C	15.030794	4.863817	3.974213
C	14.839212	4.617029	2.599647
C	14.549504	5.692853	1.758851
H	14.402800	5.514106	0.695147
C	14.451350	6.985916	2.254821
H	14.232882	7.814412	1.584666
C	14.626376	7.212257	3.613003
H	14.528318	8.222576	4.004716
C	14.913265	6.166490	4.492627
C	14.928746	3.217995	2.005931
H	15.228512	2.528485	2.802465
C	13.560554	2.761244	1.485655
H	12.810108	2.743375	2.285778
H	13.625964	1.753269	1.057647
H	13.192459	3.433381	0.699691
C	15.989057	3.127825	0.901565
H	15.729486	3.755926	0.040830
H	16.079447	2.095152	0.543104
H	16.973518	3.447224	1.262648
C	15.051497	6.464554	5.976716
H	15.371892	5.547774	6.484785
C	16.098708	7.547955	6.262998
H	17.082792	7.289062	5.853435
H	16.203752	7.685935	7.345647
H	15.803996	8.513733	5.833377
C	13.693214	6.870883	6.557160
H	13.316886	7.783659	6.079686
H	13.781754	7.052922	7.632303
H	12.947107	6.078779	6.407462
C	14.189957	-0.207692	6.138082
C	13.922529	-0.741407	7.412102
C	13.380895	-2.026337	7.499521
H	13.181178	-2.453485	8.480311
C	13.087467	-2.763253	6.363600
H	12.675318	-3.765581	6.450495
C	13.297759	-2.200632	5.110727
H	13.030701	-2.770544	4.225561
C	14.132287	0.048260	8.696099
H	14.594757	1.007717	8.435324
C	15.036126	-0.661330	9.712328
H	14.586310	-1.599366	10.059286
H	15.184441	-0.022717	10.591662
H	16.022902	-0.900448	9.301903
C	12.766508	0.336120	9.330256
H	12.118646	0.871094	8.628308
H	12.874694	0.944312	10.237453
H	12.263894	-0.596967	9.614386
N	12.171542	5.013076	10.607611

C	11.592147	5.104020	11.799521
C	10.294741	4.654374	12.096421
H	9.958990	4.842241	13.109563
C	9.364076	4.007224	11.264482
N	9.595649	3.636210	10.011199
C	12.357368	5.730857	12.949011
H	13.313238	5.216705	13.096957
H	11.782448	5.676170	13.875447
H	12.593446	6.780159	12.744930
C	8.001878	3.740781	11.874532
H	7.227272	4.276473	11.314403
H	7.968675	4.069044	12.915174
H	7.744599	2.678069	11.830850
C	13.468033	5.598679	10.446702
C	14.618994	4.799484	10.543267
C	15.868257	5.396163	10.386947
H	16.759779	4.774406	10.466548
C	16.008230	6.762747	10.142890
C	14.851028	7.530100	10.042176
H	14.934194	8.597221	9.837255
C	13.579296	6.969147	10.176028
C	8.560359	2.923987	9.319680
C	7.607300	3.602288	8.545957
C	6.611842	2.862686	7.903962
H	5.866481	3.397596	7.314037
C	6.542131	1.476328	7.995285
C	7.502614	0.824792	8.768003
H	7.463313	-0.259743	8.866812
C	8.505109	1.523302	9.437067
Zn	10.086118	4.950622	5.209944
Zn	11.319719	4.044061	9.075675
Zn	13.785413	2.740316	5.674154
C	13.835248	-0.920884	4.971225
C	14.020573	-0.314613	3.583402
H	13.834239	0.763632	3.674558
C	13.010961	-0.855638	2.568911
H	13.220731	-1.897511	2.296527
H	11.989178	-0.793689	2.956611
H	13.055069	-0.265256	1.647103
C	15.447173	-0.489756	3.044656
H	15.518525	-0.086075	2.026193
H	16.189136	0.031820	3.657402
H	15.715837	-1.553244	3.004431
C	11.080911	7.871486	2.751404
H	11.518597	6.865616	2.772782
H	10.064233	7.772082	2.353528
H	11.667608	8.472238	2.049344
C	9.483901	7.961199	7.546252
H	8.423271	7.926310	7.268647
H	9.781667	6.932786	7.780128
H	9.577894	8.562120	8.456774
C	12.910622	11.304980	5.960773
H	13.052537	11.937964	5.078503
H	12.533300	11.934408	6.773462
H	13.899129	10.935468	6.263066
C	10.184342	4.272528	1.252133
H	9.330891	4.958849	1.199260
H	10.965995	4.782987	1.829811
H	10.563086	4.119789	0.236297
C	8.443902	1.641111	5.196274
H	9.129670	2.107048	5.916703
H	7.495858	2.187218	5.269193
H	8.270345	0.609436	5.519735
C	9.959240	-0.757866	1.018762
H	10.873854	-0.731162	0.416288
H	10.016863	-1.628125	1.680763
H	9.118841	-0.919865	0.332442
C	14.507231	3.321533	10.811705
H	13.885704	3.111033	11.690470
H	14.043987	2.812372	9.958779
H	15.495294	2.878508	10.974283
C	12.347170	7.817159	9.988008
H	11.759390	7.453181	9.135896
H	11.685757	7.798361	10.862349
H	12.619898	8.858447	9.787472
C	17.376690	7.378843	9.986114
H	17.311259	8.462580	9.845092
H	18.000394	7.191547	10.867808
H	17.902234	6.960500	9.119317
C	7.594647	5.104743	8.411330
H	8.355484	5.577523	9.036938
H	7.781311	5.404051	7.372150
H	6.615865	5.510047	8.695857
C	5.477591	0.701482	7.260024
H	5.259155	-0.248962	7.757878
H	4.544976	1.271413	7.190982
H	5.798213	0.471232	6.235795
C	9.470517	0.788973	10.331273
H	9.201637	0.908320	11.389600
H	9.472983	-0.282509	10.107912
H	10.489055	1.168309	10.221810

5-theta50

SCF (wB97x) = -8713.99860455

Pd	11.737350	3.778833	6.636813
H	12.113897	3.392029	5.017841
H	12.630106	3.195847	7.975657
H	10.389188	4.704461	7.069504
N	8.912150	4.140103	3.821969
C	7.771531	4.731769	3.489893
C	7.419612	6.046872	3.848863
H	6.436267	6.372908	3.529396
C	8.257988	7.053526	4.365766
N	9.463659	6.836362	4.872498
C	6.772134	3.984163	2.629701
H	7.150037	3.875555	1.606906
H	5.819948	4.517563	2.589638
H	6.601437	2.973535	3.013913

C	7.731770	8.472514	4.282577	C	16.098708	7.547955	6.262998
H	7.842206	8.996364	5.237087	H	17.082792	7.289062	5.853435
H	6.679356	8.480785	3.991285	H	16.203752	7.685935	7.345647
H	8.299573	9.044506	3.539705	H	15.803996	8.513733	5.833377
C	9.246261	2.911352	3.170428	C	13.693214	6.870883	6.557160
C	9.825604	2.954073	1.891291	H	13.316886	7.783659	6.079686
C	10.072691	1.755288	1.225107	H	13.781754	7.052922	7.632303
H	10.503776	1.788955	0.224412	H	12.947107	6.078779	6.407462
C	9.774161	0.519742	1.799870	C	14.189957	-0.207692	6.138082
C	9.269341	0.506526	3.098274	C	13.922529	-0.741407	7.412102
H	9.056649	-0.447932	3.579388	C	13.380895	-2.026337	7.499521
C	9.004576	1.684239	3.800656	H	13.181178	-2.453485	8.480311
C	10.299800	7.962166	5.154395	C	13.087467	-2.763253	6.363600
C	10.337133	8.522726	6.439556	H	12.675318	-3.765581	6.450495
C	11.174161	9.613794	6.674230	C	13.297759	-2.200632	5.110727
H	11.197980	10.050545	7.672596	H	13.030701	-2.770544	4.225561
C	11.974822	10.157744	5.671787	C	14.132287	0.048260	8.696099
C	11.916017	9.583562	4.402938	H	14.594757	1.007717	8.435324
H	12.527758	9.996906	3.600753	C	15.036126	-0.661330	9.712328
C	11.098253	8.488148	4.126801	H	14.586310	-1.599366	10.059286
N	15.308554	3.757907	4.842266	H	15.184441	-0.022717	10.591662
C	16.565680	3.402532	5.045497	H	16.022902	-0.900448	9.301903
C	16.952767	2.213347	5.694158	C	12.766508	0.336120	9.330256
H	18.020811	2.077954	5.818248	H	12.118646	0.871094	8.628308
C	16.160815	1.100369	6.024209	H	12.874694	0.944312	10.237453
N	14.829739	1.068840	5.988607	H	12.263894	-0.596967	9.614386
C	17.684516	4.275959	4.517770	N	12.171542	5.013076	10.607611
H	17.658014	4.308224	3.422808	C	11.592147	5.104020	11.799521
H	18.659430	3.897473	4.831639	C	10.294741	4.654374	12.096421
H	17.572502	5.307150	4.867182	H	9.958990	4.842241	13.109563
C	16.925149	-0.153430	6.403420	C	9.364076	4.007224	11.264482
H	16.676420	-0.470314	7.419604	N	9.595649	3.636210	10.011199
H	18.001954	0.015918	6.344005	C	12.357368	5.730857	12.949011
H	16.663411	-0.984576	5.740685	H	13.313238	5.216705	13.096957
C	15.030794	4.863817	3.974213	H	11.782448	5.676170	13.875447
C	14.839212	4.617029	2.599647	H	12.593446	6.780159	12.744930
C	14.549504	5.692853	1.758851	C	8.001878	3.740781	11.874532
H	14.402800	5.514106	0.695147	H	7.227272	4.276473	11.314403
C	14.451350	6.985916	2.254821	H	7.968675	4.069044	12.915174
H	14.232882	7.814412	1.584666	H	7.744599	2.678069	11.830850
C	14.626376	7.212257	3.613003	C	13.468033	5.598679	10.446702
H	14.528318	8.222576	4.004716	C	14.618994	4.799484	10.543267
C	14.913265	6.166490	4.492627	C	15.868257	5.396163	10.386947
C	14.928746	3.217995	2.005931	H	16.759779	4.774406	10.466548
H	15.228512	2.528485	2.802465	C	16.008230	6.762747	10.142890
C	13.560554	2.761244	1.485655	C	14.851028	7.530100	10.042176
H	12.810108	2.743375	2.285778	H	14.934194	8.597221	9.837255
H	13.625964	1.753269	1.057647	C	13.579296	6.969147	10.176028
H	13.192459	3.433381	0.699691	C	8.560359	2.923987	9.319680
C	15.989057	3.127825	0.901565	C	7.607300	3.602288	8.545957
H	15.729486	3.755926	0.040830	C	6.611842	2.862686	7.903962
H	16.079447	2.095152	0.543104	H	5.866481	3.397596	7.314037
H	16.973518	3.447224	1.262648	C	6.542131	1.476328	7.995285
C	15.051497	6.464554	5.976716	C	7.502614	0.824792	8.768003
H	15.371892	5.547774	6.484785	H	7.463313	-0.259743	8.866812

C	8.505109	1.523302	9.437067
Zn	10.086118	4.950622	5.209944
Zn	11.319719	4.044061	9.075675
Zn	13.785413	2.740316	5.674154
C	13.835248	-0.920884	4.971225
C	14.020573	-0.314613	3.583402
H	13.834239	0.763632	3.674558
C	13.010961	-0.855638	2.568911
H	13.220731	-1.897511	2.296527
H	11.989178	-0.793689	2.956611
H	13.055069	-0.265256	1.647103
C	15.447173	-0.489756	3.044656
H	15.518525	-0.086075	2.026193
H	16.189136	0.031820	3.657402
H	15.715837	-1.553244	3.004431
C	11.080911	7.871486	2.751404
H	11.518597	6.865616	2.772782
H	10.064233	7.772082	2.353528
H	11.667608	8.472238	2.049344
C	9.483901	7.961199	7.546252
H	8.423271	7.926310	7.268647
H	9.781667	6.932786	7.780128
H	9.577894	8.562120	8.456774
C	12.910622	11.304980	5.960773
H	13.052537	11.937964	5.078503
H	12.533300	11.934408	6.773462
H	13.899129	10.935468	6.263066
C	10.184342	4.272528	1.252133
H	9.330891	4.958849	1.199260
H	10.965995	4.782987	1.829811
H	10.563086	4.119789	0.236297
C	8.443902	1.641111	5.196274
H	9.129670	2.107048	5.916703
H	7.495858	2.187218	5.269193
H	8.270345	0.609436	5.519735
C	9.959240	-0.757866	1.018762
H	10.873854	-0.731162	0.416288
H	10.016863	-1.628125	1.680763
H	9.118841	-0.919865	0.332442
C	14.507231	3.321533	10.811705
H	13.885704	3.111033	11.690470
H	14.043987	2.812372	9.958779
H	15.495294	2.878508	10.974283
C	12.347170	7.817159	9.988008
H	11.759390	7.453181	9.135896
H	11.685757	7.798361	10.862349
H	12.619898	8.858447	9.787472
C	17.376690	7.378843	9.986114
H	17.311259	8.462580	9.845092
H	18.000394	7.191547	10.867808
H	17.902234	6.960500	9.119317
C	7.594647	5.104743	8.411330
H	8.355484	5.577523	9.036938
H	7.781311	5.404051	7.372150
H	6.615865	5.510047	8.695857
C	5.477591	0.701482	7.260024
H	5.259155	-0.248962	7.757878
H	4.544976	1.271413	7.190982
H	5.798213	0.471232	6.235795
C	9.470517	0.788973	10.331273
H	9.201637	0.908320	11.389600
H	9.472983	-0.282509	10.107912
H	10.489055	1.168309	10.221810

5-theta55

SCF (wB97x) = -8713.99493559

Pd	11.737350	3.778833	6.636813
H	11.980293	3.446197	4.980670
H	12.724678	3.134676	7.877633
H	10.424022	4.677586	7.210367
N	8.912150	4.140103	3.821969
C	7.771531	4.731769	3.489893
C	7.419612	6.046872	3.848863
H	6.436267	6.372908	3.529396
C	8.257988	7.053526	4.365766
N	9.463659	6.836362	4.872498
C	6.772134	3.984163	2.629701
H	7.150037	3.875555	1.606906
H	5.819948	4.517563	2.589638
H	6.601437	2.973535	3.013913
C	7.731770	8.472514	4.282577
H	7.842206	8.996364	5.237087
H	6.679356	8.480785	3.991285
H	8.299573	9.044506	3.539705
C	9.246261	2.911352	3.170428
C	9.825604	2.954073	1.891291
C	10.072691	1.755288	1.225107
H	10.503776	1.788955	0.224412
C	9.774161	0.519742	1.799870
C	9.269341	0.506526	3.098274
H	9.056649	-0.447932	3.579388
C	9.004576	1.684239	3.800656
C	10.299800	7.962166	5.154395
C	10.337133	8.522726	6.439556
C	11.174161	9.613794	6.674230
H	11.197980	10.050545	7.672596
C	11.974822	10.157744	5.671787
C	11.916017	9.583562	4.402938
H	12.527758	9.996906	3.600753
C	11.098253	8.488148	4.126801
N	15.308554	3.757907	4.842266
C	16.565680	3.402532	5.045497
C	16.952767	2.213347	5.694158
H	18.020811	2.077954	5.818248
C	16.160815	1.100369	6.024209
N	14.829739	1.068840	5.988607
C	17.684516	4.275959	4.517770
H	17.658014	4.308224	3.422808

H	18.659430	3.897473	4.831639	C	10.294741	4.654374	12.096421
H	17.572502	5.307150	4.867182	H	9.958990	4.842241	13.109563
C	16.925149	-0.153430	6.403420	C	9.364076	4.007224	11.264482
H	16.676420	-0.470314	7.419604	N	9.595649	3.636210	10.011199
H	18.001954	0.015918	6.344005	C	12.357368	5.730857	12.949011
H	16.663411	-0.984576	5.740685	H	13.313238	5.216705	13.096957
C	15.030794	4.863817	3.974213	H	11.782448	5.676170	13.875447
C	14.839212	4.617029	2.599647	H	12.593446	6.780159	12.744930
C	14.549504	5.692853	1.758851	C	8.001878	3.740781	11.874532
H	14.402800	5.514106	0.695147	H	7.227272	4.276473	11.314403
C	14.451350	6.985916	2.254821	H	7.968675	4.069044	12.915174
H	14.232882	7.814412	1.584666	H	7.744599	2.678069	11.830850
C	14.626376	7.212257	3.613003	C	13.468033	5.598679	10.446702
H	14.528318	8.222576	4.004716	C	14.618994	4.799484	10.543267
C	14.913265	6.166490	4.492627	C	15.868257	5.396163	10.386947
C	14.928746	3.217995	2.005931	H	16.759779	4.774406	10.466548
H	15.228512	2.528485	2.802465	C	16.008230	6.762747	10.142890
C	13.560554	2.761244	1.485655	C	14.851028	7.530100	10.042176
H	12.810108	2.743375	2.285778	H	14.934194	8.597221	9.837255
H	13.625964	1.753269	1.057647	C	13.579296	6.969147	10.176028
H	13.192459	3.433381	0.699691	C	8.560359	2.923987	9.319680
C	15.989057	3.127825	0.901565	C	7.607300	3.602288	8.545957
H	15.729486	3.755926	0.040830	C	6.611842	2.862686	7.903962
H	16.079447	2.095152	0.543104	H	5.866481	3.397596	7.314037
H	16.973518	3.447224	1.262648	C	6.542131	1.476328	7.995285
C	15.051497	6.464554	5.976716	C	7.502614	0.824792	8.768003
H	15.371892	5.547774	6.484785	H	7.463313	-0.259743	8.866812
C	16.098708	7.547955	6.262998	C	8.505109	1.523302	9.437067
H	17.082792	7.289062	5.853435	Zn	10.086118	4.950622	5.209944
H	16.203752	7.685935	7.345647	Zn	11.319719	4.044061	9.075675
H	15.803996	8.513733	5.833377	Zn	13.785413	2.740316	5.674154
C	13.693214	6.870883	6.557160	C	13.835248	-0.920884	4.971225
H	13.316886	7.783659	6.079686	C	14.020573	-0.314613	3.583402
H	13.781754	7.052922	7.632303	H	13.834239	0.763632	3.674558
H	12.947107	6.078779	6.407462	C	13.010961	-0.855638	2.568911
C	14.189957	-0.207692	6.138082	H	13.220731	-1.897511	2.296527
C	13.922529	-0.741407	7.412102	H	11.989178	-0.793689	2.956611
C	13.380895	-2.026337	7.499521	H	13.055069	-0.265256	1.647103
H	13.181178	-2.453485	8.480311	C	15.447173	-0.489756	3.044656
C	13.087467	-2.763253	6.363600	H	15.518525	-0.086075	2.026193
H	12.675318	-3.765581	6.450495	H	16.189136	0.031820	3.657402
C	13.297759	-2.200632	5.110727	H	15.715837	-1.553244	3.004431
H	13.030701	-2.770544	4.225561	C	11.080911	7.871486	2.751404
C	14.132287	0.048260	8.696099	H	11.518597	6.865616	2.772782
H	14.594757	1.007717	8.435324	H	10.064233	7.772082	2.353528
C	15.036126	-0.661330	9.712328	H	11.667608	8.472238	2.049344
H	14.586310	-1.599366	10.059286	C	9.483901	7.961199	7.546252
H	15.184441	-0.022717	10.591662	H	8.423271	7.926310	7.268647
H	16.022902	-0.900448	9.301903	H	9.781667	6.932786	7.780128
C	12.766508	0.336120	9.330256	H	9.577894	8.562120	8.456774
H	12.118646	0.871094	8.628308	C	12.910622	11.304980	5.960773
H	12.874694	0.944312	10.237453	H	13.052537	11.937964	5.078503
H	12.263894	-0.596967	9.614386	H	12.533300	11.934408	6.773462
N	12.171542	5.013076	10.607611	H	13.899129	10.935468	6.263066
C	11.592147	5.104020	11.799521	C	10.184342	4.272528	1.252133

H	9.330891	4.958849	1.199260
H	10.965995	4.782987	1.829811
H	10.563086	4.119789	0.236297
C	8.443902	1.641111	5.196274
H	9.129670	2.107048	5.916703
H	7.495858	2.187218	5.269193
H	8.270345	0.609436	5.519735
C	9.959240	-0.757866	1.018762
H	10.873854	-0.731162	0.416288
H	10.016863	-1.628125	1.680763
H	9.118841	-0.919865	0.332442
C	14.507231	3.321533	10.811705
H	13.885704	3.111033	11.690470
H	14.043987	2.812372	9.958779
H	15.495294	2.878508	10.974283
C	12.347170	7.817159	9.988008
H	11.759390	7.453181	9.135896
H	11.685757	7.798361	10.862349
H	12.619898	8.858447	9.787472
C	17.376690	7.378843	9.986114
H	17.311259	8.462580	9.845092
H	18.000394	7.191547	10.867808
H	17.902234	6.960500	9.119317
C	7.594647	5.104743	8.411330
H	8.355484	5.577523	9.036938
H	7.781311	5.404051	7.372150
H	6.615865	5.510047	8.695857
C	5.477591	0.701482	7.260024
H	5.259155	-0.248962	7.757878
H	4.544976	1.271413	7.190982
H	5.798213	0.471232	6.235795
C	9.470517	0.788973	10.331273
H	9.201637	0.908320	11.389600
H	9.472983	-0.282509	10.107912
H	10.489055	1.168309	10.221810

5-theta60

SCF (wB97x) = -8713.99132682

Pd	11.737350	3.778833	6.636813
H	11.844841	3.502897	4.956104
H	12.811736	3.078407	7.770166
H	10.468852	4.643870	7.346865
N	8.912150	4.140103	3.821969
C	7.771531	4.731769	3.489893
C	7.419612	6.046872	3.848863
H	6.436267	6.372908	3.529396
C	8.257988	7.053526	4.365766
N	9.463659	6.836362	4.872498
C	6.772134	3.984163	2.629701
H	7.150037	3.875555	1.606906
H	5.819948	4.517563	2.589638
H	6.601437	2.973535	3.013913
C	7.731770	8.472514	4.282577
H	7.842206	8.996364	5.237087
H	6.679356	8.480785	3.991285
H	8.299573	9.044506	3.539705
C	9.246261	2.911352	3.170428
C	9.825604	2.954073	1.891291
C	10.072691	1.755288	1.225107
H	10.503776	1.788955	0.224412
C	9.774161	0.519742	1.799870
C	9.269341	0.506526	3.098274
H	9.056649	-0.447932	3.579388
C	9.004576	1.684239	3.800656
C	10.299800	7.962166	5.154395
C	10.337133	8.522726	6.439556
C	11.174161	9.613794	6.674230
H	11.197980	10.050545	7.672596
C	11.974822	10.157744	5.671787
C	11.916017	9.583562	4.402938
H	12.527758	9.996906	3.600753
C	11.098253	8.488148	4.126801
N	15.308554	3.757907	4.842266
C	16.565680	3.402532	5.045497
C	16.952767	2.213347	5.694158
H	18.020811	2.077954	5.818248
C	16.160815	1.100369	6.024209
N	14.829739	1.068840	5.988607
C	17.684516	4.275959	4.517770
H	17.658014	4.308224	3.422808
H	18.659430	3.897473	4.831639
H	17.572502	5.307150	4.867182
C	16.925149	-0.153430	6.403420
H	16.676420	-0.470314	7.419604
H	18.001954	0.015918	6.344005
H	16.663411	-0.984576	5.740685
C	15.030794	4.863817	3.974213
C	14.839212	4.617029	2.599647
C	14.549504	5.692853	1.758851
H	14.402800	5.514106	0.695147
C	14.451350	6.985916	2.254821
H	14.232882	7.814412	1.584666
C	14.626376	7.212257	3.613003
H	14.528318	8.222576	4.004716
C	14.913265	6.166490	4.492627
C	14.928746	3.217995	2.005931
H	15.228512	2.528485	2.802465
C	13.560554	2.761244	1.485655
H	12.810108	2.743375	2.285778
H	13.625964	1.753269	1.057647
H	13.192459	3.433381	0.699691
C	15.989057	3.127825	0.901565
H	15.729486	3.755926	0.040830
H	16.079447	2.095152	0.543104
H	16.973518	3.447224	1.262648
C	15.051497	6.464554	5.976716
H	15.371892	5.547774	6.484785
C	16.098708	7.547955	6.262998

H	17.082792	7.289062	5.853435
H	16.203752	7.685935	7.345647
H	15.803996	8.513733	5.833377
C	13.693214	6.870883	6.557160
H	13.316886	7.783659	6.079686
H	13.781754	7.052922	7.632303
H	12.947107	6.078779	6.407462
C	14.189957	-0.207692	6.138082
C	13.922529	-0.741407	7.412102
C	13.380895	-2.026337	7.499521
H	13.181178	-2.453485	8.480311
C	13.087467	-2.763253	6.363600
H	12.675318	-3.765581	6.450495
C	13.297759	-2.200632	5.110727
H	13.030701	-2.770544	4.225561
C	14.132287	0.048260	8.696099
H	14.594757	1.007717	8.435324
C	15.036126	-0.661330	9.712328
H	14.586310	-1.599366	10.059286
H	15.184441	-0.022717	10.591662
H	16.022902	-0.900448	9.301903
C	12.766508	0.336120	9.330256
H	12.118646	0.871094	8.628308
H	12.874694	0.944312	10.237453
H	12.263894	-0.596967	9.614386
N	12.171542	5.013076	10.607611
C	11.592147	5.104020	11.799521
C	10.294741	4.654374	12.096421
H	9.958990	4.842241	13.109563
C	9.364076	4.007224	11.264482
N	9.595649	3.636210	10.011199
C	12.357368	5.730857	12.949011
H	13.313238	5.216705	13.096957
H	11.782448	5.676170	13.875447
H	12.593446	6.780159	12.744930
C	8.001878	3.740781	11.874532
H	7.227272	4.276473	11.314403
H	7.968675	4.069044	12.915174
H	7.744599	2.678069	11.830850
C	13.468033	5.598679	10.446702
C	14.618994	4.799484	10.543267
C	15.868257	5.396163	10.386947
H	16.759779	4.774406	10.466548
C	16.008230	6.762747	10.142890
C	14.851028	7.530100	10.042176
H	14.934194	8.597221	9.837255
C	13.579296	6.969147	10.176028
C	8.560359	2.923987	9.319680
C	7.607300	3.602288	8.545957
C	6.611842	2.862686	7.903962
H	5.866481	3.397596	7.314037
C	6.542131	1.476328	7.995285
C	7.502614	0.824792	8.768003
H	7.463313	-0.259743	8.866812
C	8.505109	1.523302	9.437067

Zn	10.086118	4.950622	5.209944
Zn	11.319719	4.044061	9.075675
Zn	13.785413	2.740316	5.674154
C	13.835248	-0.920884	4.971225
C	14.020573	-0.314613	3.583402
H	13.834239	0.763632	3.674558
C	13.010961	-0.855638	2.568911
H	13.220731	-1.897511	2.296527
H	11.989178	-0.793689	2.956611
H	13.055069	-0.265256	1.647103
C	15.447173	-0.489756	3.044656
H	15.518525	-0.086075	2.026193
H	16.189136	0.031820	3.657402
H	15.715837	-1.553244	3.004431
C	11.080911	7.871486	2.751404
H	11.518597	6.865616	2.772782
H	10.064233	7.772082	2.353528
H	11.667608	8.472238	2.049344
C	9.483901	7.961199	7.546252
H	8.423271	7.926310	7.268647
H	9.781667	6.932786	7.780128
H	9.577894	8.562120	8.456774
C	12.910622	11.304980	5.960773
H	13.052537	11.937964	5.078503
H	12.533300	11.934408	6.773462
H	13.899129	10.935468	6.263066
C	10.184342	4.272528	1.252133
H	9.330891	4.958849	1.199260
H	10.965995	4.782987	1.829811
H	10.563086	4.119789	0.236297
C	8.443902	1.641111	5.196274
H	9.129670	2.107048	5.916703
H	7.495858	2.187218	5.269193
H	8.270345	0.609436	5.519735
C	9.959240	-0.757866	1.018762
H	10.873854	-0.731162	0.416288
H	10.016863	-1.628125	1.680763
H	9.118841	-0.919865	0.332442
C	14.507231	3.321533	10.811705
H	13.885704	3.111033	11.690470
H	14.043987	2.812372	9.958779
H	15.495294	2.878508	10.974283
C	12.347170	7.817159	9.988008
H	11.759390	7.453181	9.135896
H	11.685757	7.798361	10.862349
H	12.619898	8.858447	9.787472
C	17.376690	7.378843	9.986114
H	17.311259	8.462580	9.845092
H	18.000394	7.191547	10.867808
H	17.902234	6.960500	9.119317
C	7.594647	5.104743	8.411330
H	8.355484	5.577523	9.036938
H	7.781311	5.404051	7.372150
H	6.615865	5.510047	8.695857
C	5.477591	0.701482	7.260024

H	5.259155	-0.248962	7.757878
H	4.544976	1.271413	7.190982
H	5.798213	0.471232	6.235795
C	9.470517	0.788973	10.331273
H	9.201637	0.908320	11.389600
H	9.472983	-0.282509	10.107912
H	10.489055	1.168309	10.221810

6a

SCF (wB97x) = -4447.56244900
E(SCF)+ZPE(0 K)= -4445.593257
H(298 K)= -4445.482319
G(298 K)= -4445.738363
Lowest Frequency = 13.1985cm-1

Pd	-0.142429	0.035084	0.159632
Mg	0.152962	2.182314	-1.156424
Mg	1.870442	-1.344588	0.883396
Mg	-2.561638	-0.792358	0.239145
H	-0.510224	-1.476201	0.882259
H	-1.465257	0.957727	-0.416443
N	1.422945	2.604738	-2.725557
C	1.128490	3.543450	-3.616805
C	0.092111	4.486365	-3.471696
H	-0.016421	5.178176	-4.298970
C	-0.673179	4.799708	-2.328526
N	-0.757326	4.044040	-1.238078
C	1.974300	3.680755	-4.867626
H	3.003344	3.954115	-4.610706
H	1.567673	4.442600	-5.535589
H	2.026194	2.728684	-5.406371
C	-1.401281	6.130023	-2.388894
H	-2.483816	5.993932	-2.334286
H	-1.162851	6.662746	-3.311164
H	-1.121669	6.759791	-1.537692
C	2.693674	1.948436	-2.854075
C	3.810537	2.528276	-2.217430
C	5.056710	1.917848	-2.360512
H	5.927739	2.364784	-1.884519
C	5.204476	0.745779	-3.088635
H	6.183739	0.283547	-3.187191
C	4.091299	0.165815	-3.682152
H	4.203380	-0.759061	-4.245220
C	2.828399	0.752789	-3.586061
C	3.703059	3.794955	-1.377594
H	2.660136	4.129102	-1.398584
C	4.076130	3.515683	0.083939
H	3.441987	2.735586	0.524691
H	3.971431	4.424274	0.690210
H	5.116388	3.179498	0.173262
C	4.555711	4.933988	-1.949930
H	4.422534	5.847255	-1.357351
H	4.277681	5.161570	-2.985545
H	5.622999	4.681624	-1.937297
C	1.641669	0.086853	-4.264355
H	0.818678	0.808357	-4.264364
C	1.922001	-0.285003	-5.725199
H	2.285586	0.573588	-6.302316
H	1.004652	-0.651754	-6.200919
H	2.670430	-1.082236	-5.804314
C	1.187160	-1.147343	-3.474427
H	1.972794	-1.914827	-3.452883
H	0.290272	-1.593145	-3.925650
H	0.949327	-0.883786	-2.435454
C	-1.475782	4.588600	-0.120055
C	-2.884928	4.547474	-0.064079
C	-3.531603	5.224885	0.971825
H	-4.619438	5.222243	1.010396
C	-2.818765	5.887705	1.959747
H	-3.341162	6.417174	2.752749
C	-1.431365	5.834142	1.950531
H	-0.876449	6.315300	2.751391
C	-0.739084	5.181938	0.928925
C	-3.733301	3.746431	-1.045827
H	-3.066720	3.330623	-1.811306
C	-4.812927	4.579562	-1.750198
H	-5.540338	4.977039	-1.032505
H	-5.364527	3.953655	-2.462073
H	-4.398103	5.427744	-2.304198
C	-4.402819	2.574101	-0.312117
H	-3.658544	1.945530	0.189523
H	-4.973583	1.949098	-1.010221
H	-5.099840	2.937131	0.453989
C	0.785652	5.149559	0.949163
H	1.107558	4.236740	0.430153
C	1.359824	5.075919	2.367932
H	1.247745	6.025959	2.904397
H	2.430144	4.844874	2.329233
H	0.870027	4.289772	2.953229
C	1.387575	6.337459	0.187062
H	1.111515	6.322395	-0.873050
H	2.482989	6.318591	0.247337
H	1.041940	7.285042	0.619356
N	3.071671	-1.205260	2.559969
C	3.302479	-2.298478	3.268785
C	3.062444	-3.607440	2.797631
H	3.231284	-4.398226	3.519686
C	2.864191	-4.037417	1.469019
N	2.616803	-3.233190	0.438511
C	3.900047	-2.181760	4.655011
H	4.920874	-1.787873	4.593881
H	3.933383	-3.152361	5.153995
H	3.326517	-1.482392	5.271464
C	2.977261	-5.533604	1.247592
H	1.993871	-5.949987	1.006142
H	3.352953	-6.035594	2.141369
H	3.638829	-5.764390	0.407920
C	3.550435	0.058823	3.036202
C	4.842057	0.488218	2.669588

C	5.256904	1.766679	3.046285	H	6.131346	-4.823899	-0.403809
H	6.250387	2.109632	2.762309	N	-3.852474	-0.978733	1.854083
C	4.425240	2.609948	3.770769	C	-5.168304	-1.005428	1.719392
H	4.761324	3.606129	4.048968	C	-5.841531	-0.870142	0.485626
C	3.167285	2.163227	4.152793	H	-6.917073	-0.751583	0.561310
H	2.522578	2.816544	4.737890	C	-5.343042	-1.007132	-0.817001
C	2.714707	0.888169	3.808630	N	-4.070096	-1.264462	-1.127381
C	5.794334	-0.396467	1.877200	C	-6.068968	-1.183208	2.928127
H	5.304030	-1.362315	1.713007	H	-5.641448	-1.895977	3.638760
C	6.088692	0.211510	0.503057	H	-7.053439	-1.539538	2.617262
H	5.169982	0.363339	-0.075037	H	-6.204788	-0.236455	3.461176
H	6.752607	-0.441219	-0.077196	C	-6.372377	-0.836868	-1.921352
H	6.583790	1.186609	0.599780	H	-6.245865	0.158365	-2.364891
C	7.097459	-0.661654	2.641307	H	-7.389025	-0.901033	-1.527067
H	7.667622	0.262565	2.795070	H	-6.257229	-1.566394	-2.725388
H	7.734499	-1.355618	2.079461	C	-3.298841	-1.053662	3.172095
H	6.906364	-1.101344	3.626885	C	-2.620270	-2.228211	3.561645
C	1.346282	0.429140	4.283441	C	-2.087996	-2.292262	4.850265
H	1.226004	-0.626673	4.014507	H	-1.569904	-3.190305	5.174603
C	1.205237	0.535453	5.807572	C	-2.214161	-1.227642	5.734169
H	1.998923	-0.010143	6.331811	H	-1.805777	-1.302561	6.739386
H	0.239639	0.123507	6.121902	C	-2.854074	-0.065913	5.325125
H	1.246133	1.579542	6.141143	H	-2.924539	0.773248	6.013684
C	0.222209	1.202702	3.592819	C	-3.396413	0.051381	4.043814
H	0.238873	2.263450	3.879188	C	-2.505738	-3.420657	2.618054
H	-0.747370	0.786938	3.881662	H	-2.391899	-3.029141	1.598909
H	0.302076	1.144191	2.498243	C	-1.276052	-4.292917	2.883479
C	2.672513	-3.787850	-0.883939	H	-1.356973	-4.838034	3.831690
C	1.537265	-4.388497	-1.459523	H	-0.355177	-3.697078	2.906652
C	1.628334	-4.907693	-2.753695	H	-1.175639	-5.042904	2.089949
H	0.751896	-5.368308	-3.205125	C	-3.781759	-4.272939	2.629780
C	2.811418	-4.845860	-3.469983	H	-3.967381	-4.678902	3.632067
H	2.869904	-5.261239	-4.473009	H	-3.684116	-5.116887	1.934313
C	3.923908	-4.237359	-2.900074	H	-4.659161	-3.690576	2.327974
H	4.842469	-4.184331	-3.476365	C	-4.025889	1.375047	3.619179
C	3.881048	-3.691969	-1.615892	H	-4.621145	1.203668	2.716848
C	0.200432	-4.471235	-0.739470	C	-4.961566	1.957996	4.686223
H	0.331313	-4.083259	0.277162	H	-5.701614	1.226782	5.031897
C	-0.314690	-5.913143	-0.633948	H	-5.498471	2.823506	4.281424
H	-0.532032	-6.334281	-1.623064	H	-4.405234	2.305284	5.564711
H	-1.245605	-5.945835	-0.054185	C	-2.951747	2.404903	3.248401
H	0.409787	-6.576962	-0.149873	H	-2.319983	2.639664	4.114032
C	-0.836554	-3.587593	-1.440241	H	-3.415836	3.335951	2.906010
H	-0.488004	-2.553050	-1.519459	H	-2.297588	2.041930	2.446099
H	-1.777463	-3.588236	-0.874027	C	-3.814770	-1.778312	-2.443199
H	-1.059268	-3.948261	-2.452525	C	-3.388884	-0.939270	-3.489863
C	5.115512	-3.002454	-1.035772	C	-3.147153	-1.501194	-4.747423
H	4.765192	-2.087535	-0.538129	H	-2.822542	-0.858444	-5.563741
C	6.127878	-2.591375	-2.109386	C	-3.331669	-2.854647	-4.979831
H	6.897829	-1.948779	-1.668361	H	-3.148773	-3.272117	-5.966843
H	6.640782	-3.464320	-2.531962	C	-3.755831	-3.675201	-3.940732
H	5.654916	-2.040167	-2.927049	H	-3.895107	-4.736632	-4.129093
C	5.847342	-3.850472	0.016511	C	-3.995462	-3.166687	-2.664745
H	6.768081	-3.341160	0.329588	C	-3.210467	0.562717	-3.316556
H	5.249338	-4.020245	0.915093	H	-3.404609	0.805967	-2.266502

C	-4.195461	1.361089	-4.181142
H	-4.012870	1.187119	-5.248641
H	-4.080174	2.436924	-3.996829
H	-5.236514	1.088603	-3.977477
C	-1.775752	0.990553	-3.635544
H	-1.062848	0.417353	-3.033508
H	-1.635071	2.064835	-3.445091
H	-1.531240	0.818501	-4.691764
C	-4.450534	-4.109421	-1.549977
H	-4.089122	-3.691246	-0.600538
C	-3.880299	-5.526664	-1.692642
H	-4.387344	-6.085309	-2.488992
H	-4.037481	-6.084288	-0.761416
H	-2.809106	-5.523546	-1.911578
C	-5.980973	-4.217373	-1.450412
H	-6.449372	-3.283591	-1.131963
H	-6.252949	-4.986206	-0.716980
H	-6.408765	-4.508671	-2.418135
H	1.480880	0.548380	-0.055641

6b

SCF (wB97x) = -4439.06079288
E(SCF)+ZPE(0 K)= -4437.098179
H(298 K)= -4436.986909
G(298 K)= -4437.243799
Lowest Frequency = 14.0085cm-1

Pt	-0.125460	0.016605	0.163408
Mg	0.092862	2.247584	-1.132905
Mg	1.954609	-1.356290	0.861027
Mg	-2.583458	-0.864226	0.234620
H	-0.470523	-1.487679	0.856444
H	-1.483037	0.931386	-0.331515
N	1.339970	2.745454	-2.688467
C	1.019729	3.697553	-3.555345
C	-0.045707	4.604045	-3.386912
H	-0.170809	5.317363	-4.192809
C	-0.825979	4.858122	-2.239659
N	-0.893412	4.065745	-1.173533
C	1.864385	3.892133	-4.798010
H	2.885034	4.180790	-4.527479
H	1.442023	4.662629	-5.444228
H	1.940828	2.957132	-5.361751
C	-1.590074	6.168516	-2.259831
H	-2.666816	6.003469	-2.187740
H	-1.380633	6.726428	-3.172991
H	-1.310582	6.784658	-1.399307
C	2.621257	2.114778	-2.833923
C	3.729244	2.698101	-2.185469
C	4.984938	2.111906	-2.343643
H	5.848654	2.562478	-1.858437
C	5.150361	0.958432	-3.097012
H	6.136627	0.514742	-3.207111
C	4.045427	0.371148	-3.698223
H	4.169916	-0.541817	-4.277655
C	2.773019	0.934543	-3.587293
C	3.599416	3.937677	-1.309524
H	2.554094	4.262894	-1.335345
C	3.954552	3.612690	0.147001
H	3.320294	2.815021	0.553634
H	3.837367	4.499043	0.781783
H	4.994558	3.278758	0.238302
C	4.449239	5.101672	-1.832310
H	4.296828	5.994398	-1.215097
H	4.186694	5.357508	-2.864492
H	5.518111	4.860570	-1.809030
C	1.595275	0.257902	-4.270419
H	0.755269	0.958209	-4.238056
C	1.868158	-0.060052	-5.745096
H	2.197338	0.825579	-6.300064
H	0.957815	-0.441232	-6.220960
H	2.639811	-0.829801	-5.856885
C	1.182611	-1.011288	-3.513042
H	1.989140	-1.756245	-3.522540
H	0.296212	-1.468311	-3.972033
H	0.944165	-0.786613	-2.465215
C	-1.621631	4.558295	-0.038297
C	-3.028519	4.480040	0.020293
C	-3.689464	5.117290	1.072418
H	-4.776575	5.086748	1.112392
C	-2.990215	5.773430	2.074394
H	-3.523111	6.271859	2.879929
C	-1.602161	5.749422	2.063871
H	-1.056398	6.220332	2.876744
C	-0.896949	5.138178	1.026108
C	-3.855708	3.674342	-0.975114
H	-3.177946	3.292785	-1.747955
C	-4.958110	4.489946	-1.663080
H	-5.690603	4.860460	-0.937336
H	-5.498088	3.861725	-2.380521
H	-4.565711	5.354154	-2.207184
C	-4.487594	2.469642	-0.260770
H	-3.722769	1.855286	0.226910
H	-5.043862	1.841659	-0.966617
H	-5.189499	2.798379	0.515062
C	0.627891	5.132431	1.048207
H	0.966727	4.249145	0.491303
C	1.199044	5.004731	2.464068
H	1.066762	5.926083	3.042727
H	2.273049	4.796349	2.420128
H	0.722361	4.183515	3.009448
C	1.208509	6.361051	0.336612
H	0.926406	6.387279	-0.721143
H	2.303665	6.356822	0.390687
H	0.850671	7.282755	0.810740
N	3.184254	-1.230544	2.516201
C	3.473637	-2.333233	3.187309
C	3.264436	-3.637007	2.687001
H	3.481136	-4.440603	3.380983

C	3.039725	-4.035799	1.353508
N	2.742368	-3.208672	0.354868
C	4.108872	-2.232460	4.556977
H	5.117165	-1.813322	4.472161
H	4.180137	-3.211938	5.031825
H	3.539316	-1.559634	5.204060
C	3.184004	-5.520434	1.083267
H	2.207013	-5.950548	0.842851
H	3.585825	-6.040533	1.953854
H	3.836145	-5.706741	0.226201
C	3.617778	0.040277	3.016203
C	4.886312	0.529467	2.643498
C	5.253933	1.815604	3.041380
H	6.228832	2.204001	2.752425
C	4.397649	2.609562	3.792384
H	4.696495	3.612525	4.086969
C	3.162793	2.105584	4.177256
H	2.497194	2.720709	4.779834
C	2.756489	0.820807	3.811324
C	5.859789	-0.297910	1.815595
H	5.408913	-1.281593	1.645546
C	6.093421	0.346542	0.446572
H	5.155886	0.472120	-0.105898
H	6.768131	-0.265831	-0.162967
H	6.549538	1.338937	0.550703
C	7.191075	-0.516275	2.544344
H	7.723149	0.429298	2.699203
H	7.844159	-1.171943	1.957239
H	7.044234	-0.977893	3.526543
C	1.410485	0.302756	4.290518
H	1.303970	-0.734649	3.953978
C	1.313903	0.305104	5.821973
H	2.126308	-0.263816	6.287450
H	0.362160	-0.136938	6.135721
H	1.355307	1.325278	6.220873
C	0.252014	1.098226	3.687598
H	0.269038	2.140765	4.032528
H	-0.700168	0.656443	3.993171
H	0.286290	1.095857	2.589708
C	2.783907	-3.715473	-0.986257
C	1.650303	-4.317100	-1.563614
C	1.727704	-4.785462	-2.877782
H	0.852025	-5.245962	-3.330241
C	2.895621	-4.672308	-3.612076
H	2.943570	-5.048330	-4.630701
C	4.005661	-4.061435	-3.040481
H	4.911462	-3.965843	-3.630869
C	3.975598	-3.565098	-1.736355
C	0.329199	-4.454398	-0.822887
H	0.472839	-4.097154	0.203075
C	-0.146976	-5.911501	-0.754529
H	-0.367905	-6.306722	-1.752765
H	-1.067411	-5.987593	-0.163536
H	0.601347	-6.570397	-0.302737
C	-0.741260	-3.572855	-1.474746
H	-0.422181	-2.526880	-1.515016
H	-1.674490	-3.624084	-0.898307
H	-0.968328	-3.900560	-2.496528
C	5.203324	-2.864816	-1.154656
H	4.837585	-1.980152	-0.615763
C	6.179948	-2.384992	-2.232230
H	6.943621	-1.741870	-1.782958
H	6.704420	-3.226303	-2.700394
H	5.674789	-1.814655	-3.015796
C	5.980345	-3.728709	-0.148992
H	6.893955	-3.206355	0.160522
H	5.409328	-3.944771	0.756281
H	6.279720	-4.678340	-0.609100
N	-3.851508	-1.107413	1.847187
C	-5.167981	-1.154376	1.722719
C	-5.851589	-1.018772	0.494515
H	-6.928694	-0.925412	0.575990
C	-5.355499	-1.124315	-0.812529
N	-4.077198	-1.341254	-1.129641
C	-6.051169	-1.360671	2.938420
H	-5.625353	-2.114981	3.605498
H	-7.049418	-1.678176	2.633475
H	-6.148265	-0.437439	3.517545
C	-6.390399	-0.964527	-1.911770
H	-6.305174	0.048274	-2.322762
H	-7.402629	-1.083987	-1.521503
H	-6.241486	-1.662932	-2.736675
C	-3.281022	-1.193750	3.156831
C	-2.573756	-2.359846	3.517073
C	-2.030817	-2.438791	4.800581
H	-1.491303	-3.331762	5.102418
C	-2.172423	-1.394574	5.705637
H	-1.756475	-1.480911	6.706511
C	-2.832518	-0.235354	5.321629
H	-2.908788	0.591322	6.023894
C	-3.383655	-0.103641	4.045996
C	-2.432884	-3.525330	2.544270
H	-2.342442	-3.105849	1.533855
C	-1.172893	-4.362548	2.775848
H	-1.224781	-4.930485	3.711595
H	-0.273141	-3.736791	2.803507
H	-1.056626	-5.090427	1.965287
C	-3.682211	-4.415444	2.545355
H	-3.843491	-4.852954	3.537546
H	-3.568333	-5.236768	1.826920
H	-4.578840	-3.850797	2.270067
C	-4.013481	1.222826	3.633234
H	-4.630569	1.052103	2.745849
C	-4.919970	1.819512	4.716085
H	-5.666739	1.100952	5.071767
H	-5.447819	2.694336	4.321991
H	-4.344153	2.155686	5.585349
C	-2.932942	2.234665	3.231854
H	-2.289435	2.475049	4.086102
H	-3.388844	3.165112	2.879495

H	-2.291374	1.847808	2.431171
C	-3.798510	-1.822699	-2.452300
C	-3.385009	-0.951822	-3.477548
C	-3.109339	-1.482844	-4.741235
H	-2.793003	-0.815572	-5.540615
C	-3.247920	-2.836956	-4.999430
H	-3.038420	-3.230676	-5.990530
C	-3.657899	-3.689359	-3.980847
H	-3.758806	-4.751217	-4.188634
C	-3.929427	-3.211937	-2.699264
C	-3.256200	0.551225	-3.272823
H	-3.464344	0.764399	-2.219325
C	-4.266255	1.331407	-4.124360
H	-4.073886	1.185173	-5.193501
H	-4.191360	2.405996	-3.918404
H	-5.296444	1.016650	-3.930418
C	-1.835331	1.034575	-3.574479
H	-1.107279	0.477077	-2.976051
H	-1.736100	2.110237	-3.369044
H	-1.578584	0.887761	-4.630668
C	-4.360566	-4.187213	-1.603526
H	-4.015467	-3.772402	-0.646772
C	-3.745585	-5.582755	-1.766348
H	-4.226781	-6.141011	-2.577859
H	-3.895500	-6.162550	-0.848500
H	-2.673551	-5.542460	-1.973095
C	-5.887119	-4.342349	-1.513162
H	-6.385555	-3.429143	-1.183648
H	-6.139580	-5.129950	-0.794185
H	-6.300316	-4.631168	-2.487042
H	1.403982	0.697933	-0.081672

6b-theta30

SCF (wB97x) = -4438.93008717

Pt	-0.125460	0.016605	0.163408
Mg	0.092862	2.247584	-1.132905
Mg	1.954609	-1.356290	0.861027
Mg	-2.583458	-0.864226	0.234620
H	-1.240629	-1.181699	0.590847
H	-0.852439	1.364399	-0.598000
N	1.339970	2.745454	-2.688467
C	1.019729	3.697553	-3.555345
C	-0.045707	4.604045	-3.386912
H	-0.170809	5.317363	-4.192809
C	-0.825979	4.858122	-2.239659
N	-0.893412	4.065745	-1.173533
C	1.864385	3.892133	-4.798010
H	2.885034	4.180790	-4.527479
H	1.442023	4.662629	-5.444228
H	1.940828	2.957132	-5.361751
C	-1.590074	6.168516	-2.259831
H	-2.666816	6.003469	-2.187740
H	-1.380633	6.726428	-3.172991
H	-1.310582	6.784658	-1.399307
C	2.621257	2.114778	-2.833923
C	3.729244	2.698101	-2.185469
C	4.984938	2.111906	-2.343643
H	5.848654	2.562478	-1.858437
C	5.150361	0.958432	-3.097012
H	6.136627	0.514742	-3.207111
C	4.045427	0.371148	-3.698223
H	4.169916	-0.541817	-4.277655
C	2.773019	0.934543	-3.587293
C	3.599416	3.937677	-1.309524
H	2.554094	4.262894	-1.335345
C	3.954552	3.612690	0.147001
H	3.320294	2.815021	0.553634
H	3.837367	4.499043	0.781783
H	4.994558	3.278758	0.238302
C	4.449239	5.101672	-1.832310
H	4.296828	5.994398	-1.215097
H	4.186694	5.357508	-2.864492
H	5.518111	4.860570	-1.809030
C	1.595275	0.257902	-4.270419
H	0.755269	0.958209	-4.238056
C	1.868158	-0.060052	-5.745096
H	2.197338	0.825579	-6.300064
H	0.957815	-0.441232	-6.220960
H	2.639811	-0.829801	-5.856885
C	1.182611	-1.011288	-3.513042
H	1.989140	-1.756245	-3.522540
H	0.296212	-1.468311	-3.972033
H	0.944165	-0.786613	-2.465215
C	-1.621631	4.558295	-0.038297
C	-3.028519	4.480040	0.020293
C	-3.689464	5.117290	1.072418
H	-4.776575	5.086748	1.112392
C	-2.990215	5.773430	2.074394
H	-3.523111	6.271859	2.879929
C	-1.602161	5.749422	2.063871
H	-1.056398	6.220332	2.876744
C	-0.896949	5.138178	1.026108
C	-3.855708	3.674342	-0.975114
H	-3.177946	3.292785	-1.747955
C	-4.958110	4.489946	-1.663080
H	-5.690603	4.860460	-0.937336
H	-5.498088	3.861725	-2.380521
H	-4.565711	5.354154	-2.207184
C	-4.487594	2.469642	-0.260770
H	-3.722769	1.855286	0.226910
H	-5.043862	1.841659	-0.966617
H	-5.189499	2.798379	0.515062
C	0.627891	5.132431	1.048207
H	0.966727	4.249145	0.491303
C	1.199044	5.004731	2.464068
H	1.066762	5.926083	3.042727
H	2.273049	4.796349	2.420128
H	0.722361	4.183515	3.009448

C	1.208509	6.361051	0.336612
H	0.926406	6.387279	-0.721143
H	2.303665	6.356822	0.390687
H	0.850671	7.282755	0.810740
N	3.184254	-1.230544	2.516201
C	3.473637	-2.333233	3.187309
C	3.264436	-3.637007	2.687001
H	3.481136	-4.440603	3.380983
C	3.039725	-4.035799	1.353508
N	2.742368	-3.208672	0.354868
C	4.108872	-2.232460	4.556977
H	5.117165	-1.813322	4.472161
H	4.180137	-3.211938	5.031825
H	3.539316	-1.559634	5.204060
C	3.184004	-5.520434	1.083267
H	2.207013	-5.950548	0.842851
H	3.585825	-6.040533	1.953854
H	3.836145	-5.706741	0.226201
C	3.617778	0.040277	3.016203
C	4.886312	0.529467	2.643498
C	5.253933	1.815604	3.041380
H	6.228832	2.204001	2.752425
C	4.397649	2.609562	3.792384
H	4.696495	3.612525	4.086969
C	3.162793	2.105584	4.177256
H	2.497194	2.720709	4.779834
C	2.756489	0.820807	3.811324
C	5.859789	-0.297910	1.815595
H	5.408913	-1.281593	1.645546
C	6.093421	0.346542	0.446572
H	5.155886	0.472120	-0.105898
H	6.768131	-0.265831	-0.162967
H	6.549538	1.338937	0.550703
C	7.191075	-0.516275	2.544344
H	7.723149	0.429298	2.699203
H	7.844159	-1.171943	1.957239
H	7.044234	-0.977893	3.526543
C	1.410485	0.302756	4.290518
H	1.303970	-0.734649	3.953978
C	1.313903	0.305104	5.821973
H	2.126308	-0.263816	6.287450
H	0.362160	-0.136938	6.135721
H	1.355307	1.325278	6.220873
C	0.252014	1.098226	3.687598
H	0.269038	2.140765	4.032528
H	-0.700168	0.656443	3.993171
H	0.286290	1.095857	2.589708
C	2.783907	-3.715473	-0.986257
C	1.650303	-4.317100	-1.563614
C	1.727704	-4.785462	-2.877782
H	0.852025	-5.245962	-3.330241
C	2.895621	-4.672308	-3.612076
H	2.943570	-5.048330	-4.630701
C	4.005661	-4.061435	-3.040481
H	4.911462	-3.965843	-3.630869
C	3.975598	-3.565098	-1.736355
C	0.329199	-4.454398	-0.822887
H	0.472839	-4.097154	0.203075
C	-0.146976	-5.911501	-0.754529
H	-0.367905	-6.306722	-1.752765
H	-1.067411	-5.987593	-0.163536
H	0.601347	-6.570397	-0.302737
C	-0.741260	-3.572855	-1.474746
H	-0.422181	-2.526880	-1.515016
H	-1.674490	-3.624084	-0.898307
H	-0.968328	-3.900560	-2.496528
C	5.203324	-2.864816	-1.154656
H	4.837585	-1.980152	-0.615763
C	6.179948	-2.384992	-2.232230
H	6.943621	-1.741870	-1.782958
H	6.704420	-3.226303	-2.700394
H	5.674789	-1.814655	-3.015796
C	5.980345	-3.728709	-0.148992
H	6.893955	-3.206355	0.160522
H	5.409328	-3.944771	0.756281
H	6.279720	-4.678340	-0.609100
N	-3.851508	-1.107413	1.847187
C	-5.167981	-1.154376	1.722719
C	-5.851589	-1.018772	0.494515
H	-6.928694	-0.925412	0.575990
C	-5.355499	-1.124315	-0.812529
N	-4.077198	-1.341254	-1.129641
C	-6.051169	-1.360671	2.938420
H	-5.625353	-2.114981	3.605498
H	-7.049418	-1.678176	2.633475
H	-6.148265	-0.437439	3.517545
C	-6.390399	-0.964527	-1.911770
H	-6.305174	0.048274	-2.322762
H	-7.402629	-1.083987	-1.521503
H	-6.241486	-1.662932	-2.736675
C	-3.281022	-1.193750	3.156831
C	-2.573756	-2.359846	3.517073
C	-2.030817	-2.438791	4.800581
H	-1.491303	-3.331762	5.102418
C	-2.172423	-1.394574	5.705637
H	-1.756475	-1.480911	6.706511
C	-2.832518	-0.235354	5.321629
H	-2.908788	0.591322	6.023894
C	-3.383655	-0.103641	4.045996
C	-2.432884	-3.525330	2.544270
H	-2.342442	-3.105849	1.533855
C	-1.172893	-4.362548	2.775848
H	-1.224781	-4.930485	3.711595
H	-0.273141	-3.736791	2.803507
H	-1.056626	-5.090427	1.965287
C	-3.682211	-4.415444	2.545355
H	-3.843491	-4.852954	3.537546
H	-3.568333	-5.236768	1.826920
H	-4.578840	-3.850797	2.270067
C	-4.013481	1.222826	3.633234

H	-4.630569	1.052103	2.745849
C	-4.919970	1.819512	4.716085
H	-5.666739	1.100952	5.071767
H	-5.447819	2.694336	4.321991
H	-4.344153	2.155686	5.585349
C	-2.932942	2.234665	3.231854
H	-2.289435	2.475049	4.086102
H	-3.388844	3.165112	2.879495
H	-2.291374	1.847808	2.431171
C	-3.798510	-1.822699	-2.452300
C	-3.385009	-0.951822	-3.477548
C	-3.109339	-1.482844	-4.741235
H	-2.793003	-0.815572	-5.540615
C	-3.247920	-2.836956	-4.999430
H	-3.038420	-3.230676	-5.990530
C	-3.657899	-3.689359	-3.980847
H	-3.758806	-4.751217	-4.188634
C	-3.929427	-3.211937	-2.699264
C	-3.256200	0.551225	-3.272823
H	-3.464344	0.764399	-2.219325
C	-4.266255	1.331407	-4.124360
H	-4.073886	1.185173	-5.193501
H	-4.191360	2.405996	-3.918404
H	-5.296444	1.016650	-3.930418
C	-1.835331	1.034575	-3.574479
H	-1.107279	0.477077	-2.976051
H	-1.736100	2.110237	-3.369044
H	-1.578584	0.887761	-4.630668
C	-4.360566	-4.187213	-1.603526
H	-4.015467	-3.772402	-0.646772
C	-3.745585	-5.582755	-1.766348
H	-4.226781	-6.141011	-2.577859
H	-3.895500	-6.162550	-0.848500
H	-2.673551	-5.542460	-1.973095
C	-5.887119	-4.342349	-1.513162
H	-6.385555	-3.429143	-1.183648
H	-6.139580	-5.129950	-0.794185
H	-6.300316	-4.631168	-2.487042
H	1.554870	-0.132467	0.296575

6b-theta35

SCF (wB97x) = -4438.99466281

Pt	-0.125460	0.016605	0.163408
Mg	0.092862	2.247584	-1.132905
Mg	1.954609	-1.356290	0.861027
Mg	-2.583458	-0.864226	0.234620
H	-1.127217	-1.258585	0.645706
H	-0.984549	1.305894	-0.560808
N	1.339970	2.745454	-2.688467
C	1.019729	3.697553	-3.555345
C	-0.045707	4.604045	-3.386912
H	-0.170809	5.317363	-4.192809
C	-0.825979	4.858122	-2.239659
N	-0.893412	4.065745	-1.173533
C	1.864385	3.892133	-4.798010
H	2.885034	4.180790	-4.527479
H	1.442023	4.662629	-5.444228
H	1.940828	2.957132	-5.361751
C	-1.590074	6.168516	-2.259831
H	-2.666816	6.003469	-2.187740
H	-1.380633	6.726428	-3.172991
H	-1.310582	6.784658	-1.399307
C	2.621257	2.114778	-2.833923
C	3.729244	2.698101	-2.185469
C	4.984938	2.111906	-2.343643
H	5.848654	2.562478	-1.858437
C	5.150361	0.958432	-3.097012
H	6.136627	0.514742	-3.207111
C	4.045427	0.371148	-3.698223
H	4.169916	-0.541817	-4.277655
C	2.773019	0.934543	-3.587293
C	3.599416	3.937677	-1.309524
H	2.554094	4.262894	-1.335345
C	3.954552	3.612690	0.147001
H	3.320294	2.815021	0.553634
H	3.837367	4.499043	0.781783
H	4.994558	3.278758	0.238302
C	4.449239	5.101672	-1.832310
H	4.296828	5.994398	-1.215097
H	4.186694	5.357508	-2.864492
H	5.518111	4.860570	-1.809030
C	1.595275	0.257902	-4.270419
H	0.755269	0.958209	-4.238056
C	1.868158	-0.060052	-5.745096
H	2.197338	0.825579	-6.300064
H	0.957815	-0.441232	-6.220960
H	2.639811	-0.829801	-5.856885
C	1.182611	-1.011288	-3.513042
H	1.989140	-1.756245	-3.522540
H	0.296212	-1.468311	-3.972033
H	0.944165	-0.786613	-2.465215
C	-1.621631	4.558295	-0.038297
C	-3.028519	4.480040	0.020293
C	-3.689464	5.117290	1.072418
H	-4.776575	5.086748	1.112392
C	-2.990215	5.773430	2.074394
H	-3.523111	6.271859	2.879929
C	-1.602161	5.749422	2.063871
H	-1.056398	6.220332	2.876744
C	-0.896949	5.138178	1.026108
C	-3.855708	3.674342	-0.975114
H	-3.177946	3.292785	-1.747955
C	-4.958110	4.489946	-1.663080
H	-5.690603	4.860460	-0.937336
H	-5.498088	3.861725	-2.380521
H	-4.565711	5.354154	-2.207184
C	-4.487594	2.469642	-0.260770
H	-3.722769	1.855286	0.226910

H	-5.043862	1.841659	-0.966617
H	-5.189499	2.798379	0.515062
C	0.627891	5.132431	1.048207
H	0.966727	4.249145	0.491303
C	1.199044	5.004731	2.464068
H	1.066762	5.926083	3.042727
H	2.273049	4.796349	2.420128
H	0.722361	4.183515	3.009448
C	1.208509	6.361051	0.336612
H	0.926406	6.387279	-0.721143
H	2.303665	6.356822	0.390687
H	0.850671	7.282755	0.810740
N	3.184254	-1.230544	2.516201
C	3.473637	-2.333233	3.187309
C	3.264436	-3.637007	2.687001
H	3.481136	-4.440603	3.380983
C	3.039725	-4.035799	1.353508
N	2.742368	-3.208672	0.354868
C	4.108872	-2.232460	4.556977
H	5.117165	-1.813322	4.472161
H	4.180137	-3.211938	5.031825
H	3.539316	-1.559634	5.204060
C	3.184004	-5.520434	1.083267
H	2.207013	-5.950548	0.842851
H	3.585825	-6.040533	1.953854
H	3.836145	-5.706741	0.226201
C	3.617778	0.040277	3.016203
C	4.886312	0.529467	2.643498
C	5.253933	1.815604	3.041380
H	6.228832	2.204001	2.752425
C	4.397649	2.609562	3.792384
H	4.696495	3.612525	4.086969
C	3.162793	2.105584	4.177256
H	2.497194	2.720709	4.779834
C	2.756489	0.820807	3.811324
C	5.859789	-0.297910	1.815595
H	5.408913	-1.281593	1.645546
C	6.093421	0.346542	0.446572
H	5.155886	0.472120	-0.105898
H	6.768131	-0.265831	-0.162967
H	6.549538	1.338937	0.550703
C	7.191075	-0.516275	2.544344
H	7.723149	0.429298	2.699203
H	7.844159	-1.171943	1.957239
H	7.044234	-0.977893	3.526543
C	1.410485	0.302756	4.290518
H	1.303970	-0.734649	3.953978
C	1.313903	0.305104	5.821973
H	2.126308	-0.263816	6.287450
H	0.362160	-0.136938	6.135721
H	1.355307	1.325278	6.220873
C	0.252014	1.098226	3.687598
H	0.269038	2.140765	4.032528
H	-0.700168	0.656443	3.993171
H	0.286290	1.095857	2.589708
C	2.783907	-3.715473	-0.986257
C	1.650303	-4.317100	-1.563614
C	1.727704	-4.785462	-2.877782
H	0.852025	-5.245962	-3.330241
C	2.895621	-4.672308	-3.612076
H	2.943570	-5.048330	-4.630701
C	4.005661	-4.061435	-3.040481
H	4.911462	-3.965843	-3.630869
C	3.975598	-3.565098	-1.736355
C	0.329199	-4.454398	-0.822887
H	0.472839	-4.097154	0.203075
C	-0.146976	-5.911501	-0.754529
H	-0.367905	-6.306722	-1.752765
H	-1.067411	-5.987593	-0.163536
H	0.601347	-6.570397	-0.302737
C	-0.741260	-3.572855	-1.474746
H	-0.422181	-2.526880	-1.515016
H	-1.674490	-3.624084	-0.898307
H	-0.968328	-3.900560	-2.496528
C	5.203324	-2.864816	-1.154656
H	4.837585	-1.980152	-0.615763
C	6.179948	-2.384992	-2.232230
H	6.943621	-1.741870	-1.782958
H	6.704420	-3.226303	-2.700394
H	5.674789	-1.814655	-3.015796
C	5.980345	-3.728709	-0.148992
H	6.893955	-3.206355	0.160522
H	5.409328	-3.944771	0.756281
H	6.279720	-4.678340	-0.609100
N	-3.851508	-1.107413	1.847187
C	-5.167981	-1.154376	1.722719
C	-5.851589	-1.018772	0.494515
H	-6.928694	-0.925412	0.575990
C	-5.355499	-1.124315	-0.812529
N	-4.077198	-1.341254	-1.129641
C	-6.051169	-1.360671	2.938420
H	-5.625353	-2.114981	3.605498
H	-7.049418	-1.678176	2.633475
H	-6.148265	-0.437439	3.517545
C	-6.390399	-0.964527	-1.911770
H	-6.305174	0.048274	-2.322762
H	-7.402629	-1.083987	-1.521503
H	-6.241486	-1.662932	-2.736675
C	-3.281022	-1.193750	3.156831
C	-2.573756	-2.359846	3.517073
C	-2.030817	-2.438791	4.800581
H	-1.491303	-3.331762	5.102418
C	-2.172423	-1.394574	5.705637
H	-1.756475	-1.480911	6.706511
C	-2.832518	-0.235354	5.321629
H	-2.908788	0.591322	6.023894
C	-3.383655	-0.103641	4.045996
C	-2.432884	-3.525330	2.544270
H	-2.342442	-3.105849	1.533855
C	-1.172893	-4.362548	2.775848

H	-1.224781	-4.930485	3.711595
H	-0.273141	-3.736791	2.803507
H	-1.056626	-5.090427	1.965287
C	-3.682211	-4.415444	2.545355
H	-3.843491	-4.852954	3.537546
H	-3.568333	-5.236768	1.826920
H	-4.578840	-3.850797	2.270067
C	-4.013481	1.222826	3.633234
H	-4.630569	1.052103	2.745849
C	-4.919970	1.819512	4.716085
H	-5.666739	1.100952	5.071767
H	-5.447819	2.694336	4.321991
H	-4.344153	2.155686	5.585349
C	-2.932942	2.234665	3.231854
H	-2.289435	2.475049	4.086102
H	-3.388844	3.165112	2.879495
H	-2.291374	1.847808	2.431171
C	-3.798510	-1.822699	-2.452300
C	-3.385009	-0.951822	-3.477548
C	-3.109339	-1.482844	-4.741235
H	-2.793003	-0.815572	-5.540615
C	-3.247920	-2.836956	-4.999430
H	-3.038420	-3.230676	-5.990530
C	-3.657899	-3.689359	-3.980847
H	-3.758806	-4.751217	-4.188634
C	-3.929427	-3.211937	-2.699264
C	-3.256200	0.551225	-3.272823
H	-3.464344	0.764399	-2.219325
C	-4.266255	1.331407	-4.124360
H	-4.073886	1.185173	-5.193501
H	-4.191360	2.405996	-3.918404
H	-5.296444	1.016650	-3.930418
C	-1.835331	1.034575	-3.574479
H	-1.107279	0.477077	-2.976051
H	-1.736100	2.110237	-3.369044
H	-1.578584	0.887761	-4.630668
C	-4.360566	-4.187213	-1.603526
H	-4.015467	-3.772402	-0.646772
C	-3.745585	-5.582755	-1.766348
H	-4.226781	-6.141011	-2.577859
H	-3.895500	-6.162550	-0.848500
H	-2.673551	-5.542460	-1.973095
C	-5.887119	-4.342349	-1.513162
H	-6.385555	-3.429143	-1.183648
H	-6.139580	-5.129950	-0.794185
H	-6.300316	-4.631168	-2.487042
H	1.565069	0.002066	0.236662

6b-theta40

SCF (wB97x) = -4439.03063975

Pt	-0.125460	0.016605	0.163408
Mg	0.092862	2.247584	-1.132905
Mg	1.954609	-1.356290	0.861027
Mg	-2.583458	-0.864226	0.234620
H	-1.006180	-1.325767	0.696894
H	-1.110122	1.237576	-0.518104
N	1.339970	2.745454	-2.688467
C	1.019729	3.697553	-3.555345
C	-0.045707	4.604045	-3.386912
H	-0.170809	5.317363	-4.192809
C	-0.825979	4.858122	-2.239659
N	-0.893412	4.065745	-1.173533
C	1.864385	3.892133	-4.798010
H	2.885034	4.180790	-4.527479
H	1.442023	4.662629	-5.444228
H	1.940828	2.957132	-5.361751
C	-1.590074	6.168516	-2.259831
H	-2.666816	6.003469	-2.187740
H	-1.380633	6.726428	-3.172991
H	-1.310582	6.784658	-1.399307
C	2.621257	2.114778	-2.833923
C	3.729244	2.698101	-2.185469
C	4.984938	2.111906	-2.343643
H	5.848654	2.562478	-1.858437
C	5.150361	0.958432	-3.097012
H	6.136627	0.514742	-3.207111
C	4.045427	0.371148	-3.698223
H	4.169916	-0.541817	-4.277655
C	2.773019	0.934543	-3.587293
C	3.599416	3.937677	-1.309524
H	2.554094	4.262894	-1.335345
C	3.954552	3.612690	0.147001
H	3.320294	2.815021	0.553634
H	3.837367	4.499043	0.781783
H	4.994558	3.278758	0.238302
C	4.449239	5.101672	-1.832310
H	4.296828	5.994398	-1.215097
H	4.186694	5.357508	-2.864492
H	5.518111	4.860570	-1.809030
C	1.595275	0.257902	-4.270419
H	0.755269	0.958209	-4.238056
C	1.868158	-0.060052	-5.745096
H	2.197338	0.825579	-6.300064
H	0.957815	-0.441232	-6.220960
H	2.639811	-0.829801	-5.856885
C	1.182611	-1.011288	-3.513042
H	1.989140	-1.756245	-3.522540
H	0.296212	-1.468311	-3.972033
H	0.944165	-0.786613	-2.465215
C	-1.621631	4.558295	-0.038297
C	-3.028519	4.480040	0.020293
C	-3.689464	5.117290	1.072418
H	-4.776575	5.086748	1.112392
C	-2.990215	5.773430	2.074394
H	-3.523111	6.271859	2.879929
C	-1.602161	5.749422	2.063871
H	-1.056398	6.220332	2.876744
C	-0.896949	5.138178	1.026108

C	-3.855708	3.674342	-0.975114
H	-3.177946	3.292785	-1.747955
C	-4.958110	4.489946	-1.663080
H	-5.690603	4.860460	-0.937336
H	-5.498088	3.861725	-2.380521
H	-4.565711	5.354154	-2.207184
C	-4.487594	2.469642	-0.260770
H	-3.722769	1.855286	0.226910
H	-5.043862	1.841659	-0.966617
H	-5.189499	2.798379	0.515062
C	0.627891	5.132431	1.048207
H	0.966727	4.249145	0.491303
C	1.199044	5.004731	2.464068
H	1.066762	5.926083	3.042727
H	2.273049	4.796349	2.420128
H	0.722361	4.183515	3.009448
C	1.208509	6.361051	0.336612
H	0.926406	6.387279	-0.721143
H	2.303665	6.356822	0.390687
H	0.850671	7.282755	0.810740
N	3.184254	-1.230544	2.516201
C	3.473637	-2.333233	3.187309
C	3.264436	-3.637007	2.687001
H	3.481136	-4.440603	3.380983
C	3.039725	-4.035799	1.353508
N	2.742368	-3.208672	0.354868
C	4.108872	-2.232460	4.556977
H	5.117165	-1.813322	4.472161
H	4.180137	-3.211938	5.031825
H	3.539316	-1.559634	5.204060
C	3.184004	-5.520434	1.083267
H	2.207013	-5.950548	0.842851
H	3.585825	-6.040533	1.953854
H	3.836145	-5.706741	0.226201
C	3.617778	0.040277	3.016203
C	4.886312	0.529467	2.643498
C	5.253933	1.815604	3.041380
H	6.228832	2.204001	2.752425
C	4.397649	2.609562	3.792384
H	4.696495	3.612525	4.086969
C	3.162793	2.105584	4.177256
H	2.497194	2.720709	4.779834
C	2.756489	0.820807	3.811324
C	5.859789	-0.297910	1.815595
H	5.408913	-1.281593	1.645546
C	6.093421	0.346542	0.446572
H	5.155886	0.472120	-0.105898
H	6.768131	-0.265831	-0.162967
H	6.549538	1.338937	0.550703
C	7.191075	-0.516275	2.544344
H	7.723149	0.429298	2.699203
H	7.844159	-1.171943	1.957239
H	7.044234	-0.977893	3.526543
C	1.410485	0.302756	4.290518
H	1.303970	-0.734649	3.953978

C	1.313903	0.305104	5.821973
H	2.126308	-0.263816	6.287450
H	0.362160	-0.136938	6.135721
H	1.355307	1.325278	6.220873
C	0.252014	1.098226	3.687598
H	0.269038	2.140765	4.032528
H	-0.700168	0.656443	3.993171
H	0.286290	1.095857	2.589708
C	2.783907	-3.715473	-0.986257
C	1.650303	-4.317100	-1.563614
C	1.727704	-4.785462	-2.877782
H	0.852025	-5.245962	-3.330241
C	2.895621	-4.672308	-3.612076
H	2.943570	-5.048330	-4.630701
C	4.005661	-4.061435	-3.040481
H	4.911462	-3.965843	-3.630869
C	3.975598	-3.565098	-1.736355
C	0.329199	-4.454398	-0.822887
H	0.472839	-4.097154	0.203075
C	-0.146976	-5.911501	-0.754529
H	-0.367905	-6.306722	-1.752765
H	-1.067411	-5.987593	-0.163536
H	0.601347	-6.570397	-0.302737
C	-0.741260	-3.572855	-1.474746
H	-0.422181	-2.526880	-1.515016
H	-1.674490	-3.624084	-0.898307
H	-0.968328	-3.900560	-2.496528
C	5.203324	-2.864816	-1.154656
H	4.837585	-1.980152	-0.615763
C	6.179948	-2.384992	-2.232230
H	6.943621	-1.741870	-1.782958
H	6.704420	-3.226303	-2.700394
H	5.674789	-1.814655	-3.015796
C	5.980345	-3.728709	-0.148992
H	6.893955	-3.206355	0.160522
H	5.409328	-3.944771	0.756281
H	6.279720	-4.678340	-0.609100
N	-3.851508	-1.107413	1.847187
C	-5.167981	-1.154376	1.722719
C	-5.851589	-1.018772	0.494515
H	-6.928694	-0.925412	0.575990
C	-5.355499	-1.124315	-0.812529
N	-4.077198	-1.341254	-1.129641
C	-6.051169	-1.360671	2.938420
H	-5.625353	-2.114981	3.605498
H	-7.049418	-1.678176	2.633475
H	-6.148265	-0.437439	3.517545
C	-6.390399	-0.964527	-1.911770
H	-6.305174	0.048274	-2.322762
H	-7.402629	-1.083987	-1.521503
H	-6.241486	-1.662932	-2.736675
C	-3.281022	-1.193750	3.156831
C	-2.573756	-2.359846	3.517073
C	-2.030817	-2.438791	4.800581
H	-1.491303	-3.331762	5.102418

C	-2.172423	-1.394574	5.705637
H	-1.756475	-1.480911	6.706511
C	-2.832518	-0.235354	5.321629
H	-2.908788	0.591322	6.023894
C	-3.383655	-0.103641	4.045996
C	-2.432884	-3.525330	2.544270
H	-2.342442	-3.105849	1.533855
C	-1.172893	-4.362548	2.775848
H	-1.224781	-4.930485	3.711595
H	-0.273141	-3.736791	2.803507
H	-1.056626	-5.090427	1.965287
C	-3.682211	-4.415444	2.545355
H	-3.843491	-4.852954	3.537546
H	-3.568333	-5.236768	1.826920
H	-4.578840	-3.850797	2.270067
C	-4.013481	1.222826	3.633234
H	-4.630569	1.052103	2.745849
C	-4.919970	1.819512	4.716085
H	-5.666739	1.100952	5.071767
H	-5.447819	2.694336	4.321991
H	-4.344153	2.155686	5.585349
C	-2.932942	2.234665	3.231854
H	-2.289435	2.475049	4.086102
H	-3.388844	3.165112	2.879495
H	-2.291374	1.847808	2.431171
C	-3.798510	-1.822699	-2.452300
C	-3.385009	-0.951822	-3.477548
C	-3.109339	-1.482844	-4.741235
H	-2.793003	-0.815572	-5.540615
C	-3.247920	-2.836956	-4.999430
H	-3.038420	-3.230676	-5.990530
C	-3.657899	-3.689359	-3.980847
H	-3.758806	-4.751217	-4.188634
C	-3.929427	-3.211937	-2.699264
C	-3.256200	0.551225	-3.272823
H	-3.464344	0.764399	-2.219325
C	-4.266255	1.331407	-4.124360
H	-4.073886	1.185173	-5.193501
H	-4.191360	2.405996	-3.918404
H	-5.296444	1.016650	-3.930418
C	-1.835331	1.034575	-3.574479
H	-1.107279	0.477077	-2.976051
H	-1.736100	2.110237	-3.369044
H	-1.578584	0.887761	-4.630668
C	-4.360566	-4.187213	-1.603526
H	-4.015467	-3.772402	-0.646772
C	-3.745585	-5.582755	-1.766348
H	-4.226781	-6.141011	-2.577859
H	-3.895500	-6.162550	-0.848500
H	-2.673551	-5.542460	-1.973095
C	-5.887119	-4.342349	-1.513162
H	-6.385555	-3.429143	-1.183648
H	-6.139580	-5.129950	-0.794185
H	-6.300316	-4.631168	-2.487042
H	1.562401	0.136710	0.176193

6b-theta45

SCF (wB97x) = -4439.04889929

Pt	-0.125460	0.016605	0.163408
Mg	0.092862	2.247584	-1.132905
Mg	1.954609	-1.356290	0.861027
Mg	-2.583458	-0.864226	0.234620
H	-0.878441	-1.382732	0.744023
H	-1.228201	1.159966	-0.470213
N	1.339970	2.745454	-2.688467
C	1.019729	3.697553	-3.555345
C	-0.045707	4.604045	-3.386912
H	-0.170809	5.317363	-4.192809
C	-0.825979	4.858122	-2.239659
N	-0.893412	4.065745	-1.173533
C	1.864385	3.892133	-4.798010
H	2.885034	4.180790	-4.527479
H	1.442023	4.662629	-5.444228
H	1.940828	2.957132	-5.361751
C	-1.590074	6.168516	-2.259831
H	-2.666816	6.003469	-2.187740
H	-1.380633	6.726428	-3.172991
H	-1.310582	6.784658	-1.399307
C	2.621257	2.114778	-2.833923
C	3.729244	2.698101	-2.185469
C	4.984938	2.111906	-2.343643
H	5.848654	2.562478	-1.858437
C	5.150361	0.958432	-3.097012
H	6.136627	0.514742	-3.207111
C	4.045427	0.371148	-3.698223
H	4.169916	-0.541817	-4.277655
C	2.773019	0.934543	-3.587293
C	3.599416	3.937677	-1.309524
H	2.554094	4.262894	-1.335345
C	3.954552	3.612690	0.147001
H	3.320294	2.815021	0.553634
H	3.837367	4.499043	0.781783
H	4.994558	3.278758	0.238302
C	4.449239	5.101672	-1.832310
H	4.296828	5.994398	-1.215097
H	4.186694	5.357508	-2.864492
H	5.518111	4.860570	-1.809030
C	1.595275	0.257902	-4.270419
H	0.755269	0.958209	-4.238056
C	1.868158	-0.060052	-5.745096
H	2.197338	0.825579	-6.300064
H	0.957815	-0.441232	-6.220960
H	2.639811	-0.829801	-5.856885
C	1.182611	-1.011288	-3.513042
H	1.989140	-1.756245	-3.522540
H	0.296212	-1.468311	-3.972033
H	0.944165	-0.786613	-2.465215
C	-1.621631	4.558295	-0.038297
C	-3.028519	4.480040	0.020293

C	-3.689464	5.117290	1.072418
H	-4.776575	5.086748	1.112392
C	-2.990215	5.773430	2.074394
H	-3.523111	6.271859	2.879929
C	-1.602161	5.749422	2.063871
H	-1.056398	6.220332	2.876744
C	-0.896949	5.138178	1.026108
C	-3.855708	3.674342	-0.975114
H	-3.177946	3.292785	-1.747955
C	-4.958110	4.489946	-1.663080
H	-5.690603	4.860460	-0.937336
H	-5.498088	3.861725	-2.380521
H	-4.565711	5.354154	-2.207184
C	-4.487594	2.469642	-0.260770
H	-3.722769	1.855286	0.226910
H	-5.043862	1.841659	-0.966617
H	-5.189499	2.798379	0.515062
C	0.627891	5.132431	1.048207
H	0.966727	4.249145	0.491303
C	1.199044	5.004731	2.464068
H	1.066762	5.926083	3.042727
H	2.273049	4.796349	2.420128
H	0.722361	4.183515	3.009448
C	1.208509	6.361051	0.336612
H	0.926406	6.387279	-0.721143
H	2.303665	6.356822	0.390687
H	0.850671	7.282755	0.810740
N	3.184254	-1.230544	2.516201
C	3.473637	-2.333233	3.187309
C	3.264436	-3.637007	2.687001
H	3.481136	-4.440603	3.380983
C	3.039725	-4.035799	1.353508
N	2.742368	-3.208672	0.354868
C	4.108872	-2.232460	4.556977
H	5.117165	-1.813322	4.472161
H	4.180137	-3.211938	5.031825
H	3.539316	-1.559634	5.204060
C	3.184004	-5.520434	1.083267
H	2.207013	-5.950548	0.842851
H	3.585825	-6.040533	1.953854
H	3.836145	-5.706741	0.226201
C	3.617778	0.040277	3.016203
C	4.886312	0.529467	2.643498
C	5.253933	1.815604	3.041380
H	6.228832	2.204001	2.752425
C	4.397649	2.609562	3.792384
H	4.696495	3.612525	4.086969
C	3.162793	2.105584	4.177256
H	2.497194	2.720709	4.779834
C	2.756489	0.820807	3.811324
C	5.859789	-0.297910	1.815595
H	5.408913	-1.281593	1.645546
C	6.093421	0.346542	0.446572
H	5.155886	0.472120	-0.105898
H	6.768131	-0.265831	-0.162967
H	6.549538	1.338937	0.550703
C	7.191075	-0.516275	2.544344
H	7.723149	0.429298	2.699203
H	7.844159	-1.171943	1.957239
H	7.044234	-0.977893	3.526543
C	1.410485	0.302756	4.290518
H	1.303970	-0.734649	3.953978
C	1.313903	0.305104	5.821973
H	2.126308	-0.263816	6.287450
H	0.362160	-0.136938	6.135721
H	1.355307	1.325278	6.220873
C	0.252014	1.098226	3.687598
H	0.269038	2.140765	4.032528
H	-0.700168	0.656443	3.993171
H	0.286290	1.095857	2.589708
C	2.783907	-3.715473	-0.986257
C	1.650303	-4.317100	-1.563614
C	1.727704	-4.785462	-2.877782
H	0.852025	-5.245962	-3.330241
C	2.895621	-4.672308	-3.612076
H	2.943570	-5.048330	-4.630701
C	4.005661	-4.061435	-3.040481
H	4.911462	-3.965843	-3.630869
C	3.975598	-3.565098	-1.736355
C	0.329199	-4.454398	-0.822887
H	0.472839	-4.097154	0.203075
C	-0.146976	-5.911501	-0.754529
H	-0.367905	-6.306722	-1.752765
H	-1.067411	-5.987593	-0.163536
H	0.601347	-6.570397	-0.302737
C	-0.741260	-3.572855	-1.474746
H	-0.422181	-2.526880	-1.515016
H	-1.674490	-3.624084	-0.898307
H	-0.968328	-3.900560	-2.496528
C	5.203324	-2.864816	-1.154656
H	4.837585	-1.980152	-0.615763
C	6.179948	-2.384992	-2.232230
H	6.943621	-1.741870	-1.782958
H	6.704420	-3.226303	-2.700394
H	5.674789	-1.814655	-3.015796
C	5.980345	-3.728709	-0.148992
H	6.893955	-3.206355	0.160522
H	5.409328	-3.944771	0.756281
H	6.279720	-4.678340	-0.609100
N	-3.851508	-1.107413	1.847187
C	-5.167981	-1.154376	1.722719
C	-5.851589	-1.018772	0.494515
H	-6.928694	-0.925412	0.575990
C	-5.355499	-1.124315	-0.812529
N	-4.077198	-1.341254	-1.129641
C	-6.051169	-1.360671	2.938420
H	-5.625353	-2.114981	3.605498
H	-7.049418	-1.678176	2.633475
H	-6.148265	-0.437439	3.517545
C	-6.390399	-0.964527	-1.911770

H	-6.305174	0.048274	-2.322762
H	-7.402629	-1.083987	-1.521503
H	-6.241486	-1.662932	-2.736675
C	-3.281022	-1.193750	3.156831
C	-2.573756	-2.359846	3.517073
C	-2.030817	-2.438791	4.800581
H	-1.491303	-3.331762	5.102418
C	-2.172423	-1.394574	5.705637
H	-1.756475	-1.480911	6.706511
C	-2.832518	-0.235354	5.321629
H	-2.908788	0.591322	6.023894
C	-3.383655	-0.103641	4.045996
C	-2.432884	-3.525330	2.544270
H	-2.342442	-3.105849	1.533855
C	-1.172893	-4.362548	2.775848
H	-1.224781	-4.930485	3.711595
H	-0.273141	-3.736791	2.803507
H	-1.056626	-5.090427	1.965287
C	-3.682211	-4.415444	2.545355
H	-3.843491	-4.852954	3.537546
H	-3.568333	-5.236768	1.826920
H	-4.578840	-3.850797	2.270067
C	-4.013481	1.222826	3.633234
H	-4.630569	1.052103	2.745849
C	-4.919970	1.819512	4.716085
H	-5.666739	1.100952	5.071767
H	-5.447819	2.694336	4.321991
H	-4.344153	2.155686	5.585349
C	-2.932942	2.234665	3.231854
H	-2.289435	2.475049	4.086102
H	-3.388844	3.165112	2.879495
H	-2.291374	1.847808	2.431171
C	-3.798510	-1.822699	-2.452300
C	-3.385009	-0.951822	-3.477548
C	-3.109339	-1.482844	-4.741235
H	-2.793003	-0.815572	-5.540615
C	-3.247920	-2.836956	-4.999430
H	-3.038420	-3.230676	-5.990530
C	-3.657899	-3.689359	-3.980847
H	-3.758806	-4.751217	-4.188634
C	-3.929427	-3.211937	-2.699264
C	-3.256200	0.551225	-3.272823
H	-3.464344	0.764399	-2.219325
C	-4.266255	1.331407	-4.124360
H	-4.073886	1.185173	-5.193501
H	-4.191360	2.405996	-3.918404
H	-5.296444	1.016650	-3.930418
C	-1.835331	1.034575	-3.574479
H	-1.107279	0.477077	-2.976051
H	-1.736100	2.110237	-3.369044
H	-1.578584	0.887761	-4.630668
C	-4.360566	-4.187213	-1.603526
H	-4.015467	-3.772402	-0.646772
C	-3.745585	-5.582755	-1.766348
H	-4.226781	-6.141011	-2.577859
H	-3.895500	-6.162550	-0.848500
H	-2.673551	-5.542460	-1.973095
C	-5.887119	-4.342349	-1.513162
H	-6.385555	-3.429143	-1.183648
H	-6.139580	-5.129950	-0.794185
H	-6.300316	-4.631168	-2.487042
H	1.546888	0.270440	0.115626

6b-theta50

SCF (wB97x) = -4439.05715290

Pt	-0.125460	0.016605	0.163408
Mg	0.092862	2.247584	-1.132905
Mg	1.954609	-1.356290	0.861027
Mg	-2.583458	-0.864226	0.234620
H	-0.744972	-1.429047	0.786732
H	-1.337887	1.073655	-0.417500
N	1.339970	2.745454	-2.688467
C	1.019729	3.697553	-3.555345
C	-0.045707	4.604045	-3.386912
H	-0.170809	5.317363	-4.192809
C	-0.825979	4.858122	-2.239659
N	-0.893412	4.065745	-1.173533
C	1.864385	3.892133	-4.798010
H	2.885034	4.180790	-4.527479
H	1.442023	4.662629	-5.444228
H	1.940828	2.957132	-5.361751
C	-1.590074	6.168516	-2.259831
H	-2.666816	6.003469	-2.187740
H	-1.380633	6.726428	-3.172991
H	-1.310582	6.784658	-1.399307
C	2.621257	2.114778	-2.833923
C	3.729244	2.698101	-2.185469
C	4.984938	2.111906	-2.343643
H	5.848654	2.562478	-1.858437
C	5.150361	0.958432	-3.097012
H	6.136627	0.514742	-3.207111
C	4.045427	0.371148	-3.698223
H	4.169916	-0.541817	-4.277655
C	2.773019	0.934543	-3.587293
C	3.599416	3.937677	-1.309524
H	2.554094	4.262894	-1.335345
C	3.954552	3.612690	0.147001
H	3.320294	2.815021	0.553634
H	3.837367	4.499043	0.781783
H	4.994558	3.278758	0.238302
C	4.449239	5.101672	-1.832310
H	4.296828	5.994398	-1.215097
H	4.186694	5.357508	-2.864492
H	5.518111	4.860570	-1.809030
C	1.595275	0.257902	-4.270419
H	0.755269	0.958209	-4.238056
C	1.868158	-0.060052	-5.745096
H	2.197338	0.825579	-6.300064

H	0.957815	-0.441232	-6.220960
H	2.639811	-0.829801	-5.856885
C	1.182611	-1.011288	-3.513042
H	1.989140	-1.756245	-3.522540
H	0.296212	-1.468311	-3.972033
H	0.944165	-0.786613	-2.465215
C	-1.621631	4.558295	-0.038297
C	-3.028519	4.480040	0.020293
C	-3.689464	5.117290	1.072418
H	-4.776575	5.086748	1.112392
C	-2.990215	5.773430	2.074394
H	-3.523111	6.271859	2.879929
C	-1.602161	5.749422	2.063871
H	-1.056398	6.220332	2.876744
C	-0.896949	5.138178	1.026108
C	-3.855708	3.674342	-0.975114
H	-3.177946	3.292785	-1.747955
C	-4.958110	4.489946	-1.663080
H	-5.690603	4.860460	-0.937336
H	-5.498088	3.861725	-2.380521
H	-4.565711	5.354154	-2.207184
C	-4.487594	2.469642	-0.260770
H	-3.722769	1.855286	0.226910
H	-5.043862	1.841659	-0.966617
H	-5.189499	2.798379	0.515062
C	0.627891	5.132431	1.048207
H	0.966727	4.249145	0.491303
C	1.199044	5.004731	2.464068
H	1.066762	5.926083	3.042727
H	2.273049	4.796349	2.420128
H	0.722361	4.183515	3.009448
C	1.208509	6.361051	0.336612
H	0.926406	6.387279	-0.721143
H	2.303665	6.356822	0.390687
H	0.850671	7.282755	0.810740
N	3.184254	-1.230544	2.516201
C	3.473637	-2.333233	3.187309
C	3.264436	-3.637007	2.687001
H	3.481136	-4.440603	3.380983
C	3.039725	-4.035799	1.353508
N	2.742368	-3.208672	0.354868
C	4.108872	-2.232460	4.556977
H	5.117165	-1.813322	4.472161
H	4.180137	-3.211938	5.031825
H	3.539316	-1.559634	5.204060
C	3.184004	-5.520434	1.083267
H	2.207013	-5.950548	0.842851
H	3.585825	-6.040533	1.953854
H	3.836145	-5.706741	0.226201
C	3.617778	0.040277	3.016203
C	4.886312	0.529467	2.643498
C	5.253933	1.815604	3.041380
H	6.228832	2.204001	2.752425
C	4.397649	2.609562	3.792384
H	4.696495	3.612525	4.086969

C	3.162793	2.105584	4.177256
H	2.497194	2.720709	4.779834
C	2.756489	0.820807	3.811324
C	5.859789	-0.297910	1.815595
H	5.408913	-1.281593	1.645546
C	6.093421	0.346542	0.446572
H	5.155886	0.472120	-0.105898
H	6.768131	-0.265831	-0.162967
H	6.549538	1.338937	0.550703
C	7.191075	-0.516275	2.544344
H	7.723149	0.429298	2.699203
H	7.844159	-1.171943	1.957239
H	7.044234	-0.977893	3.526543
C	1.410485	0.302756	4.290518
H	1.303970	-0.734649	3.953978
C	1.313903	0.305104	5.821973
H	2.126308	-0.263816	6.287450
H	0.362160	-0.136938	6.135721
H	1.355307	1.325278	6.220873
C	0.252014	1.098226	3.687598
H	0.269038	2.140765	4.032528
H	-0.700168	0.656443	3.993171
H	0.286290	1.095857	2.589708
C	2.783907	-3.715473	-0.986257
C	1.650303	-4.317100	-1.563614
C	1.727704	-4.785462	-2.877782
H	0.852025	-5.245962	-3.330241
C	2.895621	-4.672308	-3.612076
H	2.943570	-5.048330	-4.630701
C	4.005661	-4.061435	-3.040481
H	4.911462	-3.965843	-3.630869
C	3.975598	-3.565098	-1.736355
C	0.329199	-4.454398	-0.822887
H	0.472839	-4.097154	0.203075
C	-0.146976	-5.911501	-0.754529
H	-0.367905	-6.306722	-1.752765
H	-1.067411	-5.987593	-0.163536
H	0.601347	-6.570397	-0.302737
C	-0.741260	-3.572855	-1.474746
H	-0.422181	-2.526880	-1.515016
H	-1.674490	-3.624084	-0.898307
H	-0.968328	-3.900560	-2.496528
C	5.203324	-2.864816	-1.154656
H	4.837585	-1.980152	-0.615763
C	6.179948	-2.384992	-2.232230
H	6.943621	-1.741870	-1.782958
H	6.704420	-3.226303	-2.700394
H	5.674789	-1.814655	-3.015796
C	5.980345	-3.728709	-0.148992
H	6.893955	-3.206355	0.160522
H	5.409328	-3.944771	0.756281
H	6.279720	-4.678340	-0.609100
N	-3.851508	-1.107413	1.847187
C	-5.167981	-1.154376	1.722719
C	-5.851589	-1.018772	0.494515

H	-6.928694	-0.925412	0.575990
C	-5.355499	-1.124315	-0.812529
N	-4.077198	-1.341254	-1.129641
C	-6.051169	-1.360671	2.938420
H	-5.625353	-2.114981	3.605498
H	-7.049418	-1.678176	2.633475
H	-6.148265	-0.437439	3.517545
C	-6.390399	-0.964527	-1.911770
H	-6.305174	0.048274	-2.322762
H	-7.402629	-1.083987	-1.521503
H	-6.241486	-1.662932	-2.736675
C	-3.281022	-1.193750	3.156831
C	-2.573756	-2.359846	3.517073
C	-2.030817	-2.438791	4.800581
H	-1.491303	-3.331762	5.102418
C	-2.172423	-1.394574	5.705637
H	-1.756475	-1.480911	6.706511
C	-2.832518	-0.235354	5.321629
H	-2.908788	0.591322	6.023894
C	-3.383655	-0.103641	4.045996
C	-2.432884	-3.525330	2.544270
H	-2.342442	-3.105849	1.533855
C	-1.172893	-4.362548	2.775848
H	-1.224781	-4.930485	3.711595
H	-0.273141	-3.736791	2.803507
H	-1.056626	-5.090427	1.965287
C	-3.682211	-4.415444	2.545355
H	-3.843491	-4.852954	3.537546
H	-3.568333	-5.236768	1.826920
H	-4.578840	-3.850797	2.270067
C	-4.013481	1.222826	3.633234
H	-4.630569	1.052103	2.745849
C	-4.919970	1.819512	4.716085
H	-5.666739	1.100952	5.071767
H	-5.447819	2.694336	4.321991
H	-4.344153	2.155686	5.585349
C	-2.932942	2.234665	3.231854
H	-2.289435	2.475049	4.086102
H	-3.388844	3.165112	2.879495
H	-2.291374	1.847808	2.431171
C	-3.798510	-1.822699	-2.452300
C	-3.385009	-0.951822	-3.477548
C	-3.109339	-1.482844	-4.741235
H	-2.793003	-0.815572	-5.540615
C	-3.247920	-2.836956	-4.999430
H	-3.038420	-3.230676	-5.990530
C	-3.657899	-3.689359	-3.980847
H	-3.758806	-4.751217	-4.188634
C	-3.929427	-3.211937	-2.699264
C	-3.256200	0.551225	-3.272823
H	-3.464344	0.764399	-2.219325
C	-4.266255	1.331407	-4.124360
H	-4.073886	1.185173	-5.193501
H	-4.191360	2.405996	-3.918404
H	-5.296444	1.016650	-3.930418
C	-1.835331	1.034575	-3.574479
H	-1.107279	0.477077	-2.976051
H	-1.736100	2.110237	-3.369044
H	-1.578584	0.887761	-4.630668
C	-4.360566	-4.187213	-1.603526
H	-4.015467	-3.772402	-0.646772
C	-3.745585	-5.582755	-1.766348
H	-4.226781	-6.141011	-2.577859
H	-3.895500	-6.162550	-0.848500
H	-2.673551	-5.542460	-1.973095
C	-5.887119	-4.342349	-1.513162
H	-6.385555	-3.429143	-1.183648
H	-6.139580	-5.129950	-0.794185
H	-6.300316	-4.631168	-2.487042
H	1.518648	0.402238	0.055422

6b-theta55

SCF (wB97x) = -4439.06020124

Pt	-0.125460	0.016605	0.163408
Mg	0.092862	2.247584	-1.132905
Mg	1.954609	-1.356290	0.861027
Mg	-2.583458	-0.864226	0.234620
H	-0.606787	-1.464360	0.824697
H	-1.438346	0.979298	-0.360367
N	1.339970	2.745454	-2.688467
C	1.019729	3.697553	-3.555345
C	-0.045707	4.604045	-3.386912
H	-0.170809	5.317363	-4.192809
C	-0.825979	4.858122	-2.239659
N	-0.893412	4.065745	-1.173533
C	1.864385	3.892133	-4.798010
H	2.885034	4.180790	-4.527479
H	1.442023	4.662629	-5.444228
H	1.940828	2.957132	-5.361751
C	-1.590074	6.168516	-2.259831
H	-2.666816	6.003469	-2.187740
H	-1.380633	6.726428	-3.172991
H	-1.310582	6.784658	-1.399307
C	2.621257	2.114778	-2.833923
C	3.729244	2.698101	-2.185469
C	4.984938	2.111906	-2.343643
H	5.848654	2.562478	-1.858437
C	5.150361	0.958432	-3.097012
H	6.136627	0.514742	-3.207111
C	4.045427	0.371148	-3.698223
H	4.169916	-0.541817	-4.277655
C	2.773019	0.934543	-3.587293
C	3.599416	3.937677	-1.309524
H	2.554094	4.262894	-1.335345
C	3.954552	3.612690	0.147001
H	3.320294	2.815021	0.553634
H	3.837367	4.499043	0.781783
H	4.994558	3.278758	0.238302

C	4.449239	5.101672	-1.832310
H	4.296828	5.994398	-1.215097
H	4.186694	5.357508	-2.864492
H	5.518111	4.860570	-1.809030
C	1.595275	0.257902	-4.270419
H	0.755269	0.958209	-4.238056
C	1.868158	-0.060052	-5.745096
H	2.197338	0.825579	-6.300064
H	0.957815	-0.441232	-6.220960
H	2.639811	-0.829801	-5.856885
C	1.182611	-1.011288	-3.513042
H	1.989140	-1.756245	-3.522540
H	0.296212	-1.468311	-3.972033
H	0.944165	-0.786613	-2.465215
C	-1.621631	4.558295	-0.038297
C	-3.028519	4.480040	0.020293
C	-3.689464	5.117290	1.072418
H	-4.776575	5.086748	1.112392
C	-2.990215	5.773430	2.074394
H	-3.523111	6.271859	2.879929
C	-1.602161	5.749422	2.063871
H	-1.056398	6.220332	2.876744
C	-0.896949	5.138178	1.026108
C	-3.855708	3.674342	-0.975114
H	-3.177946	3.292785	-1.747955
C	-4.958110	4.489946	-1.663080
H	-5.690603	4.860460	-0.937336
H	-5.498088	3.861725	-2.380521
H	-4.565711	5.354154	-2.207184
C	-4.487594	2.469642	-0.260770
H	-3.722769	1.855286	0.226910
H	-5.043862	1.841659	-0.966617
H	-5.189499	2.798379	0.515062
C	0.627891	5.132431	1.048207
H	0.966727	4.249145	0.491303
C	1.199044	5.004731	2.464068
H	1.066762	5.926083	3.042727
H	2.273049	4.796349	2.420128
H	0.722361	4.183515	3.009448
C	1.208509	6.361051	0.336612
H	0.926406	6.387279	-0.721143
H	2.303665	6.356822	0.390687
H	0.850671	7.282755	0.810740
N	3.184254	-1.230544	2.516201
C	3.473637	-2.333233	3.187309
C	3.264436	-3.637007	2.687001
H	3.481136	-4.440603	3.380983
C	3.039725	-4.035799	1.353508
N	2.742368	-3.208672	0.354868
C	4.108872	-2.232460	4.556977
H	5.117165	-1.813322	4.472161
H	4.180137	-3.211938	5.031825
H	3.539316	-1.559634	5.204060
C	3.184004	-5.520434	1.083267
H	2.207013	-5.950548	0.842851
H	3.585825	-6.040533	1.953854
H	3.836145	-5.706741	0.226201
C	3.617778	0.040277	3.016203
C	4.886312	0.529467	2.643498
C	5.253933	1.815604	3.041380
H	6.228832	2.204001	2.752425
C	4.397649	2.609562	3.792384
H	4.696495	3.612525	4.086969
C	3.162793	2.105584	4.177256
H	2.497194	2.720709	4.779834
C	2.756489	0.820807	3.811324
C	5.859789	-0.297910	1.815595
H	5.408913	-1.281593	1.645546
C	6.093421	0.346542	0.446572
H	5.155886	0.472120	-0.105898
H	6.768131	-0.265831	-0.162967
H	6.549538	1.338937	0.550703
C	7.191075	-0.516275	2.544344
H	7.723149	0.429298	2.699203
H	7.844159	-1.171943	1.957239
H	7.044234	-0.977893	3.526543
C	1.410485	0.302756	4.290518
H	1.303970	-0.734649	3.953978
C	1.313903	0.305104	5.821973
H	2.126308	-0.263816	6.287450
H	0.362160	-0.136938	6.135721
H	1.355307	1.325278	6.220873
C	0.252014	1.098226	3.687598
H	0.269038	2.140765	4.032528
H	-0.700168	0.656443	3.993171
H	0.286290	1.095857	2.589708
C	2.783907	-3.715473	-0.986257
C	1.650303	-4.317100	-1.563614
C	1.727704	-4.785462	-2.877782
H	0.852025	-5.245962	-3.330241
C	2.895621	-4.672308	-3.612076
H	2.943570	-5.048330	-4.630701
C	4.005661	-4.061435	-3.040481
H	4.911462	-3.965843	-3.630869
C	3.975598	-3.565098	-1.736355
C	0.329199	-4.454398	-0.822887
H	0.472839	-4.097154	0.203075
C	-0.146976	-5.911501	-0.754529
H	-0.367905	-6.306722	-1.752765
H	-1.067411	-5.987593	-0.163536
H	0.601347	-6.570397	-0.302737
C	-0.741260	-3.572855	-1.474746
H	-0.422181	-2.526880	-1.515016
H	-1.674490	-3.624084	-0.898307
H	-0.968328	-3.900560	-2.496528
C	5.203324	-2.864816	-1.154656
H	4.837585	-1.980152	-0.615763
C	6.179948	-2.384992	-2.232230
H	6.943621	-1.741870	-1.782958
H	6.704420	-3.226303	-2.700394

H	5.674789	-1.814655	-3.015796
C	5.980345	-3.728709	-0.148992
H	6.893955	-3.206355	0.160522
H	5.409328	-3.944771	0.756281
H	6.279720	-4.678340	-0.609100
N	-3.851508	-1.107413	1.847187
C	-5.167981	-1.154376	1.722719
C	-5.851589	-1.018772	0.494515
H	-6.928694	-0.925412	0.575990
C	-5.355499	-1.124315	-0.812529
N	-4.077198	-1.341254	-1.129641
C	-6.051169	-1.360671	2.938420
H	-5.625353	-2.114981	3.605498
H	-7.049418	-1.678176	2.633475
H	-6.148265	-0.437439	3.517545
C	-6.390399	-0.964527	-1.911770
H	-6.305174	0.048274	-2.322762
H	-7.402629	-1.083987	-1.521503
H	-6.241486	-1.662932	-2.736675
C	-3.281022	-1.193750	3.156831
C	-2.573756	-2.359846	3.517073
C	-2.030817	-2.438791	4.800581
H	-1.491303	-3.331762	5.102418
C	-2.172423	-1.394574	5.705637
H	-1.756475	-1.480911	6.706511
C	-2.832518	-0.235354	5.321629
H	-2.908788	0.591322	6.023894
C	-3.383655	-0.103641	4.045996
C	-2.432884	-3.525330	2.544270
H	-2.342442	-3.105849	1.533855
C	-1.172893	-4.362548	2.775848
H	-1.224781	-4.930485	3.711595
H	-0.273141	-3.736791	2.803507
H	-1.056626	-5.090427	1.965287
C	-3.682211	-4.415444	2.545355
H	-3.843491	-4.852954	3.537546
H	-3.568333	-5.236768	1.826920
H	-4.578840	-3.850797	2.270067
C	-4.013481	1.222826	3.633234
H	-4.630569	1.052103	2.745849
C	-4.919970	1.819512	4.716085
H	-5.666739	1.100952	5.071767
H	-5.447819	2.694336	4.321991
H	-4.344153	2.155686	5.585349
C	-2.932942	2.234665	3.231854
H	-2.289435	2.475049	4.086102
H	-3.388844	3.165112	2.879495
H	-2.291374	1.847808	2.431171
C	-3.798510	-1.822699	-2.452300
C	-3.385009	-0.951822	-3.477548
C	-3.109339	-1.482844	-4.741235
H	-2.793003	-0.815572	-5.540615
C	-3.247920	-2.836956	-4.999430
H	-3.038420	-3.230676	-5.990530
C	-3.657899	-3.689359	-3.980847
H	-3.758806	-4.751217	-4.188634
C	-3.929427	-3.211937	-2.699264
C	-3.256200	0.551225	-3.272823
H	-3.464344	0.764399	-2.219325
C	-4.266255	1.331407	-4.124360
H	-4.073886	1.185173	-5.193501
H	-4.191360	2.405996	-3.918404
H	-5.296444	1.016650	-3.930418
C	-1.835331	1.034575	-3.574479
H	-1.107279	0.477077	-2.976051
H	-1.736100	2.110237	-3.369044
H	-1.578584	0.887761	-4.630668
C	-4.360566	-4.187213	-1.603526
H	-4.015467	-3.772402	-0.646772
C	-3.745585	-5.582755	-1.766348
H	-4.226781	-6.141011	-2.577859
H	-3.895500	-6.162550	-0.848500
H	-2.673551	-5.542460	-1.973095
C	-5.887119	-4.342349	-1.513162
H	-6.385555	-3.429143	-1.183648
H	-6.139580	-5.129950	-0.794185
H	-6.300316	-4.631168	-2.487042
H	1.477895	0.531101	-0.003959

6b-theta60

SCF (wB97x) = -4439.06062129

Pt	-0.125460	0.016605	0.163408
Mg	0.092862	2.247584	-1.132905
Mg	1.954609	-1.356290	0.861027
Mg	-2.583458	-0.864226	0.234620
H	-0.464939	-1.488402	0.857630
H	-1.528813	0.877615	-0.299246
N	1.339970	2.745454	-2.688467
C	1.019729	3.697553	-3.555345
C	-0.045707	4.604045	-3.386912
H	-0.170809	5.317363	-4.192809
C	-0.825979	4.858122	-2.239659
N	-0.893412	4.065745	-1.173533
C	1.864385	3.892133	-4.798010
H	2.885034	4.180790	-4.527479
H	1.442023	4.662629	-5.444228
H	1.940828	2.957132	-5.361751
C	-1.590074	6.168516	-2.259831
H	-2.666816	6.003469	-2.187740
H	-1.380633	6.726428	-3.172991
H	-1.310582	6.784658	-1.399307
C	2.621257	2.114778	-2.833923
C	3.729244	2.698101	-2.185469
C	4.984938	2.111906	-2.343643
H	5.848654	2.562478	-1.858437
C	5.150361	0.958432	-3.097012
H	6.136627	0.514742	-3.207111
C	4.045427	0.371148	-3.698223

H	4.169916	-0.541817	-4.277655
C	2.773019	0.934543	-3.587293
C	3.599416	3.937677	-1.309524
H	2.554094	4.262894	-1.335345
C	3.954552	3.612690	0.147001
H	3.320294	2.815021	0.553634
H	3.837367	4.499043	0.781783
H	4.994558	3.278758	0.238302
C	4.449239	5.101672	-1.832310
H	4.296828	5.994398	-1.215097
H	4.186694	5.357508	-2.864492
H	5.518111	4.860570	-1.809030
C	1.595275	0.257902	-4.270419
H	0.755269	0.958209	-4.238056
C	1.868158	-0.060052	-5.745096
H	2.197338	0.825579	-6.300064
H	0.957815	-0.441232	-6.220960
H	2.639811	-0.829801	-5.856885
C	1.182611	-1.011288	-3.513042
H	1.989140	-1.756245	-3.522540
H	0.296212	-1.468311	-3.972033
H	0.944165	-0.786613	-2.465215
C	-1.621631	4.558295	-0.038297
C	-3.028519	4.480040	0.020293
C	-3.689464	5.117290	1.072418
H	-4.776575	5.086748	1.112392
C	-2.990215	5.773430	2.074394
H	-3.523111	6.271859	2.879929
C	-1.602161	5.749422	2.063871
H	-1.056398	6.220332	2.876744
C	-0.896949	5.138178	1.026108
C	-3.855708	3.674342	-0.975114
H	-3.177946	3.292785	-1.747955
C	-4.958110	4.489946	-1.663080
H	-5.690603	4.860460	-0.937336
H	-5.498088	3.861725	-2.380521
H	-4.565711	5.354154	-2.207184
C	-4.487594	2.469642	-0.260770
H	-3.722769	1.855286	0.226910
H	-5.043862	1.841659	-0.966617
H	-5.189499	2.798379	0.515062
C	0.627891	5.132431	1.048207
H	0.966727	4.249145	0.491303
C	1.199044	5.004731	2.464068
H	1.066762	5.926083	3.042727
H	2.273049	4.796349	2.420128
H	0.722361	4.183515	3.009448
C	1.208509	6.361051	0.336612
H	0.926406	6.387279	-0.721143
H	2.303665	6.356822	0.390687
H	0.850671	7.282755	0.810740
N	3.184254	-1.230544	2.516201
C	3.473637	-2.333233	3.187309
C	3.264436	-3.637007	2.687001
H	3.481136	-4.440603	3.380983
C	3.039725	-4.035799	1.353508
N	2.742368	-3.208672	0.354868
C	4.108872	-2.232460	4.556977
H	5.117165	-1.813322	4.472161
H	4.180137	-3.211938	5.031825
H	3.539316	-1.559634	5.204060
C	3.184004	-5.520434	1.083267
H	2.207013	-5.950548	0.842851
H	3.585825	-6.040533	1.953854
H	3.836145	-5.706741	0.226201
C	3.617778	0.040277	3.016203
C	4.886312	0.529467	2.643498
C	5.253933	1.815604	3.041380
H	6.228832	2.204001	2.752425
C	4.397649	2.609562	3.792384
H	4.696495	3.612525	4.086969
C	3.162793	2.105584	4.177256
H	2.497194	2.720709	4.779834
C	2.756489	0.820807	3.811324
C	5.859789	-0.297910	1.815595
H	5.408913	-1.281593	1.645546
C	6.093421	0.346542	0.446572
H	5.155886	0.472120	-0.105898
H	6.768131	-0.265831	-0.162967
H	6.549538	1.338937	0.550703
C	7.191075	-0.516275	2.544344
H	7.723149	0.429298	2.699203
H	7.844159	-1.171943	1.957239
H	7.044234	-0.977893	3.526543
C	1.410485	0.302756	4.290518
H	1.303970	-0.734649	3.953978
C	1.313903	0.305104	5.821973
H	2.126308	-0.263816	6.287450
H	0.362160	-0.136938	6.135721
H	1.355307	1.325278	6.220873
C	0.252014	1.098226	3.687598
H	0.269038	2.140765	4.032528
H	-0.700168	0.656443	3.993171
H	0.286290	1.095857	2.589708
C	2.783907	-3.715473	-0.986257
C	1.650303	-4.317100	-1.563614
C	1.727704	-4.785462	-2.877782
H	0.852025	-5.245962	-3.330241
C	2.895621	-4.672308	-3.612076
H	2.943570	-5.048330	-4.630701
C	4.005661	-4.061435	-3.040481
H	4.911462	-3.965843	-3.630869
C	3.975598	-3.565098	-1.736355
C	0.329199	-4.454398	-0.822887
H	0.472839	-4.097154	0.203075
C	-0.146976	-5.911501	-0.754529
H	-0.367905	-6.306722	-1.752765
H	-1.067411	-5.987593	-0.163536
H	0.601347	-6.570397	-0.302737
C	-0.741260	-3.572855	-1.474746

H	-0.422181	-2.526880	-1.515016
H	-1.674490	-3.624084	-0.898307
H	-0.968328	-3.900560	-2.496528
C	5.203324	-2.864816	-1.154656
H	4.837585	-1.980152	-0.615763
C	6.179948	-2.384992	-2.232230
H	6.943621	-1.741870	-1.782958
H	6.704420	-3.226303	-2.700394
H	5.674789	-1.814655	-3.015796
C	5.980345	-3.728709	-0.148992
H	6.893955	-3.206355	0.160522
H	5.409328	-3.944771	0.756281
H	6.279720	-4.678340	-0.609100
N	-3.851508	-1.107413	1.847187
C	-5.167981	-1.154376	1.722719
C	-5.851589	-1.018772	0.494515
H	-6.928694	-0.925412	0.575990
C	-5.355499	-1.124315	-0.812529
N	-4.077198	-1.341254	-1.129641
C	-6.051169	-1.360671	2.938420
H	-5.625353	-2.114981	3.605498
H	-7.049418	-1.678176	2.633475
H	-6.148265	-0.437439	3.517545
C	-6.390399	-0.964527	-1.911770
H	-6.305174	0.048274	-2.322762
H	-7.402629	-1.083987	-1.521503
H	-6.241486	-1.662932	-2.736675
C	-3.281022	-1.193750	3.156831
C	-2.573756	-2.359846	3.517073
C	-2.030817	-2.438791	4.800581
H	-1.491303	-3.331762	5.102418
C	-2.172423	-1.394574	5.705637
H	-1.756475	-1.480911	6.706511
C	-2.832518	-0.235354	5.321629
H	-2.908788	0.591322	6.023894
C	-3.383655	-0.103641	4.045996
C	-2.432884	-3.525330	2.544270
H	-2.342442	-3.105849	1.533855
C	-1.172893	-4.362548	2.775848
H	-1.224781	-4.930485	3.711595
H	-0.273141	-3.736791	2.803507
H	-1.056626	-5.090427	1.965287
C	-3.682211	-4.415444	2.545355
H	-3.843491	-4.852954	3.537546
H	-3.568333	-5.236768	1.826920
H	-4.578840	-3.850797	2.270067
C	-4.013481	1.222826	3.633234
H	-4.630569	1.052103	2.745849
C	-4.919970	1.819512	4.716085
H	-5.666739	1.100952	5.071767
H	-5.447819	2.694336	4.321991
H	-4.344153	2.155686	5.585349
C	-2.932942	2.234665	3.231854
H	-2.289435	2.475049	4.086102
H	-3.388844	3.165112	2.879495
H	-2.291374	1.847808	2.431171
C	-3.798510	-1.822699	-2.452300
C	-3.385009	-0.951822	-3.477548
C	-3.109339	-1.482844	-4.741235
H	-2.793003	-0.815572	-5.540615
C	-3.247920	-2.836956	-4.999430
H	-3.038420	-3.230676	-5.990530
C	-3.657899	-3.689359	-3.980847
H	-3.758806	-4.751217	-4.188634
C	-3.929427	-3.211937	-2.699264
C	-3.256200	0.551225	-3.272823
H	-3.464344	0.764399	-2.219325
C	-4.266255	1.331407	-4.124360
H	-4.073886	1.185173	-5.193501
H	-4.191360	2.405996	-3.918404
H	-5.296444	1.016650	-3.930418
C	-1.835331	1.034575	-3.574479
H	-1.107279	0.477077	-2.976051
H	-1.736100	2.110237	-3.369044
H	-1.578584	0.887761	-4.630668
C	-4.360566	-4.187213	-1.603526
H	-4.015467	-3.772402	-0.646772
C	-3.745585	-5.582755	-1.766348
H	-4.226781	-6.141011	-2.577859
H	-3.895500	-6.162550	-0.848500
H	-2.673551	-5.542460	-1.973095
C	-5.887119	-4.342349	-1.513162
H	-6.385555	-3.429143	-1.183648
H	-6.139580	-5.129950	-0.794185
H	-6.300316	-4.631168	-2.487042
H	1.424939	0.656049	-0.062067

model-2a

SCF (wB97x) = -1092.13181572
E(SCF)+ZPE(0 K)= -1092.027431
H(298 K)= -1092.016808
G(298 K)= -1092.063690
Lowest Frequency = 46.2356cm-1

Pd	0.890193	-0.361211	-0.076088
Mg	-1.211436	0.091100	-1.193299
H	0.716075	-0.420657	-1.689097
P	3.171866	-0.886909	-0.024852
C	3.583524	-1.235708	1.736444
H	3.305893	-2.275730	1.919002
H	4.655170	-1.153596	1.908936
C	2.809513	-0.305365	2.674310
H	3.165752	0.721396	2.568664
H	2.958181	-0.591744	3.713905
P	1.015527	-0.279716	2.251728
H	4.067616	0.137892	-0.370989
H	3.767484	-1.951463	-0.715930
H	0.507949	-1.384658	2.954993
H	0.518612	0.748939	3.065091

model-4

SCF (wB97x) = -6340.89029670
E(SCF)+ZPE(0 K)= -6339.450671
H(298 K)= -6339.371038
G(298 K)= -6339.566254
Lowest Frequency = 24.5447cm-1

Pd	-6.399172	6.273306	9.641851
Zn	-8.432865	4.897926	9.561039
Zn	-6.750411	7.276264	7.426255
N	-7.065046	7.084765	5.460091
N	-6.957843	9.289592	7.441012
C	-7.518726	9.947961	6.440039
C	-7.859977	9.362748	5.205135
H	-8.302281	10.034716	4.478064
C	-6.612148	5.898658	4.794687
C	-7.467269	4.783216	4.687521
C	-8.880920	4.800645	5.255157
H	-8.916936	5.588407	6.016227
C	-7.661405	7.918200	3.217010
H	-7.923238	6.900678	2.922493
H	-8.405930	8.612427	2.819286
H	-6.699894	8.154281	2.744137
C	-5.306395	5.860847	4.265054
C	-9.267690	3.484264	5.934979
H	-8.546719	3.199331	6.707950
H	-10.246567	3.580858	6.418300
H	-9.342383	2.657070	5.218329
C	-6.418711	10.013912	8.552188
C	-8.703916	10.017701	9.724670
H	-8.996069	9.561593	8.772422
C	-5.731678	3.615255	3.453279
H	-5.393838	2.728816	2.922104
C	-4.332433	7.020956	4.427199
H	-4.887858	7.884823	4.809336
C	-7.802876	11.430235	6.576837
H	-6.926882	11.972658	6.944549
H	-8.111160	11.861031	5.621867
H	-8.606948	11.589279	7.303497
C	-4.887706	4.707647	3.599088
H	-3.881896	4.664514	3.186337
C	-7.008010	3.658172	3.999586
H	-7.662894	2.797108	3.887843
C	-7.229339	10.382444	9.641677
C	-3.252928	6.670227	5.460402
H	-2.683337	5.786811	5.145355
H	-2.547236	7.502007	5.577685
H	-3.693451	6.452529	6.440920
C	-5.032761	10.300572	8.552628
C	-8.932828	8.974587	10.824346
H	-8.673451	9.384433	11.810535
H	-9.984509	8.668467	10.850905
H	-8.319083	8.083032	10.653031
C	-2.670084	9.665552	7.813334
H	-2.575941	9.005341	8.682407
H	-2.109287	9.215407	6.986752
H	-2.180708	10.617953	8.050596
C	-9.914909	5.146924	4.174559
H	-9.861429	4.431317	3.344284
H	-10.929361	5.103224	4.589883
H	-9.764219	6.151233	3.764897
C	-4.132289	9.860768	7.400428
H	-4.502752	8.886861	7.056484
C	-4.499235	11.000065	9.635681
H	-3.439770	11.240289	9.651532
C	-9.607589	11.234370	9.962287
H	-9.456524	12.019627	9.212647
H	-10.659599	10.926168	9.930651
H	-9.425625	11.680806	10.947435
C	-6.644629	11.072943	10.707345
H	-7.263587	11.365594	11.553330
C	-5.295357	11.391259	10.706851
H	-4.859685	11.937672	11.539890
C	-3.689531	7.441128	3.099954
H	-4.444329	7.657269	2.335187
H	-3.082648	8.342901	3.243537
H	-3.028527	6.661130	2.703906
C	-4.198371	10.825367	6.207407
H	-3.901820	11.837125	6.512078
H	-3.511714	10.496317	5.416769
H	-5.200313	10.876207	5.771491
C	-7.531022	8.085263	4.719581
H	-5.390567	6.713295	8.372147
H	-7.539357	5.775713	10.777974
N	-8.136198	2.892435	9.609901
N	-10.421011	4.806846	9.478846
C	-11.061547	3.659800	9.253291
C	-10.446214	2.408672	9.098216
H	-11.115907	1.585598	8.873545
C	-9.109624	2.035603	9.363954
C	-12.575146	3.667902	9.165899
H	-12.962828	2.662047	8.993588
H	-13.017576	4.061841	10.086653
H	-12.912046	4.317661	8.351444
C	-8.856494	0.542227	9.429781
H	-9.062314	0.176828	10.443103
H	-9.527505	0.014992	8.746222
H	-7.822770	0.285146	9.189946
C	-6.882717	2.425888	10.123441
C	-6.781708	2.063097	11.482476
C	-5.746373	2.403681	9.288227
C	-5.541818	1.651985	11.976871
C	-4.531519	1.976208	9.826843
C	-4.422454	1.598799	11.159182
H	-5.452454	1.369545	13.024003
H	-3.651182	1.938659	9.190475
H	-3.466802	1.267248	11.558187
C	-5.834491	2.781973	7.817487
H	-6.655961	3.499575	7.712892

C	-7.960900	2.146897	12.444087
H	-8.870916	2.339763	11.865981
C	-6.161371	1.552343	6.959335
H	-6.196423	1.826807	5.898879
H	-5.393273	0.778896	7.088584
H	-7.129747	1.114322	7.227033
C	-4.575373	3.476399	7.292803
H	-3.720964	2.790389	7.229052
H	-4.761551	3.859333	6.282945
H	-4.298979	4.323470	7.931665
C	-7.778378	3.324461	13.409912
H	-6.874730	3.194679	14.019218
H	-7.687342	4.271623	12.864271
H	-8.635199	3.401024	14.091423
C	-8.176784	0.844382	13.224861
H	-8.263455	-0.019745	12.556526
H	-7.348810	0.645739	13.915538
H	-9.093680	0.907805	13.822344
C	-11.192394	5.957509	9.856933
C	-11.709657	6.836799	8.884836
C	-11.409363	6.208013	11.229171
C	-12.465753	7.933973	9.304190
C	-12.199937	7.297786	11.597489
C	-12.731210	8.158968	10.647445
H	-12.866441	8.618973	8.559639
H	-12.392266	7.483610	12.652175
H	-13.339447	9.006817	10.952908
C	-10.805102	5.341467	12.327417
H	-10.126429	4.620655	11.857964
C	-9.978032	6.190198	13.302107
H	-9.527123	5.558163	14.075317
H	-9.172627	6.715851	12.775143
H	-10.596196	6.940719	13.808692
C	-11.879198	4.539511	13.072633
H	-11.420580	3.904843	13.840936
H	-12.596810	5.203622	13.570145
H	-12.439768	3.890132	12.390183
C	-11.464525	6.647617	7.395067
H	-10.970345	5.679316	7.255232
C	-10.524397	7.736275	6.864270
H	-9.561203	7.719912	7.388609
H	-10.329390	7.605500	5.793607
H	-10.959451	8.734402	7.004641
C	-12.768493	6.636474	6.585951
H	-12.560280	6.421595	5.531258
H	-13.474247	5.883004	6.953611
H	-13.273531	7.608702	6.626067
N	-4.795588	6.600686	11.342341
C	-3.626534	7.316630	10.834710
H	-3.179796	6.752871	10.010176
H	-3.935164	8.297823	10.461331
H	-2.868900	7.456260	11.628127
C	-4.398003	5.282489	11.836172
H	-5.274074	4.744319	12.209535
H	-3.965922	4.694952	11.020377
H	-3.654380	5.369049	12.650101
C	-5.413121	7.379199	12.416088
H	-5.740404	8.348717	12.025424
H	-6.287013	6.845446	12.801068
H	-4.701234	7.548639	13.244976

model-5-d

SCF (wB97x) = -5466.89896839
E(SCF)+ZPE(0 K)= -5466.883568
H(298 K)= -5466.873740
G(298 K)= -5466.921509
Lowest Frequency = 70.3692cm-1

Pd	0.000194	0.000032	-0.000008
Zn	-0.590503	-2.468680	0.000014
Zn	-1.842273	1.745472	-0.000045
Zn	2.433224	0.723794	-0.000008
H	-1.696327	0.001239	-0.000021
H	0.849258	1.468864	-0.000018
H	0.846768	-1.470957	0.000025

model-5

SCF (wB97x) = -5466.89884992
E(SCF)+ZPE(0 K)= -5466.877982
H(298 K)= -5466.868837
G(298 K)= -5466.915378
Lowest Frequency = 70.6465cm-1

Pd	0.000194	0.000032	-0.000008
Zn	-0.590503	-2.468680	0.000014
Zn	-1.842273	1.745472	-0.000045
Zn	2.433224	0.723794	-0.000008
H	-1.696327	0.001239	-0.000021
H	0.849258	1.468864	-0.000018
H	0.846768	-1.470957	0.000025

model-5-theta30

SCF (wB97x) = -5466.83114327

Pd	0.000064	0.000087	0.000005
Zn	2.322359	1.024989	0.000029
Zn	-0.273207	-2.523123	-0.000028
Zn	-2.049270	1.497993	-0.000010
H	0.685196	-1.551936	-0.000027
H	-1.686705	0.182255	-0.000000
H	1.002122	1.369896	0.000036

model-5-theta35

SCF (wB97x) = -5466.87891887

Pd	0.000064	0.000087	0.000005

Zn	2.322369	1.024964	0.000029
Zn	-0.273233	-2.523120	-0.000028
Zn	-2.049255	1.498014	-0.000010
H	0.817841	-1.486326	-0.000028
H	-1.696163	0.034567	0.000001
H	0.878937	1.452010	0.000038

model-5-theta40

SCF (wB97x) = -5466.89655789

Pd	0.000064	0.000086	0.000005
Zn	2.322069	1.025643	0.000029
Zn	-0.272495	-2.523200	-0.000028
Zn	-2.049693	1.497414	-0.000010
H	0.944691	-1.409120	-0.000029
H	-1.692680	-0.113895	0.000002
H	0.748604	1.523302	0.000039

model-5-theta45

SCF (wB97x) = -5466.89859537

Pd	0.000064	0.000086	0.000005
Zn	2.321464	1.027013	0.000029
Zn	-0.271007	-2.523361	-0.000028
Zn	-2.050576	1.496205	-0.000010
H	1.064696	-1.320800	-0.000029
H	-1.676150	-0.261983	0.000002
H	0.612065	1.583106	0.000041

model-5-theta50

SCF (wB97x) = -5466.89462593

Pd	0.000064	0.000086	0.000005
Zn	2.320568	1.029035	0.000029
Zn	-0.268808	-2.523596	-0.000028
Zn	-2.051879	1.494417	-0.000010
H	1.176833	-1.221960	-0.000029
H	-1.646576	-0.408513	0.000003
H	0.470346	1.630832	0.000042

model-5-theta55

SCF (wB97x) = -5466.89024448

Pd	0.000064	0.000086	0.000005
Zn	2.319405	1.031653	0.000029
Zn	-0.265960	-2.523898	-0.000028
Zn	-2.053564	1.492101	-0.000010
H	1.280120	-1.113304	-0.000029
H	-1.604076	-0.552283	0.000005
H	0.324548	1.665981	0.000042

model-5-theta60

SCF (wB97x) = -5466.88844657

Pd	0.000064	0.000086	0.000005
Zn	2.318009	1.034786	0.000029
Zn	-0.262549	-2.524255	-0.000028
Zn	-2.055578	1.489324	-0.000010
H	1.373635	-0.995648	-0.000029
H	-1.548895	-0.692085	0.000006
H	0.175839	1.688162	0.000043

model-6a

SCF (wB97x) = -728.895201768
E(SCF)+ZPE(0 K)= -728.875630
H(298 K)= -728.866385
G(298 K)= -728.909873
Lowest Frequency = 88.5782cm-1

Pd	-0.000147	-0.000030	0.000000
Mg	-2.496103	-0.320174	0.000008
Mg	0.970865	2.321537	0.000008
Mg	1.525483	-2.001318	-0.000016
H	-1.021536	1.351458	0.000011
H	1.680774	0.211465	-0.000006
H	-0.655416	-1.562105	-0.000005

model-6a-theta30

SCF (wB97x) = -728.748781194

Pd	0.000000	0.000000	0.000000
Mg	-1.258223	2.179307	0.000000
Mg	2.516447	0.000000	0.000000
Mg	-1.258223	-2.179307	0.000000
H	0.000000	1.694049	0.000000
H	1.467089	-0.847024	0.000000
H	-1.467089	-0.847024	0.000000

model-6a-theta35

SCF (wB97x) = -728.827691785

Pd	0.000000	0.000000	0.000000
Mg	-1.443375	2.061352	0.000000
Mg	2.506871	0.219323	0.000000
Mg	-1.063496	-2.280675	0.000000
H	0.000000	1.694049	0.000000
H	1.467089	-0.847024	0.000000
H	-1.467089	-0.847024	0.000000

model-6a-theta40

SCF (wB97x) = -728.868747019

Pd	0.000000	0.000000	0.000000
Mg	-1.617541	1.927710	0.000000
Mg	2.478216	0.436976	0.000000
Mg	-0.860675	-2.364686	0.000000
H	0.000000	1.694049	0.000000
H	1.467089	-0.847024	0.000000
H	-1.467089	-0.847024	0.000000

model-6a-theta45

SCF (wB97x) = -728.887131747

Pd	0.000000	0.000000	0.000000
Mg	-1.779397	1.779397	0.000000
Mg	2.430701	0.651304	0.000000
Mg	-0.651304	-2.430701	0.000000
H	0.000000	1.694049	0.000000
H	1.467089	-0.847024	0.000000
H	-1.467089	-0.847024	0.000000

model-6a-theta50

SCF (wB97x) = -728.893634630

Pd	0.000000	0.000000	0.000000
Mg	-1.927710	1.617541	0.000000
Mg	2.364686	0.860675	0.000000
Mg	-0.436976	-2.478216	0.000000
H	0.000000	1.694049	0.000000
H	1.467089	-0.847024	0.000000
H	-1.467089	-0.847024	0.000000

model-6a-theta55

SCF (wB97x) = -728.895087951

Pd	0.000000	0.000000	0.000000
Mg	-2.061352	1.443375	0.000000
Mg	2.280675	1.063496	0.000000
Mg	-0.219323	-2.506871	0.000000
H	0.000000	1.694049	0.000000
H	1.467089	-0.847024	0.000000
H	-1.467089	-0.847024	0.000000

model-6a-theta60

SCF (wB97x) = -728.895191816

Pd	0.000000	0.000000	0.000000
Mg	2.179307	1.258223	0.000000
Mg	-2.179307	1.258223	0.000000
Mg	0.000000	-2.516447	0.000000
H	0.000000	1.694049	0.000000
H	-1.467089	-0.847024	0.000000
H	1.467089	-0.847024	0.000000

model-6b-d

SCF (wB97x) = -720.395942339
E(SCF)+ZPE(0 K)= -720.380413
H(298 K)= -720.370500
G(298 K)= -720.415699
Lowest Frequency = 90.1087cm-1

Pt	0.000791	-0.002786	-0.000006
Mg	-0.811617	-2.430099	0.000492
Mg	-1.695316	1.914811	0.000540
Mg	2.508621	0.510758	-0.001061
H	-1.647366	-0.326913	0.000701
H	0.528968	1.592890	-0.000350
H	1.116262	-1.258898	-0.000375

model-6b

SCF (wB97x) = -720.395942338
E(SCF)+ZPE(0 K)= -720.375213
H(298 K)= -720.366073
G(298 K)= -720.409659
Lowest Frequency = 90.1439cm-1

Pt	0.000791	-0.002786	-0.000006
Mg	-0.811617	-2.430099	0.000492
Mg	-1.695316	1.914811	0.000540
Mg	2.508621	0.510758	-0.001061
H	-1.647366	-0.326913	0.000701
H	0.528968	1.592890	-0.000350
H	1.116262	-1.258898	-0.000375

10. References

[1] Feldman, J.; McLain, S. J.; Parthasarathy, A.; Marshall, W. J.; Calabrese, J. C.; Arthur, S. D. Electrophilic Metal Precursors and a β-Diimine Ligand for Nickel(II)- and Palladium(II)-Catalyzed Ethylene Polymerization. *Organometallics* **1997**, *16*, 1514–1516.

[2] Garçon, M.; Mun, N. W.; White, A. J. P.; Crimmin, M. R. Palladium-Catalysed C–H Bond Zincation of Arenes: Scope, Mechanism, and the Role of Heterometallic Intermediates. *Angew. Chem. Int. Ed.* **2021**, *60*, 2–11.

[3] Schulz, S.; Eisenmann, T.; Schuchmann, D.; Bolte, M.; Kirchner, M.; Boese, R.; Spielmann, J.; Harder, S. Solid-state and Solution Studies on a β-Diketiminate Zinc Hydride Complex. *Z. Naturforsch.* **2009**, *64b*, 1397–1400.

[4] Garçon, M.; Bakewell, C.; Sackman, G. A.; White, A. J. P.; Cooper, R. I.; Edwards, A. J.; Crimmin, M. R. A hexagonal planar transition-metal complex. *Nature* **2019**, *574*, 390–393.

[5] Coumbarides, G. S.; Eames, J.; Weerasooriya, N. A Practical Laboratory Route to the Synthesis of Trideuteriomethyl-[^{13}C] Iodide. *J. Label Compd. Radiopharm.* **2003**, *46*, 291–296.

[6] Göttker-Schmetmann, I.; Mecking, S. A Practical Synthesis of [(tmeda)Ni(CH_3)$_2$], Isotopically Labeled [(tmeda)Ni($^{13}CH_3$)$_2$], and Neutral Chelated-Nickel Methyl Complexes. *Organometallics* **2020**, *39*, 3433–3440.

[7] Proutiere, F.; Lyngvi, E.; Aufiero, M.; Sanhueza, I. A.; Schoenebeck, F. Combining the Reactivity Properties of PCy_3 and P*t*Bu_3 into a Single Ligand, P(*i*Pr)(*t*Bu)$_2$. Reaction via Mono- or Bisphosphine Palladium(0) Centers and Palladium(I) Dimer Formation. *Organometallics* **2014**, *33*, 6879–6884.

[8] a) SHELXTL v5.1, Bruker AXS, Madison, WI, 1998. b) SHELX-2013, G.M. Sheldrick, *Acta Cryst.*, **2015**, *C71*, 3–8.

[9] Green, S. P.; Jones, C.; Stasch, A. Stable Adducts of a Dimeric Magnesium(I) Compound. *Angew. Chem. Int. Ed.* **2008**, *47*, 9079–9083.

[10] Prust, J.; Stasch, A.; Zheng, W.; Roesky, H. W.; Alexopoulos, E.; Usón, I.; Böhler, D.; Schuchardt, T. Synthesis and Structural Characterization of Monomeric Three-Coordinated β-Diketoiminate Organozinc Derivatives. *Organometallics* **2001**, *20*, 3825–3828.

[11] Schulz, S.; Eisenmann, Westphal, U.; Schmidt, S.; Flörke, U. Synthesis and Characterization of β-Diketiminate Zinc Complexes. *Z. Anorg. Allg. Chem.* **2009**, *635*, 216–220.

[12] Scheiper, C.; Schulz, S.; Wölper, C.; Bläser, D.; Roll, J. Synthesis and Single Crystal X-ray Structures of Cationic Zinc β-Diketiminate Complexes. *Z. Anorg. Allg. Chem.* **2013**, *639*, 1153–1159.

[13] A.L. Spek (2003, 2009) PLATON, A Multipurpose Crystallographic Tool, Utrecht University, Utrecht, The Netherlands. See also A.L. Spek, *Acta. Cryst.*, 2015, **C71**, 9-18

[14] Piltz, R., *Acta Cryst. J. Appl. Cryst.* **2018**, *51*, 963–965.

[15] Wilkinson, C.; Khamis, H. W.; Stansfield, R. F. D.; McIntyre, G. J. *J. Appl. Cryst.* **1988**, *21*, 471.

[16] Betteridge, P. W.; Carruthers, J. R.; Cooper, R. I.; Prout, K.; Watkin, D. J. *J. Appl. Cryst.* **2003**, *36*, 1487.

[17] Bau, R.; Drabnis, M. H. Structures of Transition Metal Hydrides Determined by Neutron Diffraction. *Inorg. Chim. Acta* **1997**, *259*, 27–50.

[18] King, R. B. Structure and Bonding in Homoleptic Transition Metal Hydride Anions. *Coord. Chem. Rev.* **2000**, *200-202*, 813–829.

[19] Olofsson, M.; Kritikos, M.; Noréus, D. The First Trigonal Planar Transition Metal–Hydrogen Complex in $NaBaPdH_3$. *Inorg. Chem.* **1998**, *37*, 2900–2902.

[20] Olofsson-Mårtensson, M.; Kritikos, M.; Noréus, D. A Novel Tetrahedral Formally Zerovalent-Palladium Hydrido Complex Stabilized by Divalent Alkaline Earth Counterions. *J. Am. Chem. Soc.* **1999**, *121*, 10908–10912.

[21] Olofsson-Mårtensson, M.; Häussermann, U.; Tomkinson, J.; Noréus, D. Stabilization of Electron-Dense Palladium–Hydrido Complexes in Solid-State Hydrides. *J. Am. Chem. Soc.* **2000**, *122*, 6960–6970.

[22] Frisch, M. J.; Trucks, G. W.; Schlegel, H. B.; Scuseria, G. E.; Robb, M. A.; Cheeseman, J. R.; Scalmani, G.; Barone, V.; Mennucci, G.; Petersson, G. A.; Nakatsuji, H.; Caricato, M.; Li, X.; Hratchian, H. P.; Izmaylov, A. F.; Bloino, J.; Zheng, G.; Sonnenberg, J. L.; Hada, M.; Ehara, M.; Toyota, K.; Fukuda, R.; Hasegawa, J.; Ishida, M.; Nakajima, T.; Honda, Y.; Kitao, O.; Nakai, H.; Vreven, T.; Montgomery, J. A., Jr.; Peralta, J. E.; Ogliaro, F.; Bearpark, M.; Heyd, J. J.; Brothers, E.; Kudin, K. N.; Staroverov, V. N.; Kobayashi, R.; Normand, J.; Raghavachari, K.; Rendell, A.; Burant, J. C.; Iyengar, S. S.; Romasi, J.; Cossi, M.; Rega, N.; Millam, J. M.; Klene, M.; Knox, J. E.; Cross, J. B.; Bakken, V.; Adamo, C.; Jaramillo, J.; Gomperts, R.; Stratmann, R. E.; Yazyev, O.; Austin, A. J.; Cammi, R.; Pomelli, C.; Ochterski, J. W.; Martin, R. L.; Morokuma, K.; Zakrzewski, V. G.; Voth, G. A.; Salvador, P.; Dannenberg, J. J.; Dapprich, S.; Daniels, A. D.; Farkas, Ö.; Foresman, J. B.; Ortiz, J. V.; Cioslowski, J.; Fox, D. J. *Gaussian 09*, *Revision D.01*; Gaussian, Inc., Wallingford, CT, USA (2009).

[23] Gaussian 16, Revision C.01, Frisch, M. J.; Trucks, G. W.; Schlegel, H. B.; Scuseria, G. E.; Robb, M. A.; Cheeseman, J. R.; Scalmani, G.; Barone, V.; Petersson, G. A.; Nakatsuji, H.; Li, X.; Caricato, M.; Marenich, A. V.;

Bloino, J.; Janesko, B. G.; Gomperts, R.; Mennucci, B.; Hratchian, H. P.; Ortiz, J. V.; Izmaylov, A. F.; Sonnenberg, J. L.; Williams-Young, D.; Ding, F.; Lipparini, F.; Egidi, F.; Goings, J.; Peng, B.; Petrone, A.; Henderson, T.; Ranasinghe, D.; Zakrzewski, V. G.; Gao, J.; Rega, N.; Zheng, G.; Liang, W.; Hada, M.; Ehara, M.; Toyota, K.; Fukuda, R.; Hasegawa, J.; Ishida, M.; Nakajima, T.; Honda, Y.; Kitao, O.; Nakai, H.; Vreven, T.; Throssell, K.; Montgomery, J. A., Jr.; Peralta, J. E.; Ogliaro, F.; Bearpark, M. J.; Heyd, J. J.; Brothers, E. N.; Kudin, K. N.; Staroverov, V. N.; Keith, T. A.; Kobayashi, R.; Normand, J.; Raghavachari, K.; Rendell, A. P.; Burant, J. C.; Iyengar, S. S.; Tomasi, J.; Cossi, M.; Millam, J. M.; Klene, M.; Adamo, C.; Cammi, R.; Ochterski, J. W.; Martin, R. L.; Morokuma, K.; Farkas, O.; Foresman, J. B.; Fox, D. J. Gaussian, Inc., Wallingford CT, 2016.

[24] Chai, J.-D.; Head-Gordon, M. Systematic Optimization of Long-Range Corrected Hybrid Density Functionals. *J. Chem. Phys.* **2008**, *128*, 084106.

[25] *NBO 6.0.* Glendening, E. D.; Badenhoop, J. K.; Reed, A. E.; Carpenter, J. E.; Bohmann, J. A.; Morales, C. M.; Landis, C. R.; Weinhold, F. Theoretical Chemistry Institute, University of Misconsin, Madison (2013).

[26] AIMAll (Version 13.10.19), Keith, T. A. TK Gristmill Software, Overland Park, KS, USA (2013) (aim.tkgristmill.com)

[27] Johnson, E. R.; Keinan, S.; Mori-Sánchez, P.; Contreras-García, J.; Cohen, A. J.; Yang, W. Revealing Noncovalent Interactions. *J. Am. Chem. Soc.* **2010**, *132*, 6498–6506.

[28] Garçon, M.; White, A. J. P.; Crimmin, M. R. Palladium-Catalysed Magnesiation of Benzene. *Chem. Commun.* **2018**, *54*, 12326–12328.

[29] Weigend, F.; Ahlrichs, R. Balanced basis sets of split valence, triple zeta valence and quadruple zeta valence quality for H to Rn: Design and assessment of accuracy. *Phys. Chem. Chem. Phys.* **2005**, *7*, 3297-3305.
