# Supplementary material for: The Continuum Between Hexagonal Planar and Trigonal Planar Geometries
Source: Angew Chem Int Ed Engl. 2022 Oct 5;61(44):e202211948. doi: 10.1002/anie.202211948 (PMC9828084; doi:10.1002/anie.202211948)

checkCIF/PLATON report

Structure factors have been supplied for datablock(s) I

THIS REPORT IS FOR GUIDANCE ONLY. IF USED AS PART OF A REVIEW PROCEDURE FOR PUBLICATION, IT SHOULD NOT REPLACE THE EXPERTISE OF AN EXPERIENCED CRYSTALLOGRAPHIC REFEREE.

No syntax errors found.   CIF dictionary   Interpreting this report

Datablock: I

Bond precision: C-C = 0.0318 A   Wavelength=0

Cell:	a=11.9440(4)	b=12.0368(6)	c=22.8595(9)
	alpha=91.871(4)	beta=104.635(3)	gamma=112.172(4)
Temperature:	173 K		

	Calculated	Reported
Volume	2914.5(2)	2914.5(2)
Space group	P -1	P -1
Hall group	-P 1	-P 1
Moiety formula	C55 H90 Mg N2 P2 Pd, 0.5(C6 H14)	C55 H90 Mg1 N2 P2 Pd1, 0.5(C6 H14)
Sum formula	C58 H97 Mg N2 P2 Pd	C58 H97 Mg1 N2 P2 Pd1
Mr	1015.03	1015.07
Dx,g cm-3	1.157	1.157
Z	2	2
Mu (mm-1)	0.000	0.000
F000	125.7	126.0
F000'	1093.85	
h,k,lmax		10,10,20
Nref		3625
Tmin,Tmax		
Tmin'		

Correction method= Not given

Data completeness=   Theta(max)= 50.370

R(reflections)= 0.1411(1899)   wR2(reflections)= wR= 0.4397(1534)

S = 0.970   Npar= 1400

The following ALERTS were generated. Each ALERT has the format
test-name_ALERT_alert-type_alert-level.
Click on the hyperlinks for more details of the test.

Alert level A

```
DIFF002_ALERT_1_A  _diffrn_radiation_wavelength is missing
            Radiation wavelength (A).
            The following tests will not be performed.
            RADNW_01,REFLT_03,REFNR_01,THETM_01
```

Author Response: This is a Laue neutron diffraction experiment, so uses a range of wavelengths. This wavelength range can be seen in the _reflns_special_details section of this CIF, under LAUEG SPECIFIC INFORMATION. If you wish to read this CIF into your refinement program of choice and refine the model yourself, uncomment the line in the CIF starting '_diffrn_radiation_wavelength' to use a wavelength of 0.85 Angstroms.

```
RINTA01_ALERT_3_A  The value of Rint is greater than 0.25
            Rint given   0.281
```

Author Response: This Rint value is calculated according to monochromatic X-ray conventions which are not applicable here. Laue neutron data are inherently noisy if you simply merge all the "measured" reflections. The 4sigma Rint value reported from the data reduction within LaueG is a meaningful discriminant of the data merging validity, and is 7.7 percent, with an esd of 6.6.

```
PLAT020_ALERT_3_A The Value of Rint is Greater Than 0.12 .........      0.281 Report
```

Author Response: This Rint value is calculated according to monochromatic X-ray conventions which are not applicable here. Laue neutron data are inherently noisy if you simply merge all the "measured" reflections. The 4sigma Rint value reported from the data reduction within LaueG is a meaningful discriminant of the data merging validity, and is 7.7 percent, with an esd of 6.6.

```
PLAT088_ALERT_3_A Poor Data / Parameter Ratio ...................       1.10 Note
```

Author Response: Laue neutron data from a reactor source render the experimental measurement feasible. It is inherent in the method that some data are affected by overlap (non unique indexing due to wavelength spectrum). The data collected and parameterization chosen give rise to a stable, convergent refinement, with the application of minimal restraints, in which anisotropic displacement parameters are included for the non-hydrogen and hydrogen atoms in the refinement. The result is a chemically and physically valid model with experimentally derived uncertainties which reflect the limitations of the data.

PLAT902_ALERT_1_A No (Interpretable) Reflections Found in FCF .... Please Check

Author Response: The reflections are interpretable, they are reported as F values as we are refining on F. The issue is with checkCIF, as it expects F squared values.

PLAT992_ALERT_5_A Repd & Actual _reflns_number_gt Values Differ by 365 Check

Author Response: 365 is the difference between _reflns_number_gt (1899) at 2sigma and _refine_ls_number_reflns (1534) at 3sigma. There is a 3sigma threshold used for the reflections included in this refinement, to produce a more chemically and physically valid model. The additional 365 reflections are included in the refinement if this threshold is reduced to 2sigma. Refining at 2sigma means _reflns_number_gt and _refine_ls_number_reflns match, and this alert is no longer present. Therefore, this is not an issue with the model, but checkCIF's expectation that the threshold used in the refinement matches the 2sigma SHELX standard.

Alert level B

PLAT084_ALERT_3_B High wR2 Value (i.e. > 0.25) .... 0.44 Report

Author Response: This wR2 value is calculated using a -10sigma cutoff, which is not meaningful in the context of this experiment. This is because Laue neutron data are inherently noisy if you simply merge all the "measured" reflections, hence using a -10sigma cutoff results in a large amount of noisy data being included in the wR2 calculation. A 3sigma cutoff was used in the refinement here, which produces a meaningful wR2 value (0.1370 in this case).

PLAT342_ALERT_3_B Low Bond Precision on C-C Bonds .... 0.03179 Ang.

Author Response: The bond precision values encoded in checkCIF are based on monochromatic X-ray assumptions. The e.s.d. derives from the data, which derives from the crystal and the experiment performed. The standard uncertainty provides a guide to how values in the structure can be compared within this structure and to others. The refinement has been optimised to yield the most informative model.

PLAT410_ALERT_2_B Short Intra H...H Contact H321 ..H462 . 1.89 Ang.
x,y,z = 1_555 Check

Author Response: This VRF alert was triggered because the expected H...H contact values are biased towards reporting X-ray derived models. When using X-ray diffraction data to determine distances to hydrogen, there is an assumption that the atoms in a model lie at the centre of the observed electron scattering density. But this is not entirely true, particularly for light atoms such as hydrogen, and results in a shortening of the distances to hydrogen in a model derived from X-ray data. These data were measured using Laue neutron diffraction, which measures nuclear distances, so the same shortening effect is not observed. Therefore shorter H...H contact values are entirely expected for a model derived from neutron diffraction data, rather than X-rays.

Alert level C

```
PLAT082_ALERT_2_C High R1 Value ..................................       0.14 Report
```

Author Response: The R1 value threshold used to trigger this VRF alert is biased towards reporting on monochromatic X-ray experiments, rather than Laue neutron experiments. The R1 value reported here, relative to the experiment, and the data collected, approaches an optimal value.

```
PLAT202_ALERT_3_C Isotropic non-H Atoms in Anion/Solvent ........          3 Check
              C101    C102    C103
```

Author Response: The solvating molecule sites have been refined isotropically, with distance and angular restraints used to maintain a sensible geometry. Anisotropic refinement of these sites resulted in physically unreasonable displacement ellipsoids, while an isotropic model is adequate.

Alert level G

```
ABSMU01_ALERT_1_G  Calculation of _exptl_absorpt_correction_mu
                not performed for this radiation type.
PLAT002_ALERT_2_G Number of Distance or Angle Restraints on AtSite        152 Note
PLAT003_ALERT_2_G Number of Uiso or Uij Restrained non-H Atoms ...         64 Report
PLAT042_ALERT_1_G Calc. and Reported Moiety Formula Strings Differ     Please Check
PLAT164_ALERT_4_G Nr. of Refined C-H H-Atoms in Heavy-Atom Struct.         96 Note
PLAT303_ALERT_2_G Full Occupancy Atom H1        with # Connections      2.00 Check
PLAT333_ALERT_2_G Large Aver C6-Ring C-C Dist C6       -C11      .      1.43 Ang.
PLAT720_ALERT_4_G Number of Unusual/Non-Standard Labels ..........          7 Note
PLAT769_ALERT_4_G CIF Embedded explicitly supplied scattering data     Please Note
PLAT860_ALERT_3_G Number of Least-Squares Restraints .............       1364 Note
PLAT871_ALERT_4_G Laue technique Related ALERTS are Suppressed ...          ! Info
PLAT909_ALERT_3_G Percentage of I>2sig(I) Data at Theta(Max) Still       100% Note
PLAT929_ALERT_5_G No Weight Pars,Obs and Calc R1,wR2,S not Checked          ! Info
PLAT956_ALERT_1_G Calculated (ThMax) and Actual (FCF) Hmax Differ         10 Units
PLAT957_ALERT_1_G Calculated (ThMax) and Actual (FCF) Kmax Differ         10 Units
```

```
PLAT958_ALERT_1_G Calculated (ThMax) and Actual (FCF) Lmax Differ          20 Units
```

```
   6 ALERT level A = Most likely a serious problem - resolve or explain
   3 ALERT level B = A potentially serious problem, consider carefully
   2 ALERT level C = Check. Ensure it is not caused by an omission or oversight
  16 ALERT level G = General information/check it is not something unexpected

   7 ALERT type 1 CIF construction/syntax error, inconsistent or missing data
   6 ALERT type 2 Indicator that the structure model may be wrong or deficient
   8 ALERT type 3 Indicator that the structure quality may be low
   4 ALERT type 4 Improvement, methodology, query or suggestion
   2 ALERT type 5 Informative message, check
```

---

checkCIF publication errors

Alert level A

```
PUBL004_ALERT_1_A  The contact author's name and address are missing,
            _publ_contact_author_name and _publ_contact_author_address.
PUBL005_ALERT_1_A  _publ_contact_author_email, _publ_contact_author_fax and
            _publ_contact_author_phone are all missing.
            At least one of these should be present.
PUBL006_ALERT_1_A  _publ_requested_journal is missing
            e.g. 'Acta Crystallographica Section C'
PUBL008_ALERT_1_A  _publ_section_title is missing. Title of paper.
PUBL009_ALERT_1_A  _publ_author_name is missing. List of author(s) name(s).
PUBL010_ALERT_1_A  _publ_author_address is missing. Author(s) address(es).
PUBL012_ALERT_1_A  _publ_section_abstract is missing.
            Abstract of paper in English.
```

```
   7 ALERT level A = Data missing that is essential or data in wrong format
   0 ALERT level G = General alerts. Data that may be required is missing
```

Publication of your CIF

You should attempt to resolve as many as possible of the alerts in all categories. Often the minor alerts point to easily fixed oversights, errors and omissions in your CIF or refinement strategy, so attention to these fine details can be worthwhile. In order to resolve some of the more serious problems it may be necessary to carry out additional measurements or structure refinements. However, the nature of your study may justify the reported deviations from journal submission requirements and the more serious of these should be commented upon in the discussion or experimental section of a paper or in the "special_details" fields of the CIF. *checkCIF* was carefully designed to identify outliers and unusual parameters, but every test has its limitations and alerts that are not important in a particular case may appear. Conversely, the absence of alerts does not guarantee there are no aspects of the results needing attention. It is up to the individual to critically assess their own results and, if necessary, seek expert advice.

If level A alerts remain, which you believe to be justified deviations, and you intend to submit this CIF for publication in a journal, you should additionally insert an explanation in your CIF using the Validation Reply Form (VRF) below. This will allow your explanation to be considered as part of the review process.

Validation response form

Please find below a validation response form (VRF) that can be filled in and pasted into your CIF.

```
# start Validation Reply Form
_vrf_PUBL004_GLOBAL
;
PROBLEM: The contact author's name and address are missing,
RESPONSE: ...
;
_vrf_PUBL005_GLOBAL
;
PROBLEM: _publ_contact_author_email, _publ_contact_author_fax and
RESPONSE: ...
;
_vrf_PUBL006_GLOBAL
;
PROBLEM: _publ_requested_journal is missing
RESPONSE: ...
;
_vrf_PUBL008_GLOBAL
;
PROBLEM: _publ_section_title is missing. Title of paper.
RESPONSE: ...
;
_vrf_PUBL009_GLOBAL
;
PROBLEM: _publ_author_name is missing. List of author(s) name(s).
RESPONSE: ...
;
_vrf_PUBL010_GLOBAL
;
PROBLEM: _publ_author_address is missing. Author(s) address(es).
```

```
RESPONSE: ...
;
_vrf_PUBL012_GLOBAL
;
PROBLEM: _publ_section_abstract is missing.
RESPONSE: ...
;
# end Validation Reply Form
```

If you wish to submit your CIF for publication in Acta Crystallographica Section C or E, you should upload your CIF via the web. If you wish to submit your CIF for publication in IUCrData you should upload your CIF via the web. If your CIF is to form part of a submission to another IUCr journal, you will be asked, either during electronic submission or by the Co-editor handling your paper, to upload your CIF via our web site.

PLATON version of 13/07/2021; check.def file version of 13/07/2021

Datablock I - ellipsoid plot

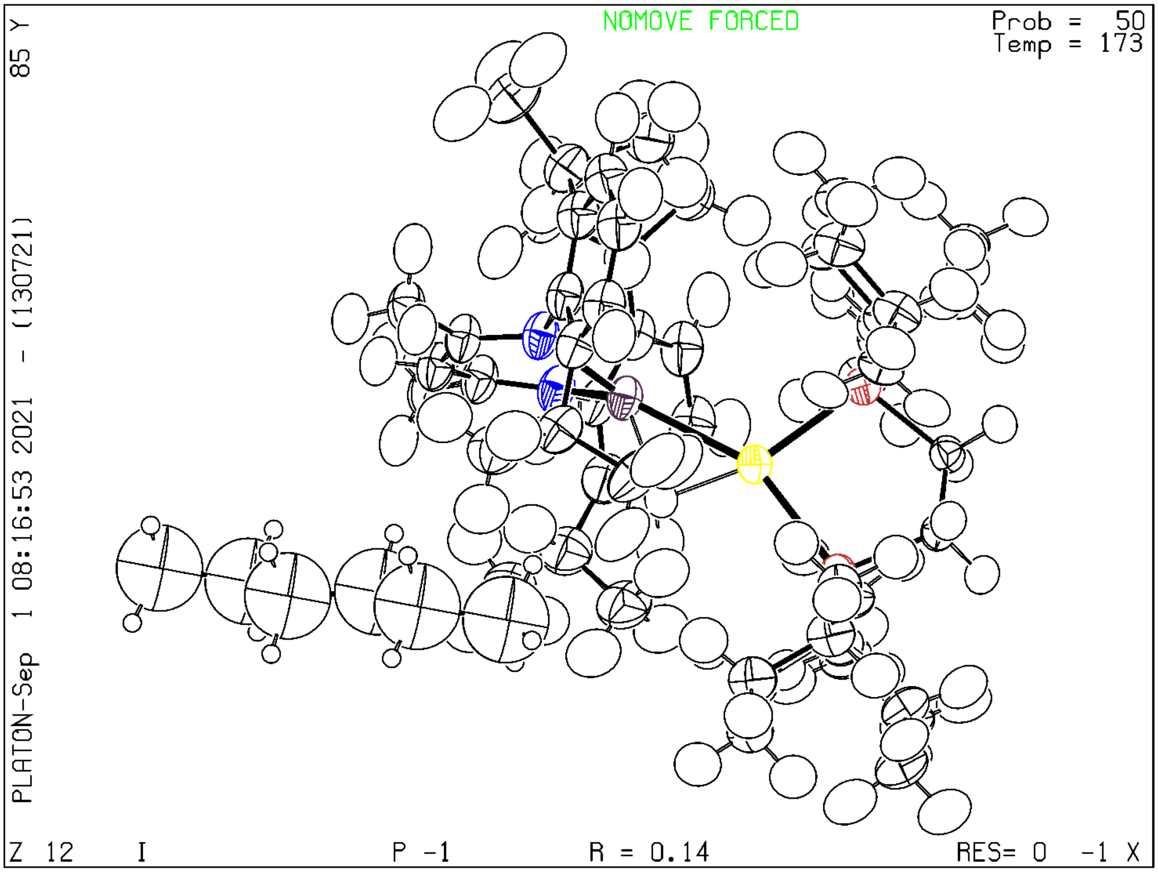

checkCIF/PLATON report

Structure factors have been supplied for datablock(s) I

THIS REPORT IS FOR GUIDANCE ONLY. IF USED AS PART OF A REVIEW PROCEDURE FOR PUBLICATION, IT SHOULD NOT REPLACE THE EXPERTISE OF AN EXPERIENCED CRYSTALLOGRAPHIC REFEREE.

No syntax errors found. CIF dictionary Interpreting this report

Datablock: I

Bond precision:	C-C = 0.0276 A		Wavelength=0
Cell:	a=14.8444(3)	b=16.0913(2)	c=16.9949(3)
	alpha=96.1668(14)	beta=97.1851(15)	gamma=97.9294(14)
Temperature:	173 K		

	Calculated	Reported
Volume	3956.87(12)	3956.87(12)
Space group	P -1	P -1
Hall group	-P 1	-P 1
Moiety formula	C76 H117 Mg2 N4 P Pt, C6 H14	C76 H117 Mg2 N4 P1 Pt1, C6 H14
Sum formula	C82 H131 Mg2 N4 P Pt	C82 H131 Mg2 N4 P1 Pt1
Mr	1447.59	1447.64
Dx,g cm-3	1.215	1.215
Z	2	2
Mu (mm-1)	0.000	0.000
F000	235.7	236.1
F000'	1535.67	
h,k,lmax		13,14,15
Nref		5161
Tmin,Tmax		
Tmin'		

Correction method= Not given

Data completeness= Theta(max)= 51.090

R(reflections)= 0.1794(2263) wR2(reflections)= wR= 0.6651(1806)

S = 0.860 Npar= 1315

The following ALERTS were generated. Each ALERT has the format
test-name_ALERT_alert-type_alert-level.
Click on the hyperlinks for more details of the test.

Alert level A

```
DIFF002_ALERT_1_A  _diffrn_radiation_wavelength is missing
            Radiation wavelength (A).
            The following tests will not be performed.
            RADNW_01,REFLT_03,REFNR_01,THETM_01
RINTA01_ALERT_3_A  The value of Rint is greater than 0.25
            Rint given   0.328
```

Author Response: This Rint value is the result of using the Laue neutron method to collect the data, which are inherently noisy when you consider all the reflections. However, the 4sigma Rint value obtained from the data reduction within LaueG is a more meaningful measure of the data quality, and in this case is 12.3 percent, with an esd of 7.8.

```
PLAT020_ALERT_3_A The Value of Rint is Greater Than 0.12 .........      0.328 Report
```

Author Response: This Rint value is the result of using the Laue neutron method to collect the data, which are inherently noisy when you consider all the reflections. However, the 4sigma Rint value obtained from the data reduction within LaueG is a more meaningful measure of the data quality, and in this case is 12.3 percent, with an esd of 7.8.

```
PLAT084_ALERT_3_A High wR2 Value (i.e. > 0.25) ..................       0.67 Report
```

Author Response: Since the Laue neutron method was used, the data collected are inherently noisy. So this wR2 value, calculated using a -10sigma cutoff in this case, will be high. The wR2 value calculated using the 3sigma cutoff actually used in the refinement is 0.1942.

```
PLAT088_ALERT_3_A Poor Data / Parameter Ratio ...................       1.37 Note
```

Author Response: The data were collected using the Laue neutron method, which is inherently noisy, and anisotropic displacement parameters have been included for the non-hydrogen atoms in the refinement. Both of these aspects contribute to the poor data / parameter ratio. However, the refinement of the parameters chosen is stable, and converges well in the context of the reflections and additional restraint observations. The result is a chemically and physically valid model.

```
PLAT902_ALERT_1_A No (Interpretable) Reflections Found in FCF ....     Please Check
```

Author Response: The reflections are interpretable, as far as we can see this is a checkCIF issue.

```
PLAT992_ALERT_5_A Repd & Actual _reflns_number_gt Values Differ by        457 Check
```

Author Response: 457 is the difference between _reflns_number_gt (2263) at 2sigma and _refine_ls_number_reflns (1806) at 3sigma. There is a 3sigma threshold used for the reflections included in this refinement, to produce a more chemically and physically valid model. The additional 457 reflections are included in the refinement if this threshold is reduced to 2sigma. Refining at 2sigma means _reflns_number_gt and _refine_ls_number_reflns match, and this alert is no longer present. Therefore, this is not an issue with the model, but checkCIF's expectation that the threshold used in the refinement matches the 2sigma SHELX standard.

Alert level B

```
PLAT082_ALERT_2_B High R1 Value ................................       0.18 Report
```

Author Response: The R1 value threshold used to trigger this VRF alert is biased towards reporting on monochromatic X-ray experiments, rather than Laue neutron experiments. The R1 value reported here, relative to the experiment, and the data collected, approaches an optimal value.

```
PLAT342_ALERT_3_B Low Bond Precision on  C-C Bonds ..............    0.02758 Ang.
```

Author Response: These high e.s.d's can be attributed to the low data / parameter ratio. The refinement of the parameters chosen is stable, and converges well in the context of the reflections and additional restraint observations. The result is a chemically and physically valid model.

Alert level C

```
PLAT026_ALERT_3_C Ratio Observed / Unique Reflections (too) Low ..        44% Check
```

Author Response: By using the Laue neutron method much of the data will be of low intensity, due to the multiple wavelength nature of the data. Whilst longer data collections may allow for the observation of additional reflections, it was not possible to acquire that amount of beam-time on KOALA. The data collected are adequate, and the refinement using these data results in a chemically and physically valid model.

```
PLAT202_ALERT_3_C Isotropic non-H Atoms in Anion/Solvent .........          6 Check
              C91     C92     C93     C94     C95     C96
```

Author Response: The carbon sites of the hexane solvate are modelled in a more physically reasonable manner when refined isotropically rather than anisotropically.

```
PLAT222_ALERT_3_C NonSolvent Resd 1  H   Uiso(max)/Uiso(min) Range        6.7 Ratio
```

Author Response: Uiso(max) is associated with one of the methyl group hydrogen atoms which appears disordered if anisotropic refinement is attempted. This large value reflects the fact that the scatterers are possibly distributed over more than one site throughout the crystal.

```
PLAT410_ALERT_2_C Short Intra H...H Contact  H662     ..H721     .       1.96 Ang.
                                                   x,y,z  =      1_555 Check
```

Author Response: There is an assumption that the atoms in a model derived from X-ray data lie at the centre of the observed electron scattering density. But this is not entirely true, and results in a shortening of the distances to hydrogen in such models. These data were measured using Laue neutron diffraction, which measures nuclear distances, so the same shortening effect is not observed. Therefore shorter H...H contact values are entirely expected for a model derived from neutron diffraction data, rather than X-rays. This VRF alert was triggered because the expected H...H contact values are biased towards reporting X-ray derived models.

```
PLAT411_ALERT_2_C Short Inter H...H Contact  H241     ..H942     .       2.08 Ang.
                                                x,-1+y,z  =      1_545 Check
```

Author Response: This VRF alert was triggered because the expected H...H contact values are biased towards reporting X-ray derived models.

```
PLAT413_ALERT_2_C Short Inter XH3 .. XHn     H4233    ..H51      .       2.13 Ang.
                                               2-x,-y,-z  =      2_755 Check
```

Author Response: This VRF alert was triggered because the expected H...H contact values are biased towards reporting X-ray derived models.

Alert level G

```
ABSMU01_ALERT_1_G  Calculation of _exptl_absorpt_correction_mu
                not performed for this radiation type.
PLAT002_ALERT_2_G Number of Distance or Angle Restraints on AtSite        212 Note
PLAT003_ALERT_2_G Number of Uiso or Uij Restrained non-H Atoms ...         90 Report
PLAT042_ALERT_1_G Calc. and Reported Moiety Formula Strings Differ     Please Check
```

```
PLAT164_ALERT_4_G Nr. of Refined C-H H-Atoms in Heavy-Atom Struct.        129 Note
PLAT303_ALERT_2_G Full Occupancy Atom H1        with # Connections       2.00 Check
PLAT303_ALERT_2_G Full Occupancy Atom H2        with # Connections       2.00 Check
PLAT720_ALERT_4_G Number of Unusual/Non-Standard Labels ..........         56 Note
PLAT769_ALERT_4_G CIF Embedded explicitly supplied scattering data     Please Note
PLAT860_ALERT_3_G Number of Least-Squares Restraints ............        1362 Note
PLAT871_ALERT_4_G Laue technique Related ALERTS are Suppressed ...          ! Info
PLAT909_ALERT_3_G Percentage of I>2sig(I) Data at Theta(Max) Still       100% Note
PLAT929_ALERT_5_G No Weight Pars,Obs and Calc R1,wR2,S not Checked          ! Info
PLAT956_ALERT_1_G Calculated (ThMax) and Actual (FCF) Hmax Differ          12 Units
PLAT957_ALERT_1_G Calculated (ThMax) and Actual (FCF) Kmax Differ          14 Units
PLAT958_ALERT_1_G Calculated (ThMax) and Actual (FCF) Lmax Differ          15 Units
```

---

   7 **ALERT level A** = Most likely a serious problem - resolve or explain
   2 **ALERT level B** = A potentially serious problem, consider carefully
   6 **ALERT level C** = Check. Ensure it is not caused by an omission or oversight
  16 **ALERT level G** = General information/check it is not something unexpected

   7 ALERT type 1 CIF construction/syntax error, inconsistent or missing data
   8 ALERT type 2 Indicator that the structure model may be wrong or deficient
  10 ALERT type 3 Indicator that the structure quality may be low
   4 ALERT type 4 Improvement, methodology, query or suggestion
   2 ALERT type 5 Informative message, check

---

checkCIF publication errors

Alert level A

```
PUBL004_ALERT_1_A  The contact author's name and address are missing,
           _publ_contact_author_name and _publ_contact_author_address.
PUBL005_ALERT_1_A  _publ_contact_author_email, _publ_contact_author_fax and
           _publ_contact_author_phone are all missing.
           At least one of these should be present.
PUBL006_ALERT_1_A  _publ_requested_journal is missing
           e.g. 'Acta Crystallographica Section C'
PUBL008_ALERT_1_A  _publ_section_title is missing. Title of paper.
PUBL009_ALERT_1_A  _publ_author_name is missing. List of author(s) name(s).
PUBL010_ALERT_1_A  _publ_author_address is missing. Author(s) address(es).
PUBL012_ALERT_1_A  _publ_section_abstract is missing.
           Abstract of paper in English.
```

---

   7 **ALERT level A** = Data missing that is essential or data in wrong format
   0 **ALERT level G** = General alerts. Data that may be required is missing

---

Publication of your CIF

You should attempt to resolve as many as possible of the alerts in all categories. Often the minor alerts point to easily fixed oversights, errors and omissions in your CIF or refinement strategy, so attention to these fine details can be worthwhile. In order to resolve some of the more serious problems it may be necessary to carry out additional measurements or structure refinements. However, the nature of your study may justify the reported deviations from journal submission requirements and the more serious of these should be commented upon in the discussion or experimental section of a paper or in the "special_details" fields of the CIF. *checkCIF* was carefully designed to identify outliers and unusual parameters, but every test has its limitations and alerts that are not important in a particular case may appear. Conversely, the absence of alerts does not guarantee there are no aspects of the results needing attention. It is up to the individual to critically assess their own results and, if necessary, seek expert advice.

If level A alerts remain, which you believe to be justified deviations, and you intend to submit this CIF for publication in a journal, you should additionally insert an explanation in your CIF using the Validation Reply Form (VRF) below. This will allow your explanation to be considered as part of the review process.

Validation response form

Please find below a validation response form (VRF) that can be filled in and pasted into your CIF.

```
# start Validation Reply Form
_vrf_PUBL004_GLOBAL
;
PROBLEM: The contact author's name and address are missing,
RESPONSE: ...
;
_vrf_PUBL005_GLOBAL
;
PROBLEM: _publ_contact_author_email, _publ_contact_author_fax and
RESPONSE: ...
;
_vrf_PUBL006_GLOBAL
;
PROBLEM: _publ_requested_journal is missing
RESPONSE: ...
;
_vrf_PUBL008_GLOBAL
;
PROBLEM: _publ_section_title is missing. Title of paper.
RESPONSE: ...
;
_vrf_PUBL009_GLOBAL
;
PROBLEM: _publ_author_name is missing. List of author(s) name(s).
RESPONSE: ...
;
_vrf_PUBL010_GLOBAL
;
PROBLEM: _publ_author_address is missing. Author(s) address(es).
```

```
RESPONSE: ...
;
_vrf_PUBL012_GLOBAL
;
PROBLEM: _publ_section_abstract is missing.
RESPONSE: ...
;
_vrf_DIFF002_I
;
PROBLEM: _diffrn_radiation_wavelength is missing
RESPONSE: ...
;
# end Validation Reply Form
```

If you wish to submit your CIF for publication in Acta Crystallographica Section C or E, you should upload your CIF via the web. If you wish to submit your CIF for publication in IUCrData you should upload your CIF via the web. If your CIF is to form part of a submission to another IUCr journal, you will be asked, either during electronic submission or by the Co-editor handling your paper, to upload your CIF via our web site.

PLATON version of 13/07/2021; check.def file version of 13/07/2021

Datablock I - ellipsoid plot

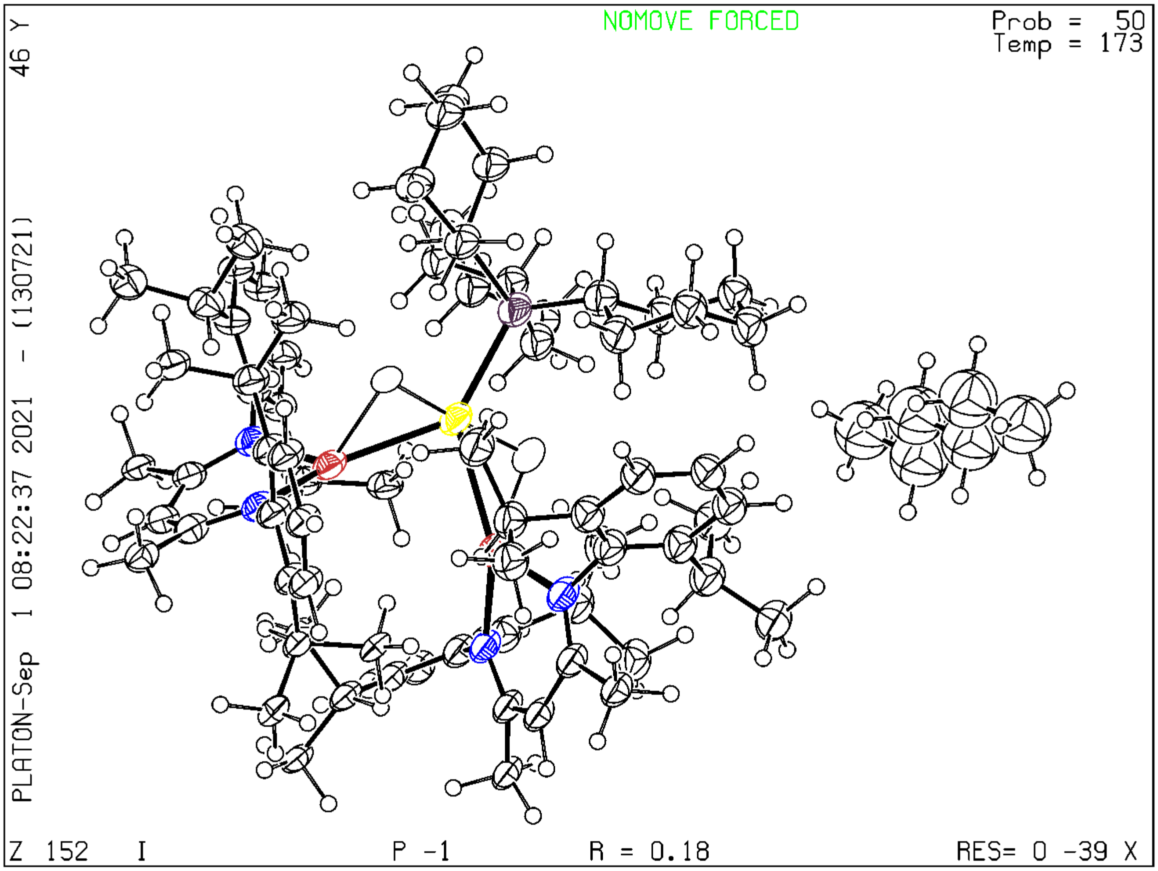

Supplement: Supplementary file 4 — Supporting Information [file ANIE-61-0-s004.pdf]
