# Supplementary material for: The Continuum Between Hexagonal Planar and Trigonal Planar Geometries
Source: Angew Chem Int Ed Engl. 2022 Oct 5;61(44):e202211948. doi: 10.1002/anie.202211948 (PMC9828084; doi:10.1002/anie.202211948)

checkCIF/PLATON report

Structure factors have been supplied for datablock(s) 2a, 4, 5, 6b

THIS REPORT IS FOR GUIDANCE ONLY. IF USED AS PART OF A REVIEW PROCEDURE FOR PUBLICATION, IT SHOULD NOT REPLACE THE EXPERTISE OF AN EXPERIENCED CRYSTALLOGRAPHIC REFEREE.

No syntax errors found. CIF dictionary Interpreting this report

Datablock: 2a

Bond precision: C-C = 0.0051 A Wavelength=1.54184

Cell:	a=11.9440(4)	b=12.0368(6)	c=22.8595(9)
	alpha=91.871(4)	beta=104.635(3)	gamma=112.172(4)
Temperature:	173 K		

	Calculated	Reported
Volume	2914.5(2)	2914.5(2)
Space group	P -1	P -1
Hall group	-P 1	-P 1
Moiety formula	C55 H90 Mg N2 P2 Pd, 0.5(C6 H14)	C55 H90 Mg N2 P2 Pd, 0.5(C6 H14)
Sum formula	C58 H97 Mg N2 P2 Pd	C58 H97 Mg N2 P2 Pd
Mr	1015.03	1015.02
Dx,g cm-3	1.157	1.157
Z	2	2
Mu (mm-1)	3.444	3.444
F000	1094.0	1094.0
F000'	1097.90	
h,k,lmax	14,14,28	14,14,28
Nref	11727	11034
Tmin,Tmax	0.343,0.930	0.405,0.928
Tmin'	0.178	

Correction method= # Reported T Limits: Tmin=0.405 Tmax=0.928
AbsCorr = ANALYTICAL

Data completeness= 0.941 Theta(max)= 73.483

R(reflections)= 0.0353(9204) wR2(reflections)= 0.0893(11034)

S = 1.017 Npar= 613

The following ALERTS were generated. Each ALERT has the format
test-name_ALERT_alert-type_alert-level.
Click on the hyperlinks for more details of the test.

Alert level C

```
PLAT222_ALERT_3_C NonSolvent Resd 1  H   Uiso(max)/Uiso(min) Range        4.6 Ratio
PLAT911_ALERT_3_C Missing FCF Refl Between Thmin & STh/L=    0.600        213 Report
```

Alert level G

```
PLAT002_ALERT_2_G Number of Distance or Angle Restraints on AtSite         12 Note
PLAT003_ALERT_2_G Number of Uiso or Uij Restrained non-H Atoms ...         12 Report
PLAT172_ALERT_4_G The CIF-Embedded .res File Contains DFIX Records          4 Report
PLAT178_ALERT_4_G The CIF-Embedded .res File Contains SIMU Records          1 Report
PLAT302_ALERT_4_G Anion/Solvent/Minor-Residue Disorder (Resd  2  )       100% Note
PLAT302_ALERT_4_G Anion/Solvent/Minor-Residue Disorder (Resd  3  )       100% Note
PLAT303_ALERT_2_G Full Occupancy Atom H1        with # Connections       2.00 Check
PLAT304_ALERT_4_G Non-Integer Number of Atoms in ..... (Resd  2  )       5.22 Check
PLAT304_ALERT_4_G Non-Integer Number of Atoms in ..... (Resd  3  )       4.78 Check
PLAT789_ALERT_4_G Atoms with Negative _atom_site_disorder_group  #         40 Check
PLAT860_ALERT_3_G Number of Least-Squares Restraints ............         43 Note
PLAT883_ALERT_1_G No Info/Value for _atom_sites_solution_primary .     Please Do !
PLAT910_ALERT_3_G Missing # of FCF Reflection(s) Below Theta(Min).          1 Note
PLAT912_ALERT_4_G Missing # of FCF Reflections Above STh/L=  0.600        480 Note
PLAT933_ALERT_2_G Number of HKL-OMIT Records in Embedded .res File         11 Note
PLAT941_ALERT_3_G Average HKL Measurement Multiplicity ..........         1.5 Low
PLAT978_ALERT_2_G Number C-C Bonds with Positive Residual Density.          5 Info
```

0 **ALERT level A** = Most likely a serious problem - resolve or explain
0 **ALERT level B** = A potentially serious problem, consider carefully
2 **ALERT level C** = Check. Ensure it is not caused by an omission or oversight
17 **ALERT level G** = General information/check it is not something unexpected

1 ALERT type 1 CIF construction/syntax error, inconsistent or missing data
5 ALERT type 2 Indicator that the structure model may be wrong or deficient
5 ALERT type 3 Indicator that the structure quality may be low
8 ALERT type 4 Improvement, methodology, query or suggestion
0 ALERT type 5 Informative message, check

Datablock: 4

Bond precision:	C-C = 0.0047 A	Wavelength=0.71073	
Cell:	a=14.6246(5)	b=16.6683(5)	c=26.2662(8)
	alpha=90	beta=103.526(3)	gamma=90
Temperature:	173 K		

	Calculated	Reported
Volume	6225.3(3)	6225.2(3)
Space group	P 21/c	P 1 21/c 1
Hall group	-P 2ybc	-P 2ybc
Moiety formula	C122 H184 N10 Pd2 Zn4, C6 H14	C122 H184 N10 Pd2 Zn4, C6 H14
Sum formula	C128 H198 N10 Pd2 Zn4	C128 H198 N10 Pd2 Zn4
Mr	2351.34	2351.23
Dx,g cm-3	1.254	1.254
Z	2	2
Mu (mm-1)	1.091	1.091
F000	2496.0	2496.0
F000'	2494.85	
h,k,lmax	19,22,34	18,21,34
Nref	15313	12392
Tmin,Tmax	0.901,0.926	0.959,1.000
Tmin'	0.858	

Correction method= # Reported T Limits: Tmin=0.959 Tmax=1.000
AbsCorr = MULTI-SCAN

Data completeness= 0.809 Theta(max)= 28.191

R(reflections)= 0.0378(8972) wR2(reflections)= 0.0790(12392)

S = 0.974 Npar= 680

The following ALERTS were generated. Each ALERT has the format
test-name_ALERT_alert-type_alert-level.
Click on the hyperlinks for more details of the test.

Alert level C

```
PLAT220_ALERT_2_C NonSolvent   Resd 1  C   Ueq(max)/Ueq(min) Range        3.7 Ratio
PLAT242_ALERT_2_C Low    'MainMol' Ueq as Compared to Neighbors of      C00O Check
PLAT242_ALERT_2_C Low    'MainMol' Ueq as Compared to Neighbors of      C013 Check
PLAT260_ALERT_2_C Large Average Ueq of Residue Including      C01Y     0.164 Check
PLAT360_ALERT_2_C Short  C(sp3)-C(sp3) Bond  C01Y     - C020     .      1.42 Ang.
PLAT910_ALERT_3_C Missing # of FCF Reflection(s) Below Theta(Min).        9 Note
PLAT911_ALERT_3_C Missing FCF Refl Between Thmin & STh/L=    0.600      135 Report
```

Alert level G

```
PLAT012_ALERT_1_G No       _shelx_res_checksum Found in CIF ......     Please Check
PLAT303_ALERT_2_G Full Occupancy Atom H9BA      with # Connections       2.00 Check
PLAT303_ALERT_2_G Full Occupancy Atom H0CA      with # Connections       2.00 Check
PLAT720_ALERT_4_G Number of Unusual/Non-Standard Labels .........       170 Note
PLAT883_ALERT_1_G No Info/Value for _atom_sites_solution_primary .     Please Do !
```

```
PLAT912_ALERT_4_G Missing # of FCF Reflections Above STh/L=  0.600       2707 Note
PLAT941_ALERT_3_G Average HKL Measurement Multiplicity ..........         1.7 Low
PLAT978_ALERT_2_G Number C-C Bonds with Positive Residual Density.          1 Info
```

---

0 **ALERT level A** = Most likely a serious problem - resolve or explain
0 **ALERT level B** = A potentially serious problem, consider carefully
7 **ALERT level C** = Check. Ensure it is not caused by an omission or oversight
8 **ALERT level G** = General information/check it is not something unexpected

2 ALERT type 1 CIF construction/syntax error, inconsistent or missing data
8 ALERT type 2 Indicator that the structure model may be wrong or deficient
3 ALERT type 3 Indicator that the structure quality may be low
2 ALERT type 4 Improvement, methodology, query or suggestion
0 ALERT type 5 Informative message, check

Datablock: 5

Bond precision: C-C = 0.0076 A Wavelength=1.54184

Cell:	a=25.5811(8)	b=13.9813(5)	c=45.002(3)
	alpha=90	beta=102.049(4)	gamma=90
Temperature:	173 K		

	Calculated	Reported
Volume	15740.7(13)	15740.7(14)
Space group	C 2/c	C 1 2/c 1
Hall group	-C 2yc	-C 2yc
Moiety formula	C75 H102 N6 Pd Zn3, C6 H14	C75 H102 N6 Pd Zn3, C6 H14
Sum formula	C81 H116 N6 Pd Zn3	C81 H116 N6 Pd Zn3
Mr	1476.37	1476.30
Dx,g cm-3	1.246	1.246
Z	8	8
Mu (mm-1)	3.175	3.175
F000	6240.0	6240.0
F000’	6216.09	
h,k,lmax	31,17,55	31,16,54
Nref	15865	15159
Tmin,Tmax	0.830,0.856	0.862,1.000
Tmin’	0.641	

Correction method= # Reported T Limits: Tmin=0.862 Tmax=1.000
AbsCorr = MULTI-SCAN

Data completeness= 0.955 Theta(max)= 73.516

R(reflections)= 0.0479(13010)

wR2(reflections)= 0.1108(15159)

S = 1.126 Npar= 873

The following ALERTS were generated. Each ALERT has the format
test-name_ALERT_alert-type_alert-level.
Click on the hyperlinks for more details of the test.

Alert level B

```
PLAT934_ALERT_3_B Number of (Iobs-Icalc)/Sigma(W) > 10 Outliers ..          6 Check
```

Author Response: A consequence of the orientation of the mounting of the crystal combined with the restrictions of the goniometer and the beam stop, it was not possible to collect intensity data for a small number of reflections. This has no significant effect on any aspect of the structure.

Alert level C

```
PLAT222_ALERT_3_C NonSolvent Resd 1   H   Uiso(max)/Uiso(min) Range        4.3 Ratio
PLAT234_ALERT_4_C Large Hirshfeld Difference C33      --C35A     .        0.16 Ang.
PLAT242_ALERT_2_C Low    'MainMol' Ueq as Compared to Neighbors of         C33 Check
PLAT250_ALERT_2_C Large U3/U1 Ratio for Average U(i,j) Tensor ....         2.3 Note
PLAT260_ALERT_2_C Large Average Ueq of Residue Including      C86S       0.141 Check
PLAT360_ALERT_2_C Short  C(sp3)-C(sp3) Bond  C89S     - C90S     .        1.43 Ang.
PLAT906_ALERT_3_C Large K Value in the Analysis of Variance ......       3.435 Check
PLAT911_ALERT_3_C Missing FCF Refl Between Thmin & STh/L=    0.600         165 Report
PLAT918_ALERT_3_C Reflection(s) with I(obs) much Smaller I(calc) .           1 Check
```

Alert level G

```
PLAT083_ALERT_2_G SHELXL Second Parameter in WGHT  Unusually Large       75.82 Why ?
PLAT171_ALERT_4_G The CIF-Embedded .res File Contains EADP Records           1 Report
PLAT301_ALERT_3_G Main Residue  Disorder .............(Resd  1  )           5% Note
PLAT303_ALERT_2_G Full Occupancy Atom H         with # Connections        2.00 Check
PLAT303_ALERT_2_G Full Occupancy Atom Ha        with # Connections        2.00 Check
PLAT303_ALERT_2_G Full Occupancy Atom Hb        with # Connections        2.00 Check
PLAT380_ALERT_4_G Incorrectly? Oriented X(sp2)-Methyl Moiety .....         C49 Check
PLAT380_ALERT_4_G Incorrectly? Oriented X(sp2)-Methyl Moiety .....         C76 Check
PLAT412_ALERT_2_G Short Intra XH3 .. XHn     H11B      ..H35D     .       2.04 Ang.
                                                        x,y,z  =      1_555 Check
PLAT412_ALERT_2_G Short Intra XH3 .. XHn     H16       ..H23B     .       2.08 Ang.
                                                        x,y,z  =      1_555 Check
PLAT412_ALERT_2_G Short Intra XH3 .. XHn     H28       ..H34A     .       2.08 Ang.
                                                        x,y,z  =      1_555 Check
PLAT720_ALERT_4_G Number of Unusual/Non-Standard Labels ..........           2 Note
PLAT910_ALERT_3_G Missing # of FCF Reflection(s) Below Theta(Min).           1 Note
PLAT912_ALERT_4_G Missing # of FCF Reflections Above STh/L=  0.600         470 Note
PLAT941_ALERT_3_G Average HKL Measurement Multiplicity ..........          1.7 Low
PLAT978_ALERT_2_G Number C-C Bonds with Positive Residual Density.           0 Info
```

```
 0 ALERT level A = Most likely a serious problem - resolve or explain
 1 ALERT level B = A potentially serious problem, consider carefully
 9 ALERT level C = Check. Ensure it is not caused by an omission or oversight
16 ALERT level G = General information/check it is not something unexpected

 0 ALERT type 1 CIF construction/syntax error, inconsistent or missing data
12 ALERT type 2 Indicator that the structure model may be wrong or deficient
 8 ALERT type 3 Indicator that the structure quality may be low
 6 ALERT type 4 Improvement, methodology, query or suggestion
 0 ALERT type 5 Informative message, check
```

Datablock: 6b

Bond precision: C-C = 0.0062 A Wavelength=1.54184

Cell:	a=16.2798(5)	b=13.0600(5)	c=45.8501(10)
	alpha=90	beta=93.226(2)	gamma=90
Temperature:	173 K		

	Calculated	Reported
Volume	9732.9(5)	9732.9(5)
Space group	P 21/c	P 21/c
Hall group	-P 2ybc	-P 2ybc
Moiety formula	C87 H126 Mg3 N6 Pt [+ solvent]	C87 H126 Mg3 N6 Pt, C7 H8, C6 H14
Sum formula	C87 H126 Mg3 N6 Pt [+ solvent]	C100 H148 Mg3 N6 Pt
Mr	1523.96	1702.26
Dx,g cm-3	1.040	1.162
Z	4	4
Mu (mm-1)	3.172	3.220
F000	3216.0	3616.0
F000’	3206.13	
h,k,lmax	20,16,57	20,15,55
Nref	19601	18623
Tmin,Tmax	0.812,0.876	0.592,0.890
Tmin’	0.324	

Correction method= # Reported T Limits: Tmin=0.592 Tmax=0.890
AbsCorr = ANALYTICAL

Data completeness= 0.950 Theta(max)= 73.505

R(reflections)= 0.0376(14088)   wR2(reflections)= 0.0897(18623)

S = 0.982   Npar= 951

The following ALERTS were generated. Each ALERT has the format
test-name_ALERT_alert-type_alert-level.
Click on the hyperlinks for more details of the test.

Alert level C

```
PLAT220_ALERT_2_C NonSolvent   Resd 1  C   Ueq(max)/Ueq(min) Range       5.4 Ratio
PLAT222_ALERT_3_C NonSolvent Resd 1  H   Uiso(max)/Uiso(min) Range       5.5 Ratio
PLAT242_ALERT_2_C Low    'MainMol' Ueq as Compared to Neighbors of       C15 Check
PLAT242_ALERT_2_C Low    'MainMol' Ueq as Compared to Neighbors of       C57 Check
PLAT911_ALERT_3_C Missing FCF Refl Between Thmin & STh/L=    0.600       230 Report
```

Alert level G

```
FORMU01_ALERT_2_G  There is a discrepancy between the atom counts in the
            _chemical_formula_sum and the formula from the _atom_site* data.
            Atom count from _chemical_formula_sum:C100 H148 Mg3 N6 Pt1
            Atom count from the _atom_site data:  C87 H126 Mg3 N6 Pt1
CELLZ01_ALERT_1_G Difference between formula and atom_site contents detected.
CELLZ01_ALERT_1_G ALERT: Large difference may be due to a
            symmetry error - see SYMMG tests
           From the CIF: _cell_formula_units_Z    4
           From the CIF: _chemical_formula_sum  C100 H148 Mg3 N6 Pt
           TEST: Compare cell contents of formula and atom_site data

           atom    Z*formula  cif sites diff
           C        400.00    348.00   52.00
           H        592.00    504.00   88.00
           Mg        12.00     12.00    0.00
           N         24.00     24.00    0.00
           Pt         4.00      4.00    0.00
PLAT002_ALERT_2_G Number of Distance or Angle Restraints on AtSite         21 Note
PLAT003_ALERT_2_G Number of Uiso or Uij Restrained non-H Atoms ...         16 Report
PLAT041_ALERT_1_G Calc. and Reported SumFormula    Strings  Differ     Please Check
PLAT051_ALERT_1_G Mu(calc) and Mu(CIF) Ratio Differs from 1.0 by .       1.50 %
PLAT176_ALERT_4_G The CIF-Embedded .res File Contains SADI Records          9 Report
PLAT178_ALERT_4_G The CIF-Embedded .res File Contains SIMU Records          3 Report
PLAT301_ALERT_3_G Main Residue  Disorder .............(Resd  1  )          9% Note
PLAT303_ALERT_2_G Full Occupancy Atom H1        with # Connections       3.00 Check
PLAT303_ALERT_2_G Full Occupancy Atom H2        with # Connections       3.00 Check
PLAT303_ALERT_2_G Full Occupancy Atom H3        with # Connections       3.00 Check
PLAT412_ALERT_2_G Short Intra XH3 .. XHn     H4B       ..H14A     .      1.97 Ang.
                                                      x,y,z  =      1_555 Check
PLAT412_ALERT_2_G Short Intra XH3 .. XHn     H5C       ..H29A     .      2.01 Ang.
                                                      x,y,z  =      1_555 Check
PLAT412_ALERT_2_G Short Intra XH3 .. XHn     H8A       ..H14F     .      2.14 Ang.
                                                      x,y,z  =      1_555 Check
PLAT412_ALERT_2_G Short Intra XH3 .. XHn     H22A      ..H28B     .      1.95 Ang.
                                                      x,y,z  =      1_555 Check
PLAT412_ALERT_2_G Short Intra XH3 .. XHn     H38A      ..H44F     .      1.89 Ang.
                                                      x,y,z  =      1_555 Check
```

```
PLAT606_ALERT_4_G Solvent Accessible VOID(S) in Structure ........          ! Info
PLAT860_ALERT_3_G Number of Least-Squares Restraints .............         72 Note
PLAT869_ALERT_4_G ALERTS Related to the Use of SQUEEZE  Suppressed          ! Info
PLAT883_ALERT_1_G No Info/Value for _atom_sites_solution_primary .     Please Do !
PLAT910_ALERT_3_G Missing # of FCF Reflection(s) Below Theta(Min).          2 Note
PLAT912_ALERT_4_G Missing # of FCF Reflections Above STh/L=  0.600        671 Note
PLAT933_ALERT_2_G Number of HKL-OMIT Records in Embedded .res File         12 Note
PLAT941_ALERT_3_G Average HKL Measurement Multiplicity ...........        1.6 Low
PLAT952_ALERT_5_G Calculated (ThMax) and CIF-Reported Lmax Differ.          2 Units
PLAT958_ALERT_1_G Calculated (ThMax) and Actual (FCF) Lmax Differ.          2 Units
PLAT978_ALERT_2_G Number C-C Bonds with Positive Residual Density.          0 Info
```

```
   0 ALERT level A = Most likely a serious problem - resolve or explain
   0 ALERT level B = A potentially serious problem, consider carefully
   5 ALERT level C = Check. Ensure it is not caused by an omission or oversight
  29 ALERT level G = General information/check it is not something unexpected

   6 ALERT type 1 CIF construction/syntax error, inconsistent or missing data
  16 ALERT type 2 Indicator that the structure model may be wrong or deficient
   6 ALERT type 3 Indicator that the structure quality may be low
   5 ALERT type 4 Improvement, methodology, query or suggestion
   1 ALERT type 5 Informative message, check
```

It is advisable to attempt to resolve as many as possible of the alerts in all categories. Often the minor alerts point to easily fixed oversights, errors and omissions in your CIF or refinement strategy, so attention to these fine details can be worthwhile. In order to resolve some of the more serious problems it may be necessary to carry out additional measurements or structure refinements. However, the purpose of your study may justify the reported deviations and the more serious of these should normally be commented upon in the discussion or experimental section of a paper or in the "special_details" fields of the CIF. checkCIF was carefully designed to identify outliers and unusual parameters, but every test has its limitations and alerts that are not important in a particular case may appear. Conversely, the absence of alerts does not guarantee there are no aspects of the results needing attention. It is up to the individual to critically assess their own results and, if necessary, seek expert advice.

Publication of your CIF in IUCr journals

A basic structural check has been run on your CIF. These basic checks will be run on all CIFs submitted for publication in IUCr journals (*Acta Crystallographica*, *Journal of Applied Crystallography*, *Journal of Synchrotron Radiation*); however, if you intend to submit to *Acta Crystallographica Section C* or *E* or *IUCrData*, you should make sure that full publication checks are run on the final version of your CIF prior to submission.

Publication of your CIF in other journals

Please refer to the *Notes for Authors* of the relevant journal for any special instructions relating to CIF submission.

PLATON version of 20/01/2022; check.def file version of 19/01/2022

Datablock 2a - ellipsoid plot

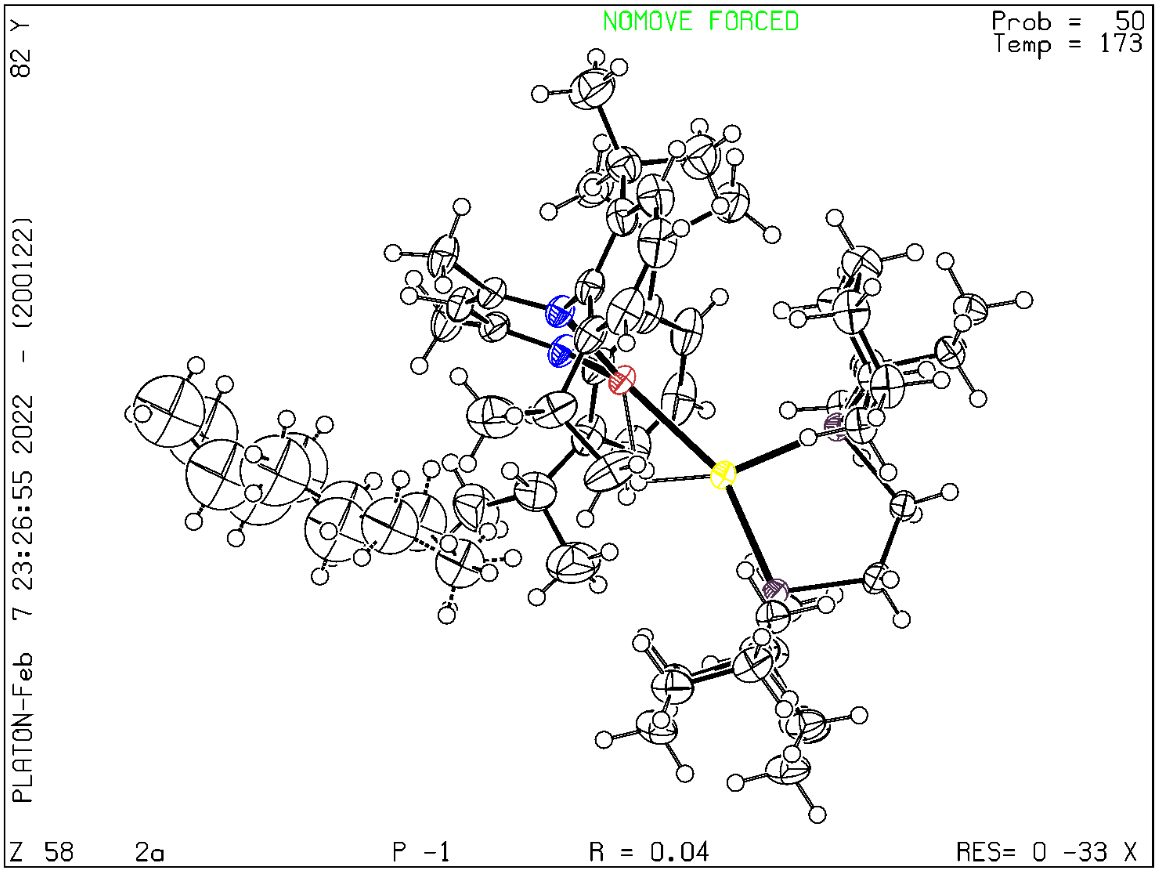

Datablock 4 - ellipsoid plot

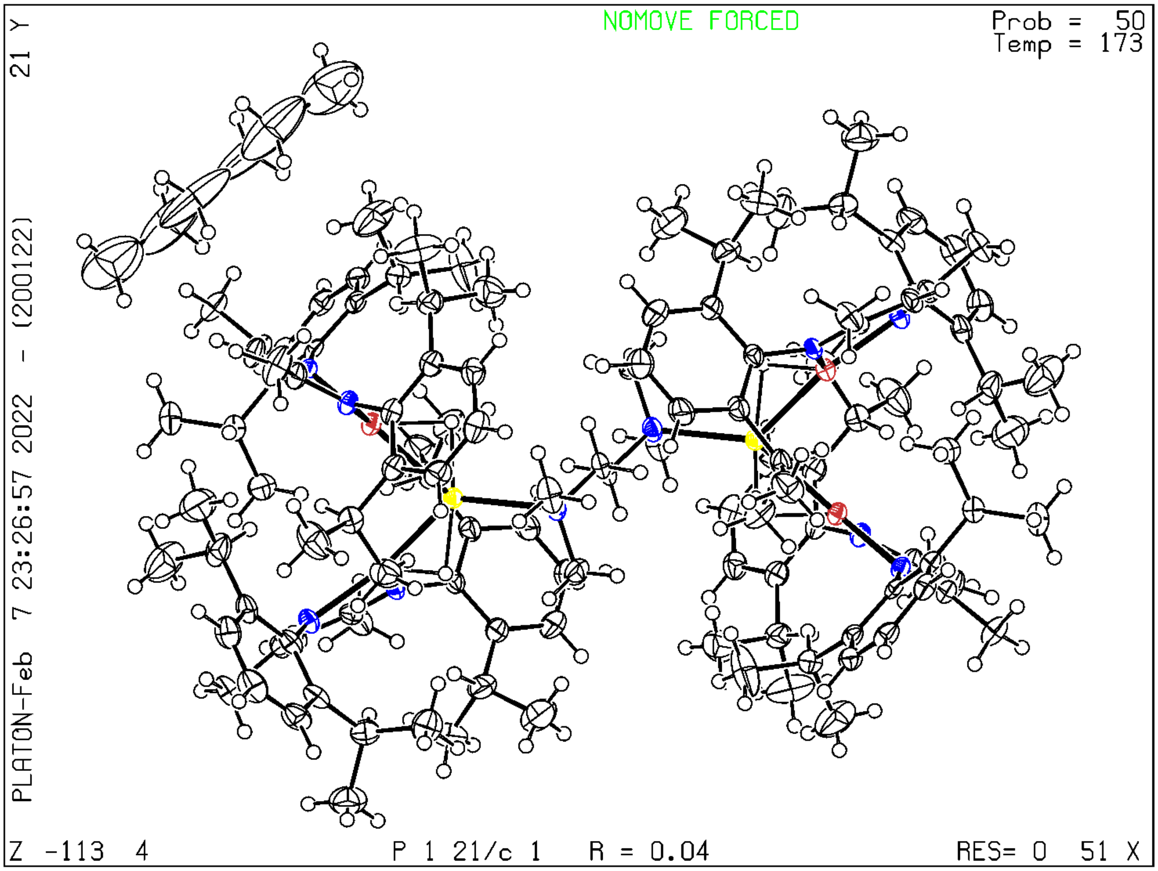

Datablock 5 - ellipsoid plot

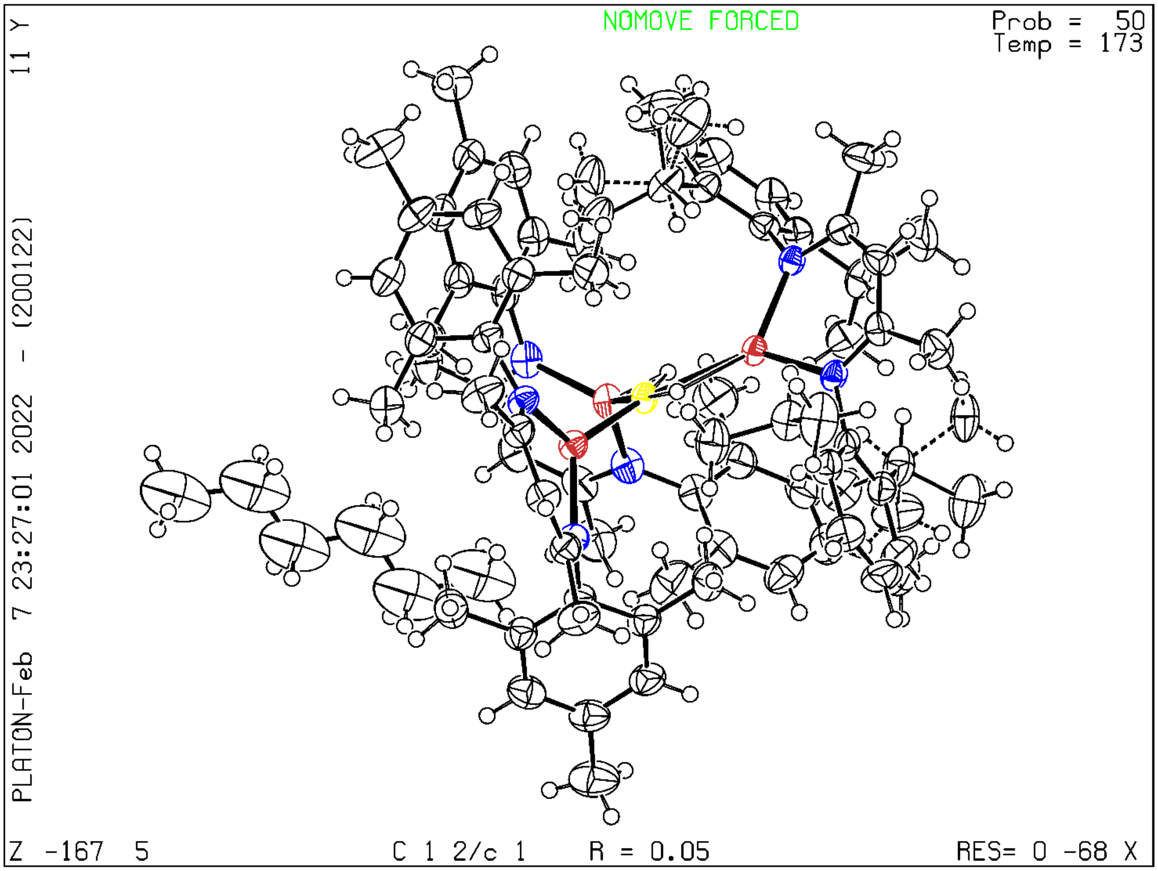

Datablock 6b - ellipsoid plot

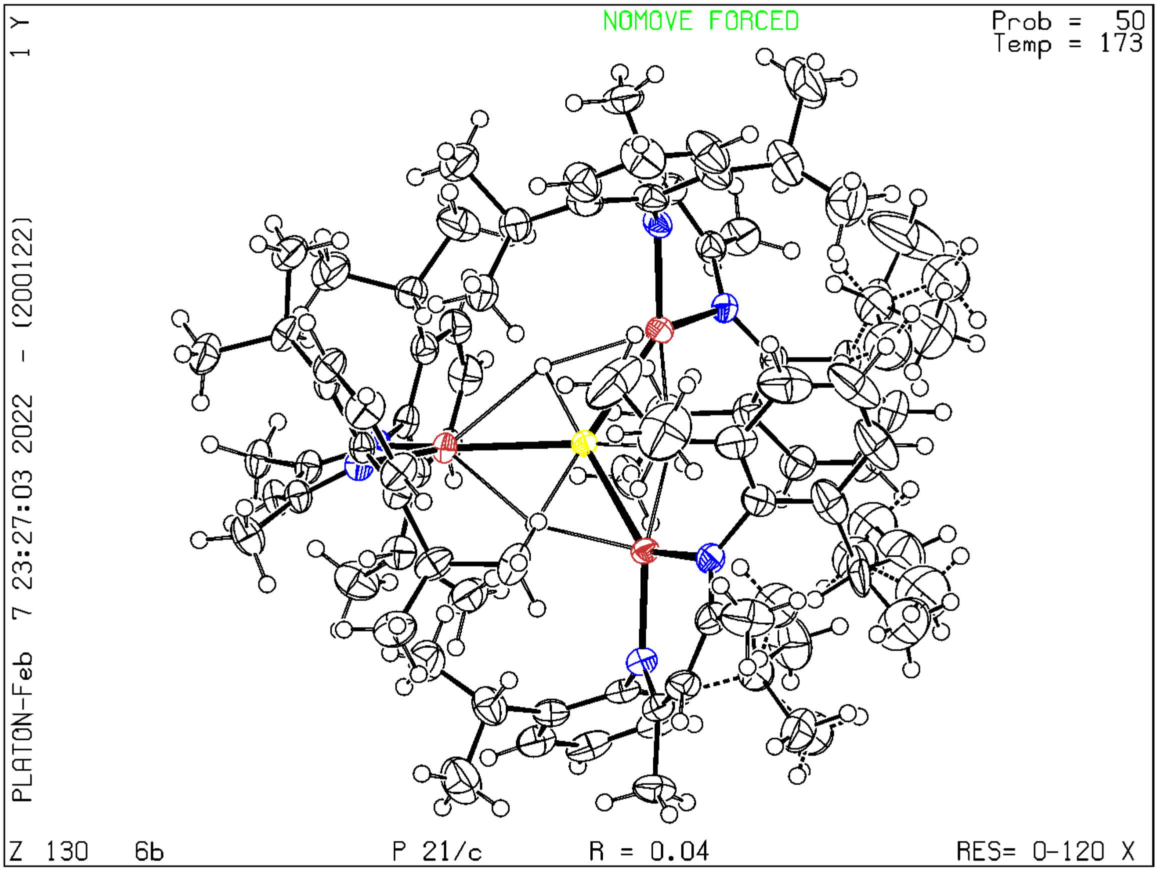

Supplement: Supplementary file 6 — Supporting Information [file ANIE-61-0-s001.pdf]
